# Rational Design of a Facially Coordinating *P,N,N* Ligand for Manganese‐Catalysed Enantioselective Hydrogenation of Cyclic Ketones

**DOI:** 10.1002/anie.202212479

**Published:** 2022-12-08

**Authors:** Conor L. Oates, Alister S. Goodfellow, Michael Bühl, Matthew L. Clarke

**Affiliations:** ^1^ EaStCHEM School of Chemistry University of St Andrews Purdie Building North Haugh St Andrews, KY16 9ST UK

**Keywords:** Asymmetric Reduction, Chirality, Computational Design, Earth Abundant Metals, Pincer Ligands

## Abstract

DFT calculations on the full catalytic cycle for manganese catalysed enantioselective hydrogenation of a selection of ketones have been carried out at the PBE0‐D3_PCM_//RI‐BP86_PCM_ level. Mn complexes of an enantiomerically pure chiral *P,N,N* ligand have been found to be most reactive when adopting a facial coordination mode. The use of a new ligand with an *ortho‐*substituted dimethylamino‐pyridine motif has been calculated to completely transform the levels of enantioselectivity possible for the hydrogenation of cyclic ketones relative to the first‐generation Mn catalysts. *In silico* evaluation of substrates has been used to identify those likely to be reduced with high enantiomer ratios (*er)*, and others that would exhibit less selectivity; good agreements were then found in experiments. Various cyclic ketones and some acetophenone derivatives were hydrogenated with *er's* up to 99 : 1.

## Graphical abstract

A Mn catalyst has been rationally redesigned to repel sp^3^hybridised substituents while attracting sp^2^ substituents, and hence enable enantioselective hydrogenation of cyclic ketones. DFT calculations enable a robust prediction of the level of enantiocontrol across a range of substrates.

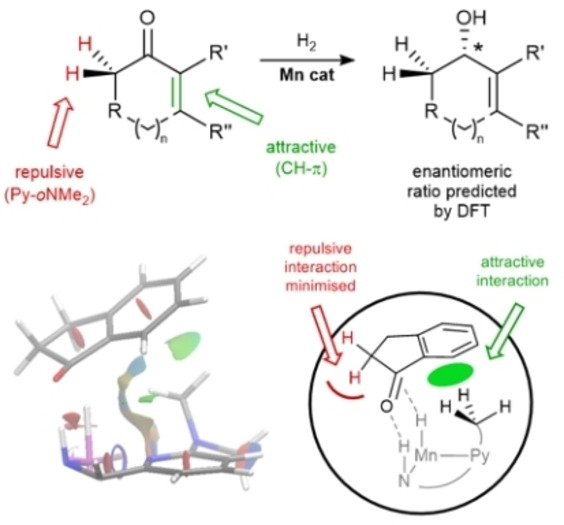

Ruthenium and iridium catalysed hydrogenation and transfer hydrogenations are core methods for making chiral alcohols. After the key early discoveries, there has been an extensive and fertile period where further exploration has revealed more and more applications of these types of catalyst.^[1]^ This includes both identifying new opportunities and solving known problematic reactions. This has overlapped with the early stages of a current major topic of research effort focused on developing the reactivity of earth abundant metal hydrogenation catalysts;[^2^, ^3^, ^4^] manganese catalysts[^3^, ^4^, ^5^] are very promising and of high current interest. The advantages of Mn catalysts are well‐known; an indefinitely sustainable supply of Mn salts are available for the catalysis industry, whilst also presenting lower toxicity concerns than precious metals. The latter feature can potentially lower energy consumption and waste in fine chemical batch‐type processes. Mn reduction catalysts will only truly compete with the precious metal hydrogenation chemistry if it proves possible to use Mn catalysts for a wide range of substrates and applications, and indeed refine catalysts towards a specific application. Our project in manganese hydrogenation catalysis began by examining a range of *P,N,X* (X=NH_2_, NRH, heterocycle, OR) ligands, initially designed for Ru and Ir catalysis,^[6]^ in Mn catalysed reduction of ketones and esters. Key impetus for studying manganese was provided by the report of achiral Mn/PNP catalysts of Beller and co‐workers.^[3a]^ One of us developed catalyst **1**, which is unusually reactive in hydrogenation of ketones, aswell as being quite enantioselective especially for ketones with a combination of aromatic group and secondary or tertiary alkyl group.^[4a]^ This work was followed up by some kinetic studies that successfully identified catalysts (Figure 1, catalysts **2** and **3**) with better physical properties, faster rates and optimised milder, inexpensive promoter conditions.[^4b^, ^4c^] This general ligand class of *P,N,N* ligand derived from chiral ferrocenes have also been applied in other Mn catalysed reactions.^[5]^ There has also been a collection of interesting papers reporting Mn catalysts for enantioselective hydrogenation, with just a few catalysts pictured in Figure 1 for brevity.[^3^, ^4^, ^5^] In terms of enantioselective ketone hydrogenation, there were various limitations in substrate scope. We felt this catalyst family merited our focus due to offering good reactivity for a range of substrates, and that further mechanistic knowledge would be the best way to unleash enhanced substrate scope in highly enantioselective hydrogenations. Here we show how DFT calculations of the catalytic cycle have been found to be robust enough to enable *in silico* evaluation of substrates, and report a new catalyst that gives high enantioselectivity for a range of important substrates.

**Figure 1:**
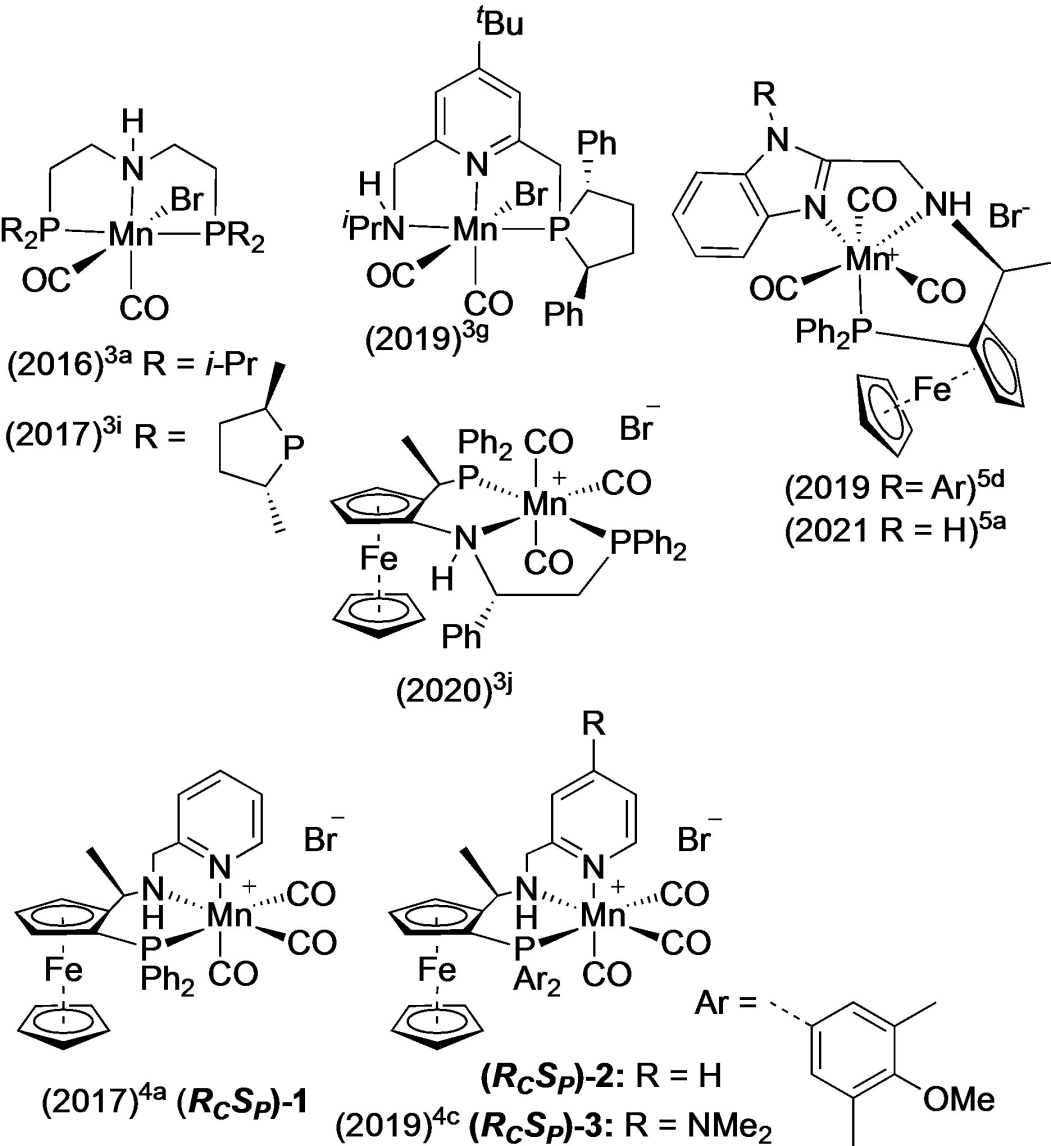
Selected Mn hydrogenation catalysts.

One key mechanistic question that was unanswered is if the ligand retains its facial coordination mode, observed as the major isomer for the pre‐catalyst,^[4c]^ through to the hydride transfer step and, if this is significant for reactivity. Following on from this, catalytic cycles including the key transition states for hydride transfer can be calculated for both known and new catalysts in this family. If such calculations show strong agreement with experiment, we envisaged it leading to the exciting prospect of using *in silico* catalyst design to identify the most suitable catalyst structure and suitable types of substrate.^[7]^ Since it is known from extensive literature that metal‐catalysed hydrogenations have relatively broad functional group tolerance, and success in one shape of substrate generally leads to similar performance for other similarly shaped substrates, we would argue this is a needed step‐forward in approach from extensive substrate testing. We also distinguish this integrated approach of prior evaluation *in silico* from the more widespread use of DFT to post‐rationalise an outcome from one experiment. Here we show that this rational‐design approach has been highly successful and offers solutions to some unresolved challenges in earth abundant metal reduction catalysis.

The facial nature of the pre‐catalyst was backed up by X‐ray crystallographic studies of the proto‐type catalyst, **1**.^[4a]^ DFT calculations (PBE0‐D3_PCM_//RI‐BP86_PCM_ level, benchmarked against first‐row transition metal hydricities)^[8]^ for the bare cation (without bromide) confirm the facial coordination mode is the most stable one by 4–8 kcal mol^−1^ (see Supporting Information). As noted in ref. [4c], however, the pre‐catalyst structure does not mean that this facial coordination mode must persist during catalysis, although partial ligand dissociation from this stable species would be expected to have a significant energetic cost. DFT calculations on the Mn‐hydride that would form as the active catalyst reveal that meridional isomer (*R*_*C,*_ *S_P_,S_N_*)‐1H‐*mer* is significantly higher in energy, but the (*R*_*C,*_ *S_P_,R_N_*)‐1H‐*mer* isomer is slightly more stable (Figure 2). However, this places the Mn–H bond parallel to the bulky ferrocene unit and prevents the approach of any ketone from above Mn–H (topographic steric maps of the Mn−H site are shown in Figure 2).

**Figure 2:**
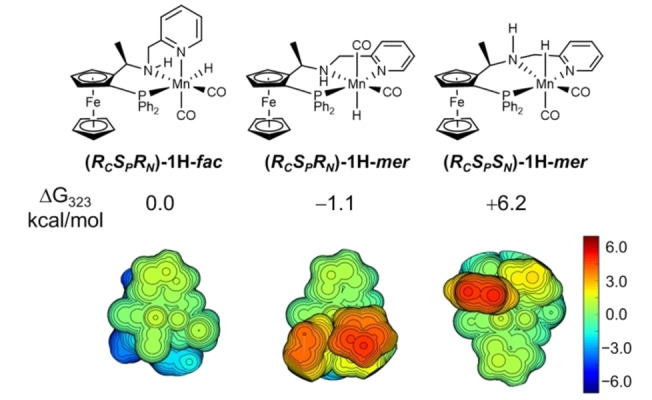
Relative energies of possible structural isomers of the active catalyst. DFT calculations were carried out at the level of PBE0‐D3_PCM(EtOH)_/def2TZVP//RI‐BP86_PCM(EtOH)_/def2SVP. Steric maps of the Mn‐hydride region for the competing isomers of catalyst.

Regarding the relative energies of the hydride transfer transition states for the reaction of Mn‐hydride with benzophenone, it can be seen that: each of the meridional coordination modes are disfavoured by >9 kcal mol^−1^ relative to the facial coordination mode as in (*R*_*C,*_ *S*_*P,*_ *R_N_*)‐1H‐*fac* (see Supporting Information, Figure S1).

The ketone structure‐performance relationships we reported in ref. [4c], combined with the transition states for reduction calculated here, (see Supporting Information, Figure S2) make it very likely that enantioselective ketone hydrogenations with this type of *P,N,N* ligand rely predominantly on a π‐stacking interaction between the substrate's aromatic ring and the pyridine of catalysts **1**–**3**. A recent paper also proposed and validated this type of interaction by DFT calculations between a quinoline substrate and catalyst with a similar backbone but benzimidazole in place of pyridine ligand.^[6a]^ Our design idea was to consider a broader class of substrates where one side of the ketone had an sp^3^ alkyl group and the other side a less 3‐dimensional substituent, including any sp^2^ carbon group. We felt the desired catalyst would include a more sterically hindered pyridine, which could repel the more 3‐dimensional ketone substituent. This might improve upon the selectivity exhibited by catalysts **1** to **3** for some substrates, but also expand the scope to substrates for which asymmetric reductions are rarely very successful (e.g. cyclohexenones). The choice of substituted pyridine needs to be synthetically accessible, but also needs to lead to a ligand that can still coordinate strongly to Mn. Since the mesomeric effect of amino groups on pyridines should enhance the ligand donor strength, and indeed since the *para*‐dimethylaminopyridine containing catalyst, **(*R***_***C***_ ***,S***_***P***_ **)‐3** was especially active, the *ortho*‐dimethyl amino substituted ligand, **(*R***_***C***_ ***,S***_***P***_ **)‐L4** was selected as a new catalyst (Scheme 1). We found we can either make catalysts *in situ* or use an isolated catalyst, **(*R***_***C***_ ***,S***_***P***_ **)‐4**, with similar results (Table 1, entries 2a and 2b), so most of the studies discussed here are carried out using more convenient in situ catalysts. Geometric isomers of the new *ortho*‐substituted catalyst, **(*Rc,Sp*)‐4**, have also been considered and follow the same reactivity to that of catalyst **1** in Figure 2 (see Supporting Information Section 2.3)

**Scheme 1:**
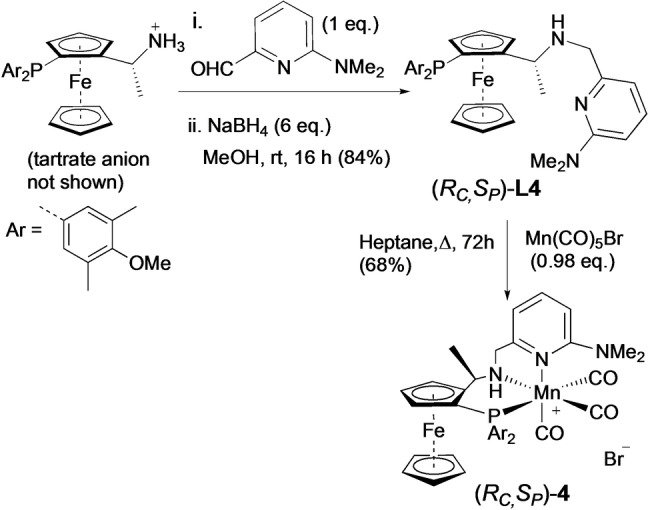
Synthesis of new ligand and catalyst proposed to favour sp^2^ centres relative to sp^3^ centres being located above the pyridyl substituent in the stereo‐determining step. The enantiomer, (*S*_*C,*_ *R_P_*)‐**4** is also prepared similarly.

**Table 1:** Comparison between experimental and computed er values for the Mn‐catalysed hydrogenation of ketones with **L4**.

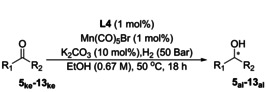					
Entry	Product [cpd. number]^[a]^	Conversion [Yield]^[b]^	*er* (*S : R*)^[c]^	Predicted ΔΔ^≠^ *G* (*S‐R*) [kcal mol^−1^	Predicted *er* (*S : R*)
1	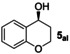	>99 [98]	99 : 1	−1.81	94 : 6
2a 2b	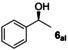	>99 [91]^[d]^ 90 [70]	92 : 8 92 : 8	−1.84 −1.84	95 : 5 95 : 5
3^[e]^	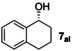	>99 [90]	2 : 98	1.84	5 : 95
4	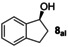	>99 [91]	97 : 3	−1.91	95 : 5
5^[e]^	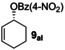	>99 [36]	5 : 95	1.87	5 : 95
6^[e,f]^	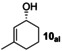	>99 [88]	6 : 94	0.50	31 : 69
7^[f]^	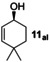	>99 [76]	93 : 7	−0.74	76 : 24
8^[e]^	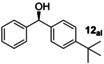	>99 [81]	32 : 68	0.39	35 : 65
9	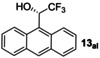	>99 [85]	64 : 36	−0.49	68 : 32

For conditions see equation unless otherwise noted. Catalyst is formed in situ using (*R*_*C,*_ *S_P_*)*‐* **L4** except where noted (footnotes d and e). [a] Determined by ^1^H NMR using 1,4‐dimethoxybenzene as internal standard. [b] Isolated yields. [c] Determined by chiral HPLC. [d] Isolated complex from complexation of **L4** to Mn(CO)_5_Br used. [e] (*S*_*C,*_ *R_P_*) enantiomer of **L4** used.[f] 1.5 mol% **L4**/Mn(CO)_5_Br used.

Within the sp^3^‐alkyl/sp^2^ substituted ketones, our plan was to focus on cyclic ketones as a ketone class that gives poor *er* with catalysts **1**–**3**. This includes especially challenging non‐aromatic cyclic ketones, but our initial model substrate for calculations was indanone. DFT calculations were carried out at the PBE0‐D3_PCM_//RI‐BP86_PCM_ level to see if the *ortho*‐dimethyl amino group might clash with certain ketone substituents. A full catalytic cycle has been computed and is presented in Figure 3, taking indanone, **8 ke** as our model example.

**Figure 3:**
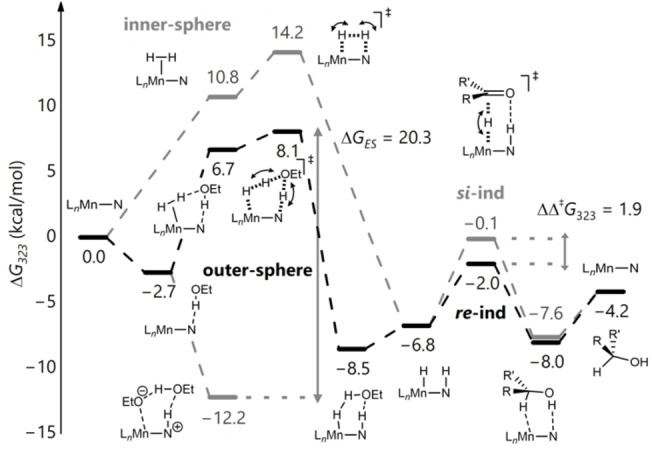
Potential free energy surface (Δ*G*_323_, kcal mol^−1^) for the hydrogenation of indanone using the *ortho*‐substituted catalyst **4**.

The reaction is indicated to proceed via two steps, the first being H_2_ activation, which is shown to be rate‐determining, and the second being that of an asynchronous transfer of hydride and proton to the substrate (see Supporting Information). The latter step is key for determining the stereochemistry of the product. Consideration of the on‐cycle species leads to the prediction of a barrier of 12.4 kcal mol^−1^, which is low for a reaction that is performed experimentally at 25–50 °C. However, we were able to characterise computationally a number of low‐lying, off‐cycle intermediates that need to be taken into account when evaluating the overall energy‐span for the catalytic cycle. One such possible solvated species forms from two ethanol molecules reacting with the Mn‐amido species to give the solvated ethoxide species shown (at −12.2 kcal mol^−1^ in Figure 3). Consideration of this off‐cycle species affords a larger, more realistic energy span of 20.3 kcal mol^−1^. There are a range of related off‐cycle resting states possible, including some that are computed to be even more stable, but given the large excess of ethanol present and the high likelihood of a mixture of solvates being present, this was chosen as a representative resting state. The DFT study reveals that the cause of the stereochemical bias is more complex than being simply steric bulk in the *ortho*‐position; it arises from a combination of a rigid proton that can point directly towards a sterically encumbered area for the *si‐*face being disfavoured, and an attractive interaction if the alternative *re*‐face sits above the *ortho*‐dimethylamino group. Scheme 2 shows the favoured and disfavoured transition states for hydrogenation of indanone (ΔΔ^≠^ *G*_323_=1.91 kcal mol^−1^). This preference can be traced back to the difference in interaction energy of the catalyst and substrate between the diastereomers of the transition state (ΔΔ*E*_int_=−3.48 kcal mol^−1^) (see Supporting Information, Section 2.6).

**Scheme 2:**
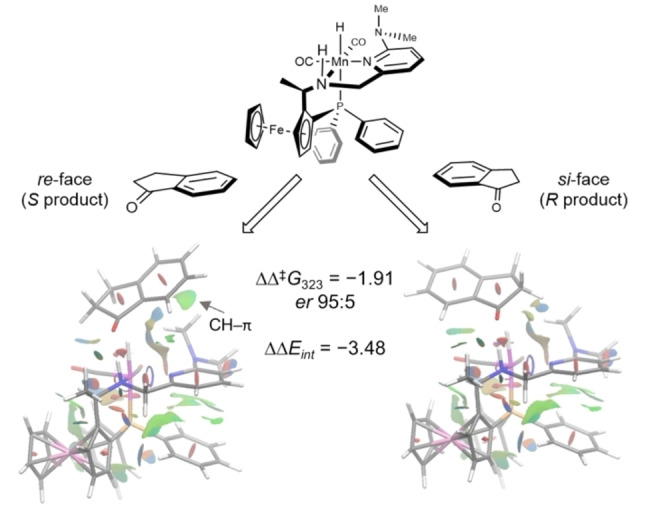
Comparison of the stereodefining transition states of indanone. Areas with attractive non‐covalent interactions shown in green.

The synthetic work and computational work were carried out in parallel and after the full cycle was determined (Figure 3), the robust nature of the calculations enabled us to evaluate substrates *in silico*, and assess the promise of substrates of being reduced enantioselectively using the new catalyst to a degree that is synthetically meaningful (Table 1 and Supporting Information Table S1). The predictions, based on the energetic differences between *R* and *S* pathways, can be simplified as: (i) low selectivity (ΔΔ^≠^ *G* ≈0‐0.5 kcal mol^−1^), (ii) moderate selectivity (ΔΔ^≠^ *G* ≈ 0.5‐1.5 kcal mol^−1^), or (iii) high selectivity (ΔΔ^≠^ *G* > 1.5 kcal mol^−1^).

To our knowledge, cyclic non‐aromatic ketones have not been hydrogenated in high *er* by earth abundant metal catalysts; for example, reference [3i] reports an *er* of 75 : 25 for **10 ke**. When cyclohexenones **9**–**11 ke** approach catalyst **4**, one of the C−H bonds on the sp^3^ carbons points downwards towards the *ortho* substituent and hence represents a disfavoured hydride transfer transition state relative to one where the flat alkenyl group is positioned above this substituent where it benefits from an attractive C−H‐π interaction. Experimentally reduction of ketone **9 ke** is reduced to give **9 al** with an *er* of 5 : 95, but with a large amount of C=C reduction,^[9]^ while related substrate **10 ke** is reduced without C=C reduction with an *er* of 6 : 94. Indanone, **8 ke**, discussed earlier gives *er* of 97 : 3. Various substrates examined *in silico* and examined experimentally are shown in Table 1. Within the accepted errors of DFT calculations, especially predictions across a family of substrates, the agreement is remarkable, successfully grading the expected selectivities as: low, moderate or high, which we suggest is the accuracy needed for the experimentalist.

While there is some slight divergence between predicted relative energies and experimental results for ketones **10 ke** and **11 ke** the DFT approach still suggests the substrates merit experimental investigation and lower selectivity for **11 ke** (ΔΔ^≠^ *G*_323_=0.74 kcal mol^−1^) than ketone **8 ke** was predicted and observed (Fortunately in this case experimentally higher selectivity is observed than predicted [Table 1, Entries 4 compared to 6 and 7]).

Further examples of enantioselective reductions we envisaged to be of synthetic use are discussed later, but to further illustrate the value of prior evaluation of a possible target *in silico* with this system, a quite different substrate was considered. There has been repeated interest in the asymmetric hydrogenation of diaryl ketones to give chiral benzhydrols.^[10]^ Most examples are based on the steric differences between *ortho*‐substituted (hetero)aromatics and less substituted aromatics. While there are various elegant and ingenious ways to enhance the substrate scope of such reductions, to the best of our knowledge, there are no examples where the difference between a solely *para*‐substituted aromatic relative to a less substituted aromatic is the basis for enantioselective reduction.

Using a simplistic physical sketch or model, there seemed a *slight* possibility for enantioface binding of this type of ketone such that the substituted aromatic avoided the pyridine unit. The reduction of ketone **12 ke** with **(*R***_***C***_ ***S***_***P***_ **)‐4** was then evaluated as a model substrate *in silico*. This predicted however that this simple analysis was incorrect, and, while it was not totally unselective, it falls into the low selectivity range (ΔΔ^≠^ *G*=0.39 kcal mol^−1^). This was indeed the case experimentally and an *er* of 32 : 68 was recorded. In a similar way it was not initially obvious which enantiomer 9‐trifluoroacetyl‐anthracene would form; DFT calculations predicted only relatively low selectivity to the (*S*) enantiomer, and this was then seen experimentally. Being able to predict less successful reactions in addition to successful ones is, we suggest, a useful test of the robustness of the calculations.

Since the products of this type of reduction find various synthetic uses, and the catalyst shown here is so synthetically accessible, some structurally related ketones were also hydrogenated with high *er* with the results shown in Scheme 3. The DFT predictions from Table 1 also represent model substrates that enable the experimentalist to predict high selectivity for the reactions in Scheme 3 (with model substrates being recommended when substrates are computationally expensive to evaluate e.g. **21ke**). Some of the reactions in Scheme 3 have subsequently been successfully post‐rationalised with a discussion on this in the Supporting Information, Sections 2.6 & 2.7.

**Scheme 3:**
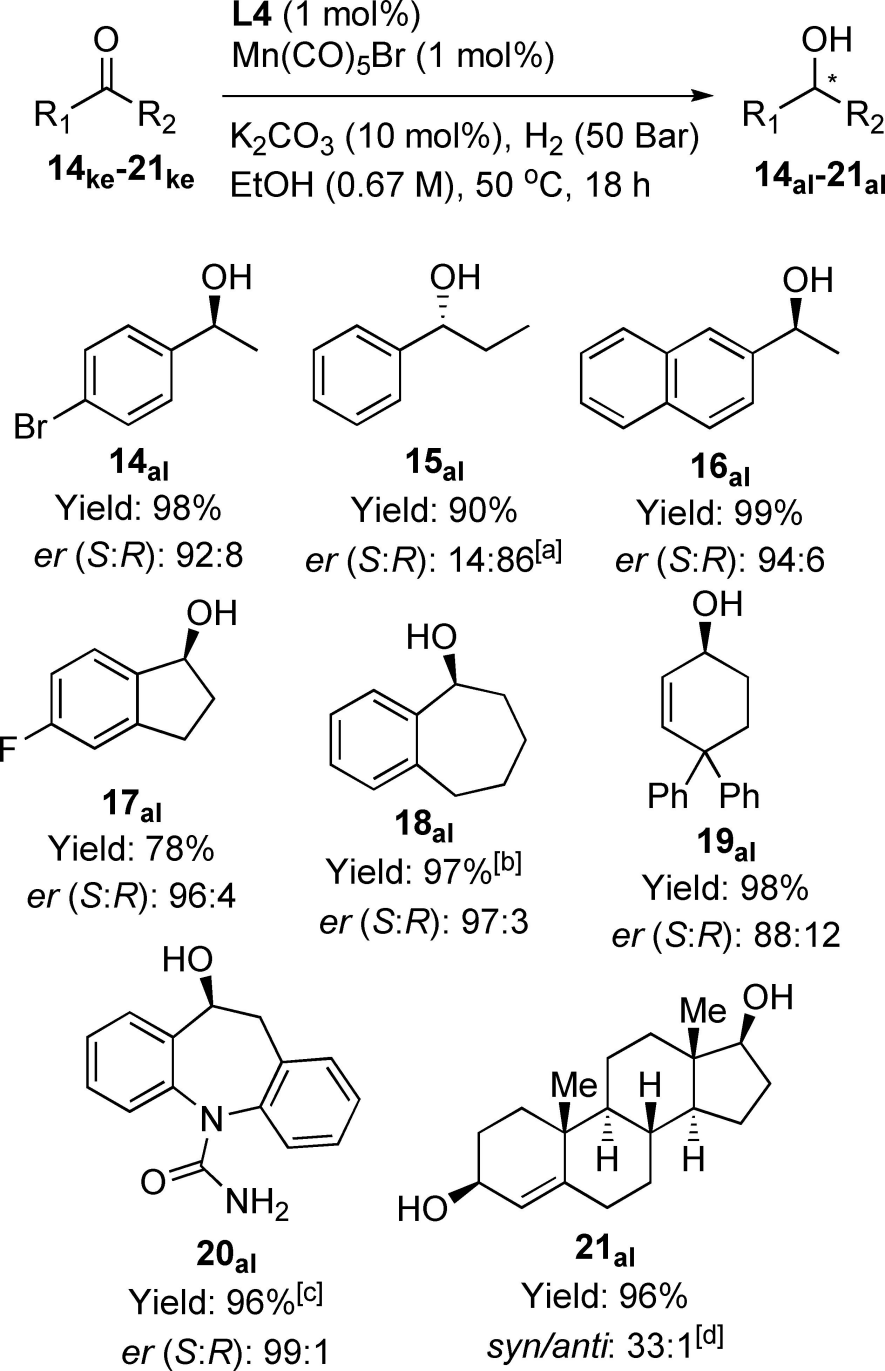
Enantioselective hydrogenation of some other ketone substrates using Mn/(*R*_*C,*_ *S_P_*)*‐* **L4** except **15 ke**. For conditions see equation unless otherwise noted. Yields are for isolated purified alcohols. Enantiomer ratio determined by chiral HPLC. [a] (*S*_*C,*_ *R_P_*)‐**L4** used. [b] 1.25 mol% **L4**/Mn(CO)_5_Br used. [c] 1.5 mol% **L4**/Mn(CO)_5_Br used. [d] Determined by ^1^H NMR.

It should be noted that very good results have already been obtained using a different phospholano‐amino‐pyridine Mn catalyst for aromatic cyclic ketones.^[3g]^ This includes tetralone and the drug precursor, **20 ke**.^[11]^ Two other acyclic aromatic substrates were also reduced with high *er*, suggesting various acetyl‐aromatics are likely good substrates with this catalyst. Testosterone was reduced with the expected diastereoselectivity that is consistent with the model described for the prochiral substrates.

In summary, the DFT method used to study Mn catalysts with chelating *P,N,N* ligands is sufficiently robust to enable the viability of a new member of our catalyst family to be validated against a range of new ketone substrates *in silico*. Predictions have predominantly been made for ketones that have one sp^3^ alkyl group and one sp^2^ flatter group; some of these are substrates that the first generation catalysts gave very low selectivity for: even indanone is reduced with almost no selectivity at all with the original catalysts **1** or **3**, but can now be reduced with high enantiomer ratio. Some of these reductions also represent unresolved challenges for asymmetric reduction catalysts in general, showing the value of the harnessing computational and synthetic chemistry in synergy. Mn/L**4** catalyst is derived from a ligand that is readily accessible from relatively cheap chiral starting materials available at significant scale, with the conditions of potassium carbonate in ethanol also being economic and benign. The use of manganese is likely to be more sustainable than precious metals, and could lead to less intensive purification processes to meet regulations for pharma ingredients. We believe this family of catalysts could be of significant use in the synthesis of various chiral molecules. Meanwhile, the ability presented here to rationally design catalysts and evaluate a selection of substrates *in silico* before considering experiments should also be enabling to not only the use of these catalysts, but the development of asymmetric Mn catalysts in a wide sense.

## Author Contributions

Mr C. L. Oates (experiments, joint first author), Mr A. S. Goodfellow (calculations, joint first author), Prof M. Bühl (calculations correspondence author), Prof. M. L. Clarke (main corresponding author).

## Conflict of interest

The authors declare no conflict of interest.

## Supporting information

As a service to our authors and readers, this journal provides supporting information supplied by the authors. Such materials are peer reviewed and may be re‐organized for online delivery, but are not copy‐edited or typeset. Technical support issues arising from supporting information (other than missing files) should be addressed to the authors.

Supporting Information

Supporting Information

Supporting Information

Supporting Information

Supporting Information

## Data Availability

The data underpinning this manuscript is openly available from the University of St Andrews PURE database (see ref. [12]).
