# Supplementary material for: Rational Design of a Facially Coordinating P,N,N Ligand for Manganese‐Catalysed Enantioselective Hydrogenation of Cyclic Ketones
Source: Angew Chem Int Ed Engl. 2022 Dec 8;62(3):e202212479. doi: 10.1002/anie.202212479 (PMC10107995; doi:10.1002/anie.202212479)
Supplement: Supplementary file 1 — Supporting Information [file ANIE-62-0-s003.pdf]

Angewandte Chemie
Eine Zeitschrift der Gesellschaft Deutscher Chemiker

Supporting Information

Rational Design of a Facially Coordinating *P,N,N* Ligand for Manganese-Catalysed Enantioselective Hydrogenation of Cyclic Ketones

C. L. Oates, A. S. Goodfellow, M. Bühl, M. L. Clarke**

Table of Contents:

S3- **Section 1.1**: General Information

S4-S7- **Section 1.2**: Preparation of Starting Materials, Ligands and Catalysts

S8-S9- **Section 1.3**: General Procedure for Manganese-Catalysed Hydrogenation of ketones using **L4**

S9-S16- **Section 1.4**: Characterisation Data for Alcohol Products

S16: **Section 1.5**: References

S17- **Section 2.1**: Computational Details

S17- **Section 2.2**: Derivation of SMHP Entropic Correction

S17-S19- **Section 2.3**: Coordination Mode of Tridentate Ligand

S20- **Section 2.4**: Transition State IRCs

S21- **Section 2.5**: Off-Cycle Species

S21-S24- **Section 2.6**: Origin of Stereoselectivity

S25-S26- **Section 2.7**: Discussion regarding further substrates that were considered

S26- **Section 2.8**: References

S27-S66- **Section 3.1**: NMR/ HPLC Data

S67-S189- **Section 3.2**: Cartesian Coordinates and Energetics of Optimised Structures

Section 1.1: General Information:

The preparation of solutions for the use in catalytic reactions were carried out under either argon or nitrogen atmospheres. All glassware was used oven dried or flame dried and cooled under vacuum before use. Unless otherwise stated, all chemicals were purchased from SigmaAldrich, Acros, Alfa Aesar, Strem or TCI and used as received. Substrates were not dried before use in catalysis reactions. Unless otherwise stated, solvents used were 'Extra Dry, AcroSeal™, ACROS Organics' purchased from Fisher Scientific. Room temperature refers to the temperature range 15- 25 °C. Ligand **L4**/ Complex were stored under dry inert gas and weighed in air. Heating the reaction mixtures was done with either an oil bath or a Drysyn heating block. Reported temperature is the oil bath or heating block temperature and not internal temperature. *In vacuo* refers to either the use of a Heidolph Laborota 4001 rotary evaporator or the use of a high-vacuum line. Analytical thin layer chromatography (TLC) was carried out on pre-coated plastic plates (Kieselgel 60 F254 silica). TLC visualization was carried out using a UV lamp (254nm) or using a 1% potassium permanganate aqueous solution. Flash silica chromatography was performed using Kieselgel 60 silica. ^{1}H, ^{13}C, ^{31}P, NMR was carried out using either a Bruker Avance II 400 (400 MHz ^{1}H, 100 MHz ^{13}C, 161 MHz ^{31}P or a Bruker Ultrashield 500 (500 MHz ^{1}H, 125 MHz ^{13}C, 202 MHz ^{31}P). NMR analyses were carried out at room temperature in deuterated solvent. The chemical shifts are quoted as parts per million (ppm). Coupling constants, J, are quoted in Hz. Multiplicities are indicated by: s (singlet), d (doublet), t (triplet), q (quartet) and m (multiplet). The abbreviation "b" is used to denote broad peak shape. Mass spectrometric (m/z) data were acquired by electrospray ionisation (ESI) or electron impact (EI) at the University of St Andrews Mass Spectrometry facility (using Micromass LCT spectrometer or Micromass GCT spectrometer). Values are reported as a ratio of mass to charge in Daltons. Optical rotations were measured on a Perkin Elmer 341 polarimeter using a 1 ml cell with a 1 dm path length at room temperature using the sodium D-line, and a suitable solvent that is reported along with the concentration (c = g/100ml). HPLC analysis was determined using a Varian Prostar operated by Galaxie workstation PC software. For chromatograms where peak baselines were not fully resolved the lowest point between the two peaks were chosen as the cut-off point. (S_cR_p) and (R_cS_p)-N-dimethyl-1-(2-bis(4-methoxy-3,5- dimethylphenyl)phosphino)ferrocenylethylamine was obtained from Solvias, Basel, Switzerland. Racemic alcohol samples were prepared as follows: corresponding ketone (1 eq.) was dissolved in MeOH (0.25 M) and then $NaBH_4$ (3 eq.) was added and the reaction stirred overnight at rt. The reaction was then quenched with water and extracted with CH_2Cl_2. The combined organic layers were washed with brine and then dried over Na_2SO_4. After filtering, the solvent was removed *in vacuuo* and the samples used without further purification.

Section 1.2: Preparation of Starting Materials, Ligands and Catalysts

Preparation of 2-dimethylamino-6-bromopyridine[1]

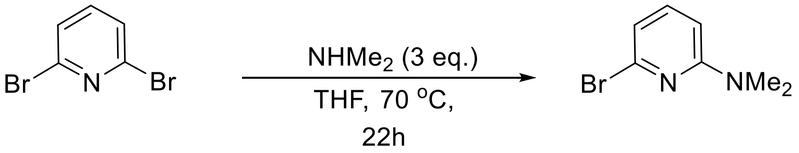

(Procedure adapted from ref. S1) To a screw cap vial equipped with magnetic stirrer bar equipped was added 2,6-dibromopyridine (1.19 g, 5 mmol, 1 eq.) followed by $NHMe_2$ (2M soln. in THF) (7.5 mL, 15 mmol, 3 eq.). The cap was tightly secured and the reaction stirred at 70 °C for 24 h. After completion of the reaction, the reaction was washed with water (40 mL) then the aqueous layer was extracted with EtOAc (3 x 20 mL). The combined organic layers were washed with brine, dried over Na_2SO_4, filtered and dried *in vacuo* to give:

2-dimethylamino-6-bromopyridine

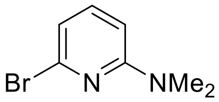

Yield (5 mmol Scale): 0.936 g (93%) of pale brown oil
^{1}H NMR (500 MHz, $CDCl_3$) δ 7.24 (1H, dd, *J* = 8.4, 7.4 Hz, Ar-H), 6.66 (1H, d, *J* = 7.4 Hz, Ar-H), 6.37 (1H, d, *J* = 8.4 Hz, Ar-H), 3.05 (6H, s, NMe_2). ^{13}C NMR (126 MHz, $CDCl_3$) δ 159.3 (Ar-Br), 140.2 (Ar-N), 139.2 (Ar-H), 114.3 (Ar-H), 103.9 (Ar-H), 38.0 (CH_3). HRMS (ESI+): Calculated for [$C_7H_{10}BrN_2$]: 201.0022 Found: 201.0024. Compound has been prepared previously using alternative route and data reported here is consistent with previously published data.[1]

Preparation of 2-dimethylamino-6-formylpyridine[2]

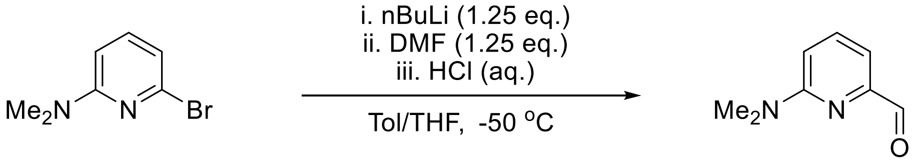

(Procedure adapted from ref. S2) A two-neck round bottom flask equipped with magnetic stirrer bar and capped with rubber septa was flame dried under vacuum. After cycling the flask between vacuum and Ar atmosphere, one rubber septum was replaced by a thermometer. Dry toluene (4 mL) was added *via* syringe. The toluene was cooled to -60 °C and *n*-BuLi (1.3 M soln. in Hexane) (1.9 mL, 2.5 mmol, 1.25 eq.) was added. Once the temperature of the solution reached -60 °C, a soln. of 2-dimethylamino-6-bromopyridine (402 mg, 2 mmol, 1 eq. in 2.7 mL dry THF) was slowly added ensuring the reaction temperature remained below -50 °C. The solution was stirred for 40 minutes at -50 °C and then DMF (0.20 mL, 2.5 mmol, 1.25 eq.) was added over a 2 minute period. The reaction was then allowed to warm to 15 °C and HCl (1M aq. Soln.) (3.2 mL, 3.2 mmol, 1.6 eq.) was carefully added. The reaction was then diluted with CH_2Cl_2 (25 mL), washed with sat. $NaHCO_3$ soln. (50 mL). The aqueous phase was extracted with CH_2Cl_2 (3 x 15 mL), the combined organic layers washed with brine and then dried over Na_2SO_4. After filtration and concentration *in vacuo*, the crude material was dissolved in CH_2Cl_2 and washed with NaOH (2M aq. Soln.) (2 x 50 mL). The organic layer was washed with brine, dried over Na_2SO_4, filtered and concentrated *in vacuo* and finally purified by flash column chromatography on silica gel using Hexane/ EtOAc (9:1) as eluent to give:

2-dimethylamino-6-formylpyridine

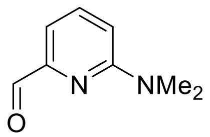

Yield (2 mmol scale): 200.2 mg (67%) of yellow oil
^{1}H NMR (400 MHz, $CDCl_3$) δ 9.88 (1H, s, HC=O), 7.55 (1H, dd, *J*= 7.2 Hz, 8.6Hz, Ar-H), 7.18 (1H, d, *J* = 7.2 Hz, Ar-H), 6.69 (1H, d, *J* = 8.6 Hz, Ar-H), 3.13 (6H, s, NMe_2). ^{13}C NMR (101 MHz, $CDCl_3$) δ 194.7 (C=O), 159.4 (Ar-C), 151.2 (Ar-N), 137.8 (Ar-H), 110.4 (Ar-H), 110.0 (Ar-H), 37.9 (CH_3). HRMS (ESI+): Calculated for [$C_8H_{11}N_2O$]: 151.0866 Found: 151.0865. Compound has been prepared previously using an alternative route and data reported here is consistent with previously published data.[3]

[(S_C,R_p)-1-(2-bis[4-methoxy-3, 5-dimethylphenyl]phosphino)ferrocenylethylamine L-tartrate salt was prepared using a previously reported synthesis starting from the commercially available (S_cR_p)-N-dimethyl-1-(2-bis(4-methoxy-3,5- dimethylphenyl)phosphino)ferrocenylethylamine.[4]

Preparation of S_cR_p-**L4**

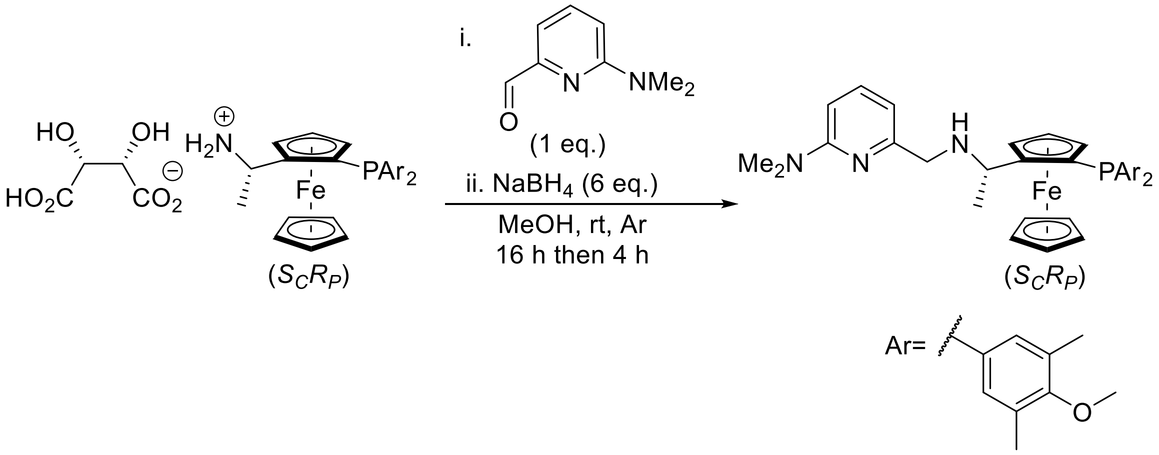

A microwave vial was flame dried under vacuum, cooled to rt and then cycled between vacuum and Ar atmosphere 3 times. Dry methanol was added and degassed by sparging with Ar for 30 minutes. A 2-neck round bottom flask equipped with magnetic stirrer and capped with rubber septa was flame dried under vacuum, cooled and cycled between vacuum and Ar atmosphere 3 times. A rubber septum was briefly removed to add (S_C,R_p)-1-(2-bis[4-methoxy-3, 5-dimethylphenyl]phosphino)ferrocenylethylamine L-tartrate salt (350 mg, 0.515 mmol, 1 eq.) the flask was again placed under vacuum and once the methanol had been degassed the flask was cycled between vacuum and Ar atmosphere three times. In a separate vial equipped with rubber septa was added 2-dimethylamino-6-formylpyridine (77.4 mg, 0.515 mmol, 1 eq.) and the vial placed under vacuum and cycled between vacuum and Ar atmosphere three times. The degassed methanol was then transferred to the vial containing aldehyde *via* syringe in portions (2 mL then 2 mL then 1 mL) to dissolve the aldehyde and each time transferring the aldehyde solution to the 2-neck flask *via* syringe. The reaction was then allowed to stir at rt for 16 h under an Ar atmosphere. Upon which time an aliquot was taken and analysed by ^{1}H NMR to ensure full conversion to imine had occurred. The rubber septum was again briefly removed and $NaBH_4$ (39 mg, 1.03 mmol, 2 eq.) was added and the reaction stirred for 1 h. A further 2 eq. of $NaBH_4$ was added and the reaction stirred for a further 1 h when another 2 eq. of $NaBH_4$ was added to the reaction. The reaction proceeded for another 2 h (4 h total) upon which time it was quenched with sat. K_2CO_3 solution (5 mL) and extracted with CH_2Cl_2 (5 mL) was added and the organic layer decanted off *via* syringe to a

Schlenk tube charged with $MgSO_4$ under Ar atmosphere. The CH_2Cl_2 addition/ decantation was repeated four more times. The combined dried organic layer was filtered under Ar atmosphere to a 2-neck round bottom flask and concentrated *in vacuuo.* The crude material was then Purified by flash column chromatography on silica gel (deactivated by 1% (v/v) NEt_3 in hexane) (Hexane/ EtOAc (1:1) as eluent) under Ar atmosphere and dried extensively under high vacuum to give:

(S_C, R_p)-N- [2-dimethylaminopyridine-6-methyl]-1-(2-bis[4-methoxy- 3, 5-dimethylphenyl]phosphino)ferrocenylethylamine- **L4**

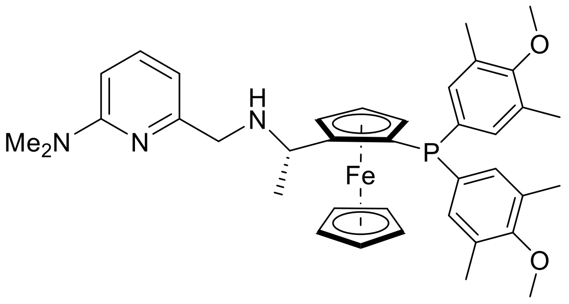

Yield (0.515 mmol Scale): 288.5 mg (84%) of yellow solid
^{1}H NMR (500 MHz, MeOD) δ 7.25 – 7.16 (3H, m, 2Ar-H, Py-H), 6.74 (2H, d, *J* = 7.1 Hz, 2Ar-H), 6.33 (1H, d, *J* = 8.4 Hz, Py-H), 5.85 (1H, d, *J* = 7.3 Hz, Py-H), 4.61 – 4.56 (1H, m, Fc-H), 4.45-4.38 (1H, m, Fc-H), 4.33-4.22 (1H, m, CH-Fc), 3.97 (5H, s, 5Fc-H), 3.96 – 3.91 (1H, m, Fc-H), 3.74 (3H, s, OMe), 3.58 (3H, s, OMe), 3.47 – 3.39 (2H, m, CH_2-Py), 2.89 (6H, s, NMe_2), 2.28 (6H, s, Me-Ar), 2.00 (6H, s, Me-Ar), 1.60 (3H, d, *J* = 6.7 Hz, CH_3-CHFc). $^{31}P\{^{1}H\}$ NMR (202 MHz, MeOD) δ -28.2. ^{13}C NMR (126 MHz, MeOD) δ 160.1 (Py-N) 159.4 (Ar-O), 158.5 (Ar-O), 157.3 (Py-C), 138.5 (Ar-H), 136.9 (d_{JC}, 22.4 Hz, Ar-P) , 136.3 (d_{JC}, 7.3 Hz, Ar-C), 133.9 (d_{JC}, 19.1 Hz, Ar-P), 133.5 (d_{JC}, 7.3 Hz, Ar-C), 132.0 (d_{JC}, 6.9 Hz, Ar-H), 131.7 (d_{JC}, 8.9 Hz, Ar-H), 109.5 (Py-H), 104.9 (Py-H), 97.7 (d_{JC}, 25.4 Hz, Fc-P), 77.3 (d_{JC}, 8.9 Hz, Fc-C), 72.6 (d_{JC}, 4.1 Hz, Fc-H), 70.7 (Fc-H), 70.6 (Fc-H), 70.4 (Fc-H), 60.2 (O-CH_3), 60.0 (O-CH_3), 52.0 (d_{JC}, 10.3 Hz, CH), 51.4 (CH_2), 38.10 (CH_3), 18.8 (CH_3), 16.3 (CH_3), 16.2 (CH_3). [α_D^{20}]: +201.2 (c. 6.6, MeOH).
HRMS (ESI+): Calculated for [$C_{38}H_{47}FeN_3O_2P$]: 664.2750 Found: 664.2737.

(R_C,S_P)-N- [2-dimethylaminopyridine-6-methyl]-1-(2-bis[4-methoxy- 3, 5-dimethylphenyl]phosphino)ferrocenylethylamine- **L4**

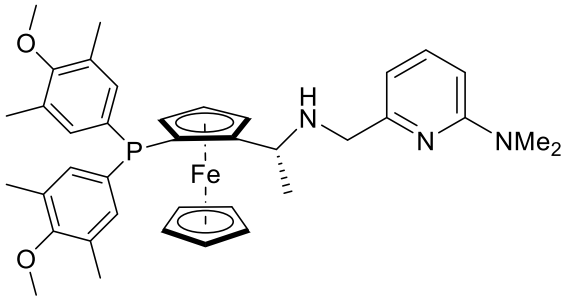

Prepared in same way as other enantiomer using the [(R_C,C_p)-1-(2-bis[4-methoxy-3, 5-dimethylphenyl]phosphino)ferrocenylethylamine L-tartrate salt in the imine reductive amination step.
Yield (0.29 mmol Scale): 128.1 mg (67%) of yellow solid
[α_D^{20}]: -199.1 (c. 6.6, MeOH)
HRMS (ESI+): Calculated for [$C_{38}H_{47}FeN_3O_2P$]: 664.2750 Found: 664.2739.

Preparation of [(*Rc, Sp*)-N-(2-(dimethylamino)pyridine-2-methyl)-1-(-2-bis(4-methoxy-3,5-dimethylphenyl)phosphino)ferrocenylethylamine]-κN1 -κN2 -κP-tricarbonyl manganese (I) bromide

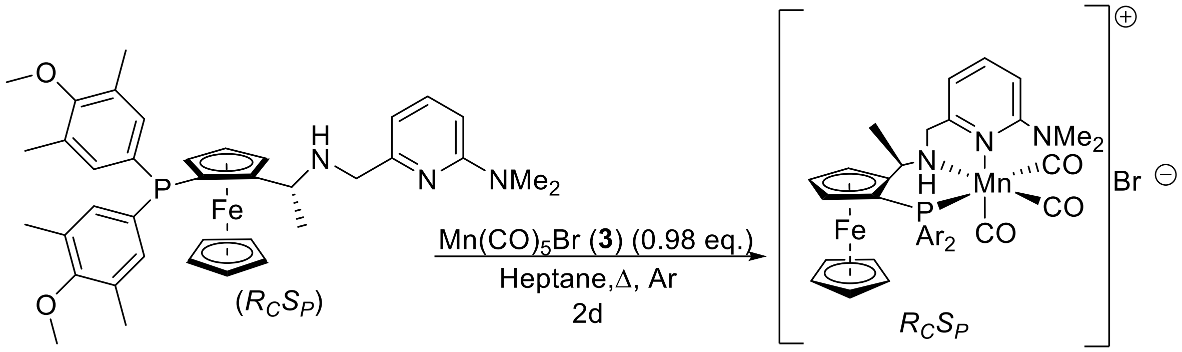

In a flame dried Schlenk tube dry heptane was degassed by sparging with Ar for 40 minutes. A microwave vial charged with a magnetic stirrer and fitted with a rubber septum was flame dried under vacuum. The vial was cooled to rt and cycled between vacuum and Ar atmosphere three times. The rubber septum was removed to allow R_CS_P-**L4** (117.8 mg, 0.178 mmol, 1 eq.) and $Mn(CO)_5Br$ (48.1 mg, 0.175 mmol, 0.98 eq.) to be added and the septum replaced with a crimp cap. The vial was again placed under vacuum while the solvent was degassed. Upon which time the vial was cycled three times between vacuum and Ar atmosphere and then degassed heptane (0.90 mL) was added *via* syringe. The vial was sealed and the reaction stirred at reflux for 48 h. After cooling the reaction to room temperature, the precipitate that was formed was collected by filtration, washed with hexane (50 mL) and dried extensively under high vacuum to give:

[(*Rc, Sp*)-N-(2-(dimethylamino)pyridine-2-methyl)-1-(-2-bis(4-methoxy-3,5-dimethylphenyl)phosphino)ferrocenylethylamine]-κN1 -κN2 -κP-tricarbonyl manganese (I) bromide

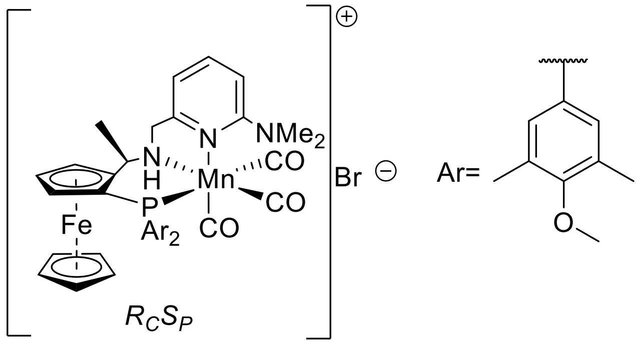

Yield (0.175 mmol scale): 105.7 mg (68%) of bright yellow solid
^{1}H NMR (500 MHz, MeOD) δ 7.65 (1H, s, Ar-H), 7.63 (1H, s, Ar-H), 7.49 (1H, t, *J* = 7.7 Hz, Py-H), 6.86 (1H, d, *J* = 7.7 Hz, Py-H), 6.79 (1H, d, *J* = 7.7 Hz, Py-H), 6.30 (1H, s, Ar-H), 6.28 (1H, s, Ar-H), 4.81-4.79 (1H, m, Fc-H), 4.69 – 4.67 (1H, m, Fc-H), 4.55 – 4.52 (1H, m, Fc-H), 4.20 – 4.11 (1H, m, C**H**-Fc), 3.84 (5H, s, 5Fc-H), 3.81 (3H, s, OMe), 3.76 – 3.70 (2H, m, CH_2-Py), 3.60 (3H, s, OMe), 2.67 (3H, bs, NMe), 2.51 (3H, bs, NMe) 2.38 (6H, s, Me-Ar), 1.97 (6H, s, Me-Ar), 1.90 – 1.85 (m, 1H), 1.67 (d, *J* = 6.7 Hz, 3H). $^{31}P\{^{1}H\}$ NMR (202 MHz, MeOD) δ 39.98. HRMS (ESI+): Calculated for [$C_{41}H_{46}FeMnN_3O_5P$]: 802.1900 Found: 802.1893.

Preparation of 4-tertbutyl benzophenone (**12$_{ke}$**)[5]

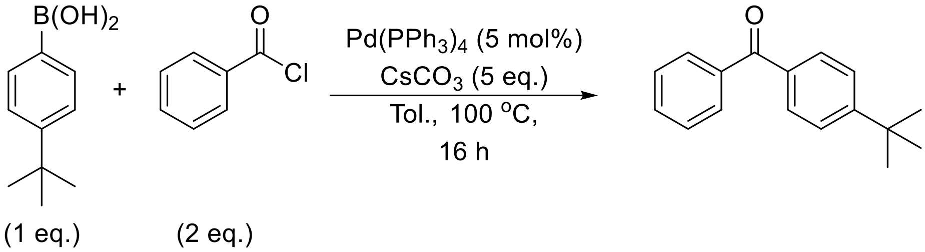

(Procedure adapted from ref. 6) A Schlenk tube was flame dried under vacuum, cooled and then cycled between vacuum and Ar atmosphere 3 times. 4-^{t}BuB(OH)$_2$ (534.2 mg, 3 mmol, 1 eq.), $CsCO_3$ (5.29 g, 15 mmol, 5 eq.) and $Pd(PPh_3)_4$ (173 mg, 0.15 mmol, 5 mol%) were then added to the flask which was placed under vacuum for a further 15 minutes. Upon which time, the flask was cycled through Ar atmosphere and vacuum a further 3 times and dry toluene (30 mL) followed by BzCl (0.20 mL, 6 mmol, 2 eq.) were then added *via* syringe. The reaction was then allowed to stir at 100 °C overnight under an Ar atmosphere. After this time, the reaction was cooled to rt, passed through a pad of celite and diluted with 50 mL EtOAc. The organic phase was washed with water, conc. $NaHCO_3$ solution and brine. The organic phase was then dried over Na_2SO_4, filtered and concentrated *in vacuo*. The crude material was then purified by flash column chromatography using Hexane/ EtOAc (9:1) as eluent and dried *in vacuo* to give:

4-tertbutyl benzophenone (**12$_{ke}$**)

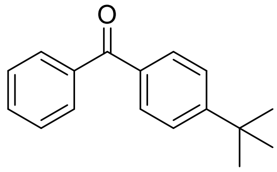

Yield (3 mmol scale): 353.4 mg (49%) of viscous yellow oil
^{1}H NMR (500 MHz, $CDCl_3$) δ 7.83 – 7.79 (2H, m, Ar-H), 7.78 – 7.74 (2H, m, Ar-H), 7.61 – 7.55 (1H, m, Ar-H), 7.52 – 7.45 (4H, m, Ar-H), 1.37 (9H, s, $(CH_3)_3$). ^{13}C NMR (126 MHz, $CDCl_3$) δ 196.7 (C=O), 156.3 (Ar-C), 138.0 (Ar-C), 134.9 (Ar-C), 132.3 (Ar-H), 130.3 (Ar-H), 130.1 (Ar-H), 128.3 (Ar-H), 125.4 (Ar-H), 35.3 (C), 31.3 (CH_3). Compound has been prepared previously using an alternative route and data reported here is consistent with previously published data.[7]

Section 1.3: General Procedures

General Procedure for the Manganese-Catalysed Pressure Hydrogenation of ketones using **L4** and $Mn(CO)_5Br$ (when ketone was solid at rt)- **Method A**

A microwave vial charged with magnetic stirrer bar and fitted with rubber septum was flame dried under vacuum. The vial was cooled to rt and then cycled between vacuum and Ar atmosphere three times. Working quickly, the rubber septum was removed then: substrate (1 eq.), ligand (0.01 eq.), $Mn(CO)_5Br$ (0.01 eq.) and K_2CO_3 (0.1 eq.) were added and the vial fitted with a crimp camp and septum. The vial was placed back under vacuum for a further 10 minutes. After cycling vacuum and argon atmosphere three more times, EtOH (0.67 M) was added *via* syringe, the septum pierced by two 21G needles and the vial placed directly into an autoclave which had been cycled between vacuum and Ar atmosphere 3 times. The autoclave was sealed and pressurised to 15 Bar of H_2 which was subsequently vented, this was repeated two more times to degas the solvent. Finally the autoclave was pressurised to 50 Bar H_2 and the autoclave was then placed in an oil bath preheated to 50 °C and stirred at 700 rpm for 18 h. Upon which time, the autoclave was cooled to rt, carefully vented in a fume cupboard and 1,4-dimethoxybenzene (0.25 eq. [relative to ketone]) was added to

the sample as internal standard. The reaction was diluted in $CDCl_3$ (1 mL) and a small aliquot taken for ^{1}H NMR analysis to determine conversion. The NMR sample was retrieved and all solvent was removed *in vacuo* prior to chromatographic purification.

General Procedure for the Manganese-Catalysed Pressure Hydrogenation of ketones using **L4** and $Mn(CO)_5Br$ (when ketone was liquid at rt)- **Method B**

A microwave vial charged with magnetic stirrer bar and fitted with rubber septum was flame dried under vacuum. The vial was cooled and then cycled between vacuum and Ar atmosphere three times. Working quickly, the rubber septum was removed then: ligand (0.01 eq.), $Mn(CO)_5Br$ (0.01 eq.) and K_2CO_3 (0.1 eq.) were added and the vial fitted with a crimp camp and septum. The vial was placed back under vacuum for a further 10 minutes. After cycling vacuum and argon atmosphere three more times, EtOH (0.67 M) was added *via* syringe followed by substrate (1 eq.) being added *via* microliter syringe, the septum was pierced by two 21G needles and the vial placed directly into an autoclave which had been cycled between vacuum and Ar atmosphere 3 times. The autoclave was sealed and pressurised to 15 Bar of H_2 which was subsequently vented, this was repeated two more times to degas the solvent. Finally the autoclave was pressurised to 50 Bar H_2 and the autoclave was then placed in an oil bath preheated to 50 °C and stirred at 700 rpm for 18 h. Upon which time, the autoclave was cooled to rt, carefully vented in a fume cupboard and 1,4-dimethoxybenzene (0.25 eq. [relative to ketone]) was added to the sample as internal standard. The reaction was diluted in $CDCl_3$ (1 mL) and a small aliquot taken for ^{1}H NMR analysis to determine conversion. The NMR sample was retrieved and all solvent was removed *in vacuo* prior to chromatographic purification.

Section 1.4: Characterisation Data for Alcohol Products

4-Chromanol (**5**$_{al}$)

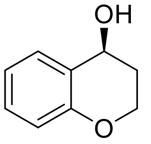

Produced by method A

Purified by flash column chromatography on silica gel: CH_2Cl_2 then CH_2Cl_2/MeOH (97:3) as eluent

Yield (0.2 mmol scale): 29.3 mg (98%) of off-white solid

^{1}H NMR (500 MHz, $CDCl_3$) δ 7.34-7.30 (1H, m, Ar-H), 7.24-7.18 (1H, m, Ar-H), 6.96-6.90 (1H, m, Ar-H), 6.87-6.81 (1H, m, Ar-H), 4.80 (1H, t, *J* = 4.0 Hz, C**H**-OH), 4.31-4.24 (2H, m, C**HH'**-OAr), 2.19 – 2.09 (1H, m, C**H**H'-CHOH), 2.08-2.01 (1H, m, CH**H'**-CHOH), 1.74 (s, O-H). ^{13}C NMR (126 MHz, $CDCl_3$) δ 154.7 (Ar-O), 129.9 (Ar-H), 129.8 (Ar-C), 124.4 (Ar-H), 120.7 (Ar-H), 117.2 (Ar-H), 63.4 (CH), 62.0 (CH_2), 30.9 (CH_2). [α_D^{20}]: -34.6 (c. 0.5, $CHCl_3$); Lit.[7]: -65.1 (c. 1.0, $CHCl_3$, ee. 94%, *S*). HRMS (ESI-): Calculated for [$C_9H_9O_2$]: 149.0608 Found: 149.0597. Chiral analysis was performed using a Chiralcel OD-H column using Hexane/ ^{i}PrOH (95:5) as mobile phase with 0.5 mL/min flowrate: t_R (*S*)= 19.81 min (99%), t_R (*R*)= 23.04 min (1%). 98% ee, *S*. Compound has been prepared previously using an alternative route and data reported here is consistent with previously published data.[8]

1-Phenylethanol (**6**$_{al}$)

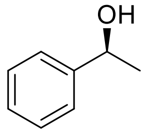

Produced by method B

Purified by flash column chromatography on silica gel: CH_2Cl_2/MeOH (98:2) as eluent

Yield (0.43 mmol scale): 36.5 mg (70%) of pale yellow oil

^{1}H NMR (400 MHz, $CDCl_3$) δ 7.41 – 7.33 (4H, m, 4Ar-H), 7.30 – 7.27 (1H, m, Ar-H), 4.91 (q, *J* = 6.4 Hz, 1H), 1.51 (d, *J* = 6.4 Hz, 3H). [α_D^{20}]: -40.4 (c. 1.2, $CHCl_3$); Lit.[9]: 42.9 (c. 1.04, $CHCl_3$, ee. 98%, *R*). LRMS (EI+): Calculated for [C_8H_9O]: 123 Found: 123. Chiral analysis was performed using a Chiralcel OD-H column using Hexane/ ^{i}PrOH (95:5) as mobile phase with 0.5 mL/min flowrate: t_R (*R*)= 17.10 min (8%), t_R (*S*)= 21.66 min (92%). 84% ee, *S*. Compound has been prepared previously using an alternative route and data reported here is consistent with previously published data.[8]

α-Tetralol (**7**$_{al}$)

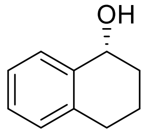

Produced by method B

Purified by flash column chromatography on silica gel: Hexane/ EtOAc (9:1 then 4:1) as eluent

Yield (0.2 mmol scale): 26.7 mg (90%) of off-white solid

^{1}H NMR (500 MHz, $CDCl_3$) δ 7.47 – 7.42 (1H, m, Ar-H), 7.24 – 7.18 (2H, m, 2Ar-H), 7.14 – 7.09 (1H, m, Ar-H), 4.82 – 4.77 (1H, m, C**H**-OH), 2.89-2.80 (1H, m, C**H**H'-Ar), 2.77 – 2.69 (1H, m, CH**H'**-Ar), 2.04 – 1.89 (3H, m, C**HH'**-CHOH, C**H**H'-CH_2Ar), 1.83 – 1.74 (1H, m, CH**H'**-CH_2Ar), 1.67 (1H, s, O-H). ^{13}C NMR (126 MHz, $CDCl_3$) δ 138.9 (Ar-C), 137.3 (Ar-C), 129.2 (Ar-H), 128.8 (Ar-H), 127.8 (Ar-H), 126.3 (Ar-H), 68.3 (CH), 32.4 (CH_2), 29.4 (CH_2), 18.9 (CH_2). [α_D^{20}]: -27.1 (c. 1.1, $CHCl_3$); Lit.[11]: -26.2 (c. 1.0, $CHCl_3$, ee. 98%, *R*). HRMS (ESI-): Calculated for [$C_{10}H_{11}O$]: 147.0815 Found: 147.0804. Chiral analysis was performed using a Chiralcel OD-H column using Hexane/ ^{i}PrOH (99:1) as mobile phase with 1 mL/min flowrate: t_R (*S*)= 21.79 min (1%), t_R (*R*)= 25.69 min (99%). 98% ee, *R*. Compound has been prepared previously using an alternative route and data reported here is consistent with previously published data.[11]

Indan-1-ol (**8**$_{al}$)

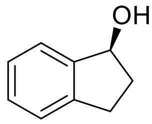

Produced by method A

Purified by flash column chromatography on silica gel: CH_2Cl_2/MeOH (97:3) as eluent

Yield (0.4 mmol scale): 48.6 mg (91%) of off-white solid

^{1}H NMR (400 MHz, $CDCl_3$) δ 7.50 – 7.41 (1H, m, ArH-(C)CHOH), 7.27 – 7.21 (3H, m, 3Ar-H), 5.26 (1H, t, *J* = 6.8 Hz, C**H**-OH), 3.15-3.01 (1H, m, C**H**H'-Ar), 2.87-2.77 (1H, m, CH**H'**-Ar), 2.56-2.39 (1H, m, C**H**H'-CHOH), 2.05-1.84 (1H, m, CH**H'**-CHOH) 1.97 (s, O-H, 1H). ^{13}C NMR (101 MHz, $CDCl_3$) δ 145.0 (Ar-H), 143.4 (Ar-H), 128.4 (Ar-H), 126.7 (Ar-H), 124.9 (Ar-H), 124.2 (Ar-H), 76.5 (CH), 36.0 (CH_2), 29.8 (CH_2). [α_D^{20}]: +20.0 (c. 1.3, $CHCl_3$); Lit.[12]: +15.7 (c. 1.0, $CHCl_3$, ee. 88%, *S*). HRMS (ESI-): Calculated for [C_9H_9O]: 133.0658 Found: 133.0648. Chiral analysis was performed using a Chiralcel OD-H column using Hexane/ ^{i}PrOH (98:2) as mobile phase with 1 mL/min flowrate: t_R (*S*)= 16.69 min (97%), t_R (*R*)= 19.92 min (3%). 94% ee, *S*. Compound has been prepared previously using an alternative route and data reported here is consistent with previously published data.[13]

Cyclohex-2-en-1-yl 4-nitrobenzoate (**9**$_{al}$)

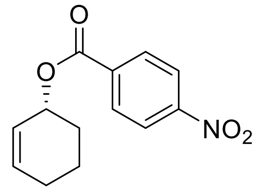

Produced by method B

Upon completion of hydrogenation reaction, after cooling and venting of autoclave, directly to the reaction solvent was added 4-NO_2BzCl (7 mmol), the reaction was diluted in 5 mL CH_2Cl_2 and then NEt_3 (7 mmol) was added and the reaction allowed to stir at rt overnight. The reaction was concentrated *in vacuo* and loaded directly onto the column.

Purified by flash column chromatography on silica gel: Hexane/ EtOAc (19:1 then 14:1) as eluent

Yield for direct hydrogenation reaction (0.2 mmol scale): 18.0 mg (36%) of pale yellow solid

^{1}H NMR (400 MHz, $CDCl_3$) δ 8.33 – 8.28 (2H, m, 2Ar-H), 8.26 – 8.21 (2H, m, 2Ar-H), 6.14 – 6.03 (1H, m, C**H**=CHCHOH), 5.91 – 5.82 (1H, m, =C**H**-CHOH), 5.60 – 5.51 (1H, m, C**H**-OH), 2.26 – 1.69 (6H, m, 3CH_2). ^{13}C NMR (126 MHz, $CDCl_3$) δ 164.5 (C=O), 150.5 (Ar-C), 136.3 (Ar-C), 133.8 (CH), 130.8 (Ar-H), 125.1 (Ar-H), 123.6 (CH), 69.9 (CH), 28.4 (CH_2), 25.0 (CH_2), 18.9 (CH_2). [α_D^{20}]: +122.5 (c. 0.8, $CHCl_3$); Lit.[14]: -146.2 (c. 1.0, $CHCl_3$, ee. 93%, *S*). HRMS (ESI): Calculated for [$C_{13}H_{14}NO_4$]: 246.0771 Found: 246.1128. Chiral analysis was performed using a Chiralcel OD-H column using Hexane/ ^{i}PrOH (99:1) as mobile phase with 0.3 mL/min flowrate: t_R (*R*)= 36.14 min (95%), t_R (*S*)= 40.02 min (5%). 90% ee, *R*. Compound has been prepared previously using an alternative route and data reported here is consistent with previously published data.[14]

3-Methyl-2-cyclohexen-1-ol (**10**$_{al}$)

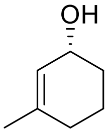

Produced by method B

Purified by flash column chromatography on silica gel: Hexane/ Et_2O (2:1) then (1:1) as eluent

Yield (0.2 mmol scale): 18.9 mg (84%) of colourless oil

^{1}H NMR (500 MHz, $CDCl_3$) δ 5.56-5.43 (1H, m, C**H**=CMe), 4.26-4.11 (1H, m, C**H**-OH), 2.02 – 1.83 (2H, m, C**HH'**-CHOH), 1.82 – 1.71 (2H, m, C**HH'**-CMe), 1.63 – 1.52 (2H, m, C**HH'**-CH_2CMe), 1.43 (s, O-H). ^{13}C NMR (126 MHz, $CDCl_3$) δ 138.9 (C), 124.4 (CH), 66.0 (CH), 31.8 (CH_2), 30.2 (CH_2), 23.8 (CH_2), 19.1 (CH_3). [α_D^{20}]: +39.4 (c. 0.7, $CHCl_3$); Lit.[15]: +62.4 (c. 1.0, $CHCl_3$, ee. 96%, *R*). LRMS (EI+): Calculated for [$C_7H_{12}O$]: 112 Found: 112. Chiral analysis was performed using a Chiralcel AD-H column using Hexane/ ^{i}PrOH (98:2) as mobile phase with 0.7 mL/min flowrate: t_R (*R*)= 17.44 min (94%), t_R (*S*)= 18.60 min (6%). 88% ee, *R*. Compound has been prepared previously using an alternative route and data reported here is consistent with previously published data.[15]

4,4-Dimethylcyclohexen-1-ol (**11**$_{al}$)

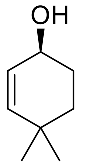

Produced by method B
Purified by flash column chromatography on silica gel: CH_2CL_2 then CH_2Cl_2/MeOH (97:3) as eluent
Yield (0.2 mmol scale): 22.2 mg (77%) of colourless oil

^{1}H NMR (500 MHz, $CDCl_3$) δ 5.59 (1H, dd, *J* = 10.0, 2.9 Hz, =C**H**-CHOH), 5.52 (1H, d, *J* = 10.0 Hz, =C**H**-CMe_2), 4.18 – 4.10 (1H, m, C**H**-OH), 1.97 – 1.86 (1H, m, C**H**H'-CHOH), 1.66 – 1.58 (1H, m, CH**H'**-CHOH), 1.58 – 1.53 (1H, m, C**H**H'-CMe_2), 1.47-1.36 (1H, m, CH**H'**-CMe_2), 1.01 (1H, s, CH_3), 0.96 (3H, s, CH_3). ^{13}C NMR (126 MHz, $CDCl_3$) δ 140.8 (CH), 127.4 (CH), 66.1 (CH), 33.7 (CH_2), 32.0 (C), 29.4 (CH_2), 29.3 (CH_3), 29.2 (CH_3). [α_D^{20}]: -91.0 (c. 0.7, $CHCl_3$); Lit.[16]: -104 (c. 0.7, $CHCl_3$, ee. 98%, S). After converting product to benzoate ester; chiral analysis was performed using Chiralcel AD-H column with using Hexane/ ^{i}PrOH (100:0) as mobile phase with 0.75 mL/min flowrate: t_R (*R*)= 12.49 min (7%), t_R (*S*)= 15.22 min (93%). 86% ee, *S*. Compound has been prepared previously using an alternative route and data reported here is consistent with previously published data.[16]

(4-^{t}BuPhenyl)(phenyl)methanol (**12**$_{al}$)

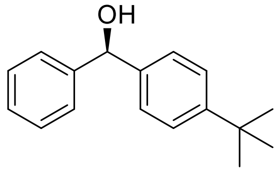

Produced by method A
Purified by flash column chromatography on silica gel: Hexane/ EtOAc (9:1, 7:1 then 4:1) as eluent
Yield (0.2 mmol scale): 46.4 mg (81%) of off-white solid

^{1}H NMR (500 MHz, $CDCl_3$) δ 7.47 – 7.27 (9H, m, 9Ar-H), 5.83 (1H, d, *J* = 3.1 Hz, C**H**-OH), 2.17 (1H, s, O-H), 1.30 (9H, s, $(CH_3)_3$). ^{13}C NMR (126 MHz, $CDCl_3$) δ 150.7 (Ar-C), 144.0 (Ar-C), 141.0 (Ar-C), 128.6 (Ar-H), 127.6 (Ar-H), 126.6 (Ar-H), 126.4 (Ar-H), 125.6 (Ar-H), 76.2 (CH), 34.7 (C), 31.47 (CH_3). [α_D^{20}]: +6.0 (c. 1.0, $CHCl_3$); Lit.[18]: +52.9 (c. 1.1, $CHCl_3$, ee. 90%, *R*). HRMS (ESI-): Calculated for [$C_{17}H_{19}O$]: 239.1441 Found: 239.1429. Chiral analysis was performed using a Chiralcel OD-H column using Hexane/ ^{i}PrOH (90:10) as mobile phase with 0.5 mL/min flowrate: t_R (*S*)= 14.82 min (32%), t_R (*R*)= 16.35 min (68%). 36% ee, *R*. Compound has been prepared previously using an alternative route and data reported here is consistent with previously published data.[17]

1-(anthracen-9-yl)-2,2,2-trifluoroethan-1-ol (**13**$_{al}$)

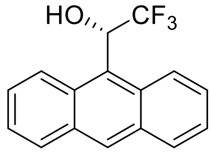

Produced by method A
Purified by flash column chromatography on silica gel: CH_2Cl_2 as eluent
Yield (0.4 mmol scale): 93.8 mg (85%) of beige solid

^{1}H NMR (500 MHz, $CDCl_3$) δ 8.97 (1H, bs, Ar-H), 8.56 (1H, s, Ar-H), 8.15 (1H, bs, Ar-H), 8.09-7.95 (2H, m, 2Ar-H), 7.57 (2H, bs, 2Ar-H), 7.56-7.39 (2H, m, 2Ar-H), 6.76-6.59 (1H, m, C**H**-OH), 3.05 (1H, s, OH). ^{19}F NMR (471 MHz, $CDCl_3$) δ -74.0. HRMS (ESI-): Calculated for [$C_{16}H_{10}F_3O$]: 275.0689 Found: 275.0690. Chiral analysis was performed using a Chiralcel OD-H column using Hexane/ ^{i}PrOH (90:10) as mobile phase with 0.5 mL/min flowrate: t_R (*R*)= 22.02 min (36%), t_R (*S*)= 44.68 min (64%). 28% ee,

S. Compound has been prepared previously using an alternative route and data reported here is consistent with previously published data.[18]

1-(4-Bromophenyl)ethanol (**14**$_{al}$)

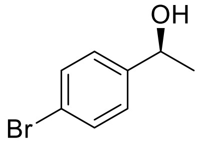

Produced by method A
Purified by flash column chromatography on silica gel using CH_2Cl_2/ MeOH (98:2) as eluent
Yield (0.5 mmol scale): 97.6 mg (98%) of pale yellow oil
^{1}H NMR (500 MHz, $CDCl_3$) δ 7.49 – 7.43 (2H, m, 2Ar-H), 7.25 – 7.22 (2H, m, 2Ar-H), 4.85 (1H, q, *J* = 6.4 Hz, C**H**-OH), 2.00 (s, O-H), 1.46 (3H, d, *J* = 6.4 Hz, CH_3). ^{13}C NMR (126 MHz, $CDCl_3$) δ 144.9 (Ar-Br), 131.7 (Ar-H), 127.3 (Ar-H), 121.3 (Ar-C), 69.9 (CH), 25.4 (CH_3). [α_D^{20}]: -30.3 (c. 1.0, $CHCl_3$); Lit.[19]: -28.0 (c. 1.0, $CHCl_3$, ee. 74%, *S*). HRMS (ESI-): Calculated for [C_8H_8Br]: 182.9814 Found: 182.9805. Chiral analysis was performed using a Chiralcel OD-H column using Hexane/ ^{i}PrOH as mobile phase with a 0.5 mL/min flowrate: t_R (*S*)= 17.87 min (91%), t_R (*R*)= 18.92 min (9%). 82% ee, *S*. Compound has been prepared previously using an alternative route and data reported here is consistent with previously published data.[20]

1-Phenylpropan-1-ol (**15**$_{al}$)

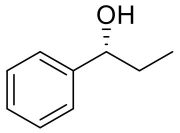

Produced by method B
Purified by flash column chromatography on silica gel: Hexane/ Et_2O (1:1) as eluent
Yield (0.2 mmol scale): 24.4 mg (90%) of colourless oil
^{1}H NMR (500 MHz, $CDCl_3$) δ 7.37-7.33 (4H, m, 4Ar-H), 7.30 – 7.27 (1H, m, Ar-H), 4.61 (1H, t, *J* = 6.6 Hz, C**H**-OH), 1.90 – 1.70 (2H, m, C**HH'**), 0.92 (3H, t, *J* = 7.4 Hz, CH_3). ^{13}C NMR (126 MHz, $CDCl_3$) δ 144.7 (Ar-C), 128.6 (Ar-H), 127.7 (Ar-H), 126.1 (Ar-H), 76.2 (CH), 32.0 (CH_2), 10.3 (CH_3). [α_D^{20}]: +18.8 (c. 0.6, $CHCl_3$); Lit.[21]: +37.0 (c. 0.95, $CHCl_3$, ee. 84%, *R*). LRMS (EI+): Calculated for [$C_9H_{12}O$]: 136 Found: 136. Chiral analysis was performed using a Chiralcel OD-H column using Hexane/ ^{i}PrOH (95:5) as mobile phase with 0.5 mL/min flowrate: t_R (R)= 15.83 min (86%), t_R (S)= 19.03 min (14%). 72% ee, *R*. Compound has been prepared previously using an alternative route and data reported here is consistent with previously published data.[22]

1-(2-Naphthyl)ethanol (**16**$_{al}$)

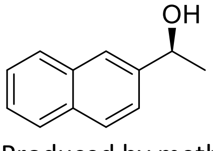

Produced by method A
Purified by flash column chromatography on silica gel: CH_2Cl_2 then CH_2Cl_2/MeOH (98:2) as eluent
Yield (0.5 mmol scale): 85.4 mg (99%) of white solid
^{1}H NMR (500 MHz, $CDCl_3$) δ 7.89-7.78 (4H, m, 4Ar-H), 7.55 – 7.44 (3H, m, 3Ar-H), 5.07 (1H, q, *J* = 6.4 Hz, C**H**), 1.92 (s, O-H), 1.59 (3H, d, *J* = 6.4 Hz, CH_3). ^{13}C NMR (126 MHz, $CDCl_3$) δ 143.3 (Ar-C), 133.4 (Ar-C), 133.0 (Ar-C), 128.5 (Ar-H), 128.1 (Ar-H), 127.8 (Ar-H), 126.3 (Ar-H), 125.9 (Ar-H), 123.9 (Ar-H), 70.7 (CH), 25.3 (CH_3). [α_D^{20}]: -39.8 (c. 1.0, $CHCl_3$); Lit.[24]: +43.3 (c. 1.2, $CHCl_3$, ee. 96%, *R*). HRMS (ESI-): Calculated for [$C_{12}H_{11}$]: 155.0866 Found: 155.0856. Chiral analysis was performed using a Chiralcel

OD-H column using Hexane/ ^{i}PrOH (95:5) as mobile phase with 0.5 mL/min flowrate: t_R (*S*)= 32.08 min (94%), t_R (*R*)= 34.61 min (6%). 88% ee, *S*. Compound has been prepared previously using an alternative route and data reported here is consistent with previously published data.[22]

5-Fluoroindan-1-ol (**17**$_{al}$)

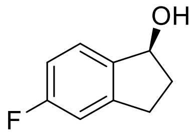

Produced by method A

Purified by flash column chromatography on silica gel: Hexane/ Et_2O (1:1.5) as eluent

Yield (0.4 mmol scale): 47.5 mg (78%) of off-white solid

^{1}H NMR (400 MHz, $CDCl_3$) δ 7.39-7.32 (1H, m, Ar-H), 7.12 – 6.87 (2H, m, 2Ar-H), 5.24 (1H, dd, *J* = 6.8, 5.1 Hz, CH-OH), 3.14 – 3.03 (1H, m, C**H**H'-Ar), 2.87-2.74 (1H, m, CH**H'**-Ar), 2.61-2.47 (1H, m, C**H**H'-CHOH), 2.07-1.95 (1H, m, CH**H'**-CHOH), 1.67 (s, OH, 1H). ^{13}C NMR (126 MHz, $CDCl_3$) δ 163.3 (d, *J* = 245.2 Hz, Ar-F), 145.8 (d, *J* = 8.6 Hz, Ar-C), 140.6 (d, *J* = 2.4 Hz, Ar-C), 125.5 (d, *J* = 9.2 Hz, Ar-H), 113.9 (d, *J* = 22.8 Hz, Ar-H), 111.8 (d, *J* = 22.0 Hz, Ar-H), 75.7 (CH), 36.37 (CH_2), 29.9 (CH_2). [α_D^{20}]: +21.5 (c. 0.8, $CHCl_3$); Lit.[25]: -28.8 (c. 1.0, $CHCl_3$, ee. 97%, *R*). HRMS (ESI-): Calculated for [C_9H_8FO]: 151.0564 Found: 151.0556. Chiral analysis was performed using a Chiralcel OD-H column using Hexane/ ^{i}PrOH (95:5) as mobile phase with 0.5 mL/min flowrate: t_R (*S*)= 16.44 min (96%), t_R (*R*)= 18.51 min (4%). 92% ee, *S*. Compound has been prepared previously using an alternative route and data reported here is consistent with previously published data.[23]

Benzosuber-1-ol (**18**$_{al}$)

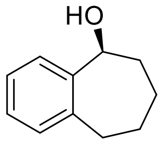

Produced by method B

Purified by flash column chromatography on silica gel: CH_2Cl_2/MeOH (97:3) as eluent

Yield (0.4 mmol scale): 63.7 mg (98%) of white solid

^{1}H NMR (400 MHz, $CDCl_3$) δ 7.44 (1H, d, *J* = 7.4 Hz, Ar-H), 7.21 (1H, td, *J* = 7.4, 1.6 Hz, Ar-H), 7.16 (1H, td, *J* = 7.4, 1.6 Hz, Ar-H), 7.10 (1H, dd, *J* = 7.4, 1.6 Hz, Ar-H), 4.98-4.90 (1H, m, C**H**-OH), 2.92 (1H, m, C**H**H'-Ar), 2.76-2.67 (1H, m, CH**H'**-Ar), 2.13 – 2.01 (1H, m, C**H**H'-CHOH), 1.96 (1H, m, CH**H'**-CHOH), 1.88 – 1.73 (3H, m, C**H**H'-CH_2Ar, C**HH'**-CH_2CHOH), 1.69 (1H, s, O-H) 1.58 – 1.41 (1H, m, CH**H'**-CH_2Ar). ^{13}C NMR (126 MHz, $CDCl_3$) δ 144.3 (Ar-C), 140.8 (Ar-C), 129.5 (Ar-H), 126.9 (Ar-H), 126.1 (Ar-H), 124.6 (Ar-H), 73.9 (CH), 36.6 (CH_2), 35.7 (CH_2) , 27.83 (CH_2), 27.58 (CH_2). [α_D^{20}]: -40.0 (c. 1.6, $CHCl_3$); Lit.[24]: -27.0 (c. 1.0, $CHCl_3$, ee. >99%, *S*). HRMS (ESI-): Calculated for [$C_{11}H_{13}O$]: 161.0971 Found: 161.0962. Chiral analysis was performed using a Chiralcel OD-H column using Hexane/ ^{i}PrOH (95:5) as mobile phase with 0.5 mL/min flowrate: t_R (*R*)= 22.99 min (3%), t_R (*S*)= 30.73 min (97%). 94% ee, *S*. Compound has been prepared previously using an alternative route and data reported here is consistent with previously published data.[13]

4,4-Diphenylcyclohexen-1-ol (**19**$_{al}$)

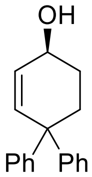

Produced by method A

Purified by flash column chromatography on silica gel: CH_2Cl_2 then CH_2Cl_2/MeOH (95:5) as eluent

Yield (0.1 mmol scale): 24.6 mg (98%) of white solid

^{1}H NMR (500 MHz, $CDCl_3$) δ 7.32 – 7.26 (3H, m, 3Ar-H), 7.26 – 7.15 (7H, m, 7Ar-H), 6.23 (1H, d, *J* = 10.1 Hz, =C**H**-CPh$_2$), 6.00 (1H, dd, *J* = 10.1, 3.2 Hz, =C**H**-OH), 4.28 (1H, m, C**H**-OH), 2.51 – 2.44 (1H, m, C**H**H'-CHOH), 2.32-2.25 (1H, m, CH**H'**-CHOH), 1.98-1.90 (1H, m, C**H**H'-CPh$_2$), 1.73-1.64 (1H, m, CH**H'**-CPh$_2$). ^{13}C NMR (126 MHz, $CDCl_3$) δ 148.1 (Ar-C), 148.0 (Ar-C), 137.5 (=CH), 130.1 (Ar-H), 128.3 (Ar-H), 128.0 (Ar-H), 127.8 (Ar-H), 126.2 (=CH), 65.8 (CH), 48.7 (C), 32.88 (CH$_2$), 29.16 (CH$_2$). [α_D^{20}]: -37.5 (c. 0.7, $CHCl_3$). HRMS (ESI-): Calculated for [$C_{18}H_{17}O$]: 249.1284 Found: 249.1270. Chiral analysis was performed using a Chiralcel OD-H column using Hexane/ ^{i}PrOH (95:5) as mobile phase with 0.5 mL/min flowrate: t_R (S)= 26.85 min (88%), t_R (R)= min (12%). 76% ee, *S*. The racemate of the above compound has been prepared previously using an alternative route and data reported here is consistent with previously published data.[25]

Licarbazepine (**20**$_{al}$)

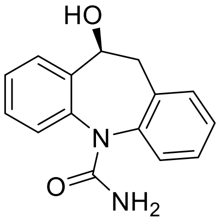

Produced by method A

Purified by flash column chromatography using CH_2Cl_2/MeOH (98:2 then 95:5)

Yield: (0.1 mmol Scale): 24.5 mg (96%) of white solid.

^{1}H NMR (400 MHz, DMSO-d_6) δ 7.60 – 7.44 (1H, m, Ar-H), 7.39 – 7.07 (7H, m, 7Ar-H), 5.77 (2H, s, NH$_2$), 5.66 (1H, s, C**H**-OH), 5.10 (1H, bs, C**H**H'-CHOH), 2.86 (1H, bs, CH**H'**-CHOH). [α_D^{20}]: +144.0 (c. 1.0, MeOH). HRMS (ESI+): Calculated for [$C_{15}H_{15}N_2O_2$]: 255.1129 Found: 255.1122. Chiral analysis was performed using a Chiralcel AD-H column using Hexane/ ^{i}PrOH (80:20) as mobile phase with 1 mL/min flowrate: t_R (*R*)= 9.77 min (1%), t_R (*S*)= 11.15 min (98%). 98% ee, *S*. Compound has been prepared previously using an alternative route and data reported here is consistent with previously published data.[13]

4-androstenediol

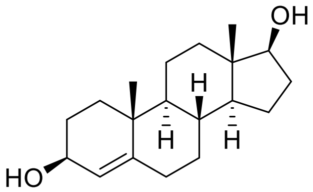

Produced by method A

Purified by flash column chromatography on silica gel: CH_2Cl_2/MeOH (95:5) as eluent

Yield (0.1 mmol scale): 27.7 mg (96%) of white solid

^{1}H NMR (400 MHz, $CDCl_3$) δ 5.35 – 5.22 (1H, m, =C**H**-CHOH), 4.18-4.09 (1H, m, OHC**H**-CH=), 3.61 (1H, t, *J* = 8.6 Hz, OHC**H**-CH$_2$), 2.24-2.13 (1H, m,), 2.12 – 1.89 (3H, m,), 1.84-1.77 (1H, m,), 1.76-1.66 (2H,

m), 1.64 – 1.19 (10H, m,), 1.05 (3H, s, CH_3), 1.01 – 0.80 (2H, m,), 0.75 (3H, s, CH_3). ^{13}C NMR (101 MHz, $CDCl_3$) δ 147.6 (=C), 123.7 (=CH), 82.0 (CH), 68.1 (CH), 54.7, 50.9, 43.0, 37.5, 36.7, 36.1, 35.6, 32.8, 32.2, 30.6, 29.6, 23.55, 20.8, 19.1, 11.2. HRMS (ESI-): Calculated for [$C_{19}H_{29}O_2$]: 289.2173 Found: 289.2157. Syn/Anti: 33:1. Compound has been prepared previously using an alternative route and data reported here is consistent with previously published data.[26]

Section 1.5: References:

[1] P. K. Agarwal, M. Saifuddin and B. Kundu, *Tetrahedron*, 2010, **66**, 862-870.
[2] D. Cai, R. D. Larsen, P. J. Reider, *Tetrahedron Lett*., 2002, **43**, 4285-4287.
[3] T. Sammakia and T. B. Hurley, J. *Org. Chem*., 1999, **64**, 4652–4664.
[4] M. B. Widegren, G. J. Harkness, A. M. Z. Slawin, D. B. Cordes and M. L. Clarke, *Angew. Chem. Int. Ed.*, 2017, **56**, 5825–5828.
[5] M. Haddach and J. R. McCarthy, *Tetrahedron Lett*., 1999, **40**, 3109-3112.
[6] M. Zabransky, I. Cisarova and P. Stepnicka, Organometallics, 2018, 37, 1615-1626.
[7] A. S. Rowan, T. S. Moody, R. M. Howard, T. J. Underwood, I. R. Miskelly, Y. He and B. Wang, *Tetrahedron Asymmetry*, 2013, **24**, 1369-1381.
[8] A. Kisic, M. Stephan and B. Mohar, *Adv. Synth. Catal*., 2015, **357**, 2540-2546.
[9] D. R. Li, A. He and J. R. Falck, *Org. Lett.*, 2010, **12**, 1756-1759.
[10] F. Ling, S. Nian, J. Chen, W. Luo, Z. Wang, Y. Lv, and W. Zhong, *J. Org. Chem*., 2018, **83**, 10749-10761.
[11] C. Tian, L. Gong and E. Meggers, *Chem. Commun*., 2016, **52**, 4207-4210.
[12] J. S. Yadav, B. V. S. Reddy, C. Sreelakshmi and A. B. Rao, *Synthesis*, 2009, **11**, 1881-1885.
[13] L. Zhang, Y. Tang, Z. Han and K. Ding, *Angew. Chem. Int. Ed.*, 2019, **58**, 4973–4977.
[14] S. Samadi, S. Nazari, H. Arvinnezhad, K. Jadidi and B. Notash, *Tetrahedron*, 2013, **69**, 6679-6686.
[15] T. Touge, T. Hakamata, H. Nara, T. Kobayashi, N. Sayo, T. Saito, Y. Kayaki and T. Ikariya, *J. Am. Chem. Soc*., 2011, **133**, 14960-14963.
[16] A. Takahashi, Y. Ogura, M. Enomoto and S. Kuwahara, *Tetrahedron*, 2016, **72**, 6634-6639.
[17] T. Yildiz and A. Yusufoglu, *Tetrahedron Asymmetry*, 2011, **22**, 1347-1352.
[18] K. H. Yong and J. M. Chong, *Org. Lett.*, 2002, **4**, 4139-4142.
[19] P. Roszkowski, J. K. Maurin and Z. Czarnocki, *Tetrahedron Asymmetry*, 2013, **24**, 643-650.
[20] Y. Li, S. Yu, X. Wu, J. Xiao, W. Shen, Z. Dong and J. Gao, *J. Am. Chem. Soc*., 2014, **136**, 4031–4039.
[21] J. L. Olivares-Romero and E. Juaristi, *Tetrahedron*, 2008, **64**, 9992-9998.
[22] L. Zeng, H. Yang, M. Zhao, J. Wen, J. H. R. Tucker and X. Zhang, *ACS Catal*., 2020, **10**, 23, 13794-13799.
[23] C. Yin, X.-Q. Dong and X. Zhang, *Adv. Synth. Catal*., 2018, **360**, 4319-4324.
[24] L. Zhang, L. Zhang, Q. Chen, L. Li, J. Jiang, H. Sun, C. Zhao, Y. Yang and C. Li, *Org. Lett*., 2022, **24**, 415-419.
[25] P. Vittorelli, J. Peter-Katalinic, G. Mukherjee-Muller, H.-J. Hansen and H. Schmid, *Helv. Chim. Acta*, 1975, **58**, 1379-1425.
[26] Y. Kyung, M. Magre and M. Rueping, *Org. Lett*., 2019, **21**, 8349-8352.

DFT Computations

Section 2.1: Computational Details

The DFT methodology was chosen following a benchmarking study of the heterolytic Mn−H bond strengths for a series of 3d transition metal hydride complexes.[1]

Geometry optimisations were performed with the BP86 functional[2,3] using the double-ζ, def2-SVP basis set from the redefinition of the Ahlrichs family of basis sets.[4-7] As a pure functional, density fitting is available and the RI (resolution of identity) approximation has been used to reduce the computational cost whilst using an ultrafine integration grid (99 radial shells with 590 angular points per shell). Implicit solvation was considered through the use of IEF-PCM (Integral Equation Formalism variant of the Polarisable Continuum Model) employing the parameters of ethanol (ε = 24.85).[8-10] All species were formally treated as closed-shell systems and restricted DFT was used throughout. The nature of minima and transition states located were verified by the computation of harmonic frequencies at the same level of theory and transition states were subject to further intrinsic reaction coordinate calculations to formally verify the corresponding minima.[11] Single-point energies were evaluated using the hybrid PBE0 functional[12-14] with a larger, triple-ζ, def2-TZVP basis. Correctional terms to treat dispersion were included with the DFT-D3 empirical correction by Grimme,[15] including Becke-Johnson dampening[16] and implicit solvation corrections were included with this level of theory, using the same ultrafine integration grid (99,590). Thermochemistry was evaluated at 1 atm and 298.15 K using thermodynamic calculations at the level of geometry optimisation in combination with energetics obtained from single-point calculations. Gibbs free energy was calculated at the elevated reaction temperature of 323.15 K using Equation 1, with additional Martin, Hay, Pratt empirical entropic corrections included (S_{MHP} = 3.929 kcal/mol per particle, evaluated at 454 atm to mimic bulk ethanol following the derivation outlined below).[17] All computations were performed using the Gaussian16, C.01 programme.[18] Topographic steric maps (main paper, Figure 2) were generated using SambVca 2.1[19] and visualisation of non-covalent interactions was performed using NCIPlot 4.0 (main paper, Scheme 2).[20]

$$\Delta G_{323.15} = \Delta H_{298.15} - T\,\Delta S_{298.15} \qquad [1]$$

Section 2.2: Derivation of S_{MHP} Entropic Correction

As calculations are performed in the gas phase, with implicit solvation around the cavity, it can be difficult to accurately predict the entropic contribution to Gibbs Free Energy of molecules. In solution, the solvent imposes additional restrictions on translational degrees of freedom that is not accounted for in gas phase calculations.[21] To compensate for this shortcoming, Martin, Hay and Pratt[17] introduced an empirical correction derived by artificially raising the pressure of gaseous water to that of the solvent phase, 1354 atm. In a reaction of mR ⇔ nP, this corresponds to a correctional term of (n - m) x 4.3 kcal/mol, irrespective of the nature of the particles involved in the reaction. This arises due to a change in the number of degrees of translational freedom when there is a change in particle numbers that must be corrected for. The use of this empirical correction has been used by our group in previous studies of catalytic systems and has been shown to be of importance in the prediction of stereochemical outcomes.[1,22-28]

The derivation of the correctional term, for the solvent ethanol, is shown below:

$$p = \frac{\rho RT}{M} = 454.40 \text{ atm}, \qquad [2]$$

$$S_{MHP} = RT\,ln\frac{p}{p^{\circ}} = 3.929 \text{ kcal/mol per particle}, \qquad [3]$$

where, p = EtOH pressure, ρ = density of EtOH (0.789 g/cm^3), M = molar mass of EtOH (46.07 g/mol), T = 323.15 K, $p°$ = 1 atm, R = ideal gas constant (8.314 J/K.mol) and S_{MHP} = Martin, Hay, Pratt entropic correctional term.

Section 2.3: Coordination Mode of Tridentate Ligand

From X-ray crystallography the pre-catalyst was found to bind facially.[29] Different conformers for the pre-catalyst have been explored *in silico* and are shown below in Table **S1**. All computations have been performed using simplified aryl phosphine groups of phenyl rather than the experimental 4-methoxy-3,5-dimethylphenyl groups.

Table S1. Comparison of the different coordination modes in the pre-catalyst.

**Relative Energies (ΔG_{323} kcal/mol)**	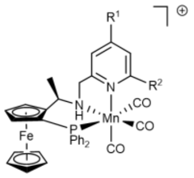	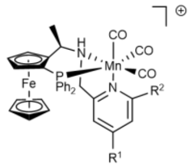	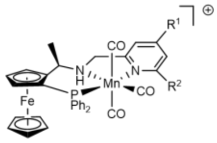	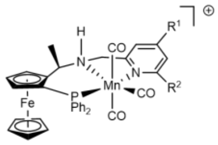
**(R_CS_P)-1′:** R^1= H, R^2= H	0.0	+4.7	+6.0	+8.6
**(R_CS_P)-3′:** R^1= NMe_2, R^2= H	0.0	+4.4	+5.9	+7.5
**(R_CS_P)-4′:** R^1= H, R^2= NMe_2	0.0	+4.0	+5.0	+9.0

The lowest energy structure across all three catalyst analogues was found to be in agreement with the conformer observed in the crystal structure.

Table S2. Comparison of the different coordination modes in the active catalyst.

**Relative Energies (ΔG_{323} kcal/mol)**	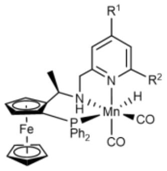	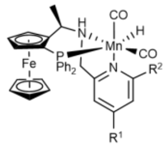	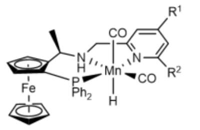	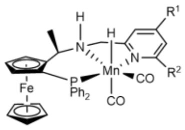
**(R_CS_P)-1′:** R^1= H, R^2= H	0.0	+4.3	−1.1	+6.2
**(R_CS_P)-3′:** R^1= NMe_2, R^2= H	0.0	+3.7	−0.8	+6.2
**(R_CS_P)-4′:** R^1= H, R^2= NMe_2	0.0	+3.5	−1.2	+5.1

While the lowest energy binding mode for the active catalyst was computed to be a meridional coordination mode (Table **S2**), it has been shown in the main paper (Figure **2**) that this conformer will not be active in the catalytic cycle as the hydride is blocked by the bulky ferrocene unit, disfavouring the approach of a substrate.

From the steric maps presented in Figure **2** and the energies of transition states shown in Figures **S1-S3**, clearly the meridional coordination mode is not a favourable route through the catalytic cycle. With a stable pre-catalyst in a facial binding mode, the interconversion via partial ligand dissociation to the meridional mode is hypothesised to be unfavourable and would also serve to raise the subsequent barrier for substrate reduction.

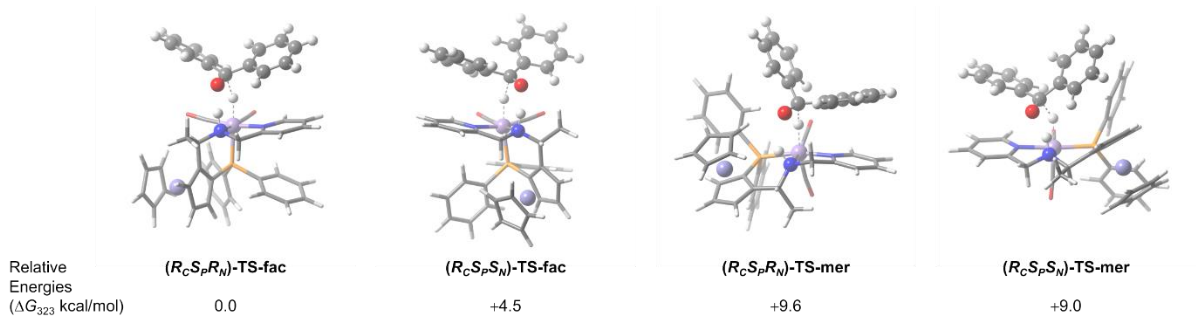

Figure S1. Hydride transfer transition state for different conformations of catalyst **1** with the symmetric benzophenone substrate.

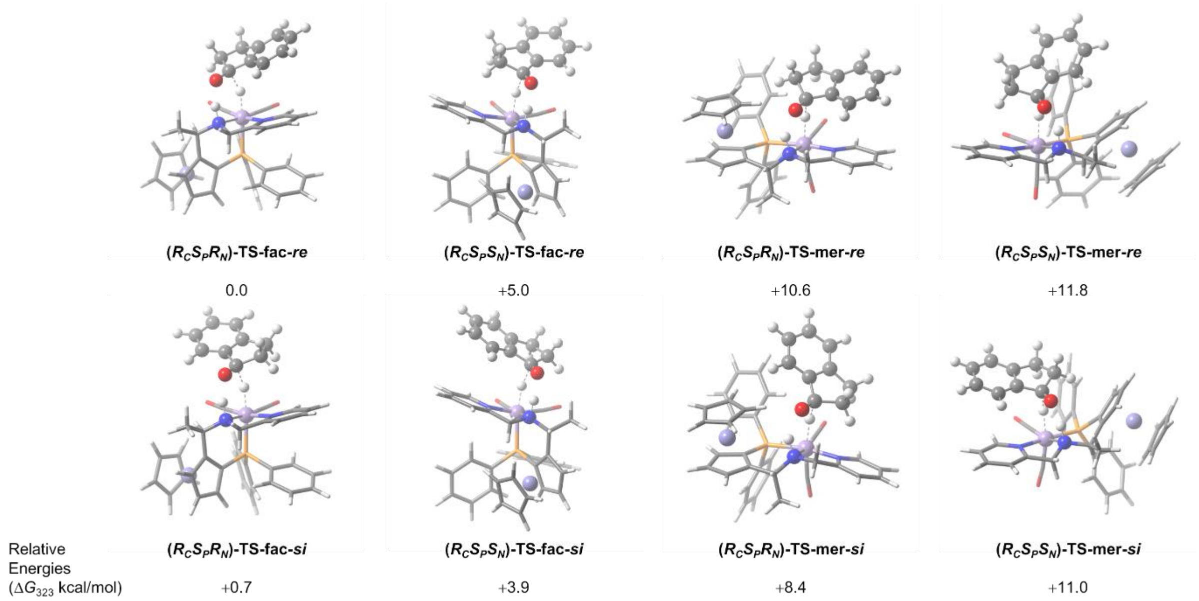

Figure S2. Hydride transfer transition state for different conformations of catalyst **1** with each prochiral face of the substrate indanone.

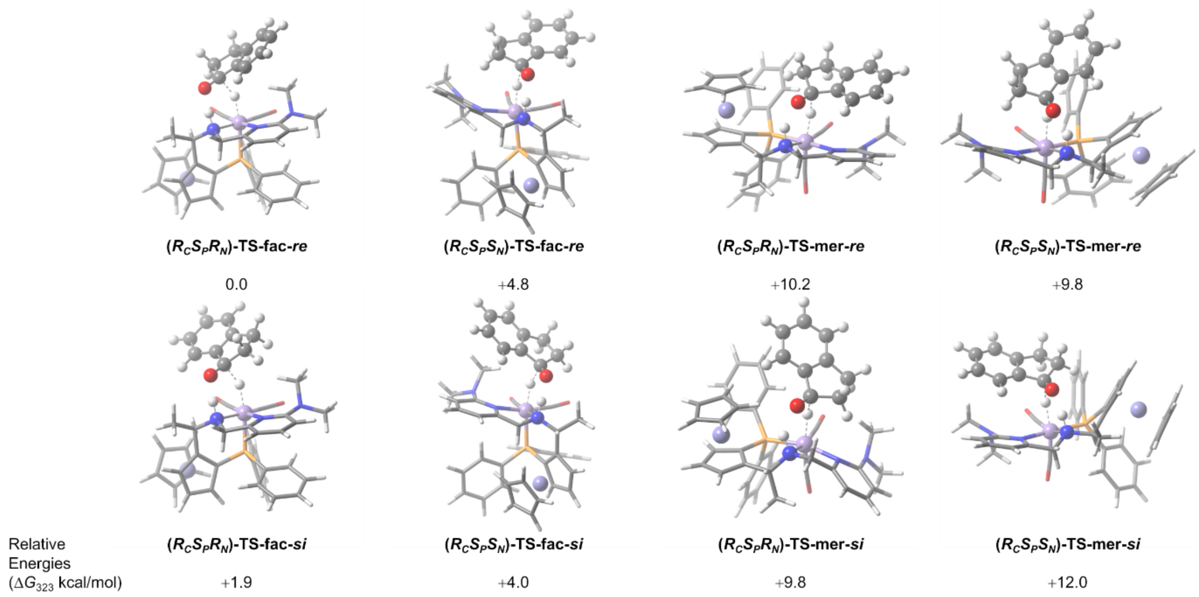

Figure S3. Hydride transfer transition state for different conformations of catalyst **4’** with each prochiral face of the substrate indanone.

Section 2.4: Transition State IRCs

IRCs have been traced for formal verification of the transition states described through this work. These calculations have been performed for the ortho substituted ligand system for the three transition states described in the main paper. Selected examples are shown in Figure **S4** below, highlighting, where applicable, concerted but asynchronous transfer of both H atoms.

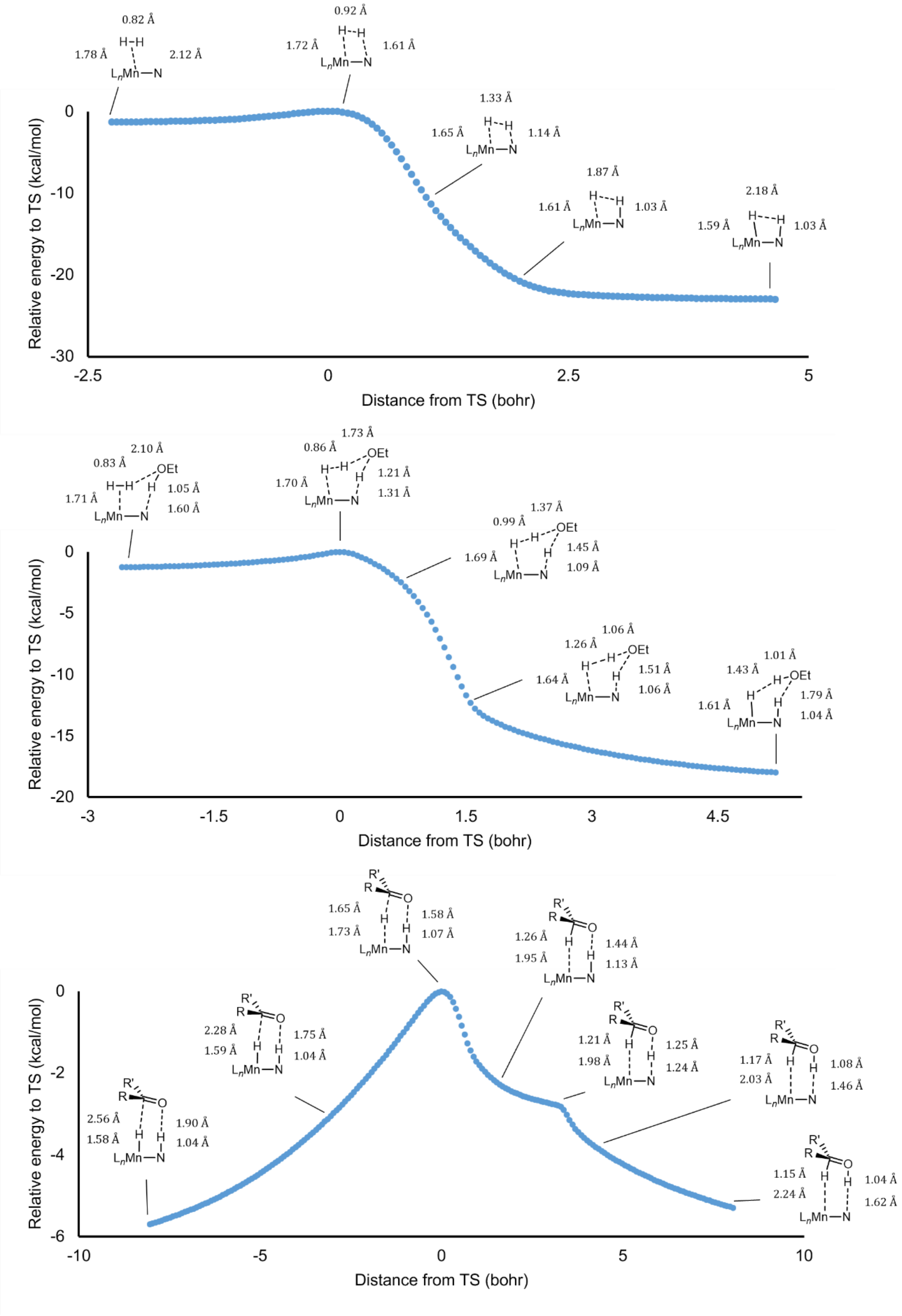

Figure S4. Intrinsic reaction coordinate for inner-sphere H_2 activation (top), outer-sphere H_2 activation (middle) and asynchronous reduction of *si*-face of indanone (bottom).

Section 2.5: Off-Cycle Species

By considering solely the active species lying on the catalytic cycle, the overall reaction barrier is 12.4 kcal/mol, obtained as the difference between the lowest solvated hydrogenated catalyst and the separated products [−4.2−(−8.5) = +4.3 kcal/mol] plus the barrier from the reactant active complex to the outer-sphere TS (8.1−0 = 8.1 kcal/mol), according to Shaik's energy span model.[30] This barrier would be low for a reaction that proceeds experimentally at 50 °C. Consequently, it is likely that the true reaction barrier, or energy span should be larger, and is likely to involve off-cycle species that are more stable than those that lie on-cycle. In solution, a variety of different solvated species are likely to exist and a number of these have been examined in order to determine a more reasonable barrier for the overall reaction.

A few solvated species have been explored and are presented on the reaction profile in Figure **S5**, including an Mn−ethoxide species and a Mn−bicarbonate species. The latter was chosen because of the base present, K_2CO_3. Due to the nature of the solvent model used, it can be challenging to accurately describe specific solvent-solute interactions that may stabilise these species, and can be advantageous to include a number of explicit solvent molecules. As shown on the reaction profile in Figure **S5**, additional explicit solvent molecules can help to stabilise these species, improving upon the simplistic continuum model by describing specific solvent-solute interactions. By including these off-cycle species, the overall calculated reaction barrier is raised to >20 kcal/mol which arguably is closer to the “real” barrier (which is unknown at this time), possibly presenting a lower limit as other, yet more stable off-cycle species may exist that have not been considered.

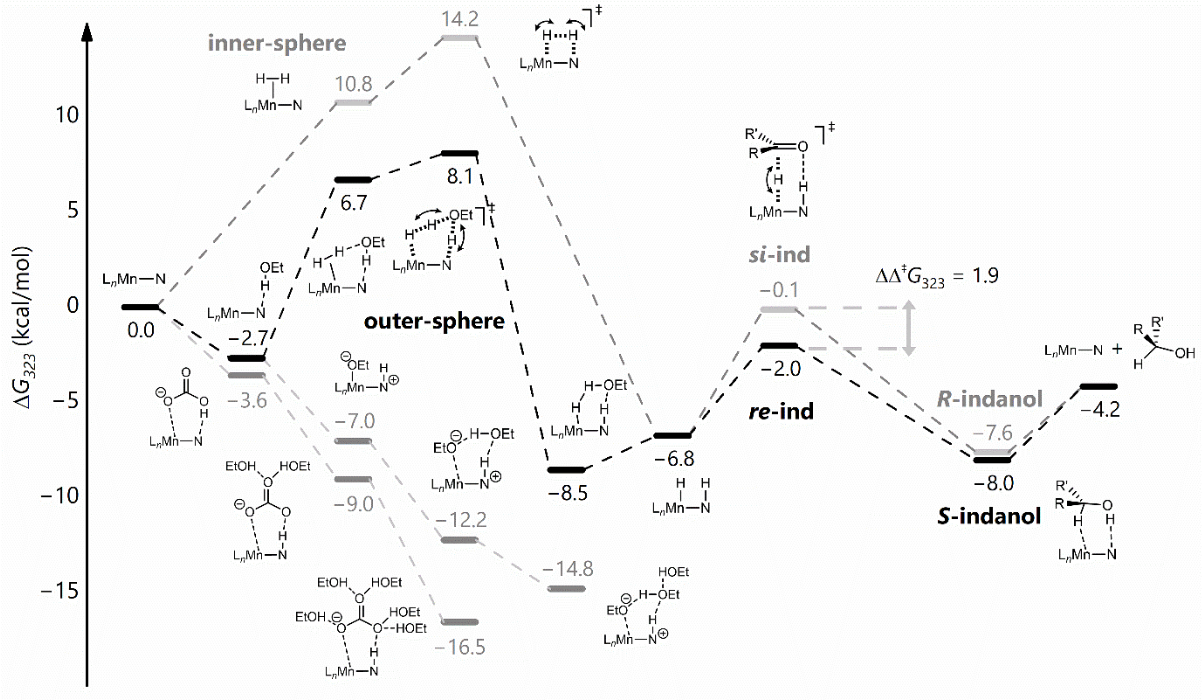

Figure S5. Potential free energy surface for the hydrogenation of indanone using the *ortho*-substituted catalyst **(*R*C*S*P)-4’** including additional off-cycle intermediates.

While the penta-solvated bicarbonate structure (at −16.5 kcal/mol in Figure **S5**) is the most stable of the off-cycle intermediates studied here, its large (and conformationally highly flexible) explicit solvation shell makes for a rather cumbersome model for comparison across different systems. Due to the large excess of ethanol present over carbonate, we chose the simpler, mono-solvated ethoxide structure (at −12.2 kcal/mol) as a representative resting state. Note that none of these considerations affect the main part of this study, stereoselectivity in any way.

Section 2.6: Origin of Stereoselectivity

The *ortho*-substituted catalyst has been show to perform a variety of reductions stereoselectively, with the preferential reduction of ketones from specific prochiral faces. For indanone, this preference (obtained as difference between the two diastereomeric TSs) amounts to $\Delta\Delta^{\ddagger}G$ = 1.91 kcal/mol with catalyst **(*R*C*S*P)-4’** corresponding to an *er* of 95:5. This bias in ΔG arises in part from a stronger interaction energy for this *re*-face transition state. Interaction energies have been calculated in the gas phase for the diastereomers of the transition state, with single-point calculations of the fragments using the geometry of the transition state, showing that the

re-face has a stronger interaction between fragments than the *si*-face ($\Delta\Delta E_{int}$ = −3.48 kcal/mol, half of it due to dispersion interactions, as inferred from separate evaluation of the Grimme dispersion correction, viz. $\Delta\Delta D3$ = −1.74 kcal/mol). This can be attributed to the more favourable interaction between the substrate and the *ortho*-dimethylamino group of the pyridine ring of the ligand backbone. This can be observed in the area attributed to a CH−π interaction shown in the NCI plot in Figure **S4**, where the planar unsaturated system can more favourably orientate above the bulk of the NMe_2 group. Similarly, for 44-dimethylcyclohexen-1-one, $\Delta\Delta^{‡}G$ = −0.74 kcal/mol, biased by a stronger interaction energy with the C=C bond orientated above the NMe_2 group ($\Delta\Delta E_{int}$ = −1.58 kcal/mol, dominated by $\Delta\Delta D3$ = −1.08 kcal/mol).

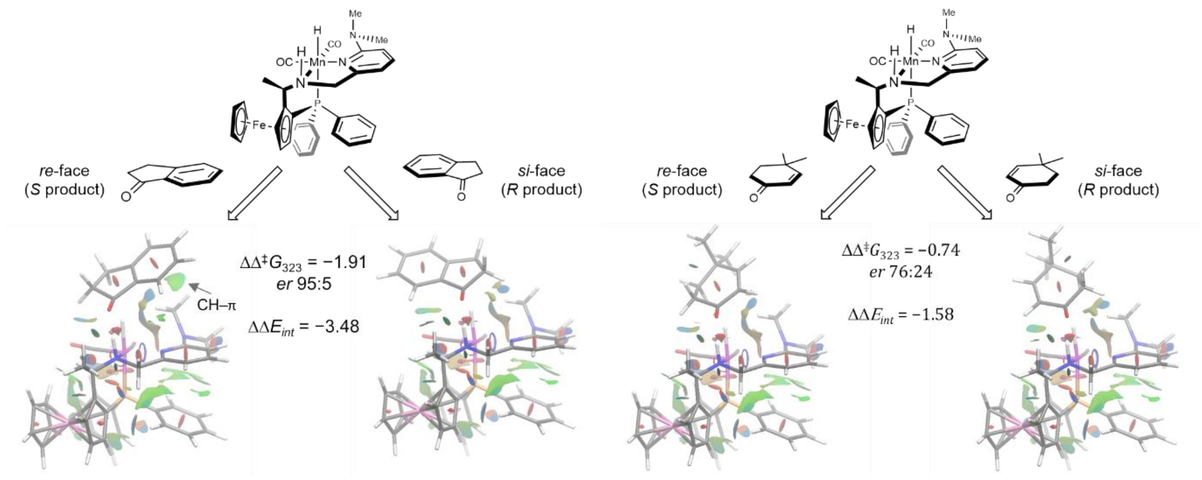

Figure S6. Comparison of the stereodefining transition states of indanone and 44-dimethylcyclohexen-1-one using catalyst **(R_CS_P)-4'**. Areas with attractive non-covalent interactions shown in green.

Table S3. Visualisation of diastereomeric transition states for all substrates using catalyst **(R_CS_P)-4'** corresponding to Table 1 in the main paper. Negative energies favour the *re*-face. Energies in kcal/mol and distances in Å.

	***re*-face (*S*)**	$\Delta^{‡}G_{323}$<br><br>*C–H*<br>*Mn–H*	$\Delta\Delta^{‡}G_{323}$<br>*er*<br>$\Delta\Delta^{‡}E$<br>$\Delta\Delta^{‡}D3$	$\Delta^{‡}G_{323}$<br><br>*C–H*<br>*Mn–H*	***si*-face (*R*)**	$\Delta\Delta E_{int}$<br>$\Delta\Delta D3_{int}$<br>**gas phase**
**5ke**<br>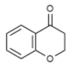	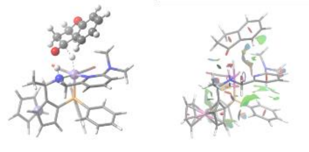	**2.76**<br><br>*1.688*<br>*1.703*	**−1.81**<br>94:6 (*S*)<br>−2.11<br>+1.39	**4.57**<br><br>*1.660*<br>*1.730*	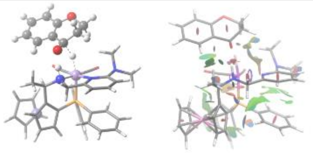	−3.28<br>−1.16
**6ke**<br>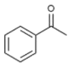	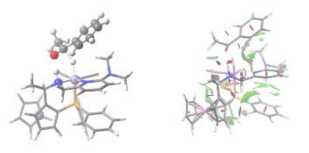	**4.29**<br><br>*1.687*<br>*1.708*	**−1.84**<br>95:5 (*S*)<br>−1.51<br>−0.99	**6.13**<br><br>*1.687*<br>*1.697*	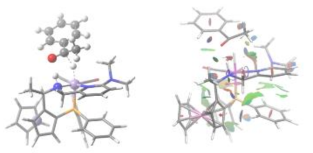	−3.52<br>−0.77
**7ke**<br>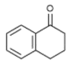	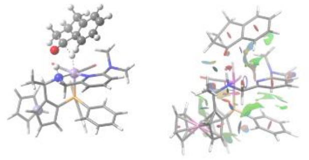	**3.60**<br><br>*1.669*<br>*1.712*	**−1.84**<br>95:5 (*S*)<br>−1.90<br>+1.76	**5.44**<br><br>*1.643*<br>*1.740*	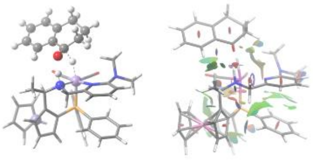	−2.36<br>−0.83

Continued on next page

Table S3 – Continued from previous page

	re-face (*S*)	$\Delta^{\ddagger}G_{323}$<br>*C–H*<br>*Mn–H*	$\Delta\Delta^{\ddagger}G_{323}$<br>er<br>$\Delta\Delta^{\ddagger}E$<br>$\Delta\Delta^{\ddagger}D3$	$\Delta^{\ddagger}G_{323}$<br>*C–H*<br>*Mn–H*	*si*-face (*R*)	$\Delta\Delta E_{int}$<br>$\Delta\Delta D3_{int}$<br>gas phase
**8ke**<br>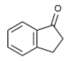	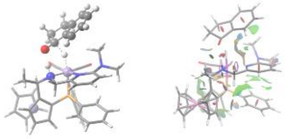	**4.73**<br><br>*1.662*<br>*1.713*	**−1.91**<br>95:5 (*S*)<br>−2.42<br>−0.16	**6.64**<br><br>*1.655*<br>*1.729*	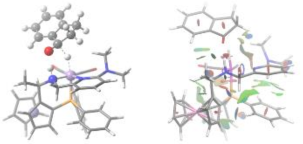	−3.48<br>−1.74
**9ke**<br>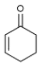	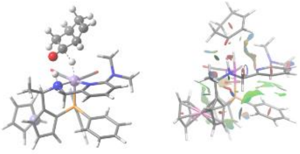	**5.72**<br><br>*1.641*<br>*1.711*	**−1.87**<br>95:5 (*S*)<br>−0.89<br>+1.00	**7.59**<br><br>*1.616*<br>*1.734*	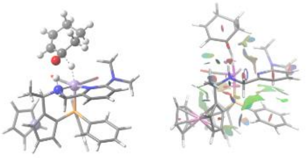	−1.26<br>−1.12
**10ke**<br>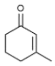	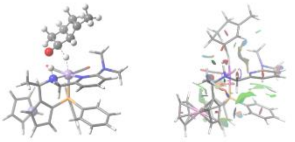	**6.88**<br><br>*1.608*<br>*1.718*	**−0.50**<br>69:31 (*S*)<br>−0.61<br>+1.47	**7.38**<br><br>*1.583*<br>*1.743*	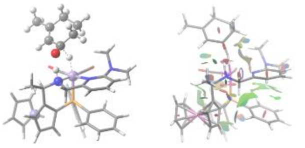	−0.75<br>−0.77
**11ke**<br>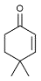	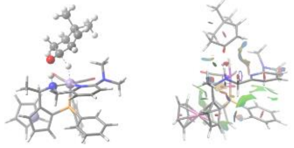	**5.68**<br><br>*1.641*<br>*1.717*	**−0.74**<br>76:24 (*S*)<br>−0.91<br>+0.84	**6.42**<br><br>*1.622*<br>*1.733*	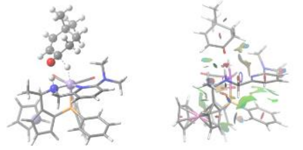	−1.58<br>−1.08
**12ke**<br>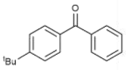	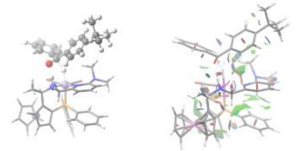	**2.73**<br><br>*1.721*<br>*1.697*	**−0.39**<br>65:35 (*S*)<br>−0.18<br>−0.07	**3.12**<br><br>*1.733*<br>*1.698*	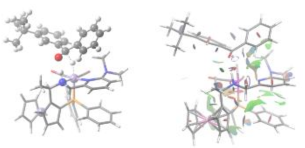	−0.12<br>−0.13
**13ke**<br>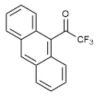	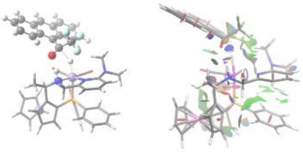	**4.42**<br><br>*2.029*<br>*1.677*	**−0.49**<br>68:32 (*S*)<br>−0.70<br>+0.43	**4.91**<br><br>*1.965*<br>*1.683*	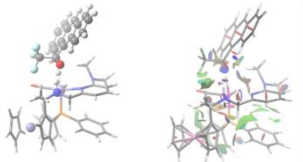	+2.45<br>+0.46
**15ke**<br>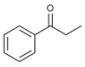	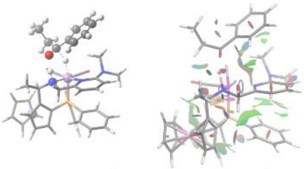	**5.34**<br><br>*1.705*<br>*1.712*	**−0.60**<br>72:28 (*S*)<br>−0.26<br>+0.44	**5.93**<br><br>*1.711*<br>*1.707*	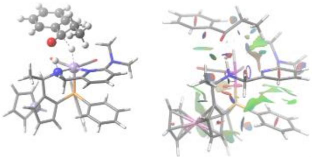	−1.40<br>+0.71
**16ke**<br>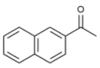	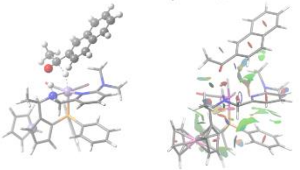	**4.11**<br><br>*1.685*<br>*1.708*	**−1.38**<br>89:11 (*S*)<br>−1.93<br>−1.32	**5.48**<br><br>*1.713*<br>*1.693*	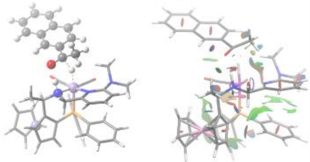	−4.14<br>−1.21
**17ke**<br>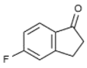	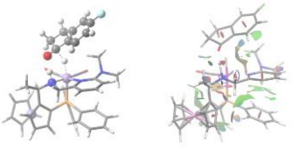	**4.84**<br><br>*1.658*<br>*1.714*	**−1.43**<br>90:10 (*S*)<br>−2.31<br>−0.12	**6.27**<br><br>*1.651*<br>*1.729*	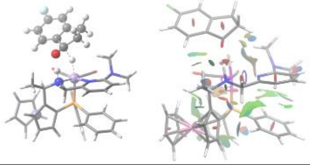	−3.35<br>−1.80

Continued on next page

Table S3 – Continued from previous page

	re-face (*S*)	$\Delta^{\ddagger}G_{323}$<br>*C–H*<br>*Mn–H*	$\Delta\Delta^{\ddagger}G_{323}$<br>*er*<br>$\Delta\Delta^{\ddagger}E$<br>$\Delta\Delta^{\ddagger}D3$	$\Delta^{\ddagger}G_{323}$<br>*C–H*<br>*Mn–H*	*si*-face (*R*)	$\Delta\Delta E_{int}$<br>$\Delta\Delta D3_{int}$<br>gas phase
**19ke**<br>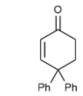 	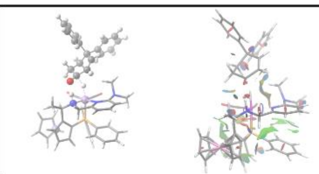	**3.18**<br><br>*1.667*<br>*1.707*	**−1.82**<br>94:6 (*S*)<br>−1.74<br>+0.19	**5.00**<br><br>*1.644*<br>*1.727*	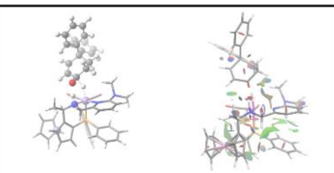	−2.67<br>−1.66
**20ke**<br>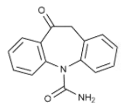 	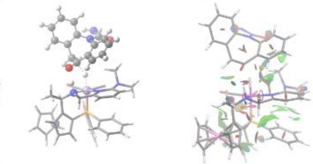	**2.61**<br><br>*1.698*<br>*1.700*	**−2.38**<br>98:2 (*S*)<br>−2.01<br>−0.36	**4.98**<br><br>*1.661*<br>*1.713*	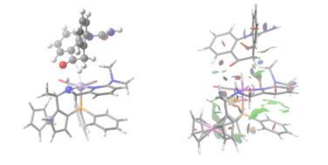	−2.71<br>−0.60
**22ke**<br>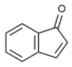 	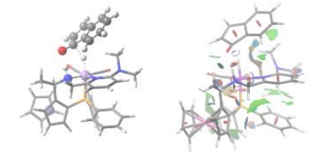	**0.30**<br><br>*1.728*<br>*1.701*	**−1.27**<br>88:12 (*S*)<br>−1.57<br>−0.94	**1.58**<br><br>*1.703*<br>*1.709*	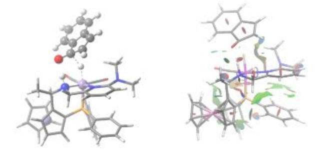	−1.45<br>−1.06
**24ke**<br>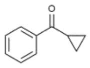 	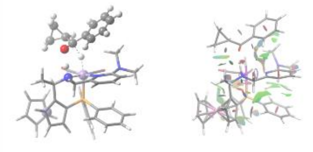	**4.52**<br><br>*1.675*<br>*1.713*	**−1.17**<br>86:14 (*S*)<br>−0.57<br>−0.92	**5.69**<br><br>*1.708*<br>*1.698*	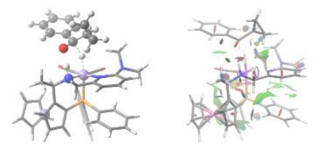	−3.24<br>−0.68
**26ke**<br>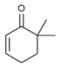 	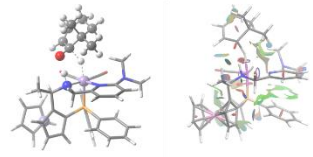	**7.25**<br><br>*1.615*<br>*1.780*	**+0.71**<br>25:75 (*R*)<br>+1.24<br>−0.58	**6.55**<br><br>*1.630*<br>*1.752*	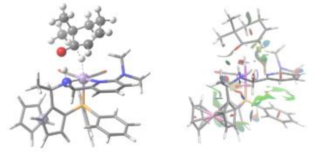	−1.00<br>−0.24
**27ke**<br>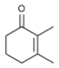 	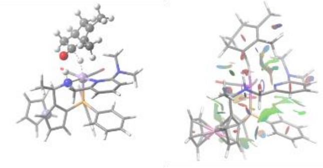	**6.27**<br><br>*1.585*<br>*1.738*	**−1.57**<br>92:8 (*S*)<br>−1.40<br>+2.10	**7.84**<br><br>*1.630*<br>*1.752*	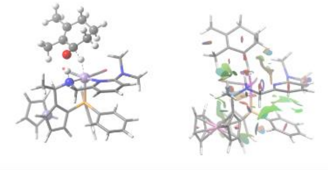	+0.12<br>+0.70

Section 2.7: Discussion regarding further substrates that were considered (either experimentally or computationally).

In this paper the catalyst was designed to operate with ketones with a sp^3 alkyl group on one side and a less three-dimensional substituent on the other (e.g. sp^2). Further to Table **1** in the main paper, some additional substrates were considered and, prompted by a comment from a reviewer and our desire to report as much as possible, we have discussed these here (Scheme **S1**). In the course of post-rationalising the selectivity of the catalyst for indanone, the enantioselectivity for reduction of the related indenone, 22ke was predicted (see Table **S3** below). This was not studied experimentally for several reasons: in the course of previous studies on hydrogenations using this class of catalyst, substrates that are very activated Michael acceptors have a tendency to undergo competing conjugate reduction of the C=C bond. In this work, this was observed with cyclohexanone itself. In addition, experimentally, we had previous tested 2,3-diphenylindenone, 23ke and found C=C reduction dominated. On this basis, combined with a focus (sp3-C(=O)-sp2 ketones), indenone reduction was not studied experimentally.

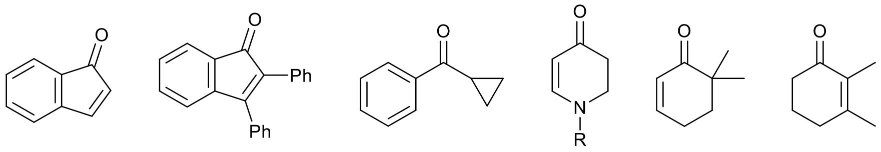

22ke **23ke** **24ke** **25ke** **26ke** **27ke**

Scheme S1. Additional substrates considered either experimentally or computationally.

Phenyl-cyclopropyl ketone, 24ke was evaluated computationally as being reduced reasonably highly selectively, and was never evaluated experimentally (rather arbitrarily, although we note we would not expect for the DFT predictions to reliably pick out slight reductions in *e.r.* relative to acetophenone as seems to be the prediction here).

Experimentally, we also devoted many experiments seeking to make and study dehydropiperidinone substrates, 25ke since they were unprecedented and the products looked like very useful building blocks. However, most substrate syntheses either failed or were difficult to purify. The hydrogenation of a small amount of 25ke (R = benzoyl) was carried out and led to decomposition. Therefore if such substrates could ever be done they would need a stabilising protecting group for the enamide functionality. A reviewer wondered if we had evaluated some of the substrates in Scheme **3** computationally. Here it is worth revisiting the potential applications of this type of research. The importance comes from being able to use a relatively rapid approach (DFT calculations) to give confidence whether or not a small group of substrates are worth studying. While most substrates here were quite simple, in a real world application, a substrate might require weeks or months of work to produce. In such a scenario with complex substrates, evaluating a more simplistic model substrate should be sufficient to determine potential activity of the catalyst. With additional complexity in substrates, there are further computational considerations of conformational flexibility which will slow down the rate of evaluation and as a result, some of the larger substrates have not been examined *in silico*. Based on the computational work for the substrates shown in Table **1**, the substrates in Scheme **3** were evaluated experimentally. However, since submission of the original manuscript, a selection (ketones 15ke, 16ke, 17ke, 19ke, 20ke) have been examined computationally and are included in Table **S3** alongside some additional examples that have not been considered experimentally.

Considering the outlook beyond the scope of this paper, it seems a reasonable expectation that sufficiently good predictions could be made for this catalyst for reduction of ketones that bear a mix of sp^2 and sp^3 groups. It is also likely that the method could be used for currently unknown variants of catalyst 4 in terms of selectivity, although this might not predict if such catalysts can be made, or have sufficient activity or chemoselectivity. It is perfectly possible that further exploration will find a limit to which the predictions are good enough in terms of different types of asymmetric reductions. In our opinion, both of these may be interesting directions for further research.

Section 2.8: References

[1] A.S. Goodfellow and M. Bühl, *Molecules*, 2021, **26**, 4072.
[2] A.D. Becke, *Phys. Rev. A*, 1988, **38**, 3098–3100.
[3] J.P. Perdew, *Phys. Rev. B*, 1986, **33**, 8822–8824.
[4] A. Schäfer, H. Horn, and R. Ahlrichs, *J. Chem. Phys.*, 1992, **97**, 2571–2577.
[5] A. Schäfer, C. Huber and R. Ahlrichs, *J. Chem. Phys.*, 1994, **100**, 5829–5835.
[6] F. Weigend and R. Ahlrichs, *Phys. Chem. Chem. Phys.*, 2005, **7**, 3297–3305.
[7] F. Weigend, *Phys. Chem. Chem. Phys.*, 2006, **8**, 1057–1065.
[8] B. Mennucci and J. Tomasi, *J. Chem. Phys.*, 1997, **106**, 5151–5158.
[9] J. Tomasi, B. Mennucci and E. Cancès, *J. Mol. Struct.*, 1999, **464**, 211–226.
[10] J. Tomasi, B. Mennucci and R. Cammi, *Chem. Rev.*, 2005, **105**, 2999–3093.
[11] K. Fukui, *Acc. Chem. Res.*, 1981, **14**, 363–368.
[12] J.P. Perdew, K. Burke and Y. Wang, *Phys. Rev. B*, 1996, **54**, 16533–16539.
[13] J.P. Perdew, K. Burke and M. Ernzerhof, *Phys. Rev. Lett.*, 1997, **78**, 1396.
[14] C. Adamo and V. Barone, *J. Chem. Phys.*, 1999, **110**, 6158–6170.
[15] S. Grimme, J. Antony, S. Ehrlich and H. Krieg, *J. Chem. Phys.*, 2010, **132**, 154104.
[16] S. Grimme, S. Ehrlich and L. Goerigk, *J. Comput. Chem.*, 2011, **32**, 1457–1465.
[17] R.L. Martin, P.J. Hay and L.R. Pratt, *J. Phys. Chem. A*, 1998, **102**, 3565–3573.
[18] M.J. Frisch, G.W. Trucks, H.B. Schlegel, G.E. Scuseria, M.A. Robb, J.R. Cheeseman, G. Scalmani, V. Barone, G.A. Petersson, H. Nakatsuji, X. Li, M. Caricato, A.V. Marenich, J. Bloino, B.G. Janesko, R. Gomperts, B. Mennucci, H.P. Hratchian, J.V. Ortiz, A.F. Izmaylov, J.L. Sonnenberg, D. Williams-Young, F. Ding, F. Lipparini, F. Egidi, J. Goings, B. Peng, A. Petrone, T. Henderson, D. Ranasinghe, V.G. Zakrzewski, J. Gao, N. Rega, G. Zheng, W. Liang, M. Hada, M. Ehara, K. Toyota, R. Fukuda, J. Hasegawa, M. Ishida, T. Nakajima, Y. Honda, O. Kitao, H. Nakai, T. Vreven, K. Throssell, J.A. Montgomery, Jr., J.E. Peralta, F. Ogliaro, M.J. Bearpark, J.J. Heyd, E.N. Brothers, K.N. Kudin, V.N. Staroverov, T.A. Keith, R. Kobayashi, J. Normand, K. Raghavachari, A.P. Rendell, J.C. Burant, S.S. Iyengar, J. Tomasi, M. Cossi, J.M. Millam, M. Klene, C. Adamo, R. Cammi, J.W. Ochterski, R.L. Martin, K. Morokuma, O. Farkas, J.B. Foresman and D.J. Fox, Gaussian 16, Revision C.01, Gaussian Inc., Wallingford CT, 2019.
[19] L. Falivene, Z. Cao, A. Petta, L. Serra, A. Poater, R. Oliva, V. Scarano and L. Cavallo, *Nat. Chem.*, 2019, **11**, 872–879.
[20] R.A. Boto, F. Peccati, R. Laplaza, C. Quan, A. Carbone, J.-P. Piquemal, Y. Maday and J. Contreras-GarcÍa, *J. Chem. Theory Comput.*, 2020, **16**, 4150–4158.
[21] H. Ryu, J. Park, H.K. Kim, J.Y. Park, S.T. Kim and M.H. Baik, *Organometallics*, 2018, **37**, 3228–3239.
[22] M. Bühl, N. Sieffert and G. Wipff, *Chem. Phys, Lett.*, 2009, **467**, 287–293.
[23] M. Bühl, N. Sieffert, A. Chaumont and G. Wipff, *Inorg. Chem.*, 2012, **51**, 1943–1952.
[24] N. Sieffert and M. Bühl, *Inorg. Chem.*, 2009, **48**, 4622–4624.
[25] N. Sieffert, and M. Bühl, *J. Am. Chem. Soc.*, 2010, **132**, 8056–8070.
[26] N. Sieffert, R. Réocreux, P. Lorusso, D.J. Cole-Hamilton and M. Bühl, *Chem. Eur. J.*, 2014, **20**, 4141–4155.
[27] S. Gallarati, P. Dingwall, J.A. Fuentes, M. Bühl and M.L. Clarke, *Organometallics*, 2020, **39**, 4544–4556.
[28] A.E. Owen, A. Preiss, A. McLuskie, C. Gao, G. Peters, M. Bühl and A. Kumar, *ACS Catal.*, 2022, **12**, 6923–6933.
[29] M.B. Widegren, G.J. Harkness, A.M.Z. Slawin, D.B. Cordes and M.L. Clarke, *Angew. Chem. Int. Ed. Engl.*, 2017, **56**, 5825–5828.
[30] S. Kozuch and S. Shaik, *Acc. Chem. Res.*, 2011, **44**, 101–110.

Section 3.1: NMR/ HPLC Data

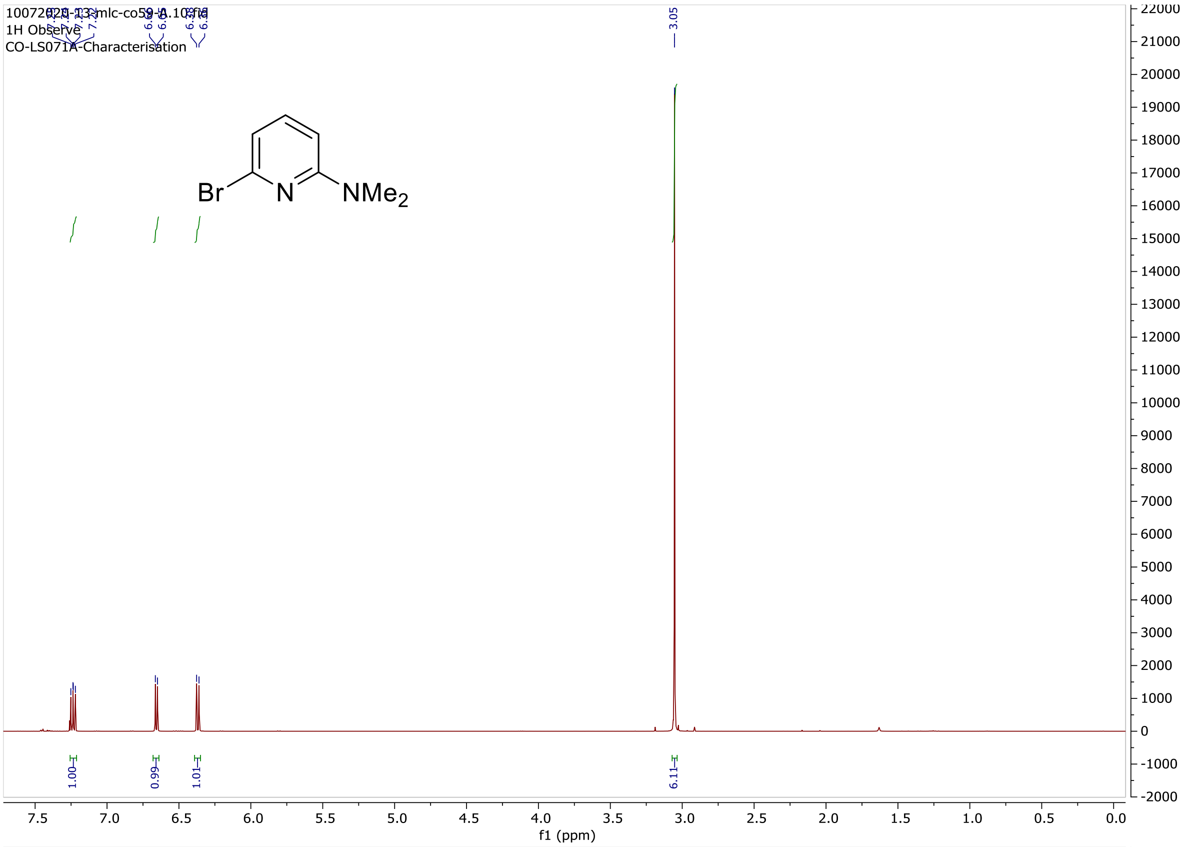

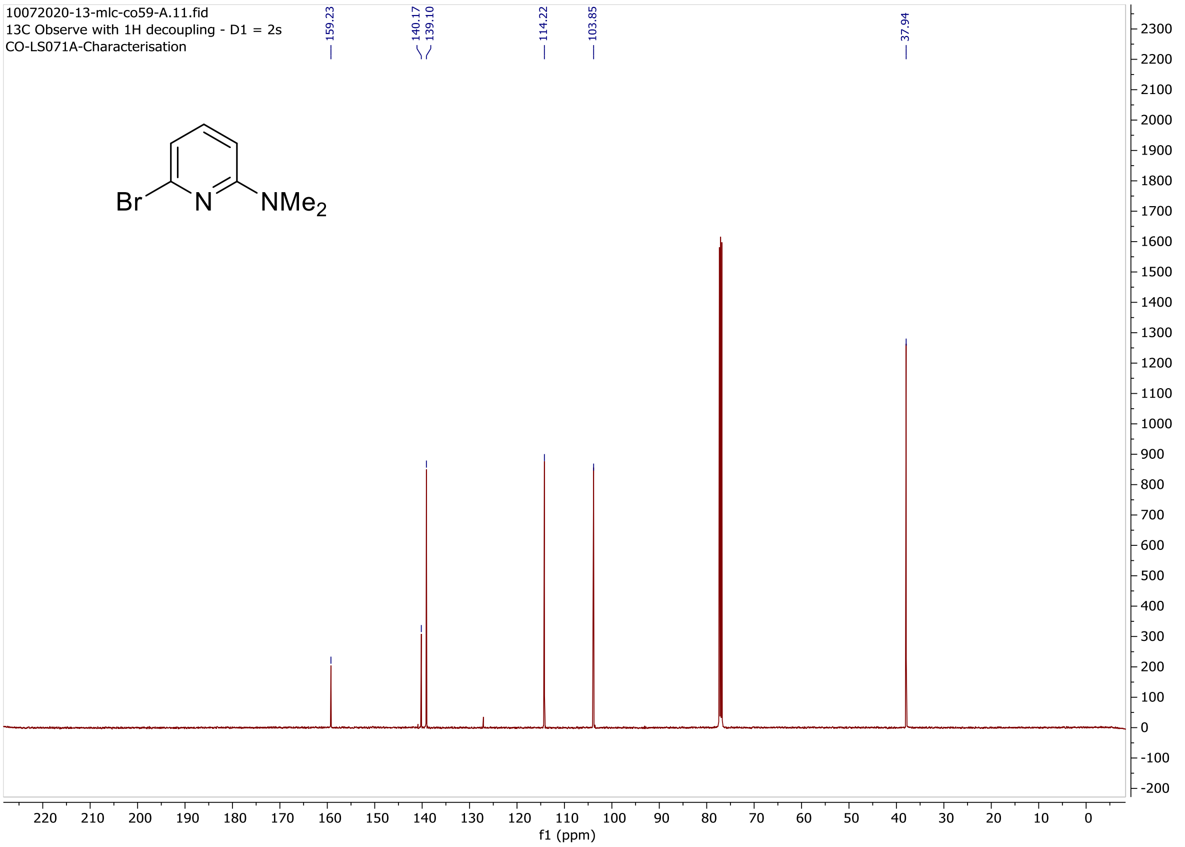

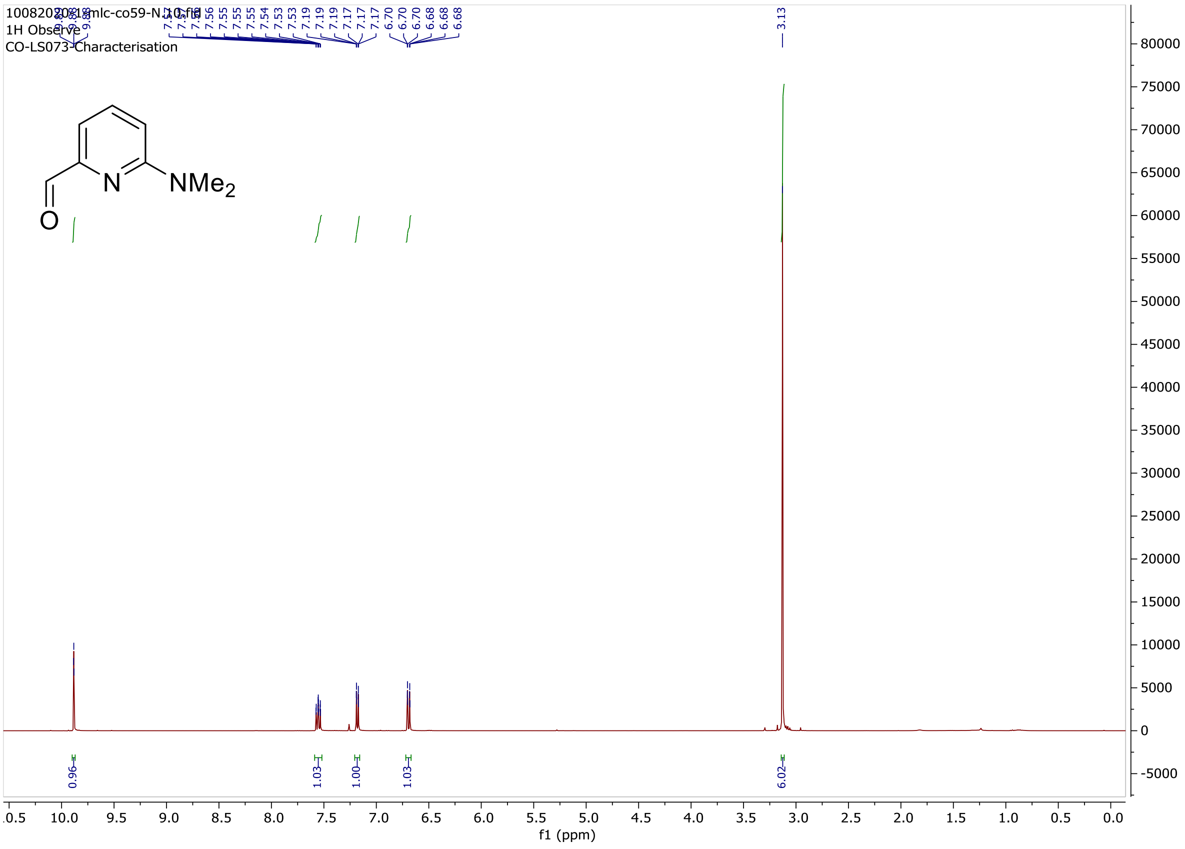
1H Observe
CO-LS073-Characterisation
NMe2
N
O
3.13
f1 (ppm)

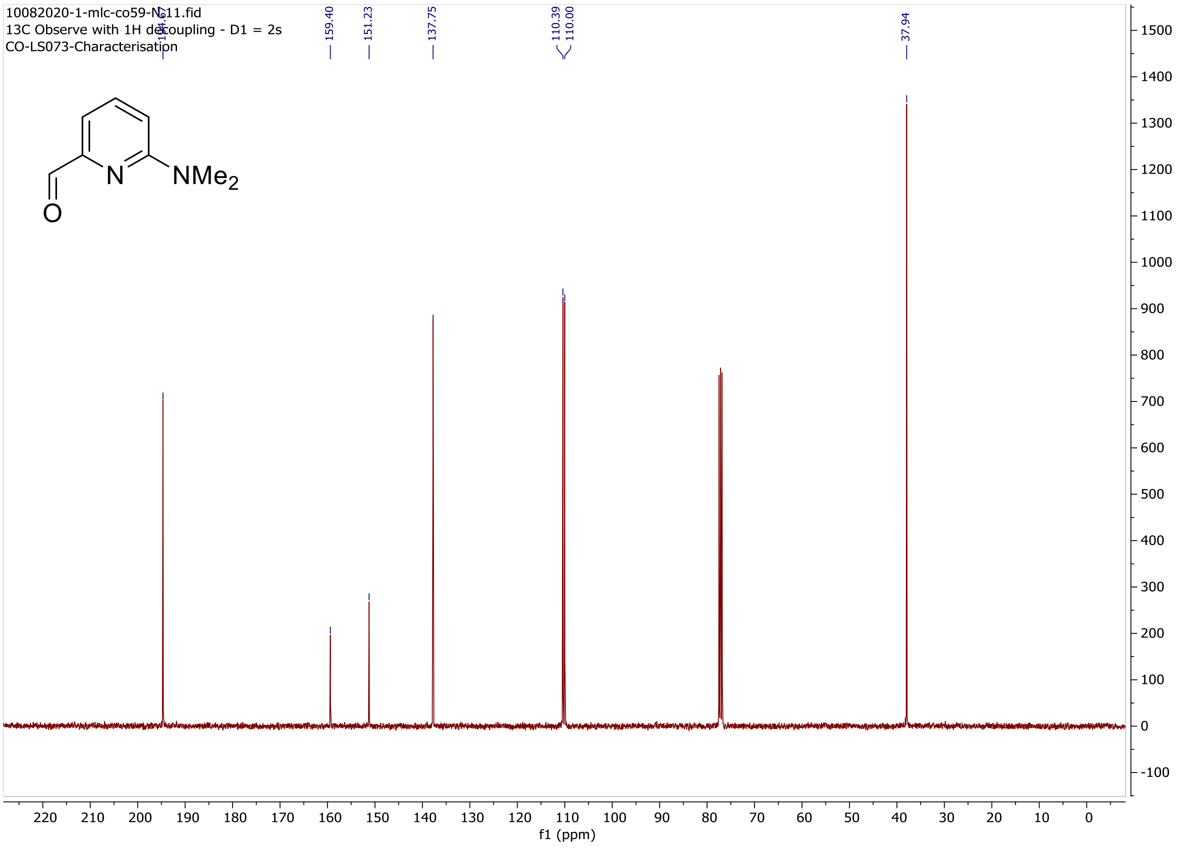
10082020-1-mlc-co59-N.11.fid
13C Observe with 1H decoupling - D1 = 2s
CO-LS073-Characterisation
159.40
151.23
137.75
110.39
110.00
37.94
NMe2
N
O
f1 (ppm)

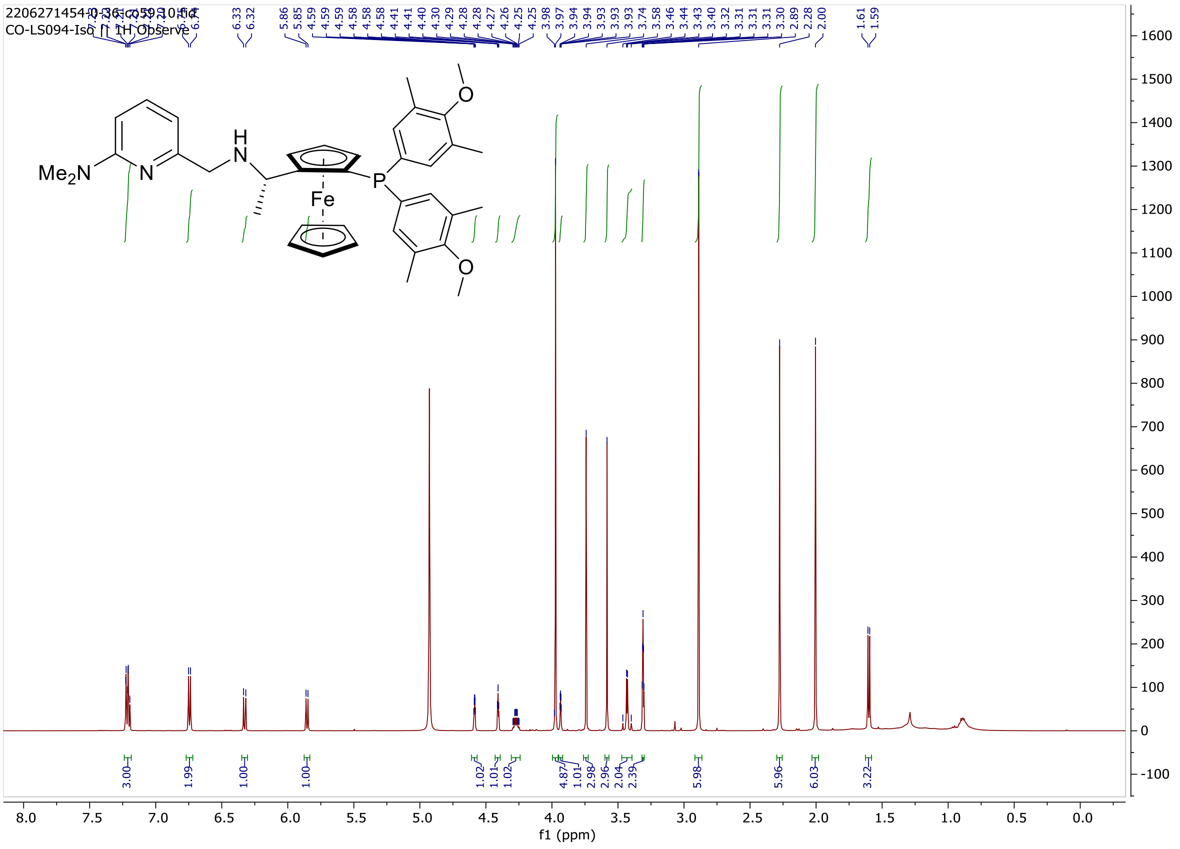
CO-LS094-Iso || 1H Observe
Me2N
N
H
N
Fe
P
O
O
f1 (ppm)

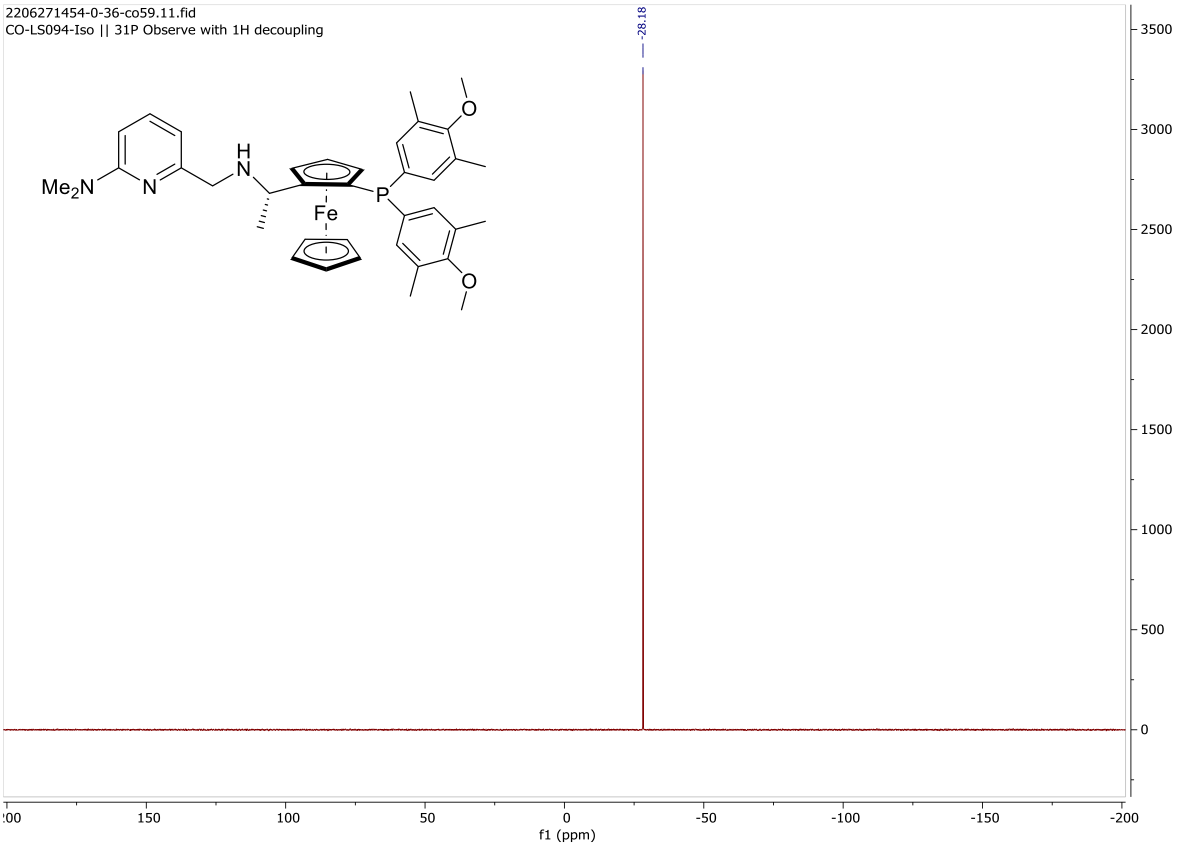
2206271454-0-36-co59.11.fid
CO-LS094-Iso || 31P Observe with 1H decoupling
-28.18
Me2N
N
H
N
Fe
P
O
O
f1 (ppm)

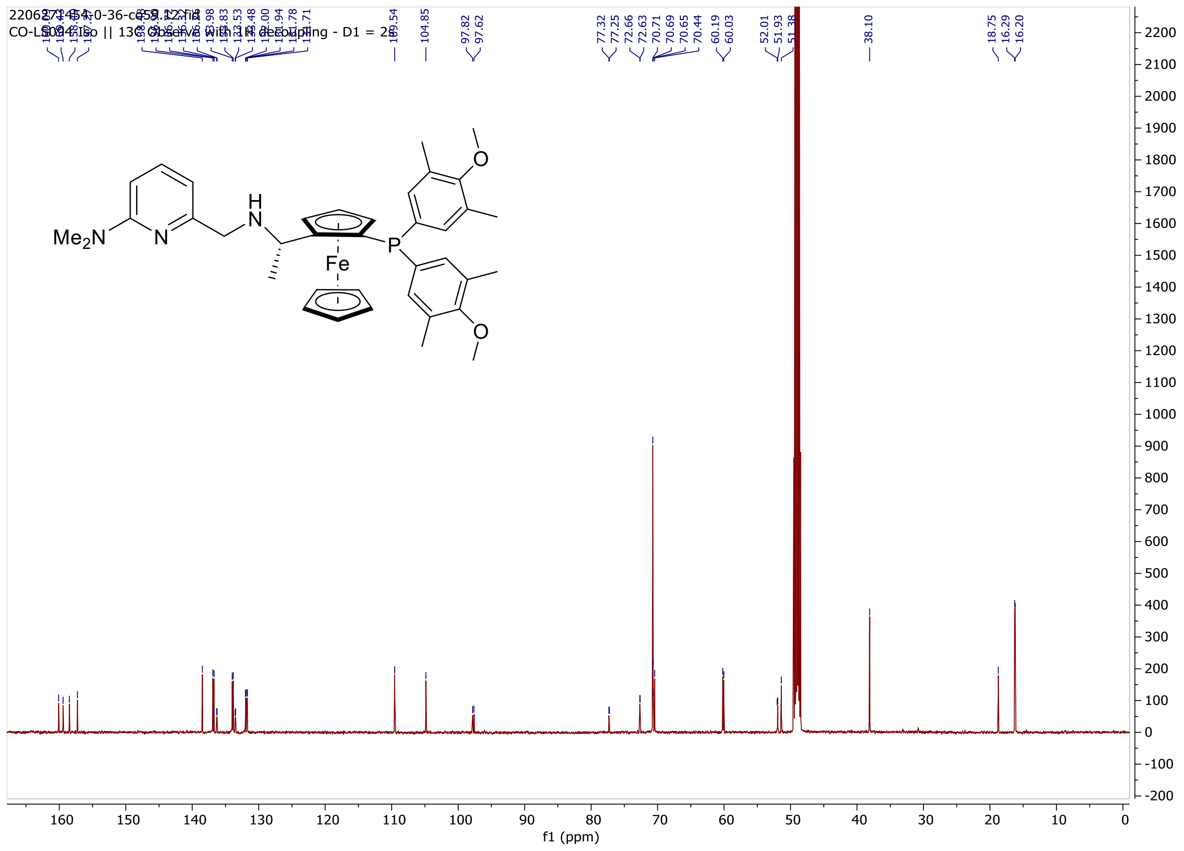
CO-LS094-Iso || 13C Observe with 1H decoupling - D1 = 2s
109.54
104.85
97.82
97.62
77.32
77.25
72.66
72.63
70.71
70.69
70.65
70.44
60.19
60.03
52.01
51.93
51.38
38.10
18.75
16.29
16.20
Me2N
N
H
N
Fe
P
O
O
f1 (ppm)

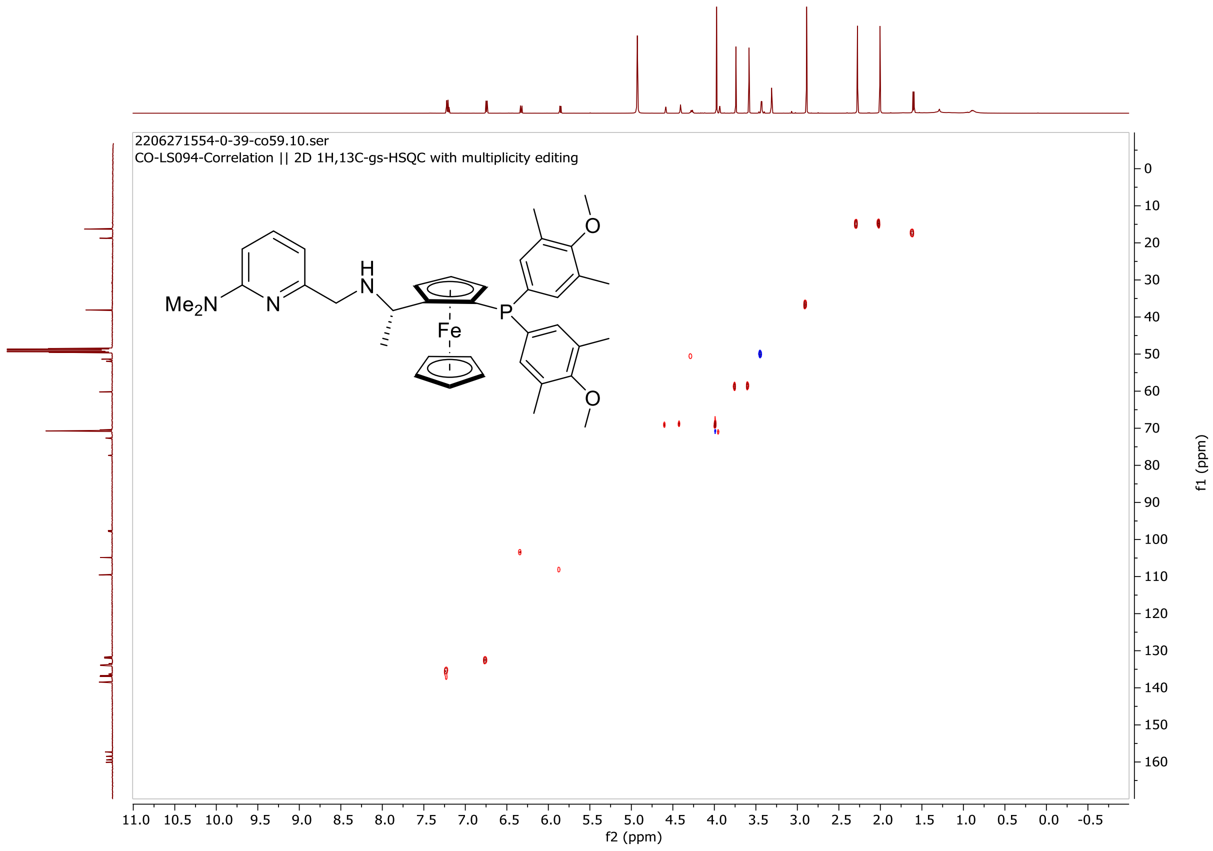
2206271554-0-39-co59.10.ser
CO-LS094-Correlation || 2D 1H,13C-gs-HSQC with multiplicity editing
Me2N
N
H
N
Fe
P
O
O
f2 (ppm)
f1 (ppm)

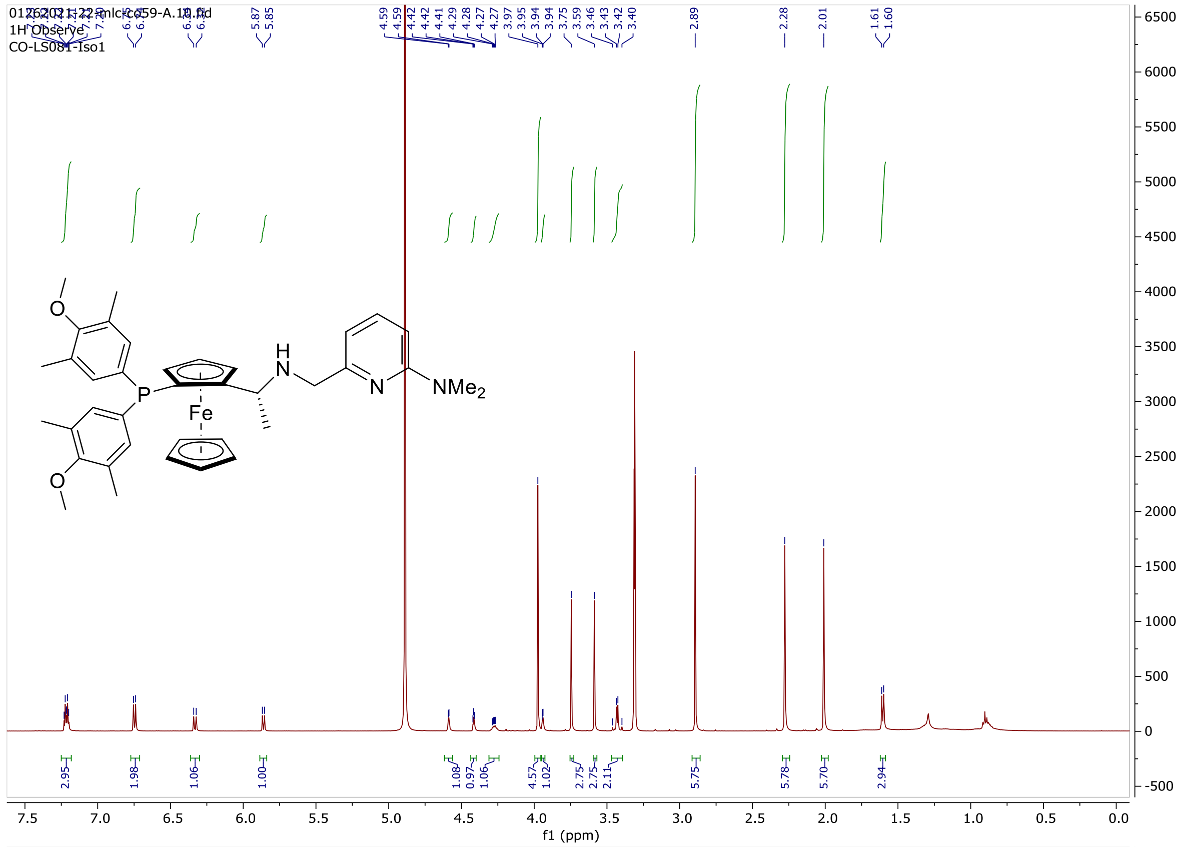
01262021-22-mlc-co59-A.10.fid
1H Observe
CO-LS081-Iso1
NMe2
Fe
f1 (ppm)

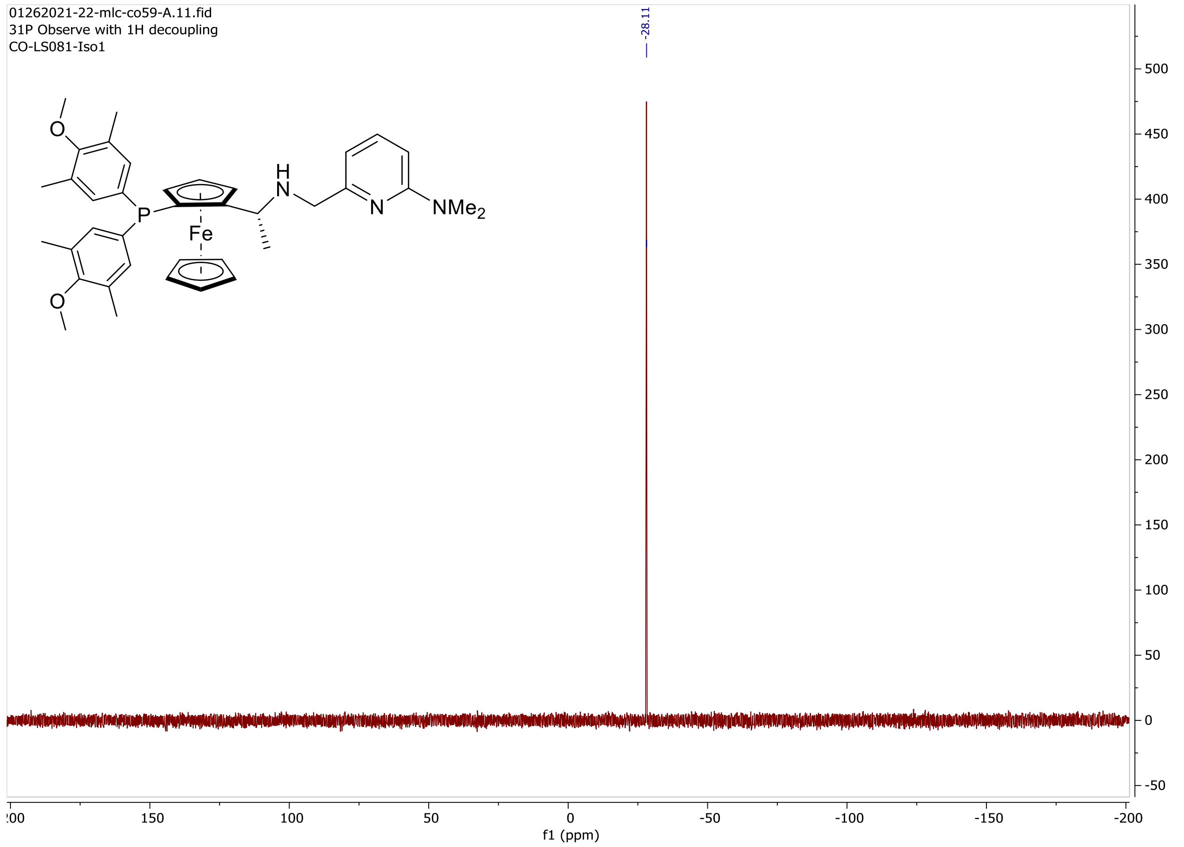
01262021-22-mlc-co59-A.11.fid
31P Observe with 1H decoupling
CO-LS081-Iso1
-28.11
NMe2
Fe
f1 (ppm)

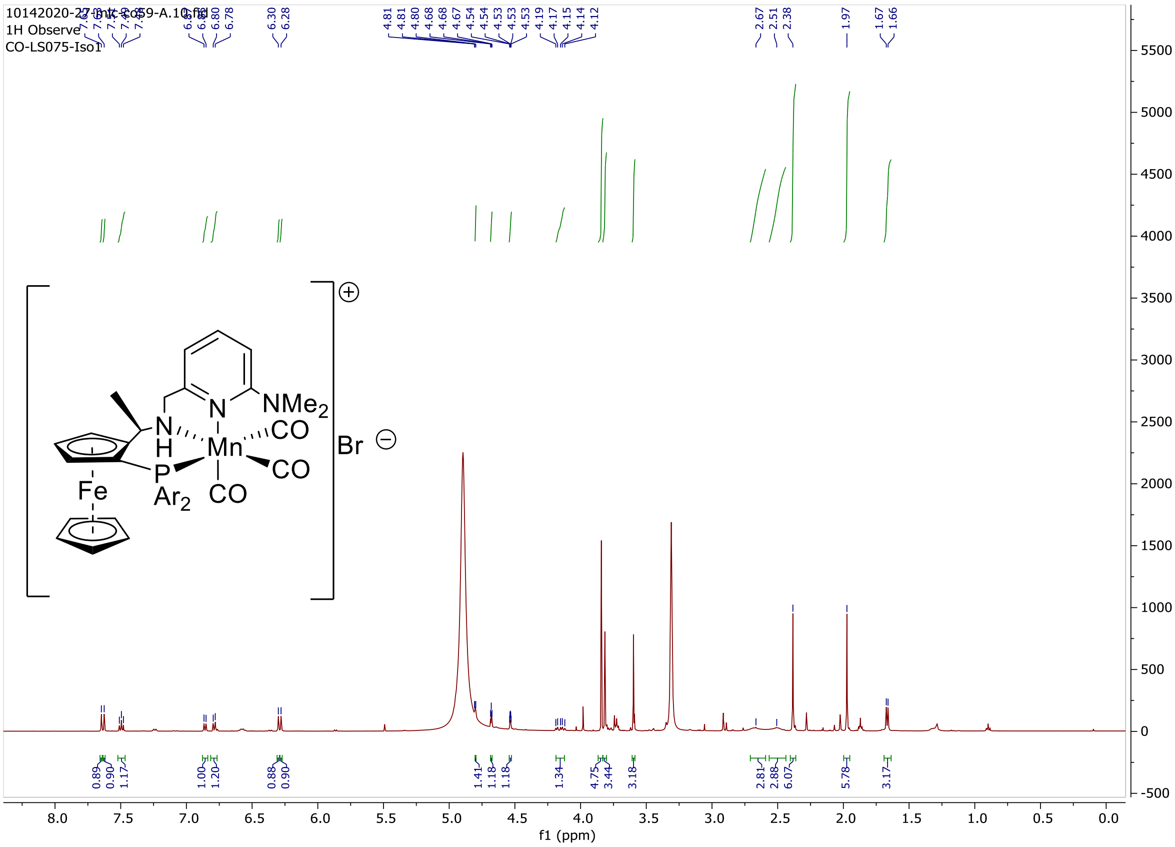

10142020-27-mlc-co59-A.10.fid
1H Observe
CO-LS075-Iso1
NMe2
N
H
Mn
CO
CO
CO
P
Ar2
Fe
Br
f1 (ppm)

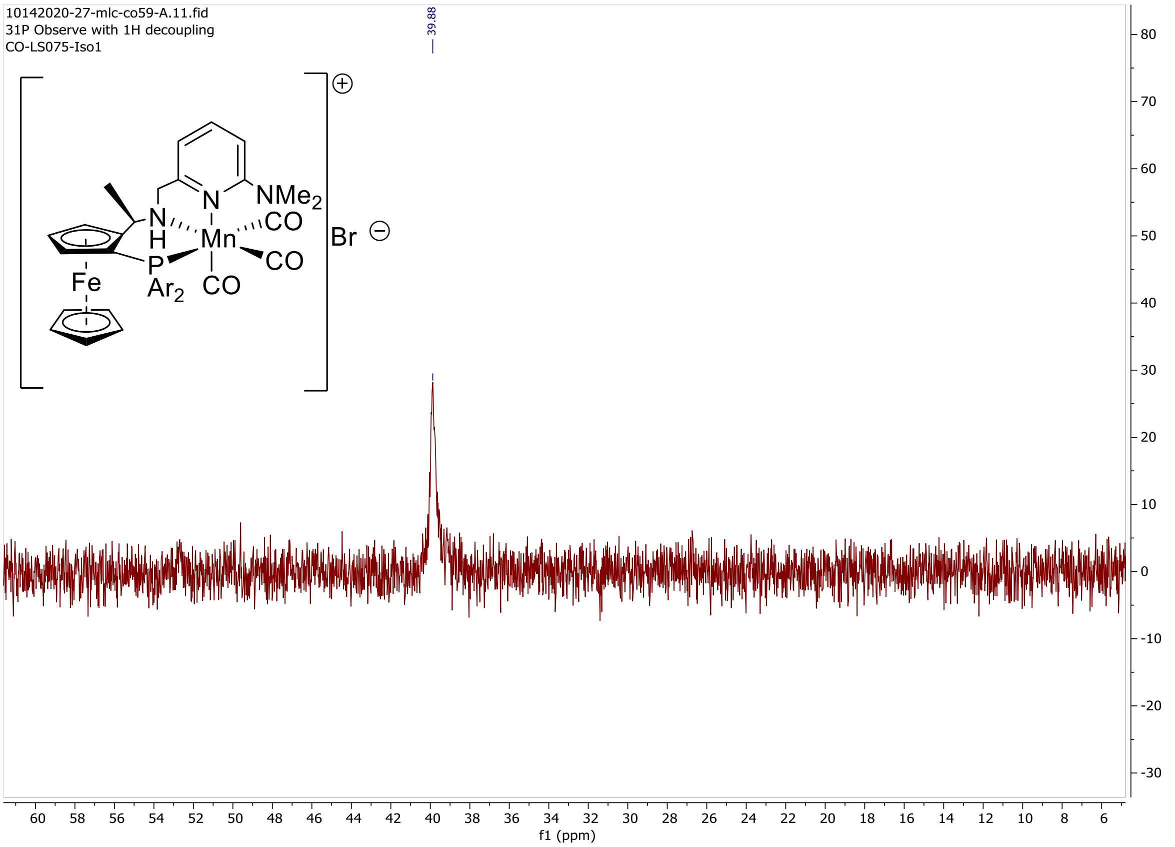

10142020-27-mlc-co59-A.11.fid
31P Observe with 1H decoupling
CO-LS075-Iso1
39.88
NMe2
N
H
Mn
CO
CO
CO
P
Ar2
Fe
Br
f1 (ppm)

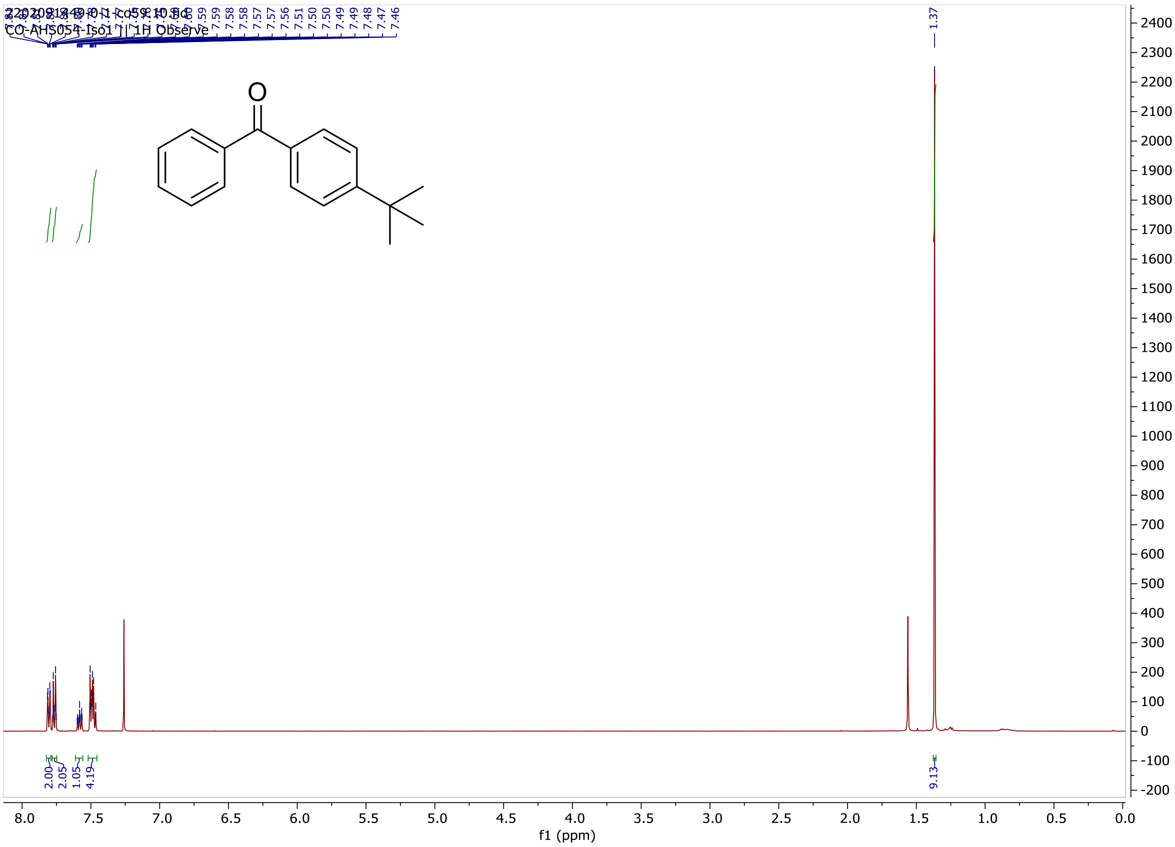
1.37
2.00
2.05
1.05
4.19
9.13
f1 (ppm)

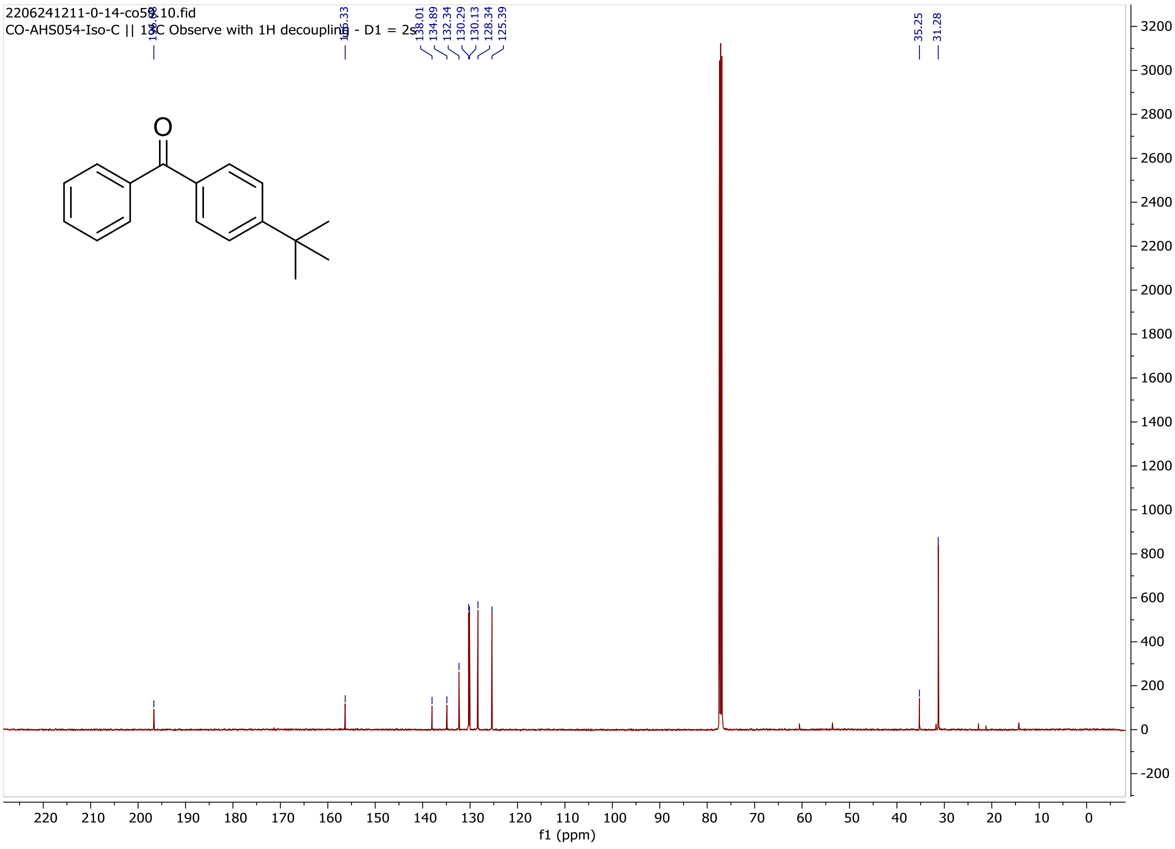
2206241211-0-14-co59.10.fid
CO-AHS054-Iso-C || 13C Observe with 1H decoupling - D1 = 2s
156.33
138.01
134.89
132.34
130.29
130.13
128.34
125.39
35.25
31.28
f1 (ppm)

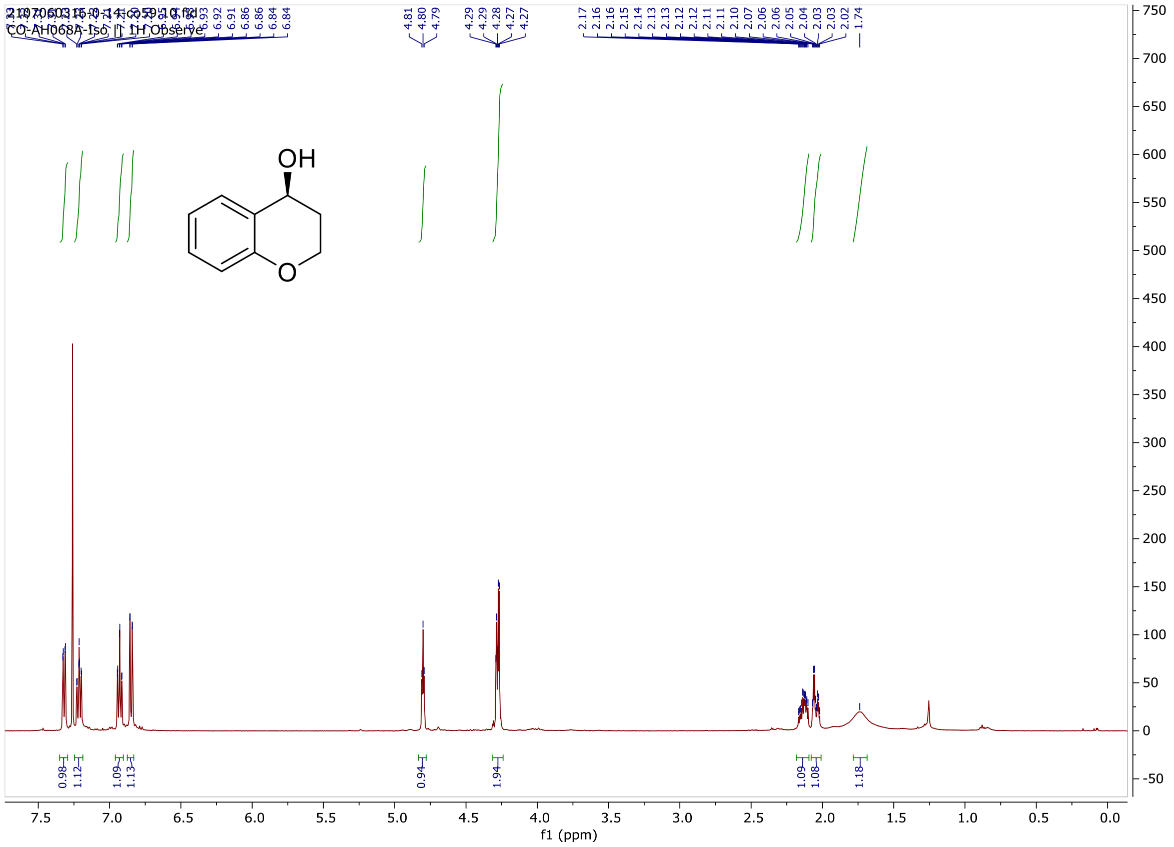
4.81
4.80
4.79
4.29
4.29
4.28
4.27
4.27
2.17
2.16
2.16
2.15
2.14
2.13
2.13
2.12
2.12
2.11
2.11
2.10
2.07
2.06
2.06
2.05
2.04
2.03
2.03
2.02
1.74
OH
O
0.98
1.12
1.09
1.13
0.94
1.94
1.09
1.08
1.18
f1 (ppm)

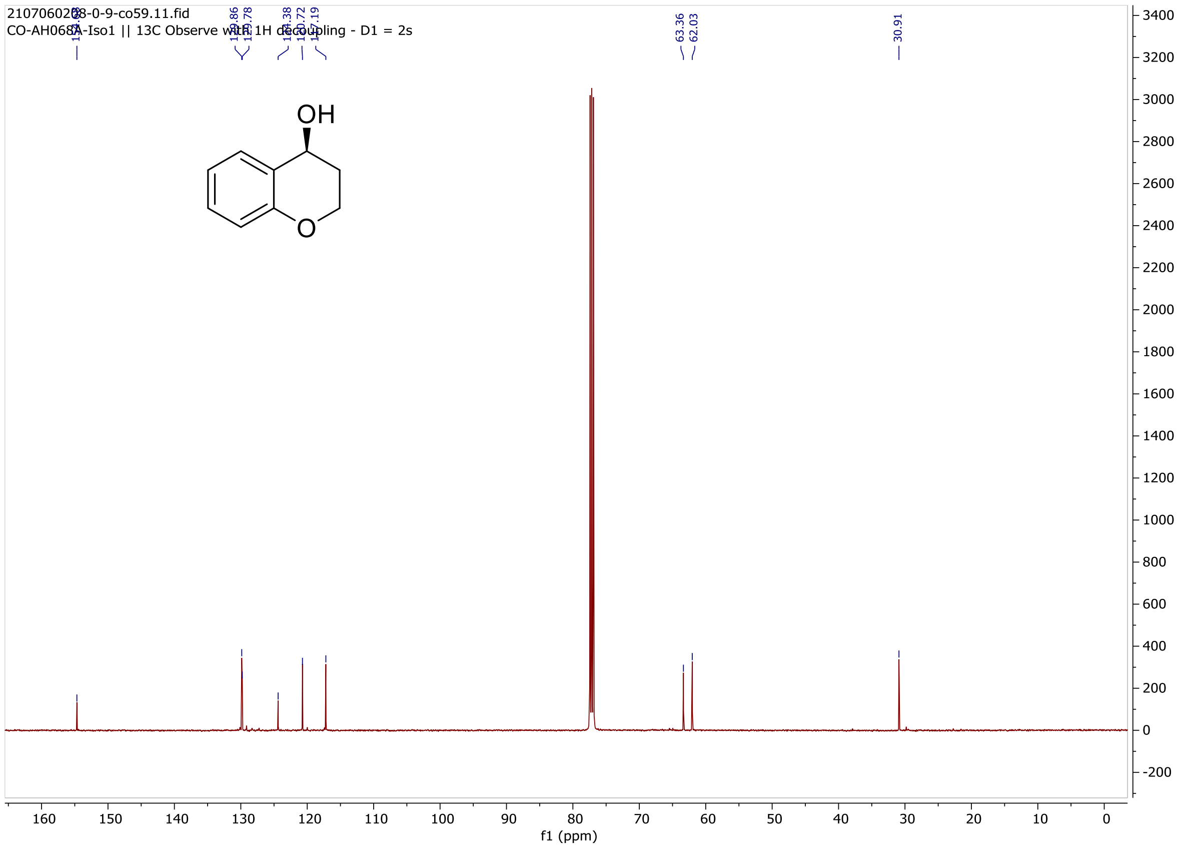
CO-AH068A-Iso1 || 13C Observe with 1H decoupling - D1 = 2s
63.36
62.03
30.91
OH
O
f1 (ppm)

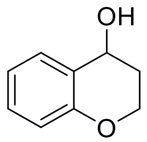

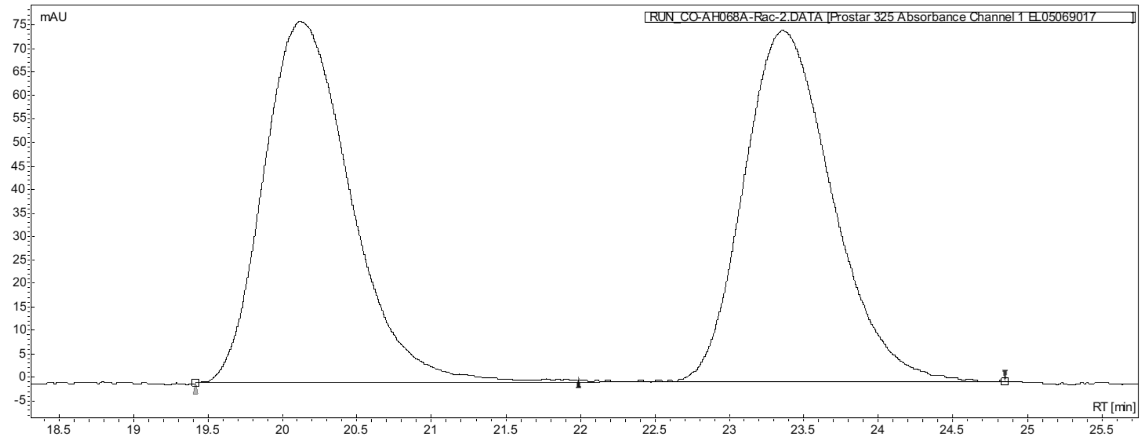

#	Name	Time [Min]	Quantity [% Are	Height [mAU]	Area [mAU.Min]	Area % [%]
1	UNKNOWN	20.12	50.17	77.1	52.3	50.173
2	UNKNOWN	23.35	49.83	74.9	51.9	49.827
Total			100.00	152.0	104.2	100.000

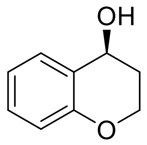

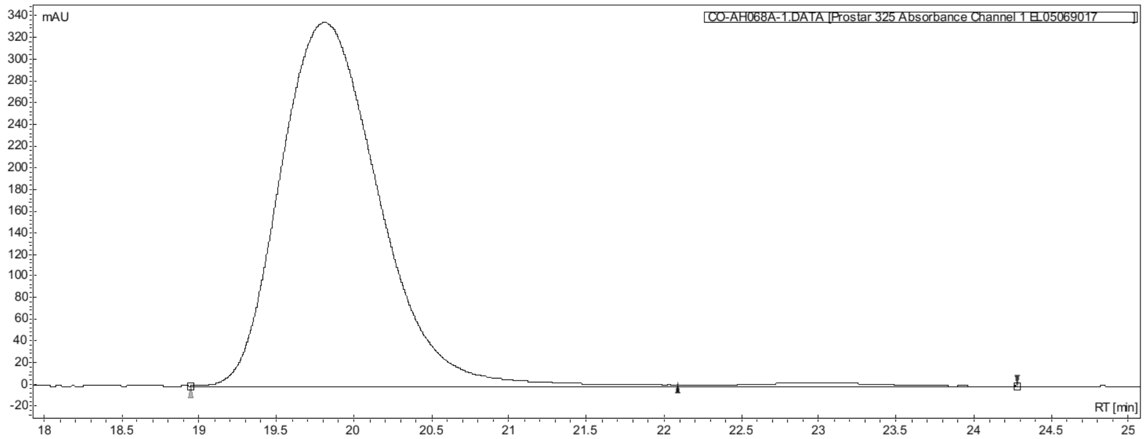

#	Name	Time [Min]	Quantity [% Are	Height [mAU]	Area [mAU.Min]	Area % [%]
1	UNKNOWN	19.81	98.95	334.9	245.8	98.947
2	UNKNOWN	23.04	1.05	3.2	2.6	1.053
Total			100.00	338.1	248.4	100.000

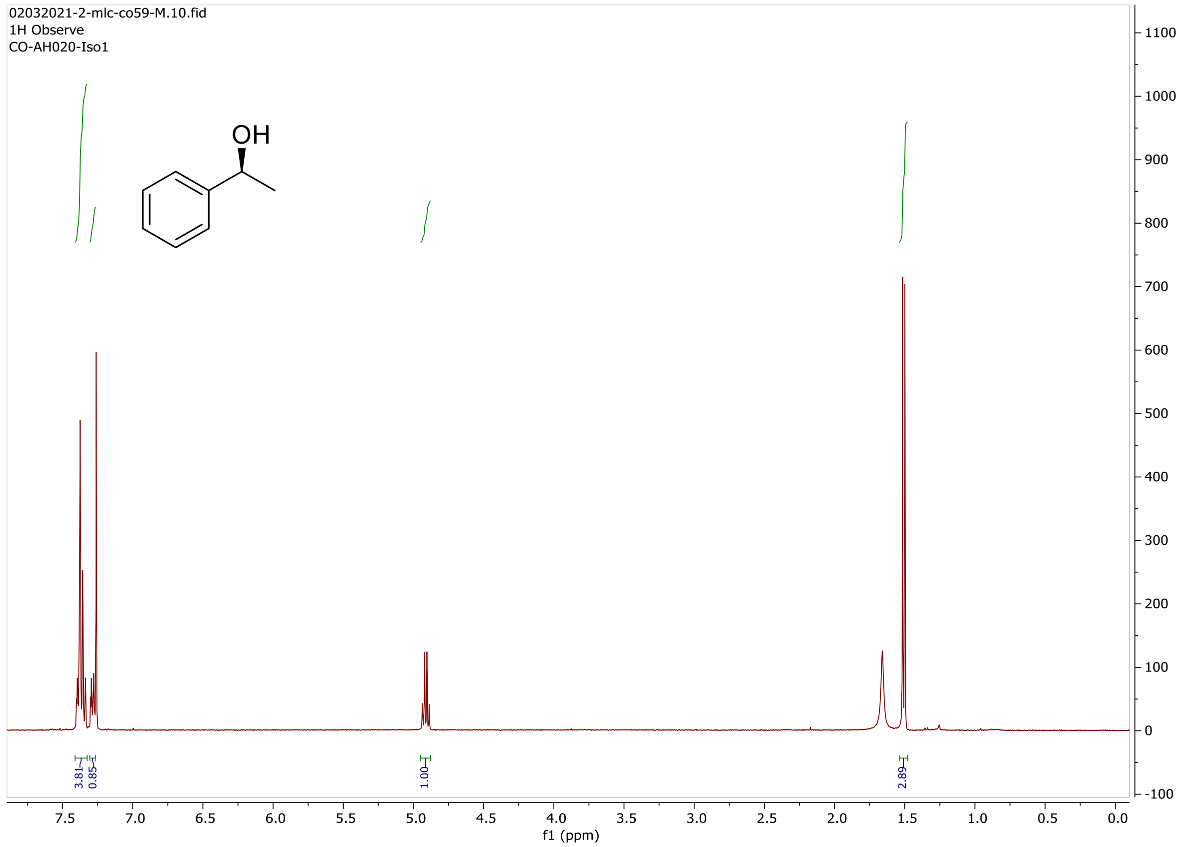

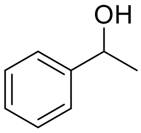

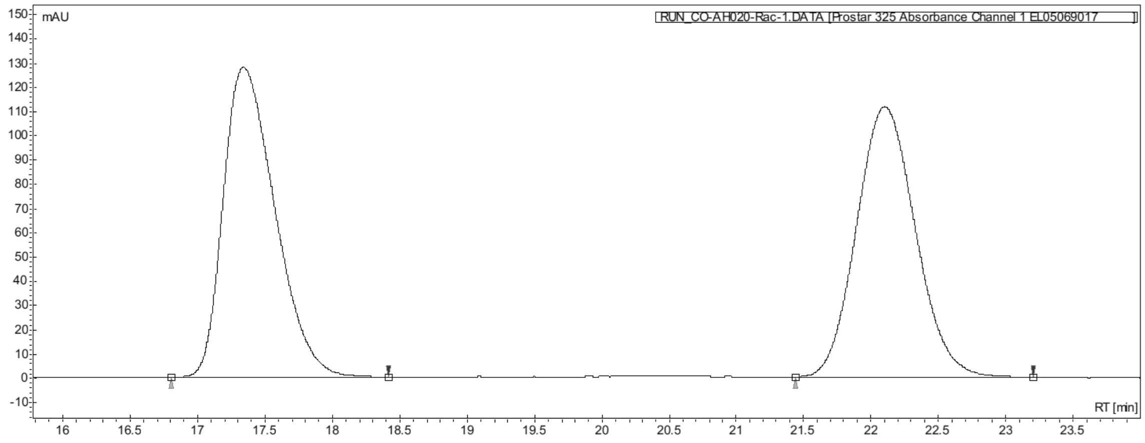

#	Name	Time [Min]	Quantity [% Area	Height [mAU]	Area [mAU.Min]	Area % [%]
1	UNKNOWN	17.34	50.17	127.9	56.9	50.170
2	UNKNOWN	22.10	49.83	111.6	56.5	49.830
Total			100.00	239.5	113.3	100.000

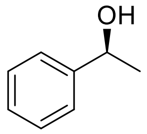

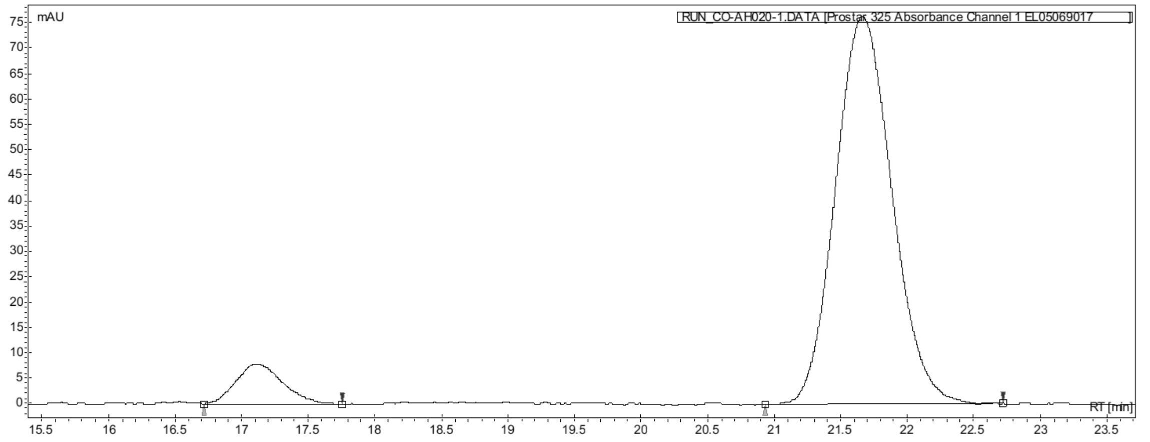

	#	Name	Time [Min]	Quantity [% Are	Height [mAU]	Area [mAU.Min]	Area % [%]
▶	1	UNKNOWN	17.10	8.00	7.9	3.3	8.002
	2	UNKNOWN	21.66	92.00	76.2	37.6	91.998
	Total			100.00	84.1	40.8	100.000

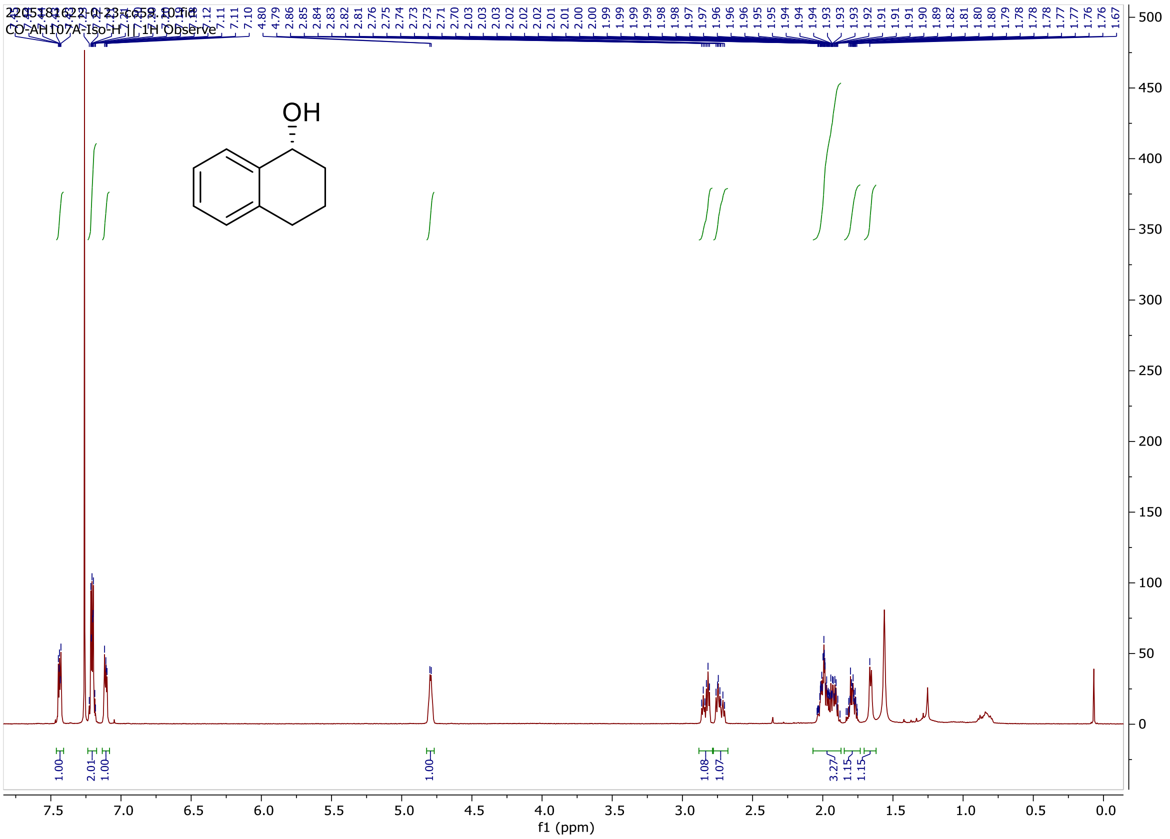

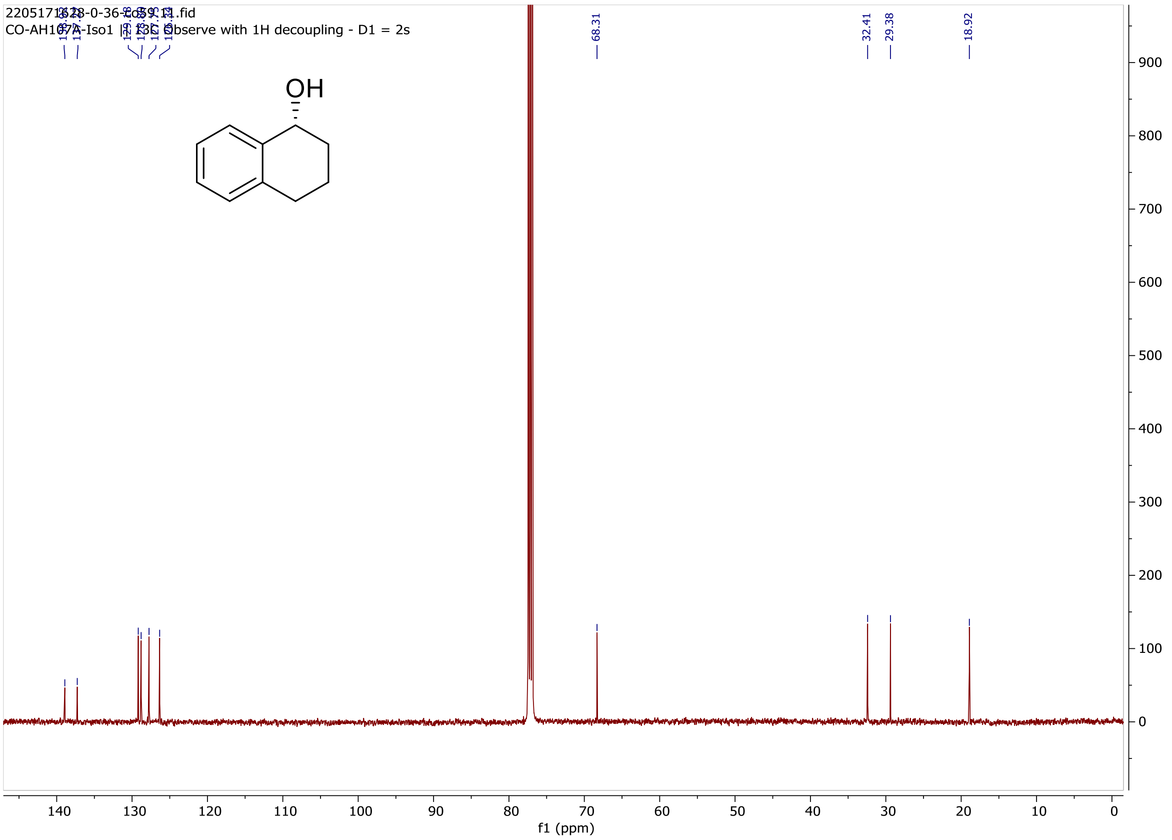

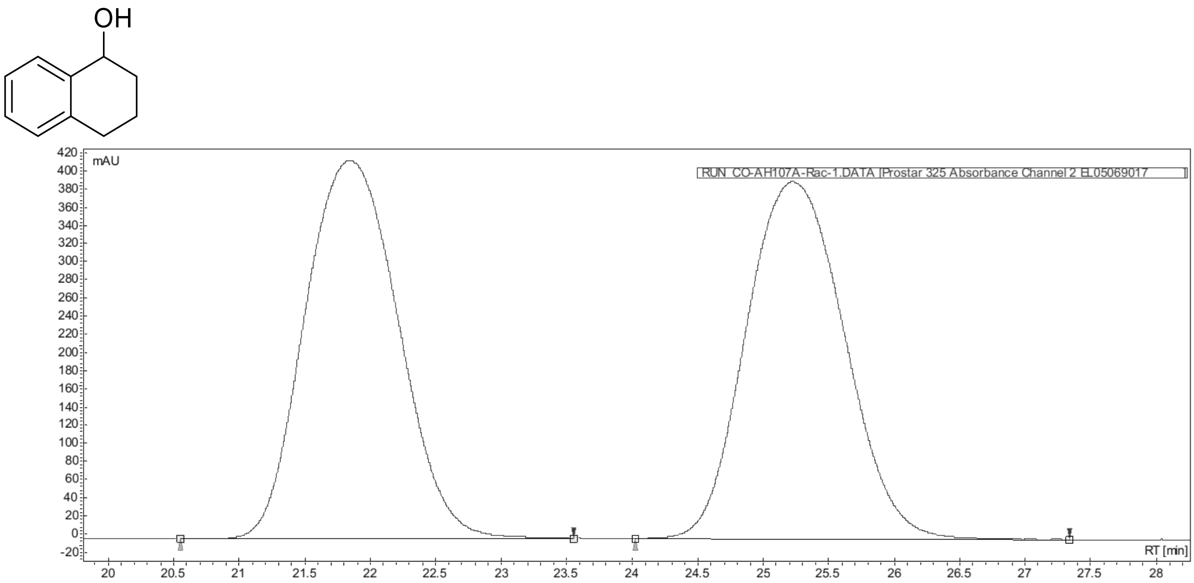

#	Name	Time [Min]	Quantity [% Area]	Height [mAU]	Area [mAU.Min]	Area % [%]
1	UNKNOWN	21.84	50.18	416.7	344.1	50.181
2	UNKNOWN	25.22	49.82	393.2	341.6	49.819
Total			100.00	809.9	685.6	100.000

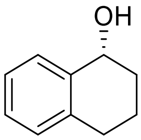

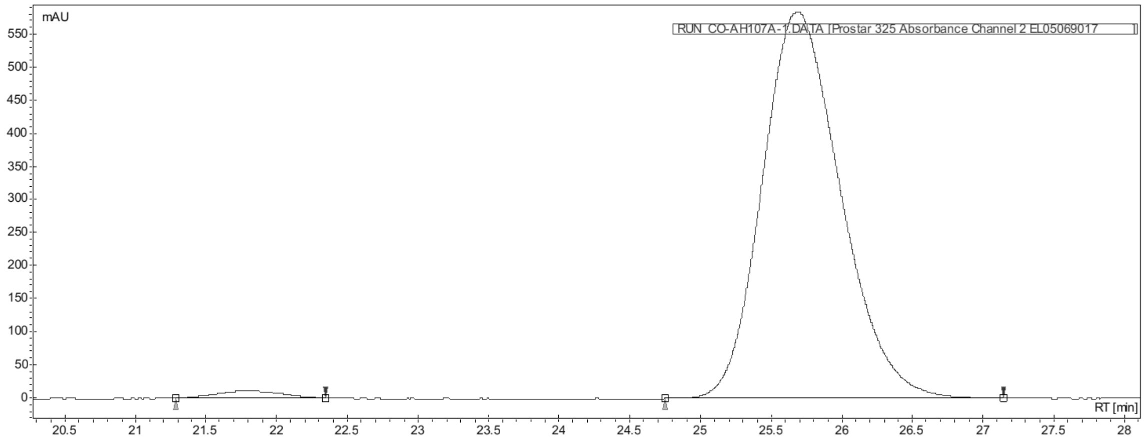

#	Name	Time [Min]	Quantity [% Area	Height [mAU]	Area [mAU.Min]	Area % [%]
1	UNKNOWN	21.79	1.50	10.6	5.7	1.496
2	UNKNOWN	25.69	98.50	585.1	375.3	98.504
Total			100.00	595.6	381.0	100.000

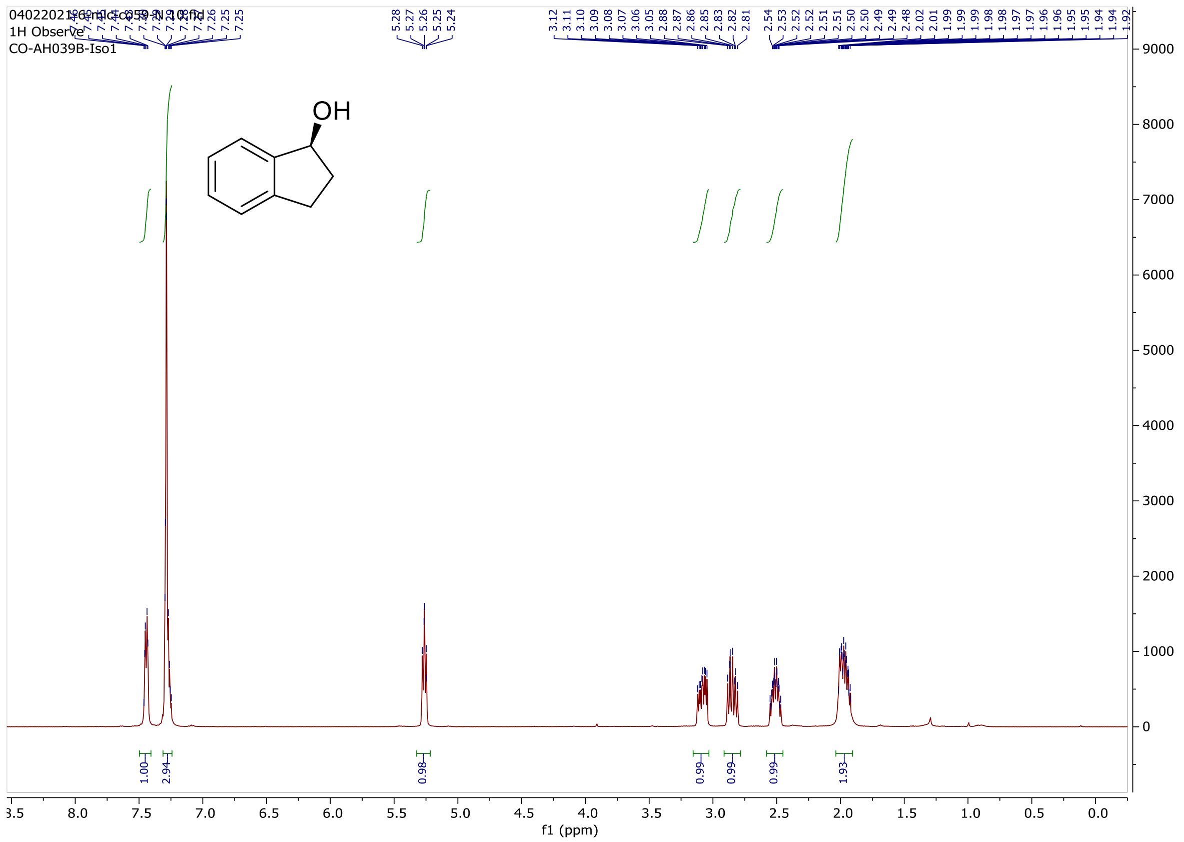
1H Observe
CO-AH039B-Iso1
OH
f1 (ppm)

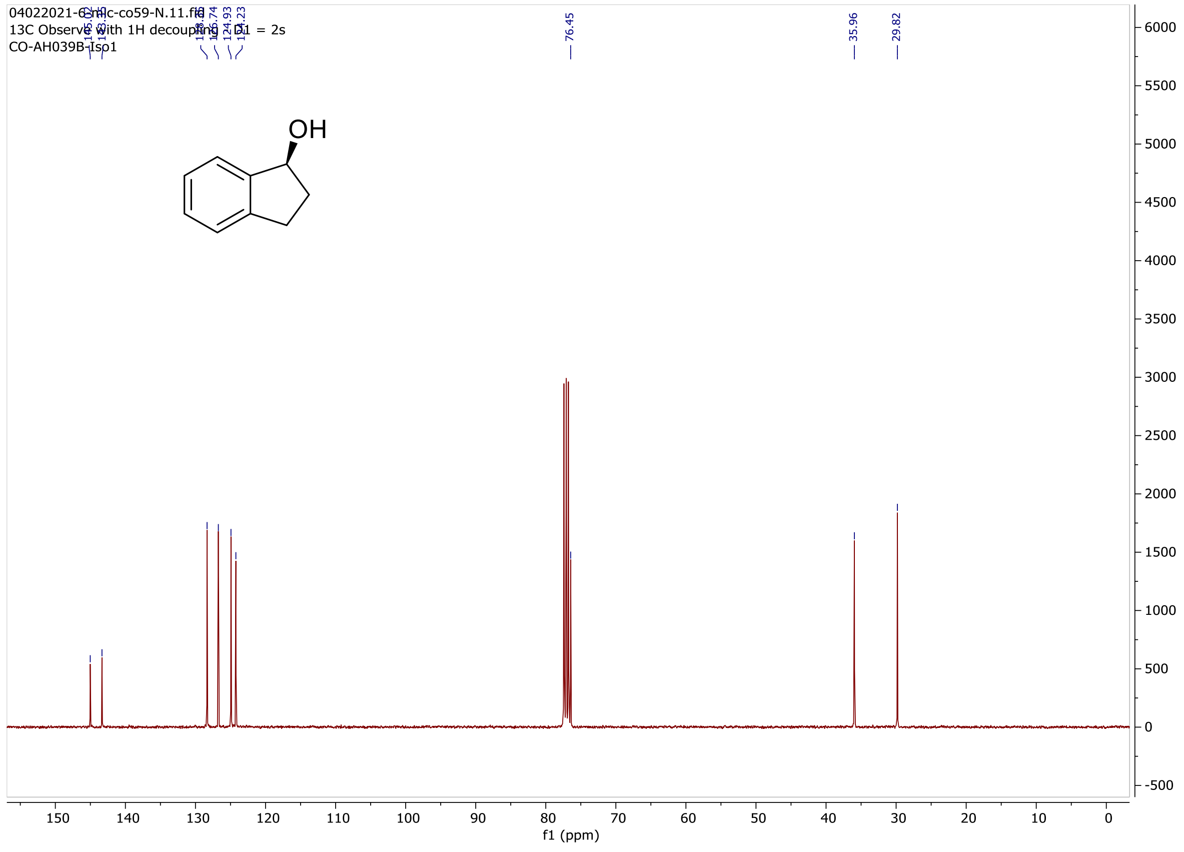
13C Observe with 1H decoupling
CO-AH039B-Iso1
OH
76.45
35.96
29.82
f1 (ppm)

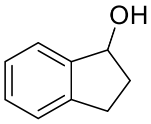

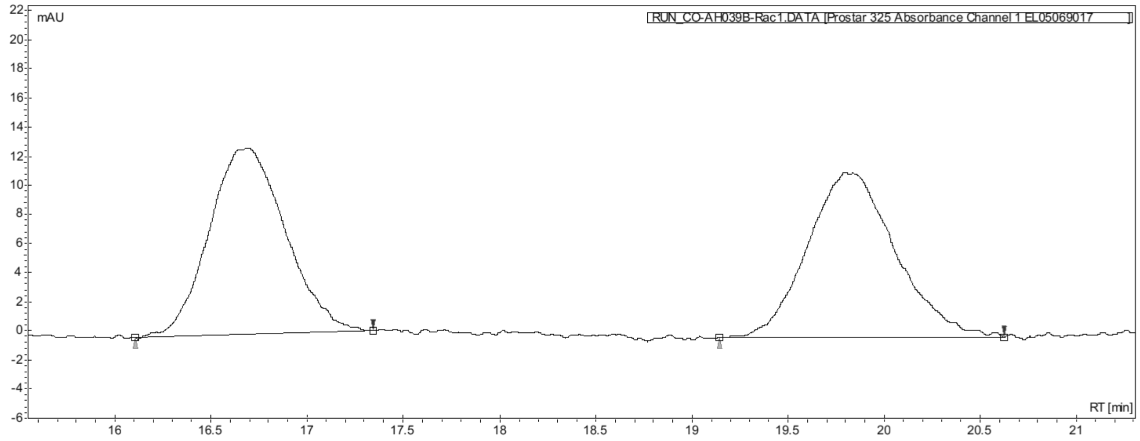

	#	Name	Time [Min]	Quantity [% Are	Height [mAU]	Area [mAU.Min]	Area % [%]
▶	1	UNKNOWN	16.69	49.50	12.8	5.9	49.500
	2	UNKNOWN	19.80	50.50	11.3	6.0	50.500
	Total			100.00	24.1	11.9	100.000

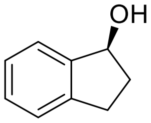

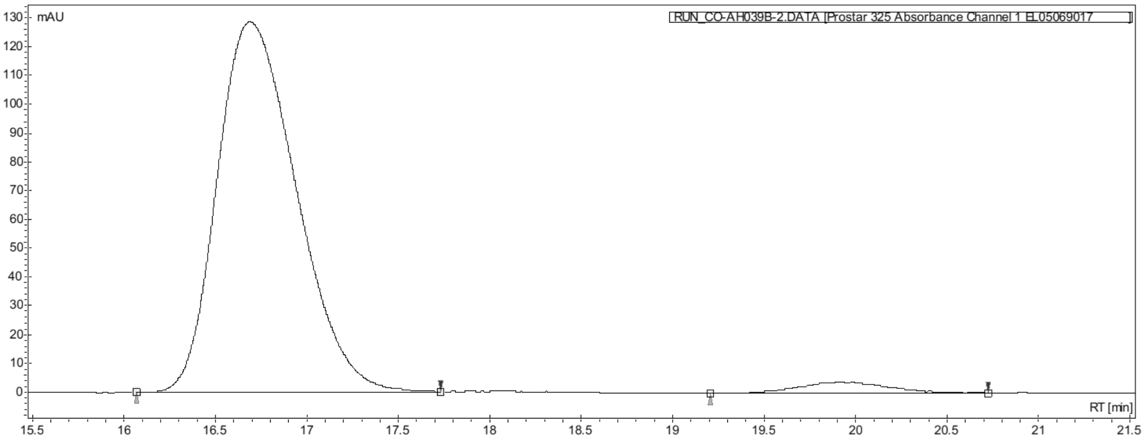

	#	Name	Time [Min]	Quantity [% Are	Height [mAU]	Area [mAU.Min]	Area % [%]
▶	1	UNKNOWN	16.69	96.75	128.7	64.4	96.749
	2	UNKNOWN	19.92	3.25	3.8	2.2	3.251
	Total			100.00	132.5	66.6	100.000

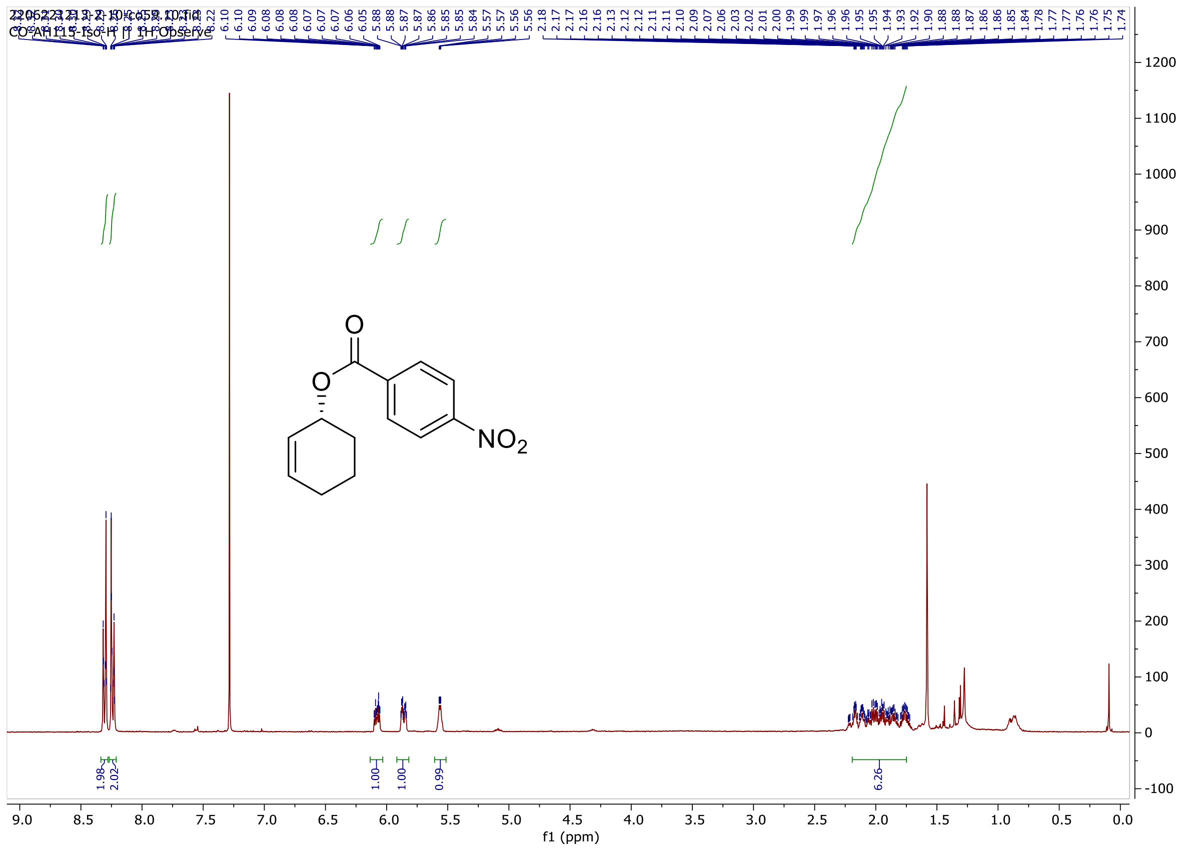
CO-AH115-Iso-H || 1H Observe
f1 (ppm)
NO2

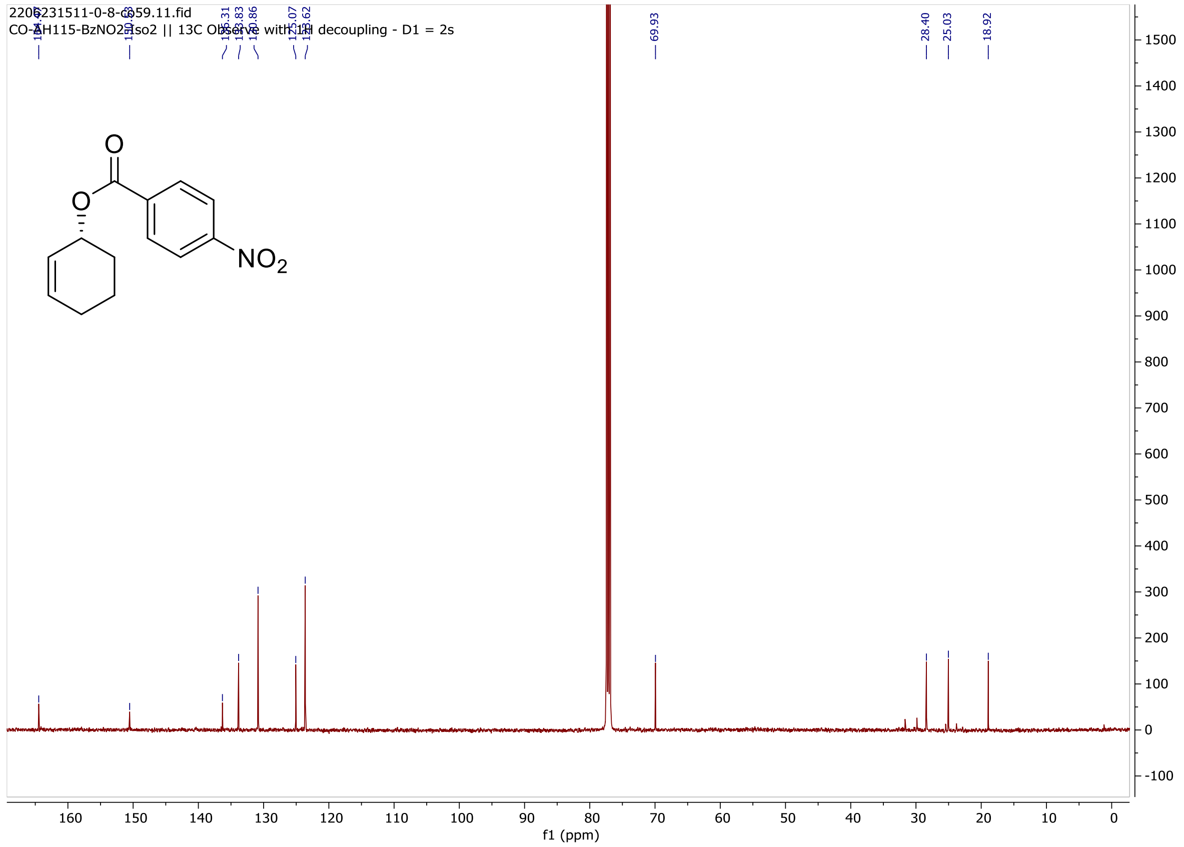
2206231511-0-8-co59.11.fid
CO-AH115-BzNO2-Iso2 || 13C Observe with 1H decoupling - D1 = 2s
f1 (ppm)
NO2

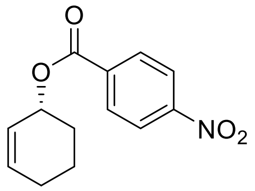

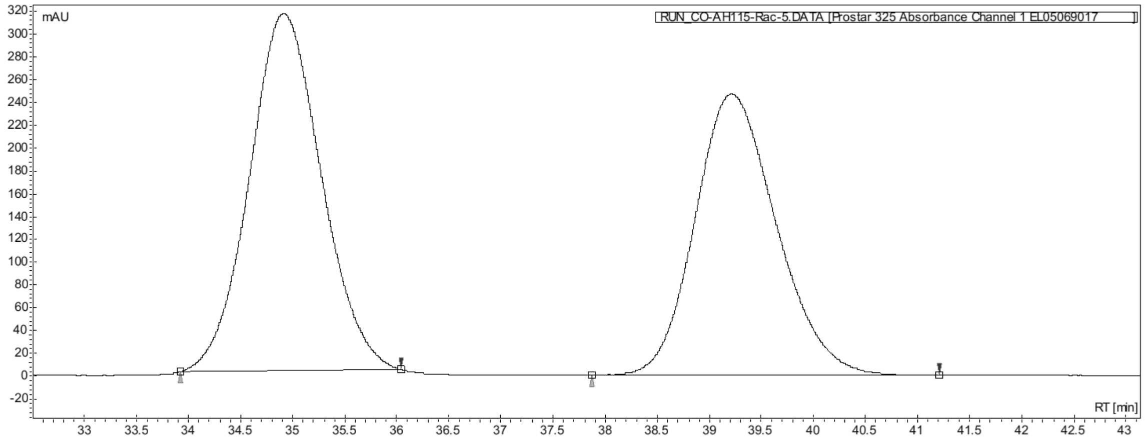

#	Name	Time [Min]	Quantity [% Area	Height [mAU]	Area [mAU.Min]	Area % [%]
1	UNKNOWN	34.91	52.98	313.3	250.8	52.980
2	UNKNOWN	39.21	47.02	246.6	222.6	47.020
Total			100.00	559.9	473.4	100.000

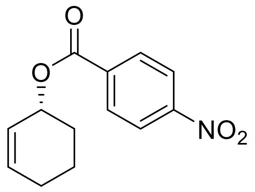

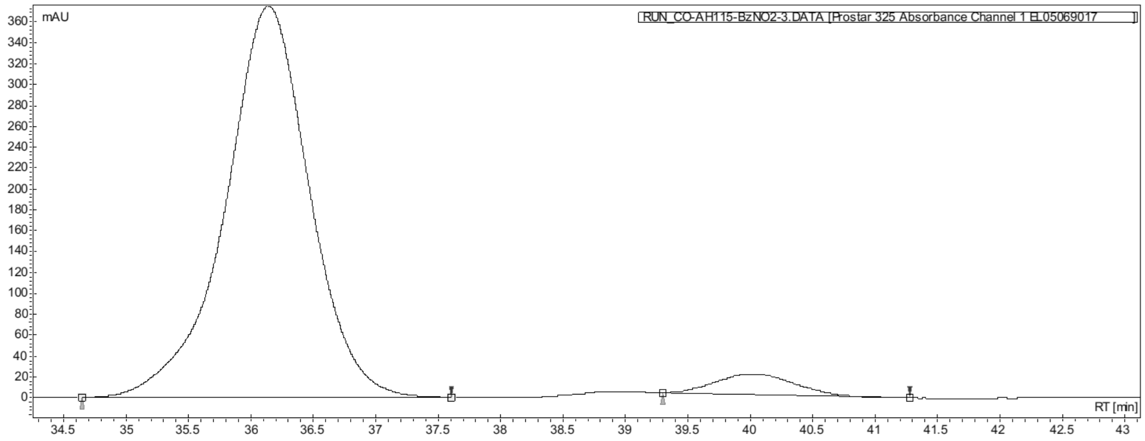

#	Name	Time [Min]	Quantity [% Area	Height [mAU]	Area [mAU.Min]	Area % [%]
1	UNKNOWN	36.14	95.30	375.0	291.8	95.296
2	UNKNOWN	40.02	4.70	19.6	14.4	4.704
Total			100.00	394.6	306.2	100.000

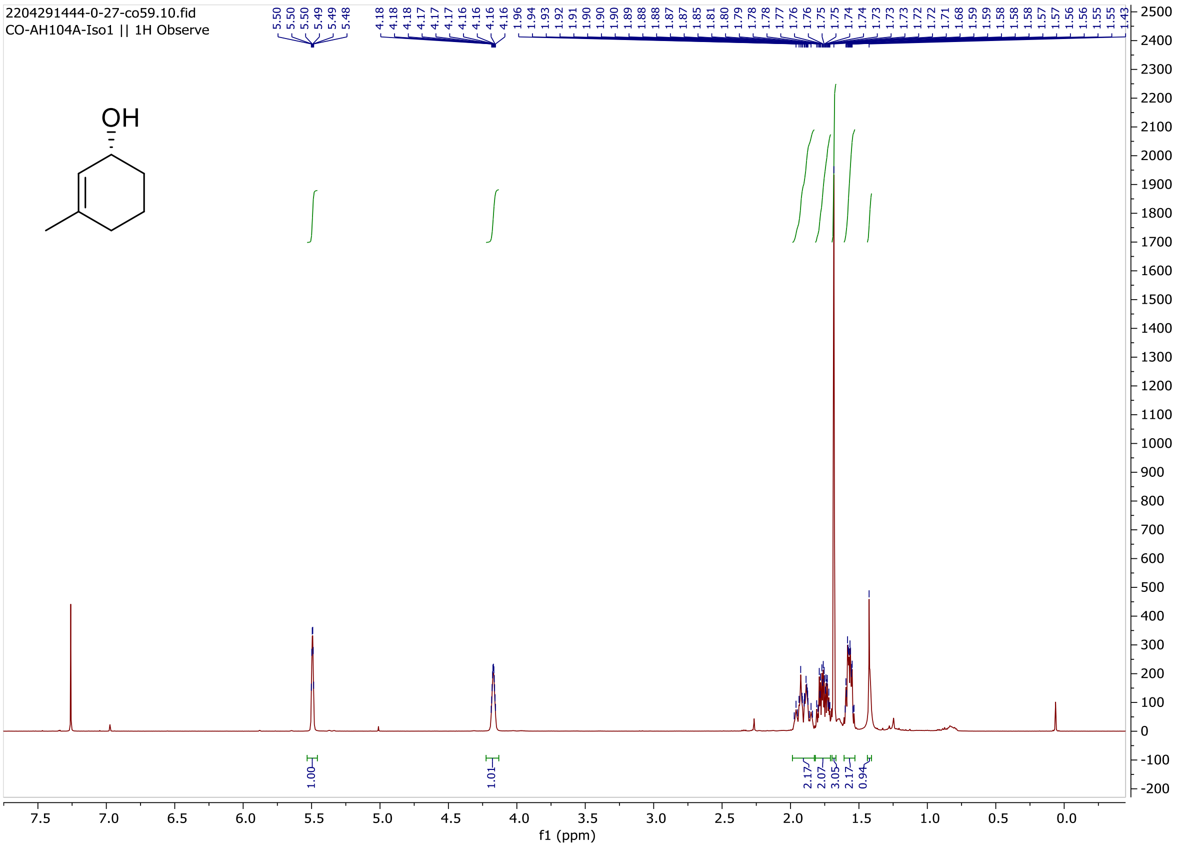
2204291444-0-27-co59.10.fid
CO-AH104A-Iso1 || 1H Observe
OH
f1 (ppm)

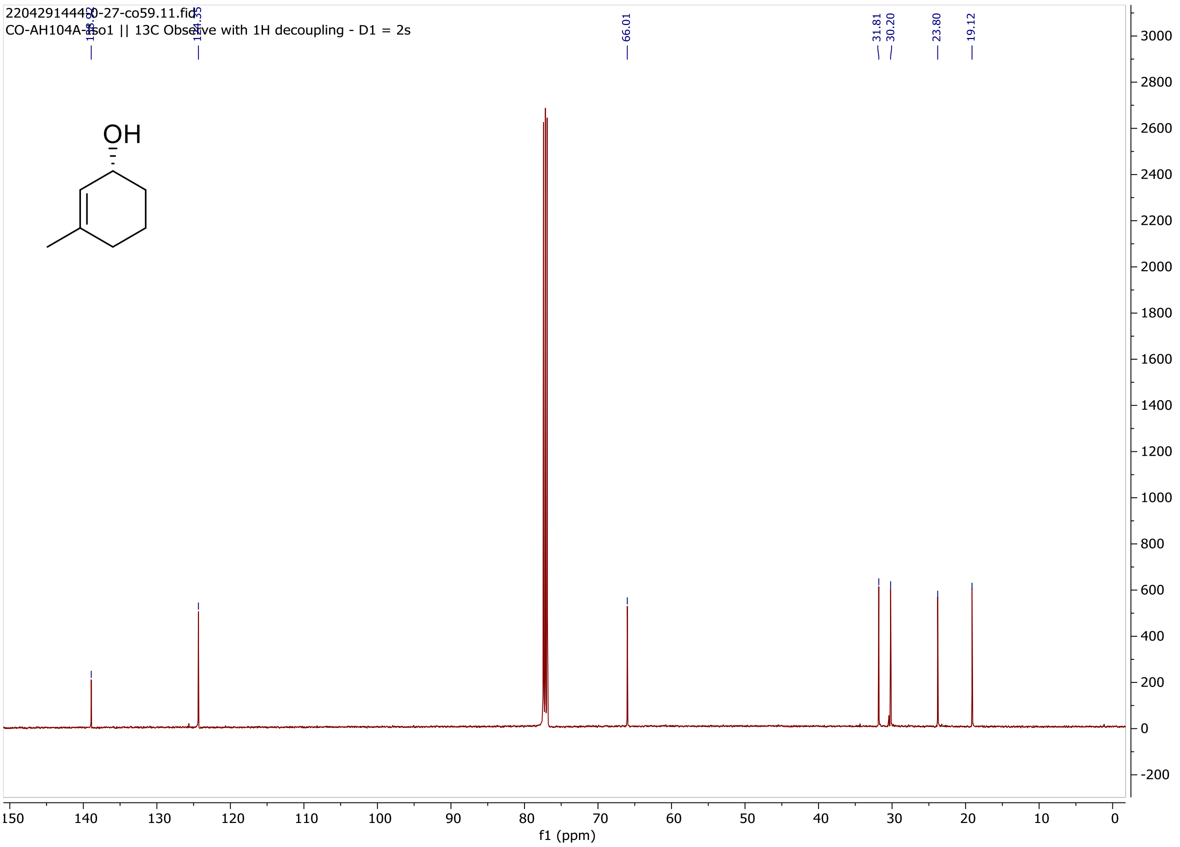
2204291444-0-27-co59.11.fid
CO-AH104A-Iso1 || 13C Observe with 1H decoupling - D1 = 2s
66.01
31.81
30.20
23.80
19.12
OH
f1 (ppm)

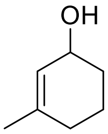

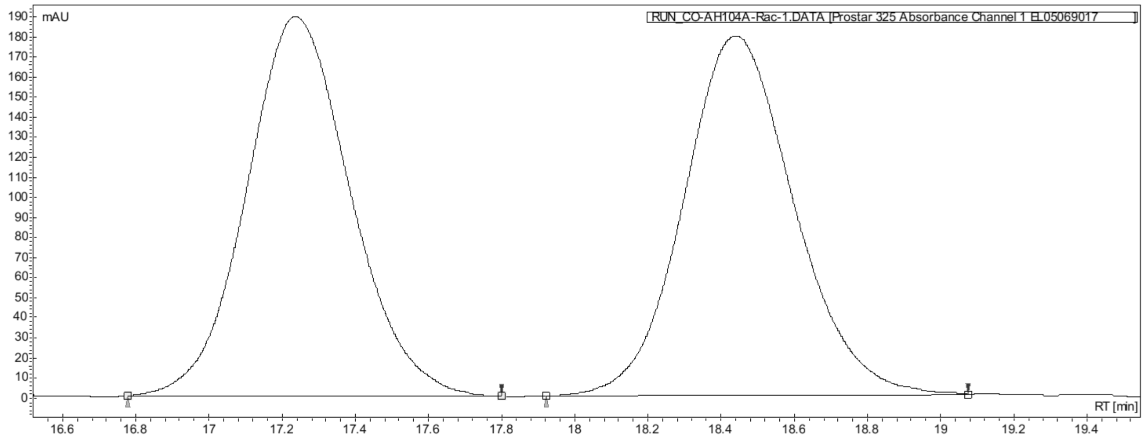

#	Name	Time [Min]	Quantity [% Are:	Height [mAU]	Area [mAU.Min]	Area % [%]
1	UNKNOWN	17.23	49.91	189.3	62.3	49.907
2	UNKNOWN	18.44	50.09	179.2	62.6	50.093
Total			100.00	368.6	124.9	100.000

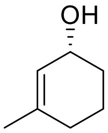

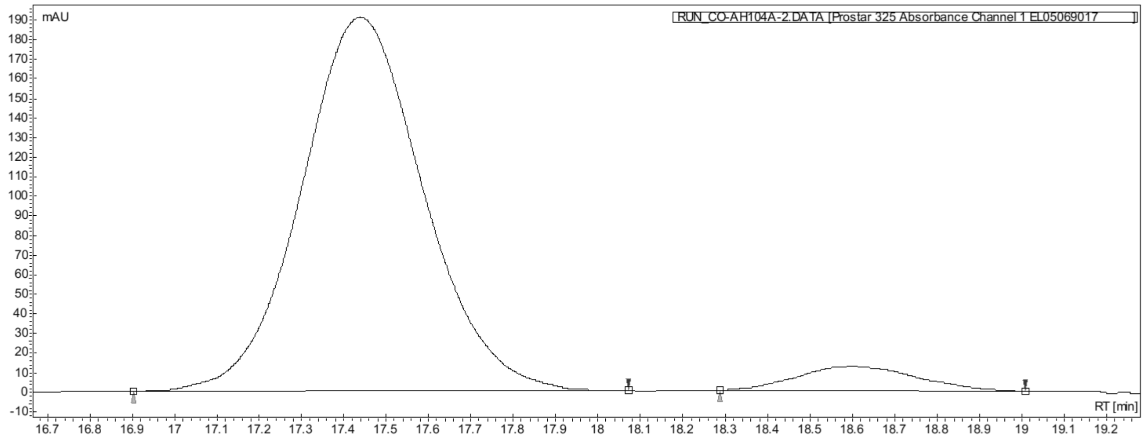

#	Name	Time [Min]	Quantity [% Are:	Height [mAU]	Area [mAU.Min]	Area % [%]
1	UNKNOWN	17.44	94.16	190.9	63.9	94.161
2	UNKNOWN	18.60	5.84	12.1	4.0	5.839
Total			100.00	203.0	67.9	100.000

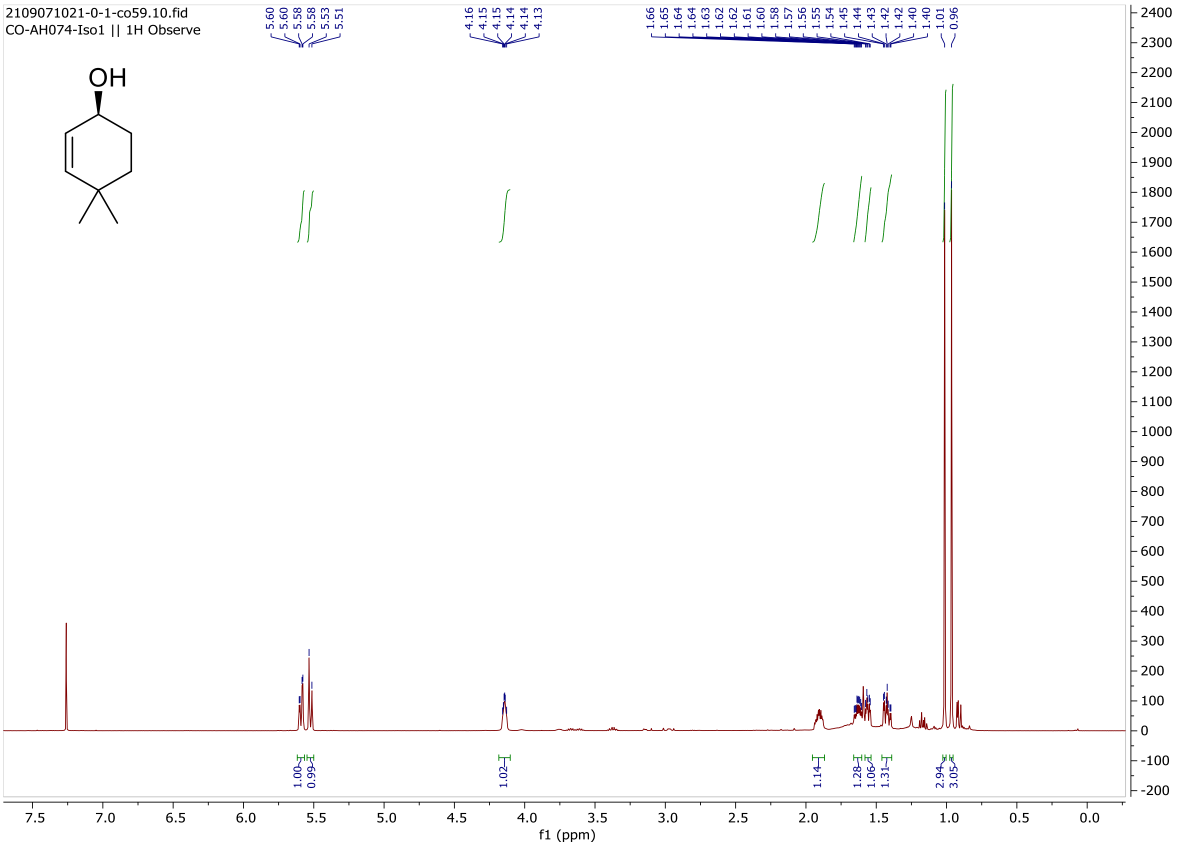
2109071021-0-1-co59.10.fid
CO-AH074-Iso1 || 1H Observe
OH
f1 (ppm)

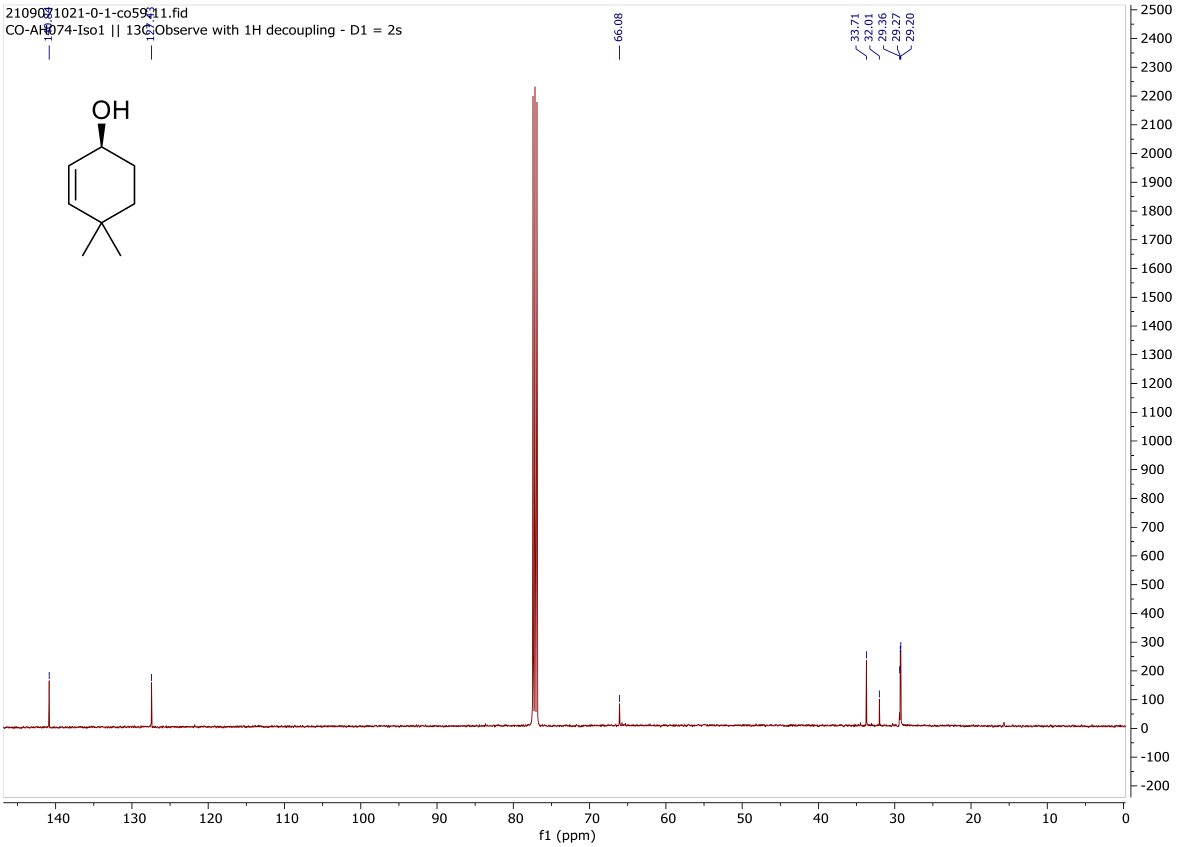
CO-AH074-Iso1 || 13C Observe with 1H decoupling - D1 = 2s
OH
66.08
33.71
32.01
29.36
29.27
29.20
f1 (ppm)

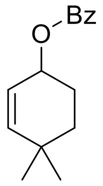

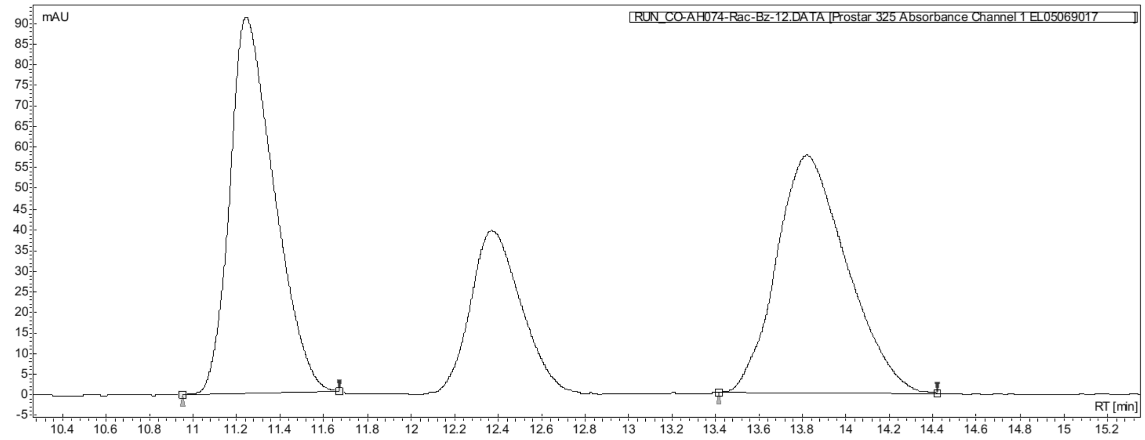

Signal at ~12.4 min trace quantity of Ethyl benzoate produced as a side product in the formation of desired benzoate ester.

	#	Name	Time [Min]	Quantity [% Area]	Height [mAU]	Area [mAU.Min]	Area % [%]
▶	1	UNKNOWN	11.24	50.04	91.3	21.6	50.040
	2	UNKNOWN	13.82	49.96	57.6	21.6	49.960
	Total			100.00	148.9	43.2	100.000

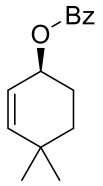

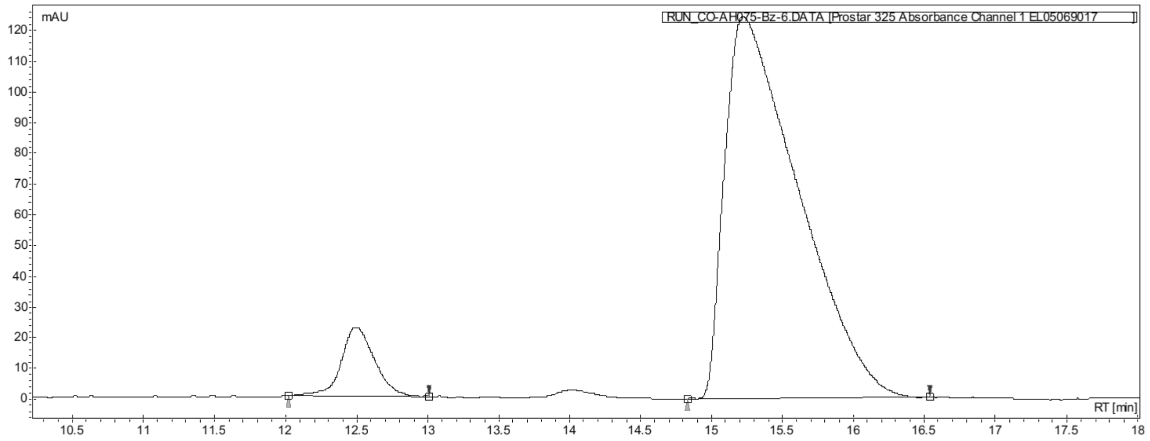

	#	Name	Time [Min]	Quantity [% Area]	Height [mAU]	Area [mAU.Min]	Area % [%]
▶	1	UNKNOWN	12.49	7.40	22.2	6.0	7.404
	2	UNKNOWN	15.22	92.60	124.3	74.7	92.596
	Total			100.00	146.5	80.6	100.000

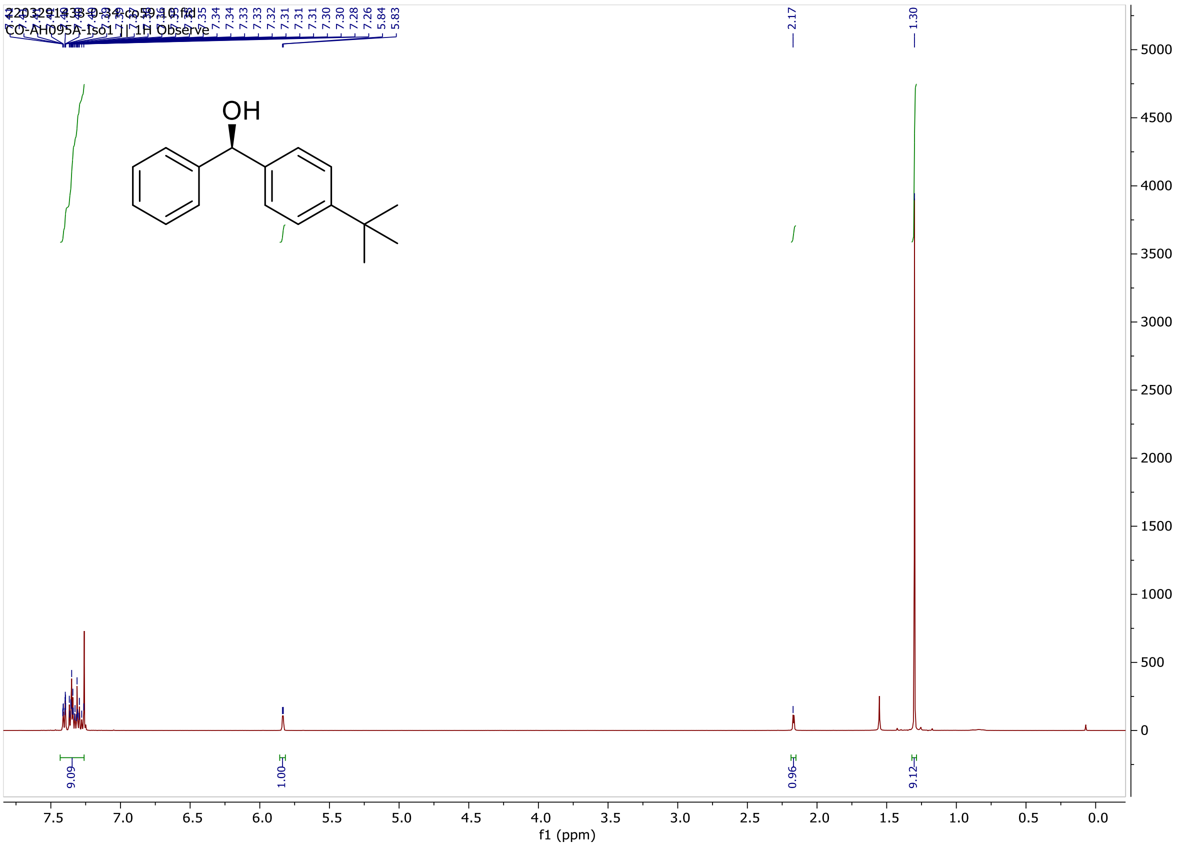
CO-AH095A-Iso1 || 1H Observe
OH
9.09
1.00
0.96
9.12
2.17
1.30
f1 (ppm)

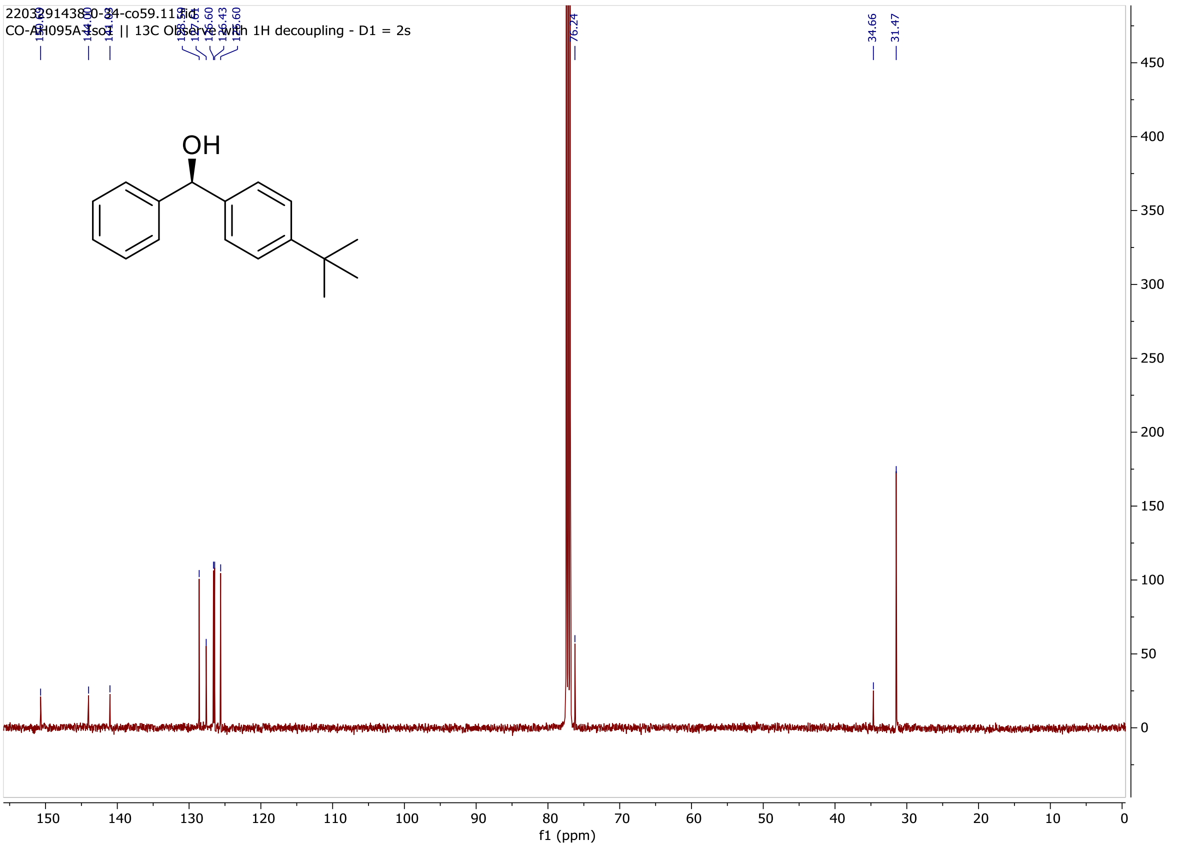
CO-AH095A-Iso1 || 13C Observe with 1H decoupling - D1 = 2s
OH
76.24
34.66
31.47
f1 (ppm)

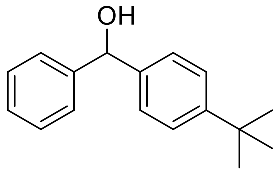

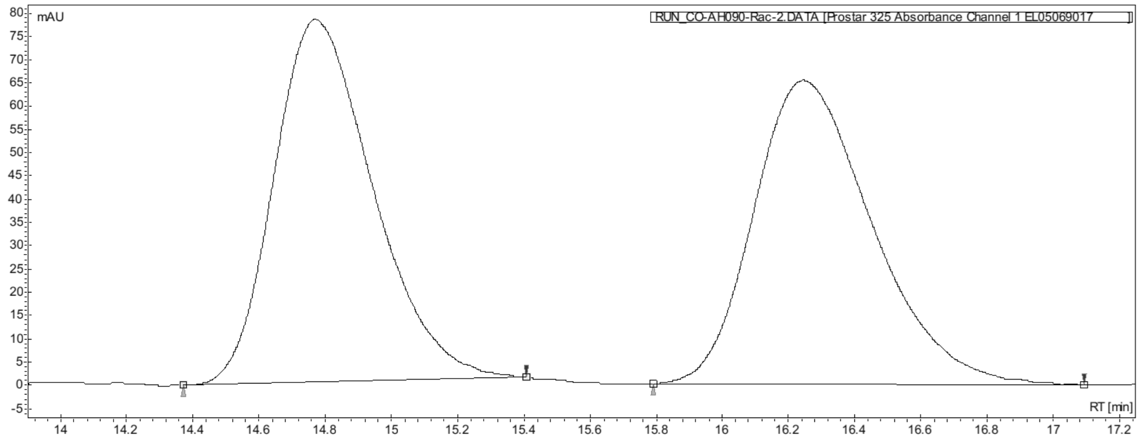

	#	Name	Time [Min]	Quantity [% Are	Height [mAU]	Area [mAU.Min]	Area % [%]
▶	1	UNKNOWN	14.77	49.92	78.1	27.0	49.916
	2	UNKNOWN	16.24	50.08	65.5	27.1	50.084
	Total			100.00	143.5	54.1	100.000

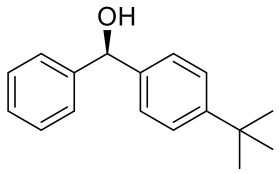

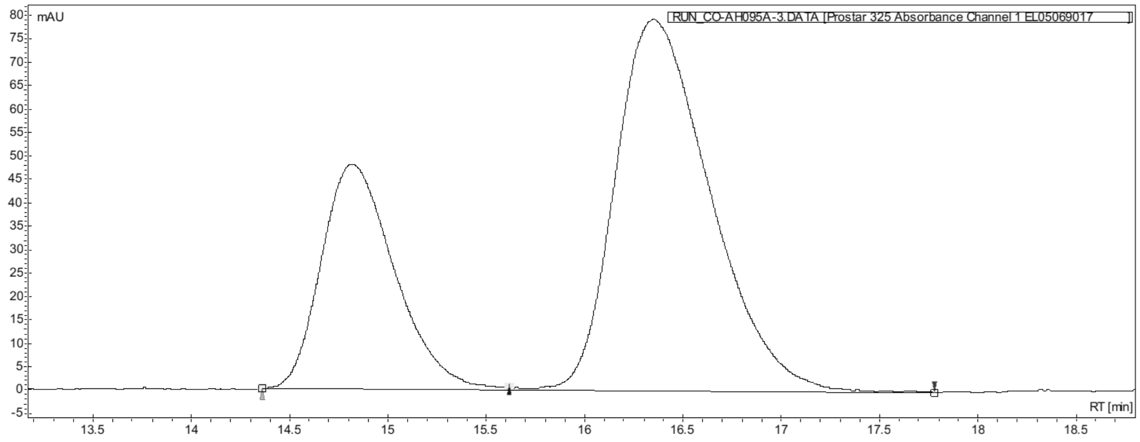

	#	Name	Time [Min]	Quantity [% Are	Height [mAU]	Area [mAU.Min]	Area % [%]
▶	1	UNKNOWN	14.82	31.87	48.1	20.8	31.875
	2	UNKNOWN	16.35	68.13	79.3	44.4	68.125
	Total			100.00	127.4	65.2	100.000

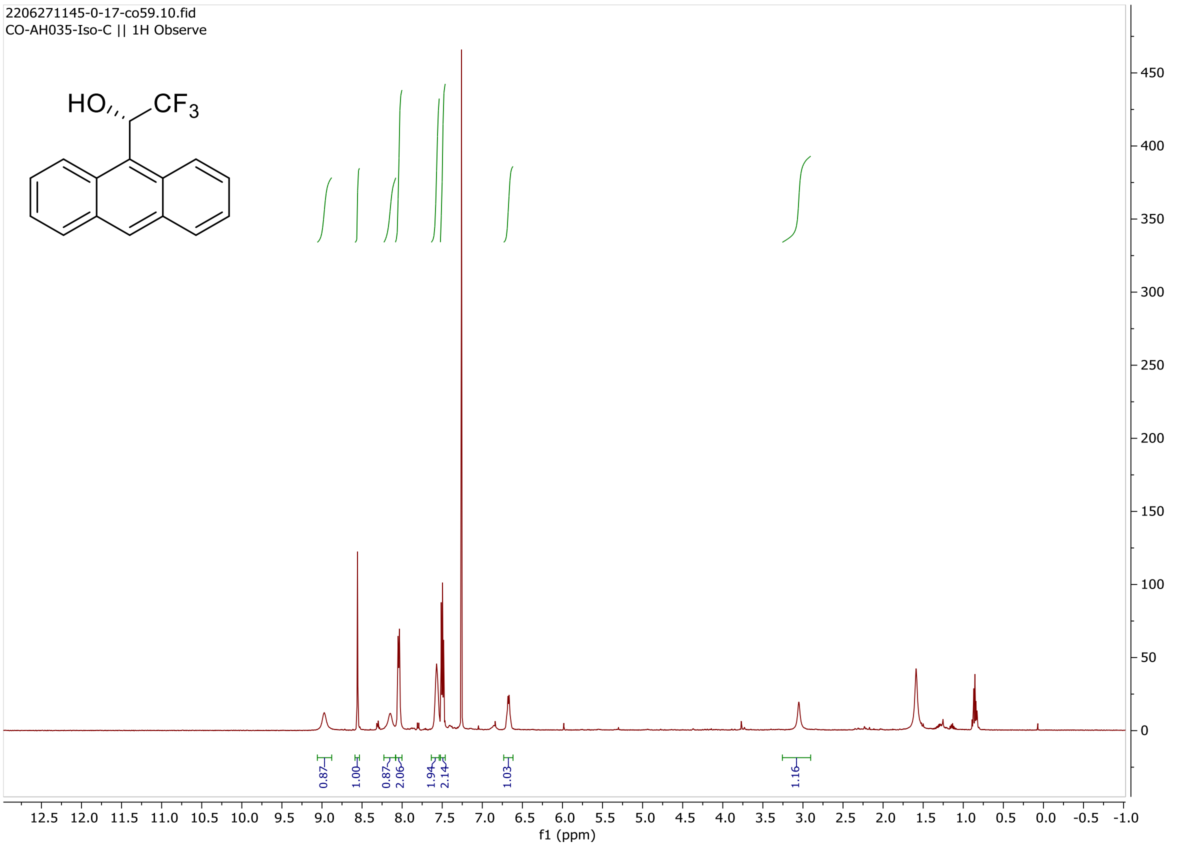
2206271145-0-17-co59.10.fid
CO-AH035-Iso-C || 1H Observe
HO
CF3
0.87
1.00
0.87
2.06
1.94
2.14
1.03
1.16
f1 (ppm)

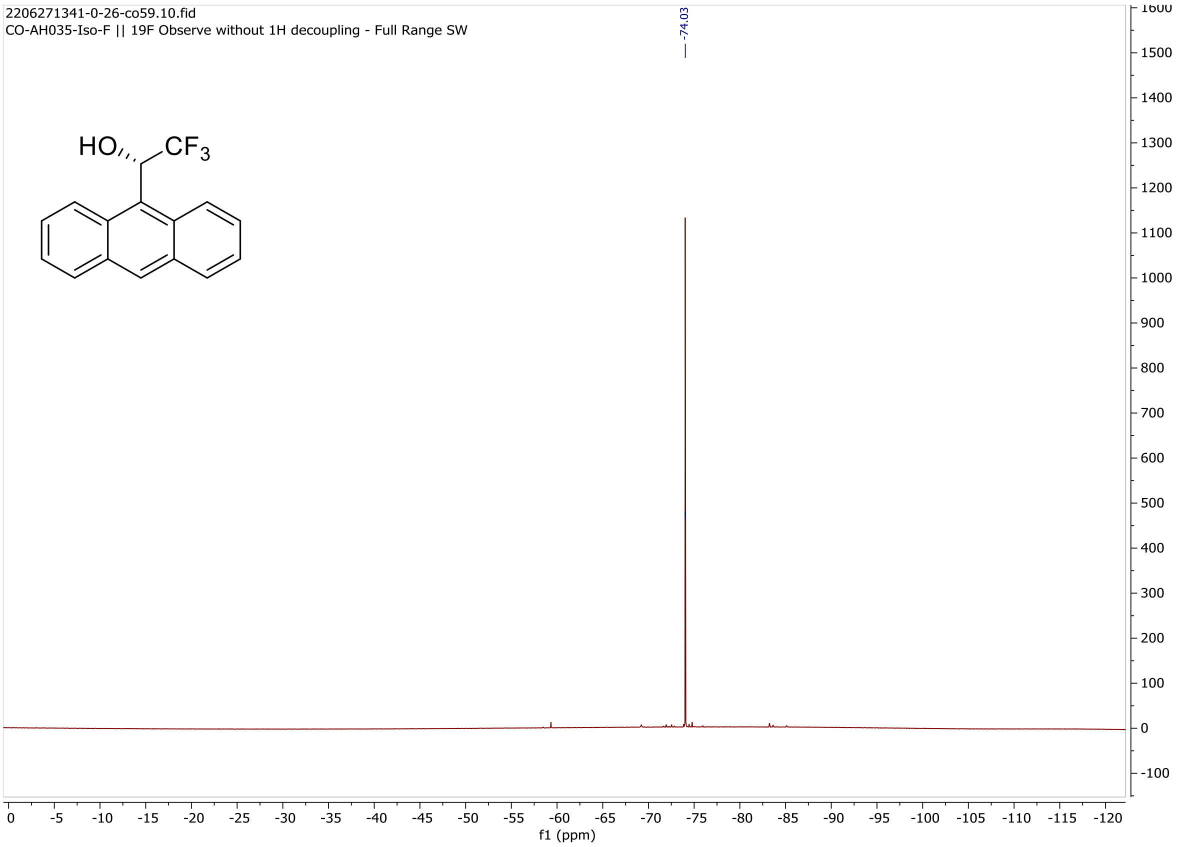
2206271341-0-26-co59.10.fid
CO-AH035-Iso-F || 19F Observe without 1H decoupling - Full Range SW
-74.03
HO
CF3
f1 (ppm)

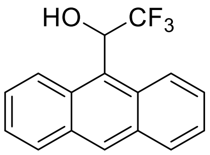

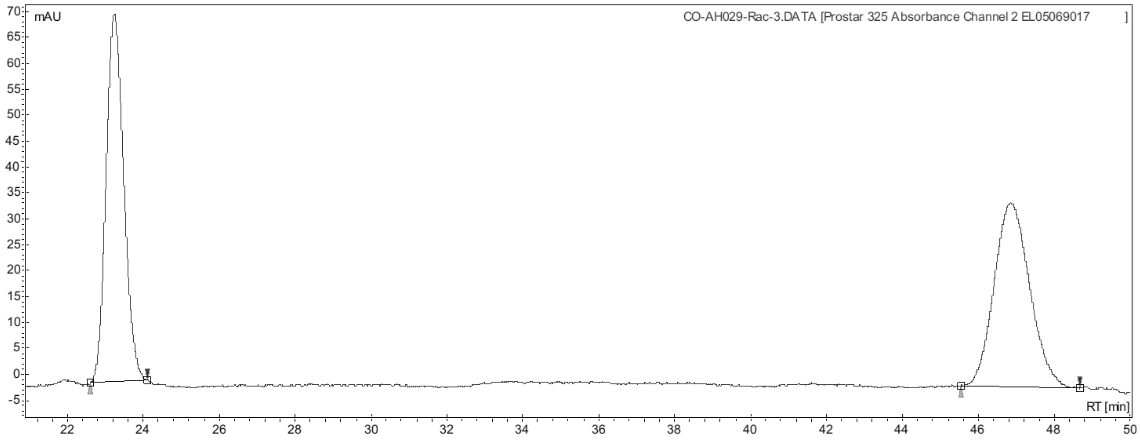

#	Name	Time [Min]	Quantity [% Area	Height [mAU]	Area [mAU.Min]	Area % [%]
1	UNKNOWN	23.25	49.75	71.0	38.3	49.749
2	UNKNOWN	46.85	50.25	35.5	38.7	50.251
Total			100.00	106.5	77.0	100.000

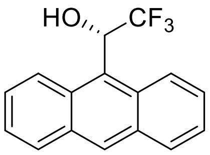

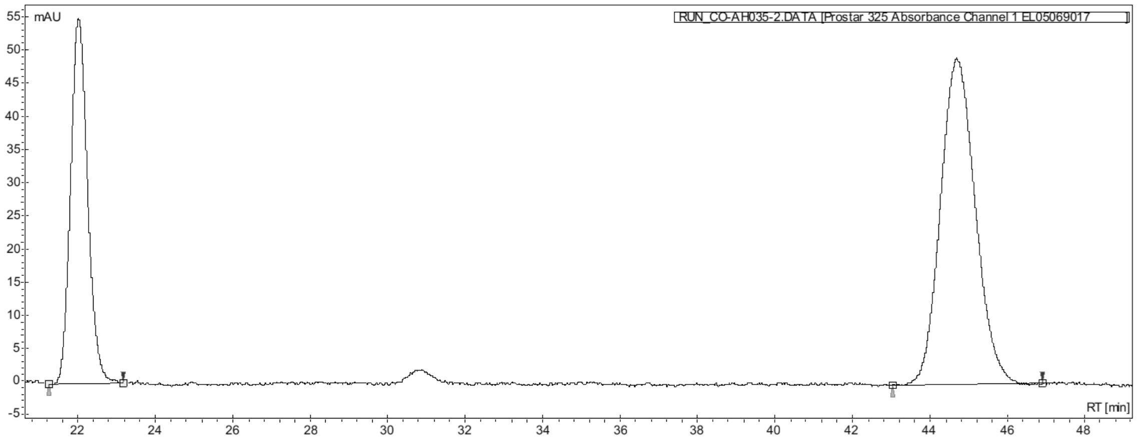

#	Name	Time [Min]	Quantity [% Area	Height [mAU]	Area [mAU.Min]	Area % [%]
1	UNKNOWN	22.02	35.59	55.2	28.2	35.587
2	UNKNOWN	44.68	64.41	49.2	51.0	64.413
Total			100.00	104.3	79.2	100.000

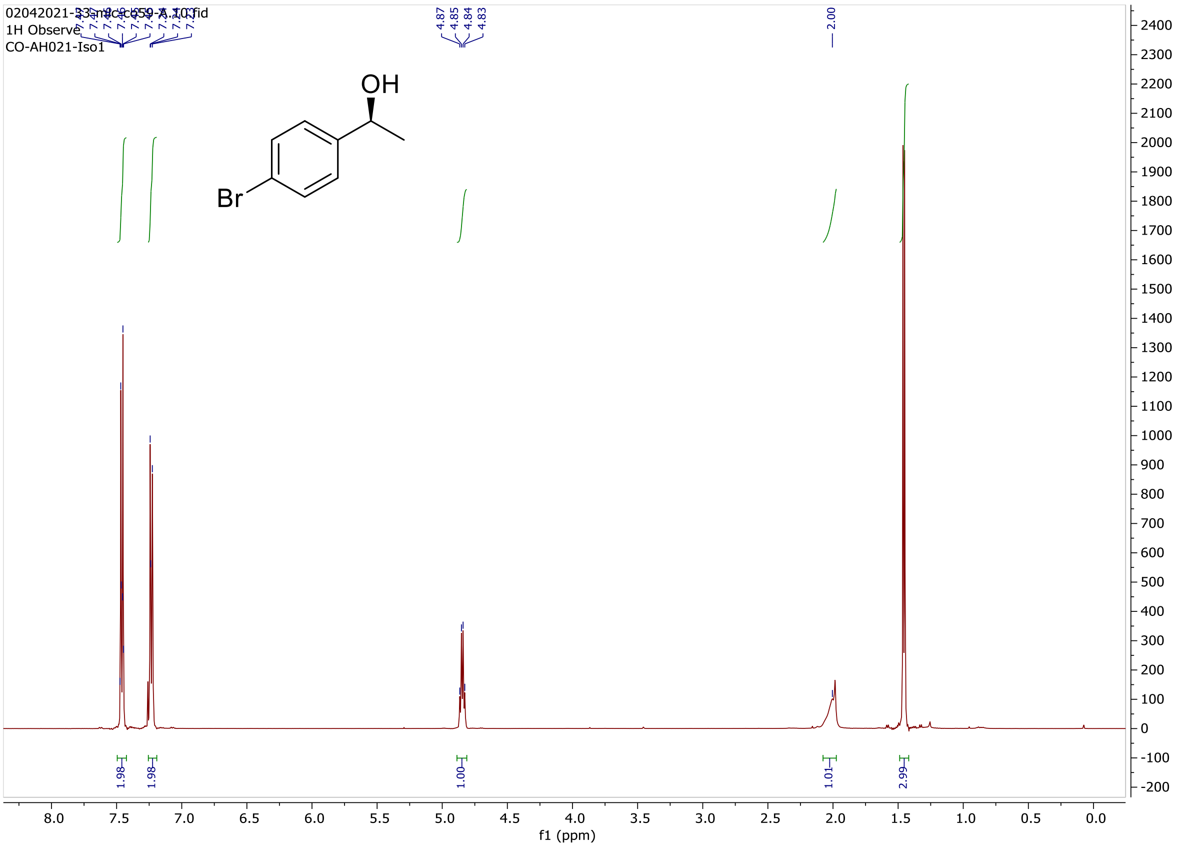
1H Observe
CO-AH021-Iso1
4.87
4.85
4.84
4.83
2.00
OH
Br
1.98
1.98
1.00
1.01
2.99
f1 (ppm)

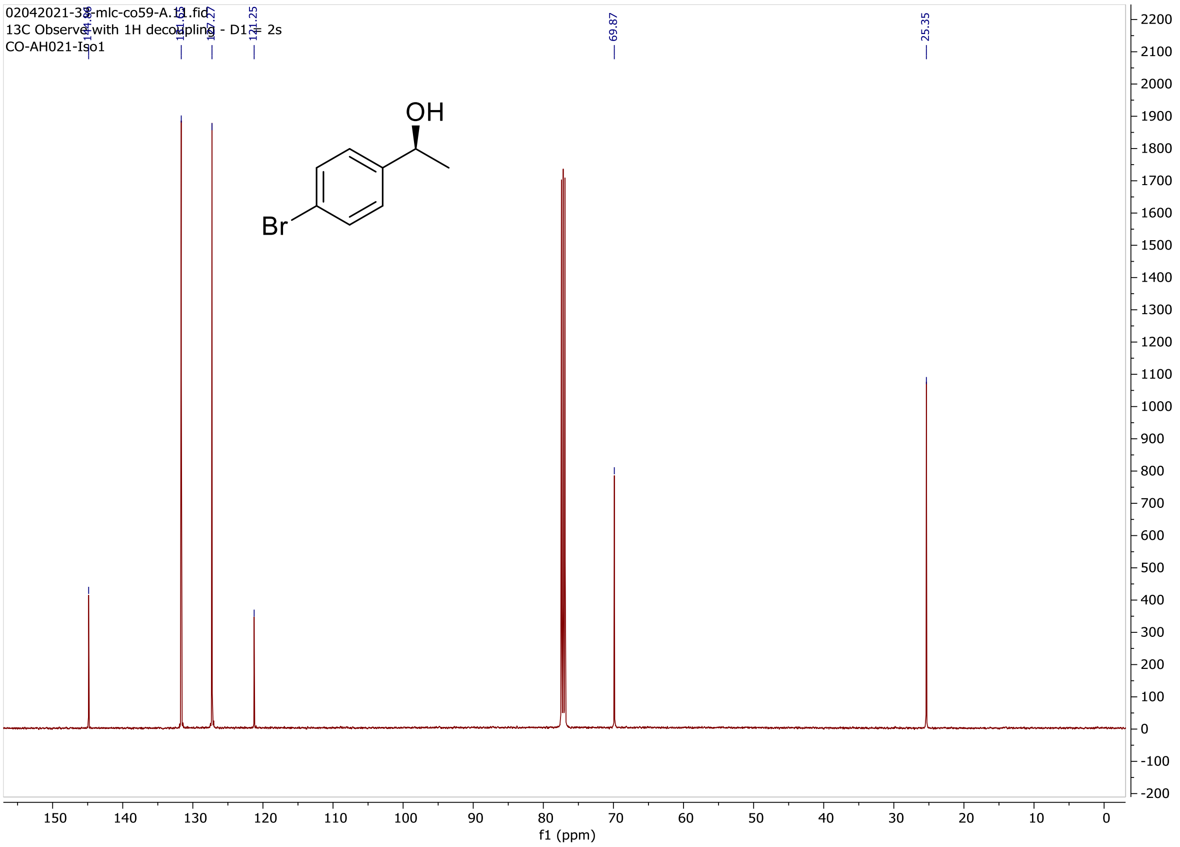
02042021-33-mlc-co59-A.11.fid
13C Observe with 1H decoupling - D1 = 2s
CO-AH021-Iso1
121.25
69.87
25.35
OH
Br
f1 (ppm)

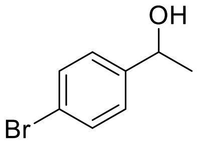

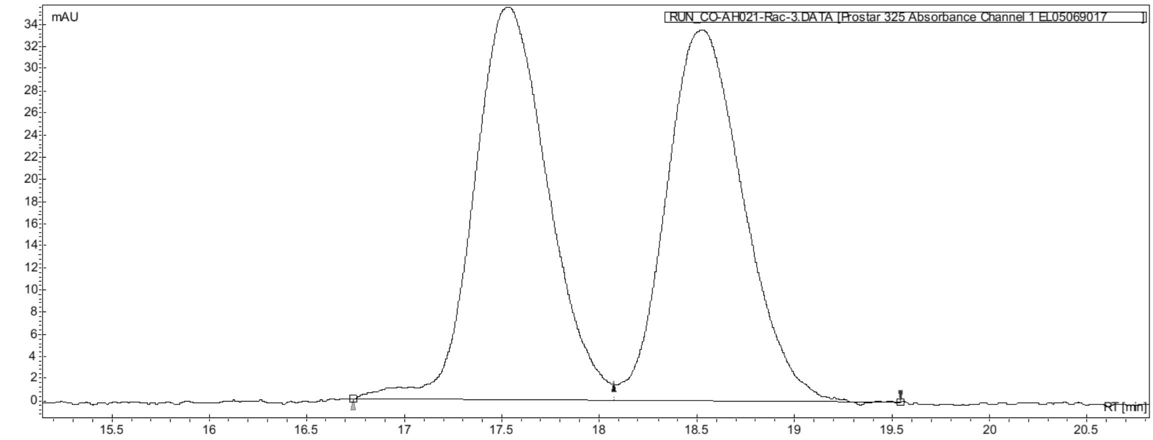

	#	Name	Time [Min]	Quantity [% Area	Height [mAU]	Area [mAU.Min]	Area % [%]
▶	1	UNKNOWN	17.53	50.31	35.5	15.6	50.305
	2	UNKNOWN	18.53	49.69	33.6	15.4	49.695
	Total			100.00	69.0	31.0	100.000

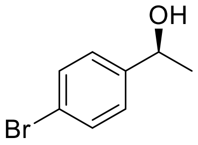

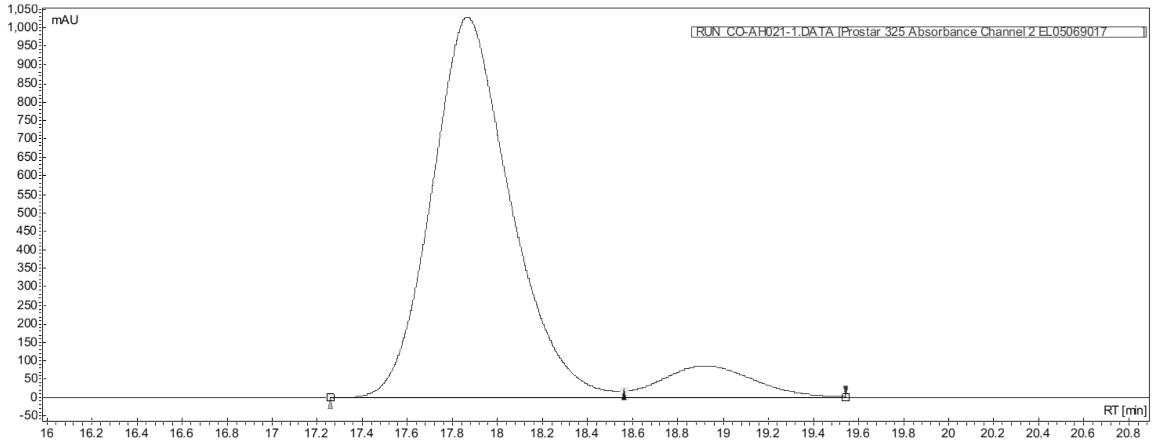

	#	Name	Time [Min]	Quantity [% Area	Height [mAU]	Area [mAU.Min]	Area % [%]
▶	1	UNKNOWN	17.87	91.33	1028.4	414.4	91.327
	2	UNKNOWN	18.92	8.67	84.0	39.4	8.673
	Total			100.00	1112.4	453.8	100.000

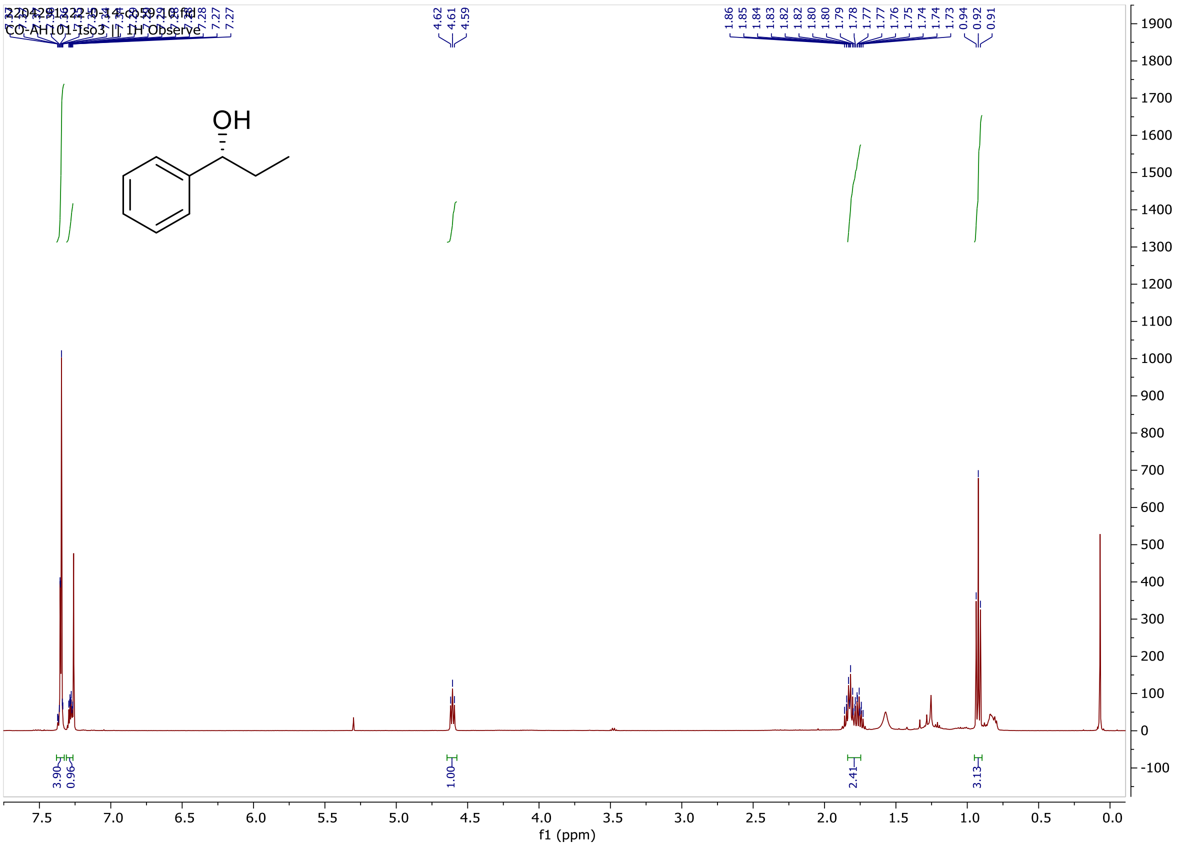

4.62
4.61
4.59
1.86
1.85
1.84
1.83
1.82
1.82
1.80
1.80
1.79
1.78
1.77
1.77
1.76
1.75
1.75
1.74
1.74
1.73
0.94
0.92
0.91
OH
3.90
0.96
1.00
2.41
3.13
f1 (ppm)

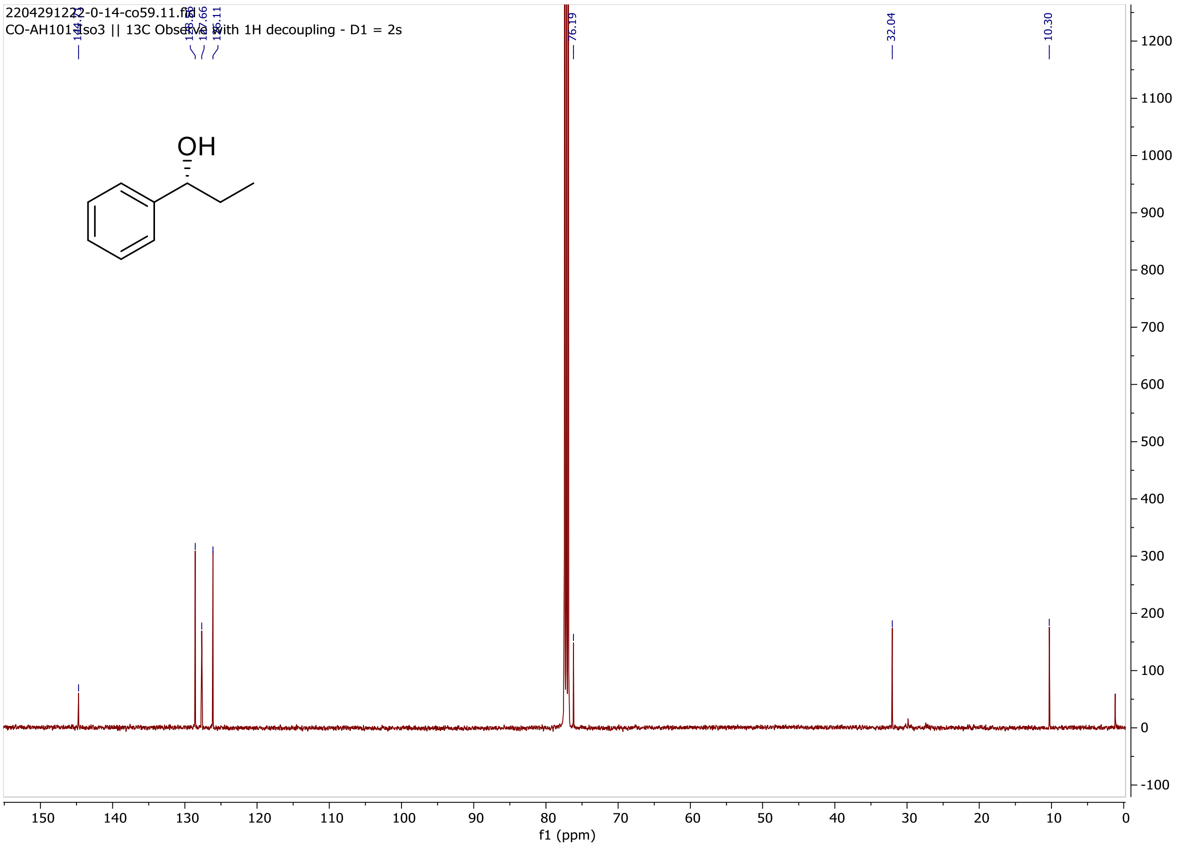

CO-AH101-Iso3 || 13C Observe with 1H decoupling - D1 = 2s
127.66
126.11
76.19
32.04
10.30
OH
f1 (ppm)

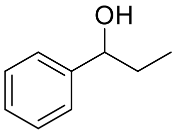

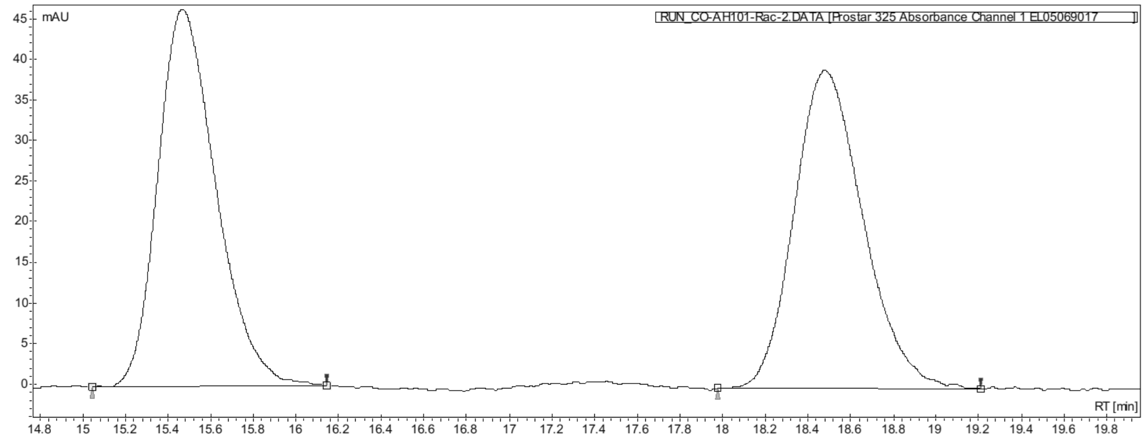

#	Name	Time [Min]	Quantity [% Area]	Height [mAU]	Area [mAU.Min]	Area % [%]
1	UNKNOWN	15.47	49.96	46.4	14.7	49.957
2	UNKNOWN	18.48	50.04	39.2	14.7	50.043
Total			100.00	85.6	29.4	100.000

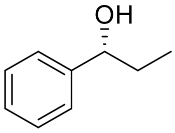

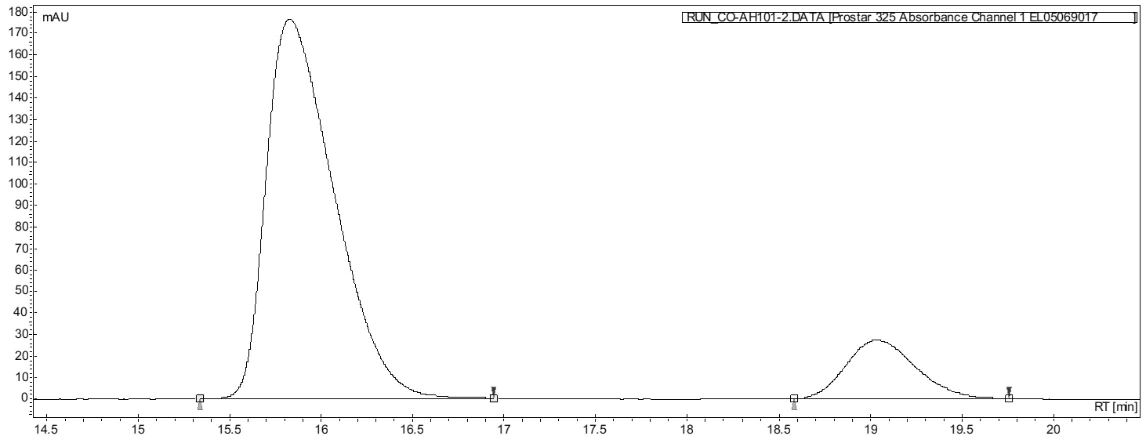

#	Name	Time [Min]	Quantity [% Area]	Height [mAU]	Area [mAU.Min]	Area % [%]
1	UNKNOWN	15.83	86.39	176.6	75.9	86.391
2	UNKNOWN	19.03	13.61	27.1	12.0	13.609
Total			100.00	203.7	87.9	100.000

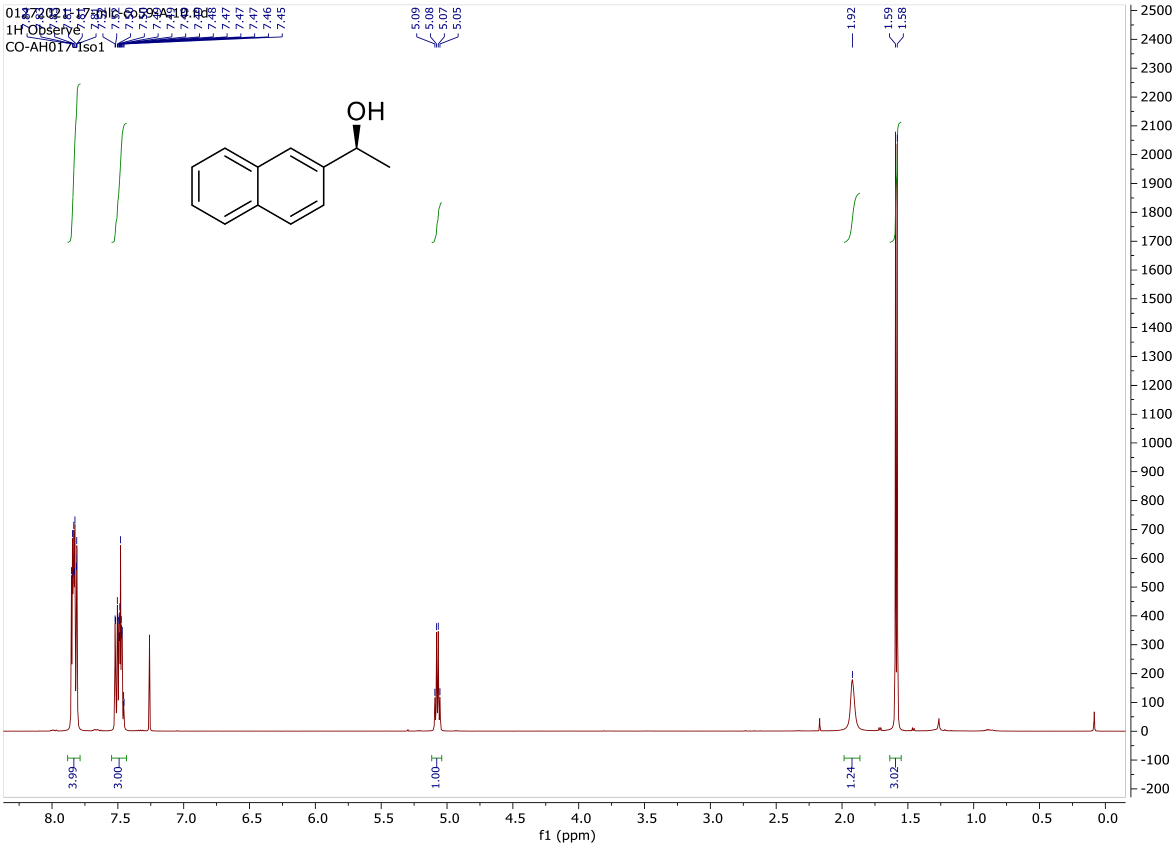

1H Observe
CO-AH017-Iso1
OH
5.09
5.08
5.07
5.05
1.92
1.59
1.58
3.99
3.00
1.00
1.24
3.02
f1 (ppm)

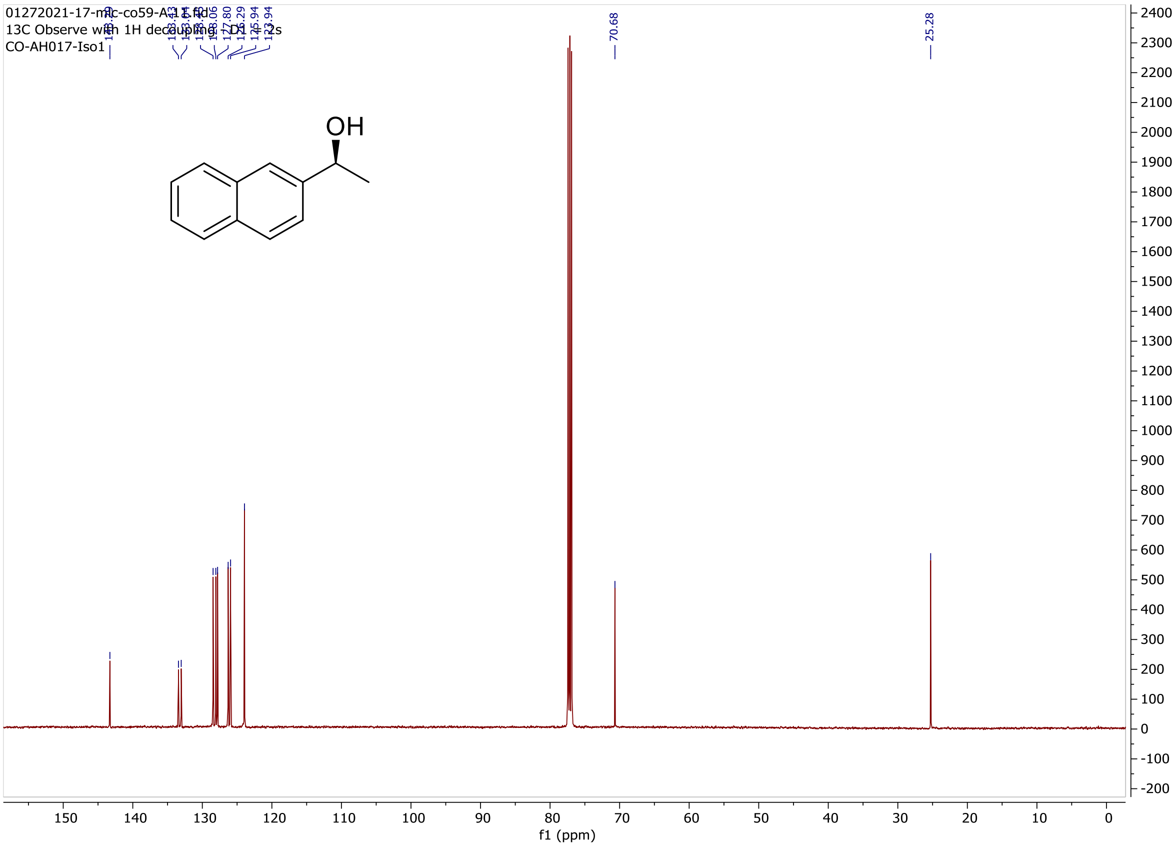

13C Observe with 1H decoupling
CO-AH017-Iso1
OH
70.68
25.28
f1 (ppm)

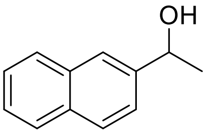

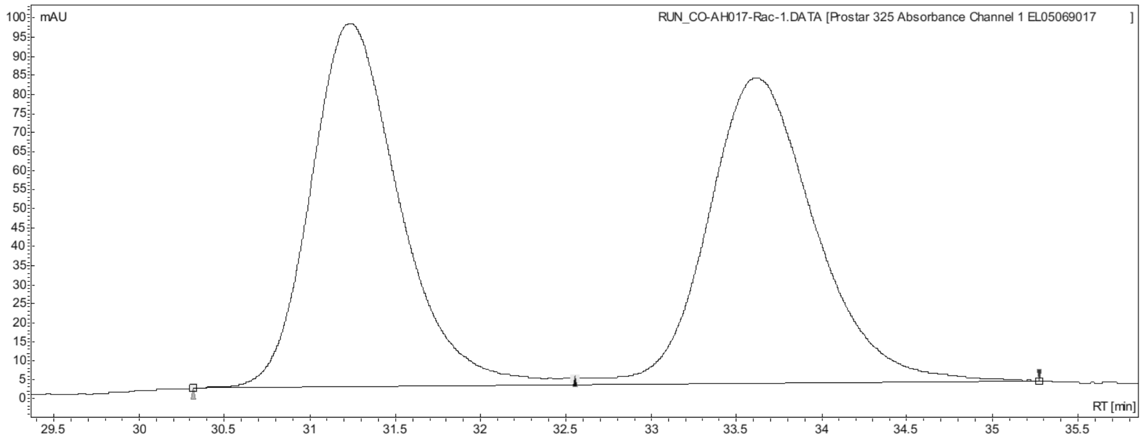

#	Name	Time [Min]	Quantity [% Area	Height [mAU]	Area [mAU.Min]	Area % [%]
1	UNKNOWN	31.24	49.69	95.6	57.8	49.686
2	UNKNOWN	33.62	50.31	80.3	58.5	50.314
Total			100.00	175.9	116.3	100.000

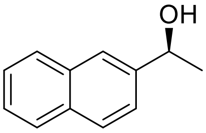

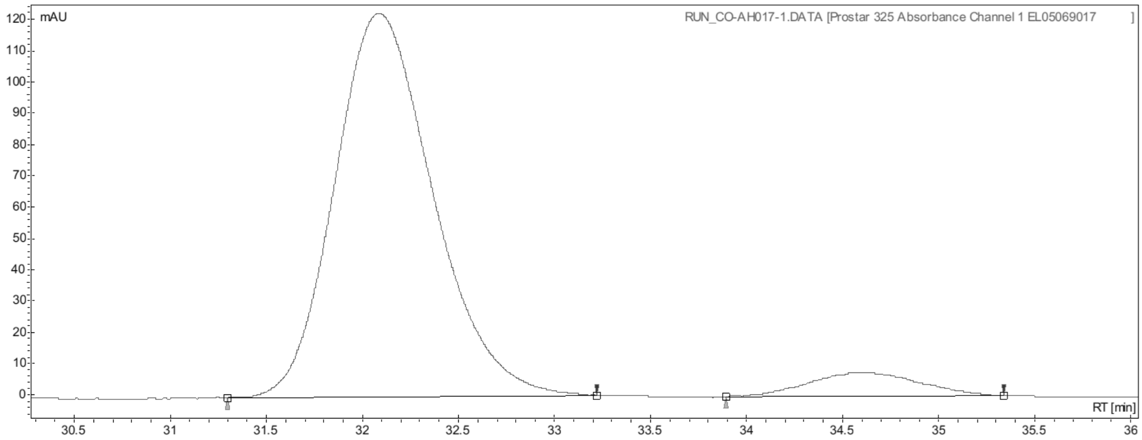

#	Name	Time [Min]	Quantity [% Area	Height [mAU]	Area [mAU.Min]	Area % [%]
1	UNKNOWN	32.08	93.52	122.6	72.6	93.521
2	UNKNOWN	34.61	6.48	7.6	5.0	6.479
Total			100.00	130.3	77.7	100.000

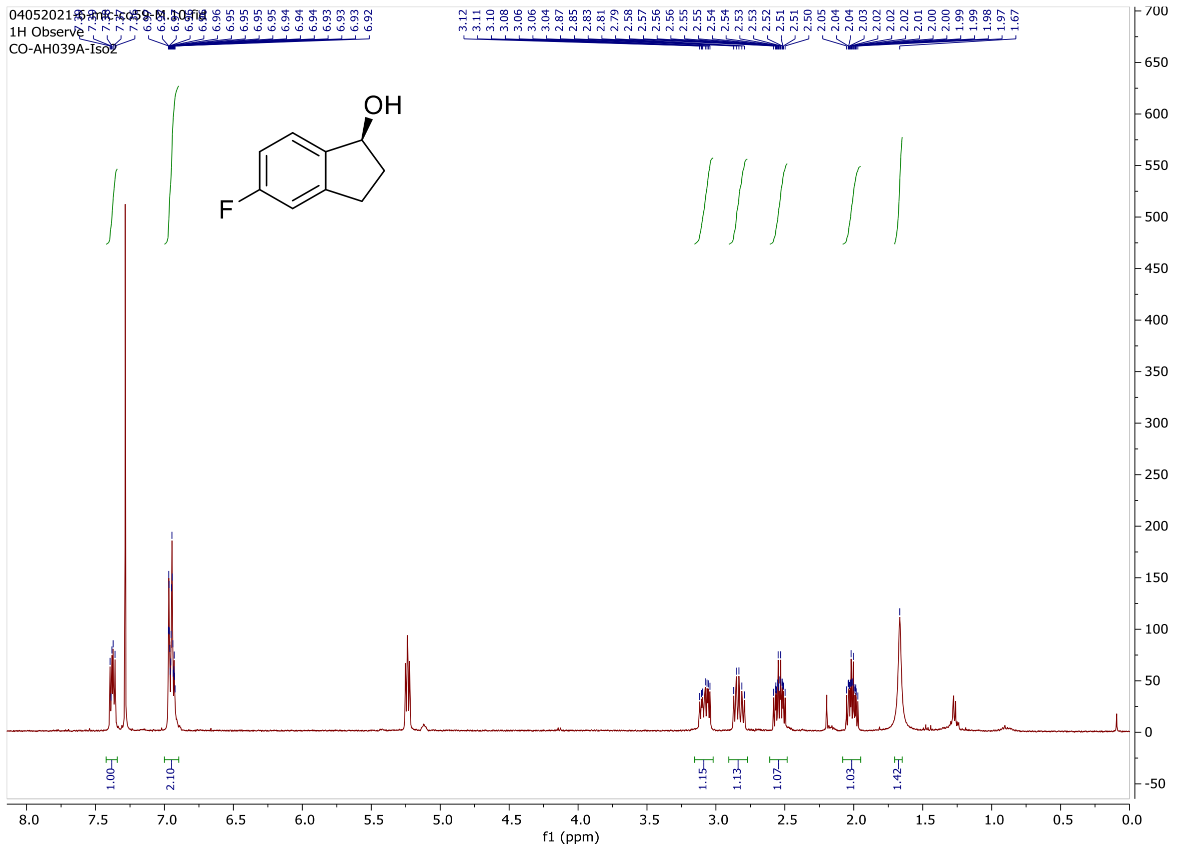
1H Observe
CO-AH039A-Iso2
OH
F
f1 (ppm)

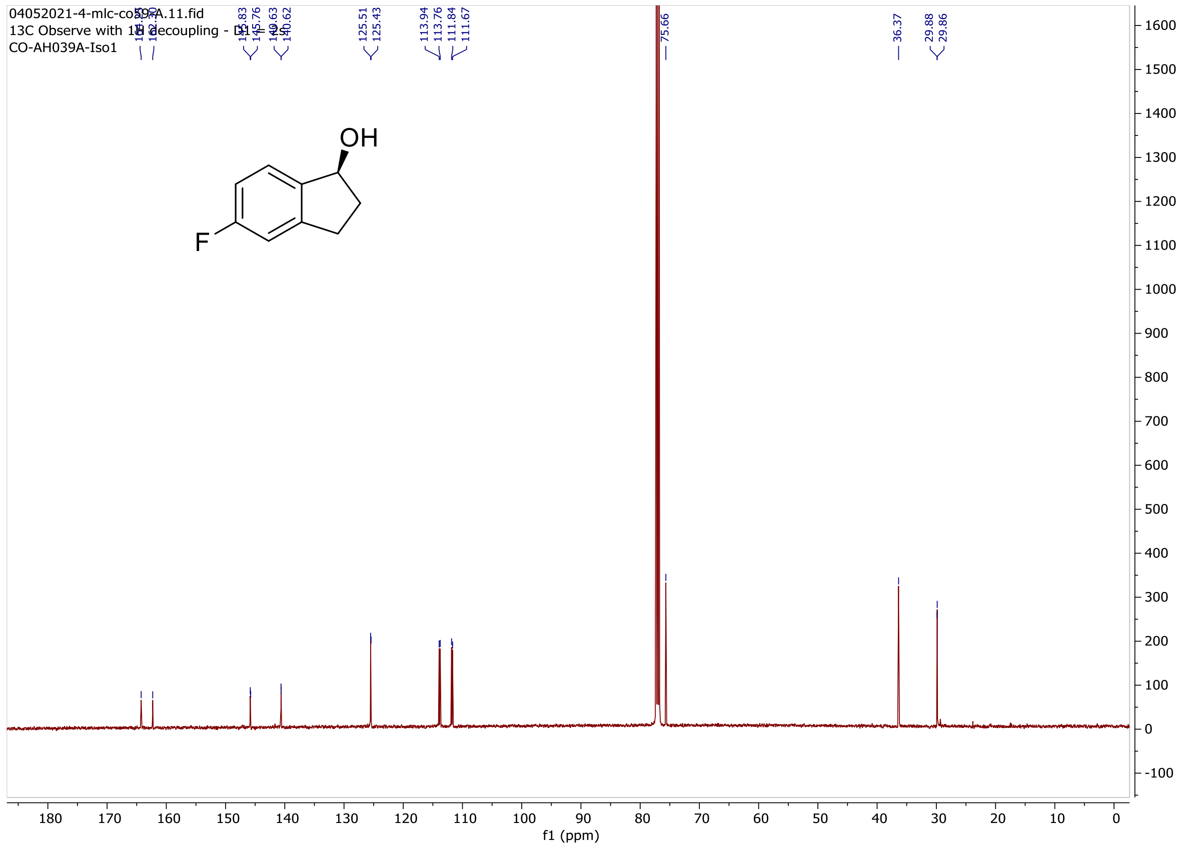
04052021-4-mlc-co59-A.11.fid
13C Observe with 1H decoupling
CO-AH039A-Iso1
OH
F
f1 (ppm)

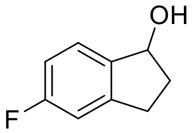

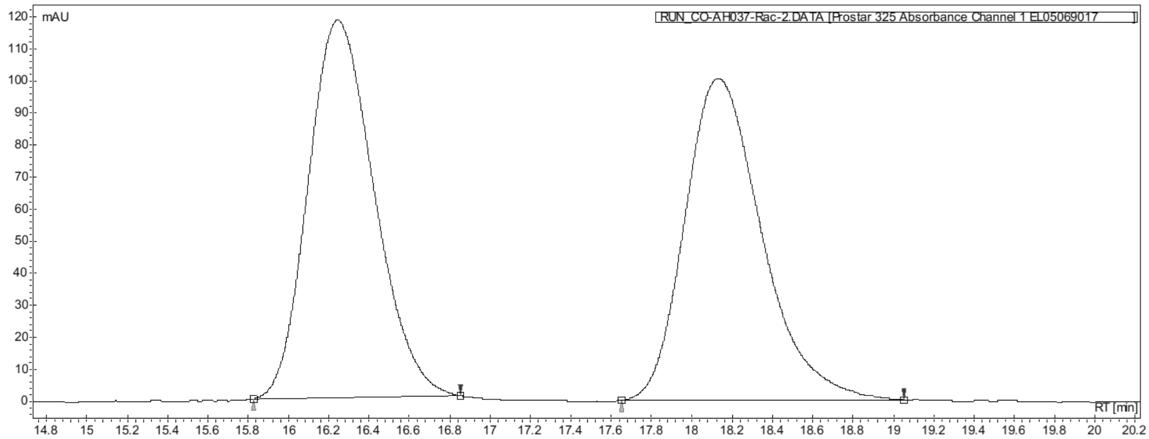

#	Name	Time [Min]	Quantity [% Area]	Height [mAU]	Area [mAU.Min]	Area % [%]
1	UNKNOWN	16.24	50.31	118.0	44.9	50.308
2	UNKNOWN	18.13	49.69	100.5	44.3	49.692
Total			100.00	218.5	89.2	100.000

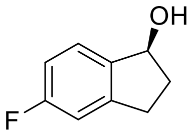

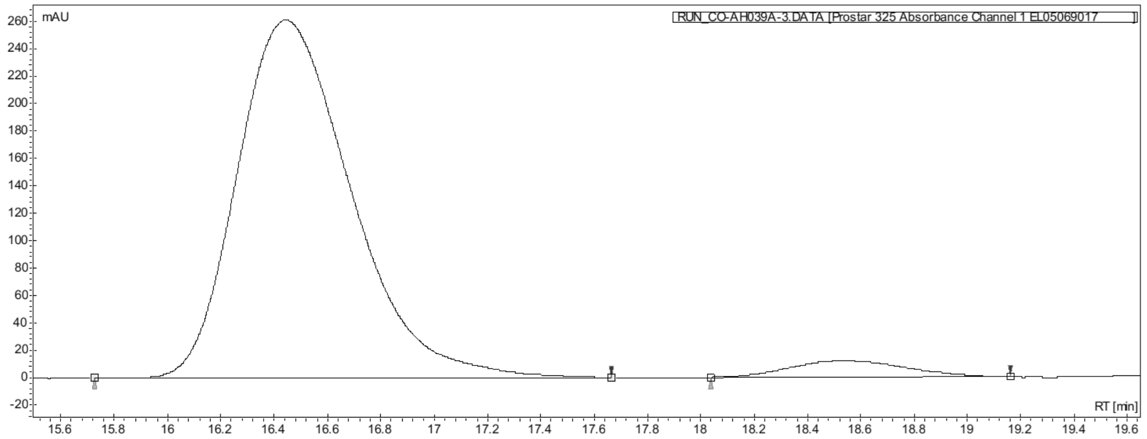

#	Name	Time [Min]	Quantity [% Area]	Height [mAU]	Area [mAU.Min]	Area % [%]
1	UNKNOWN	16.44	95.58	260.8	128.4	95.579
2	UNKNOWN	18.51	4.42	11.8	5.9	4.421
Total			100.00	272.6	134.3	100.000

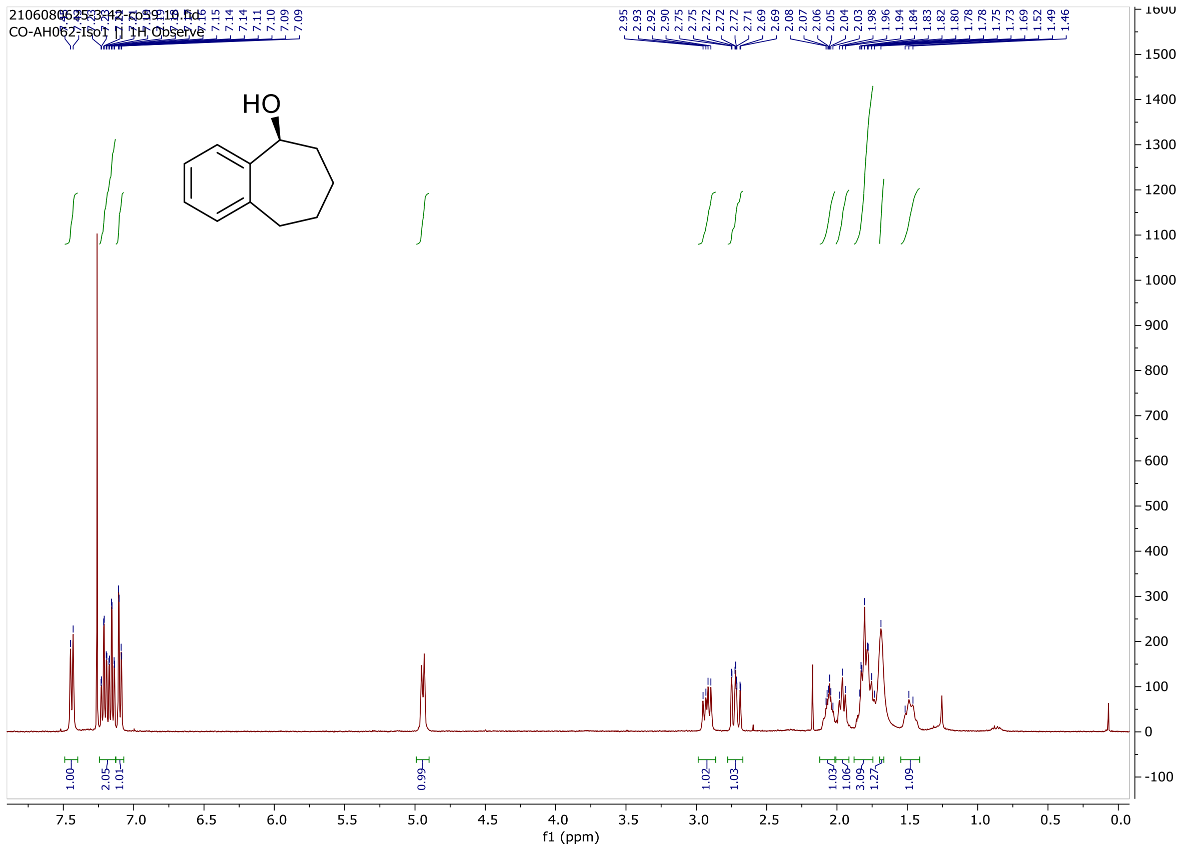
CO-AH062-Iso1 || 1H Observe
HO
2.95 2.93 2.92 2.90 2.75 2.75 2.72 2.72 2.72 2.71 2.69 2.69 2.08 2.07 2.06 2.05 2.04 2.03 1.98 1.96 1.94 1.84 1.83 1.82 1.80 1.78 1.78 1.75 1.73 1.69 1.52 1.49 1.46
7.15 7.14 7.11 7.10 7.09 7.09
1.00 2.05 1.01 0.99 1.02 1.03 1.03 1.06 3.09 1.27 1.09
f1 (ppm)

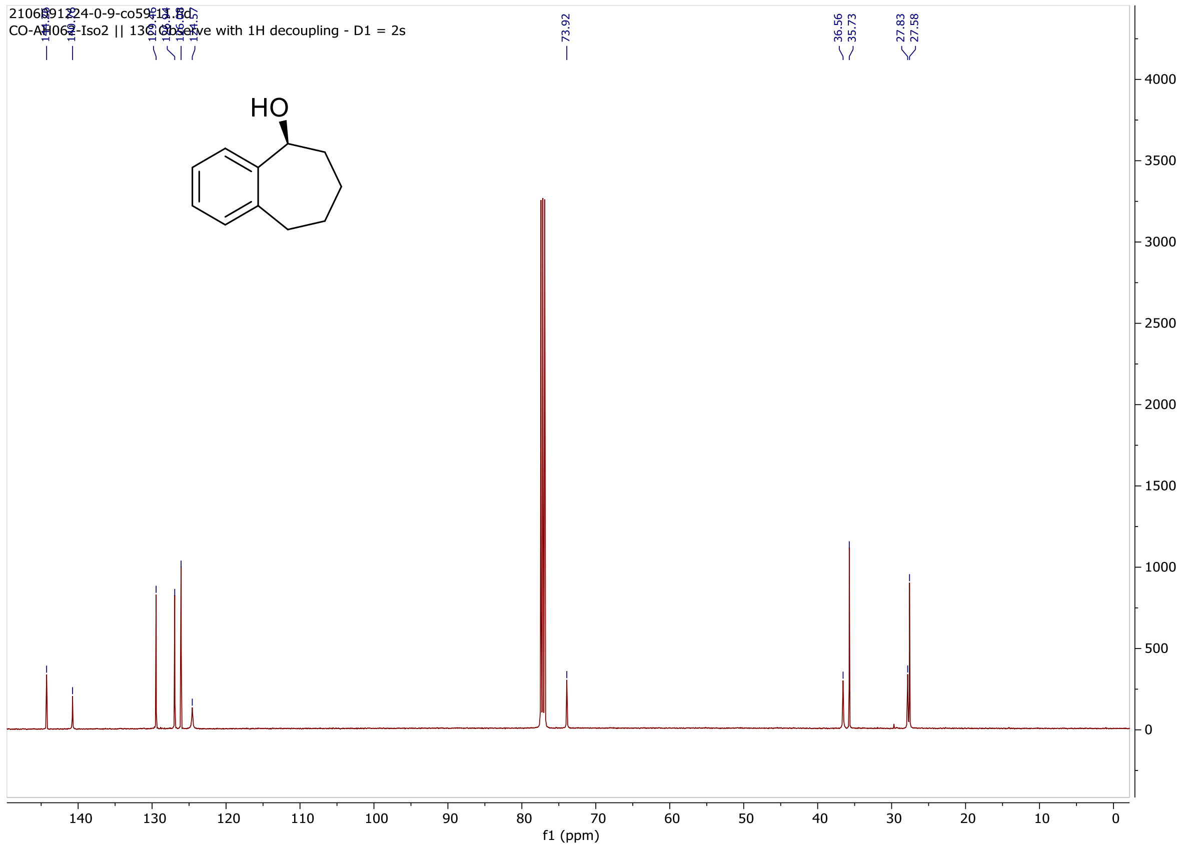
CO-AH062-Iso2 || 13C Observe with 1H decoupling - D1 = 2s
HO
73.92
36.56 35.73
27.83 27.58
f1 (ppm)

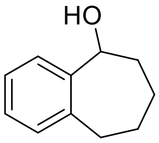

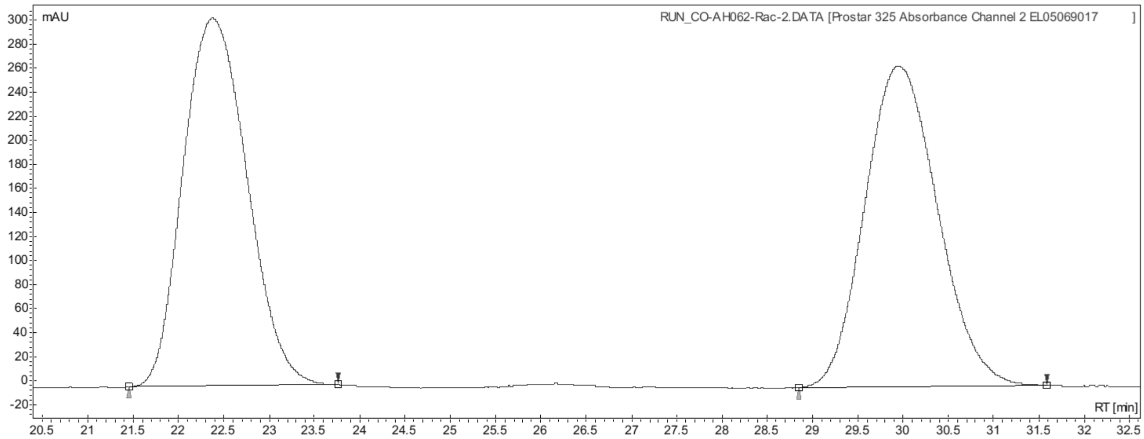

	#	Name	Time [Min]	Quantity [% Are	Height [mAU]	Area [mAU.Min]	Area % [%]
▶	1	UNKNOWN	22.37	50.16	306.2	251.0	50.164
	2	UNKNOWN	29.94	49.84	267.0	249.4	49.836
	Total			100.00	573.1	500.4	100.000

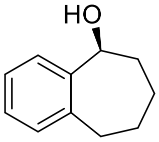

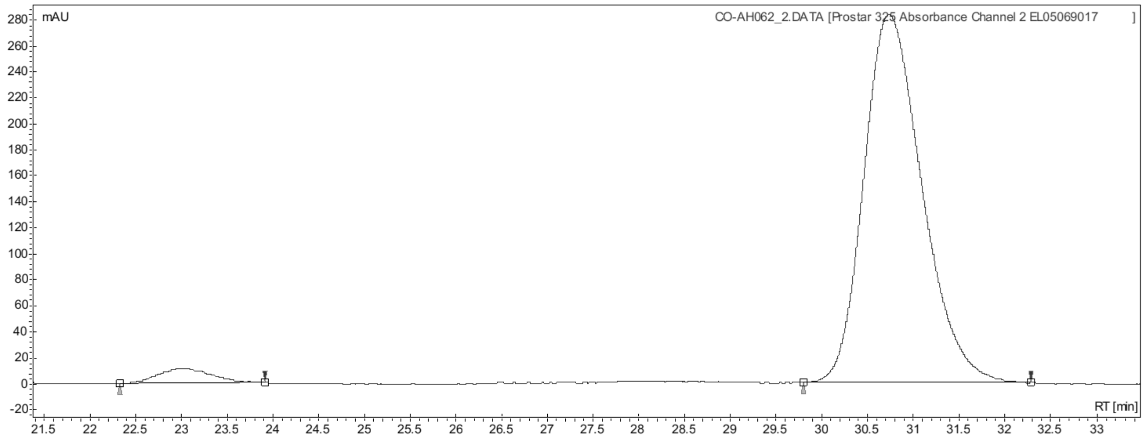

	#	Name	Time [Min]	Quantity [% Are	Height [mAU]	Area [mAU.Min]	Area % [%]
▶	1	UNKNOWN	22.99	3.31	11.0	7.2	3.314
	2	UNKNOWN	30.73	96.69	283.0	208.9	96.686
	Total			100.00	294.0	216.0	100.000

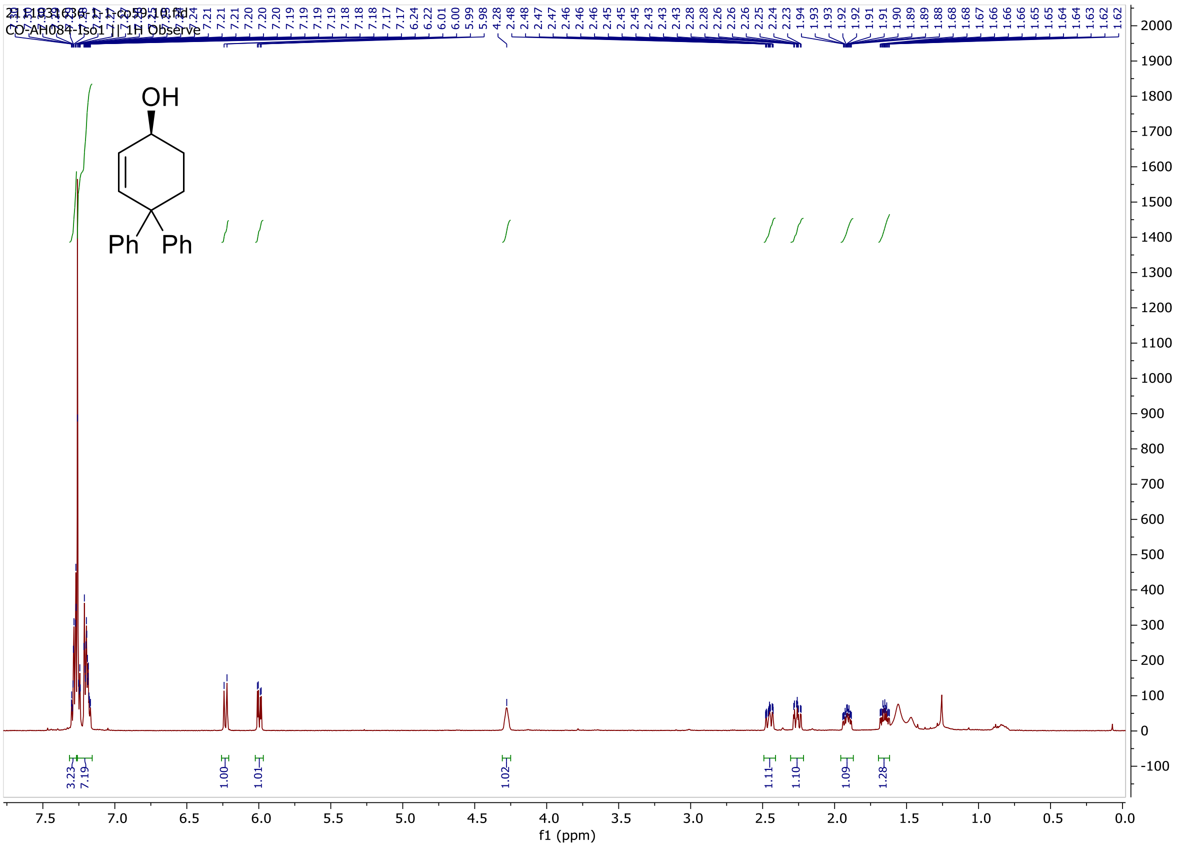
CO-AH084-Iso1 || 1H Observe
OH
Ph
Ph
f1 (ppm)

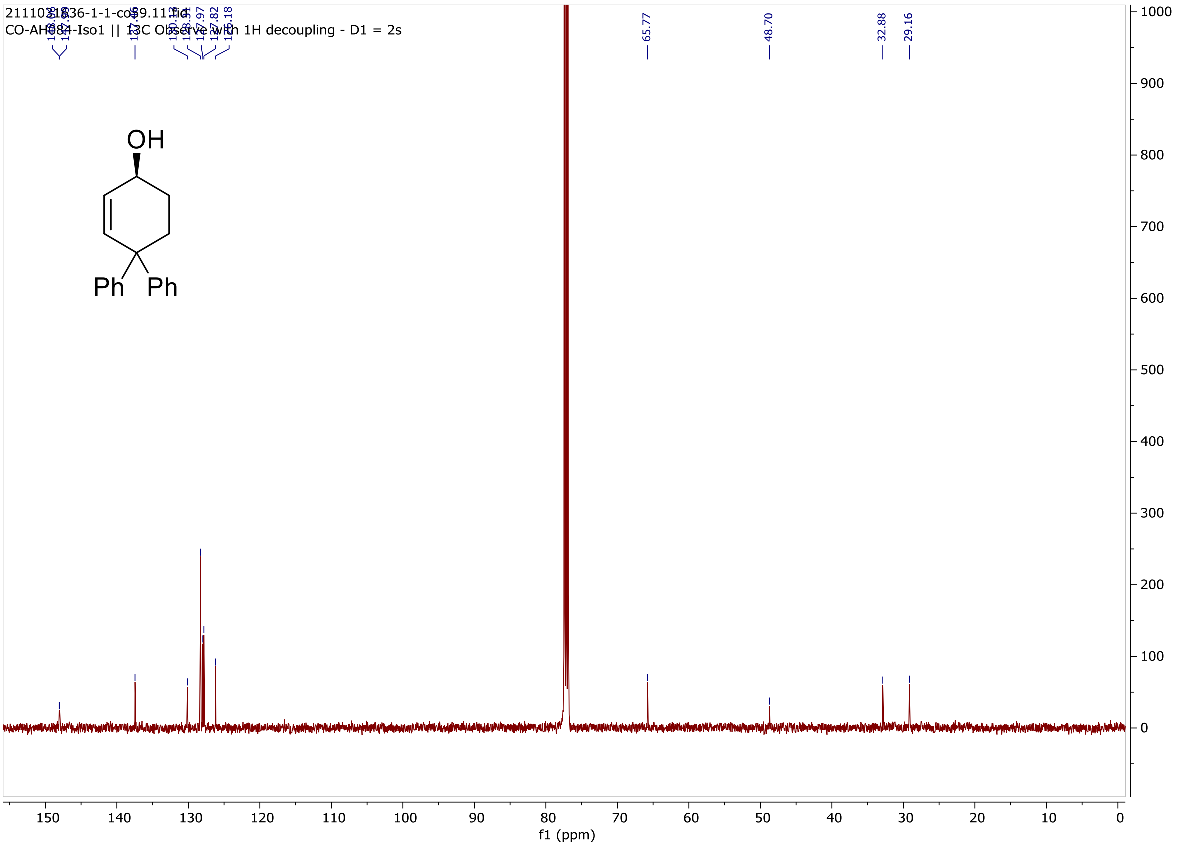
CO-AH084-Iso1 || 13C Observe with 1H decoupling - D1 = 2s
65.77
48.70
32.88
29.16
OH
Ph
Ph
f1 (ppm)

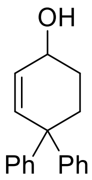

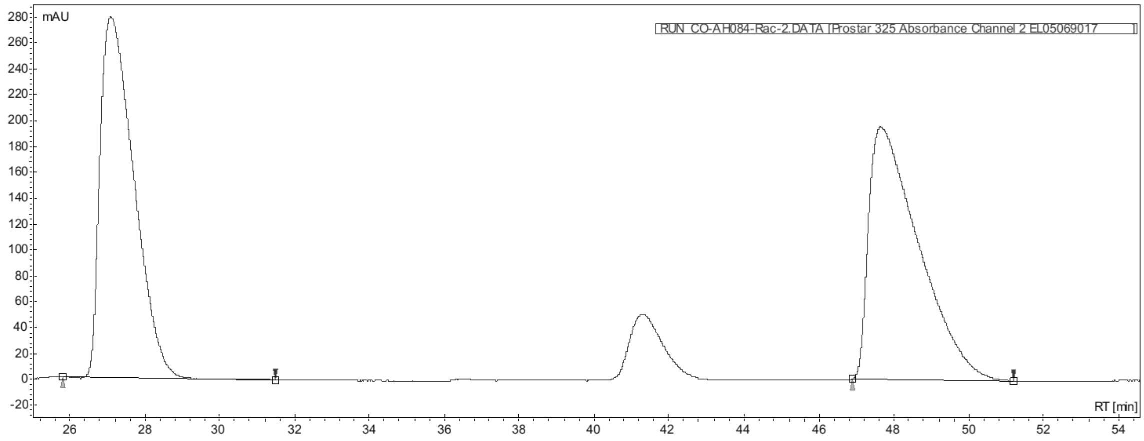

#	Name	Time [Min]	Quantity [% Area	Height [mAU]	Area [mAU.Min]	Area % [%]
1	UNKNOWN	27.09	49.01	278.8	291.1	49.007
2	UNKNOWN	47.65	50.99	195.5	302.9	50.993
Total			100.00	474.3	594.0	100.000

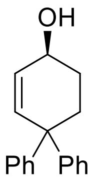

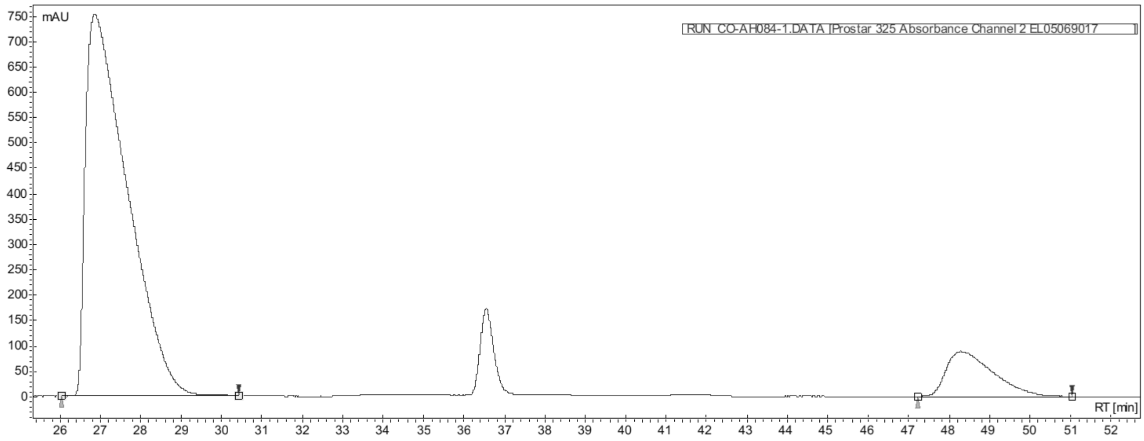

#	Name	Time [Min]	Quantity [% Area	Height [mAU]	Area [mAU.Min]	Area % [%]
1	UNKNOWN	26.85	88.38	753.5	901.2	88.379
2	UNKNOWN	48.29	11.62	88.6	118.5	11.621
Total			100.00	842.1	1019.7	100.000

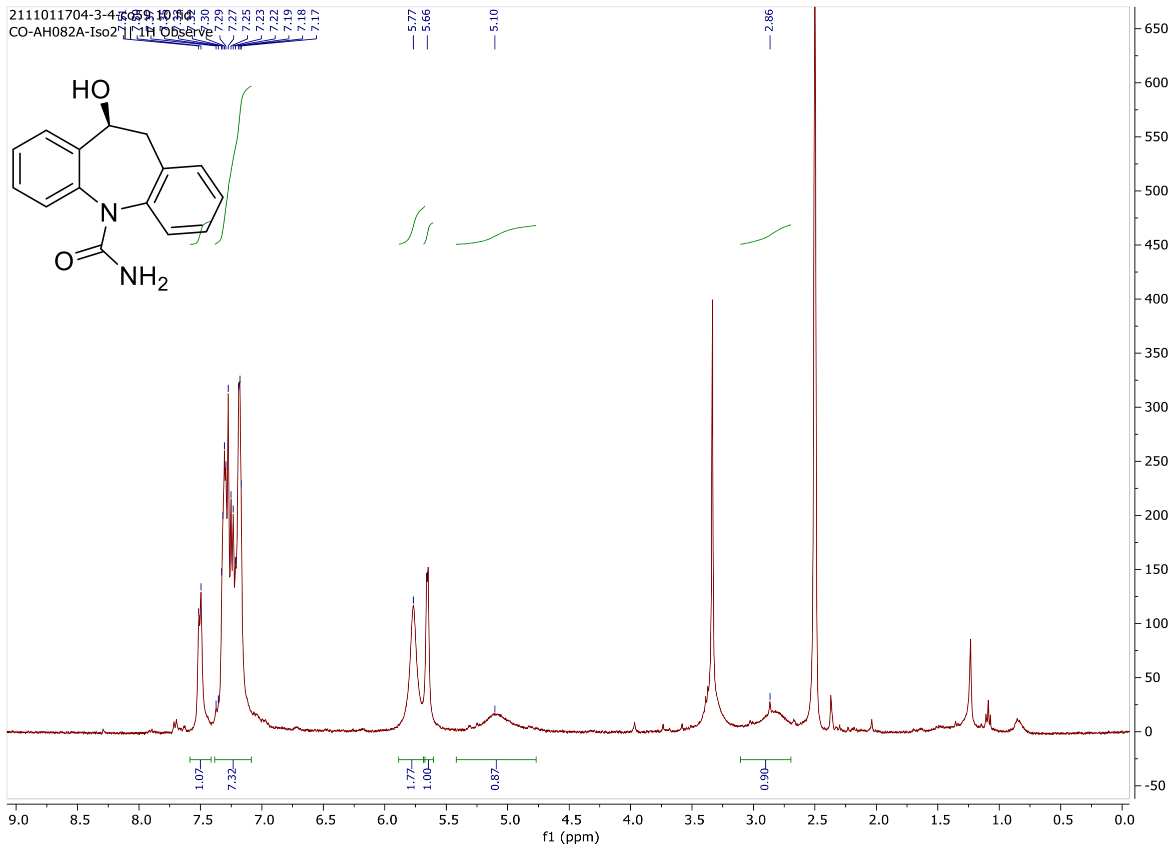

2111011704-3-4-co59.10.fid
CO-AH082A-Iso2 || 1H Observe
7.30
7.29
7.27
7.25
7.23
7.22
7.19
7.18
7.17
5.77
5.66
5.10
2.86
HO
N
O
NH2
1.07
7.32
1.77
1.00
0.87
0.90
f1 (ppm)

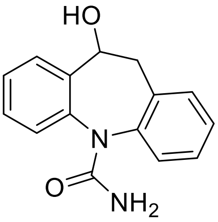

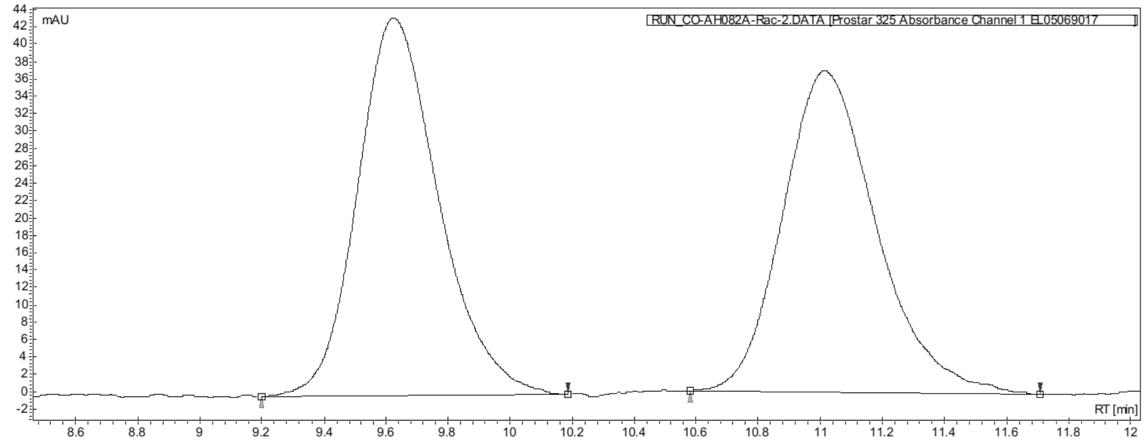

#	Name	Time [Min]	Quantity [% Area	Height [mAU]	Area [mAU.Min]	Area % [%]
1	UNKNOWN	9.62	50.34	43.4	13.2	50.338
2	UNKNOWN	11.01	49.66	37.0	13.0	49.662
Total			100.00	80.5	26.2	100.000

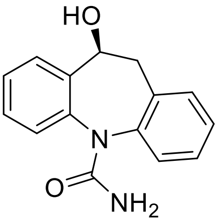

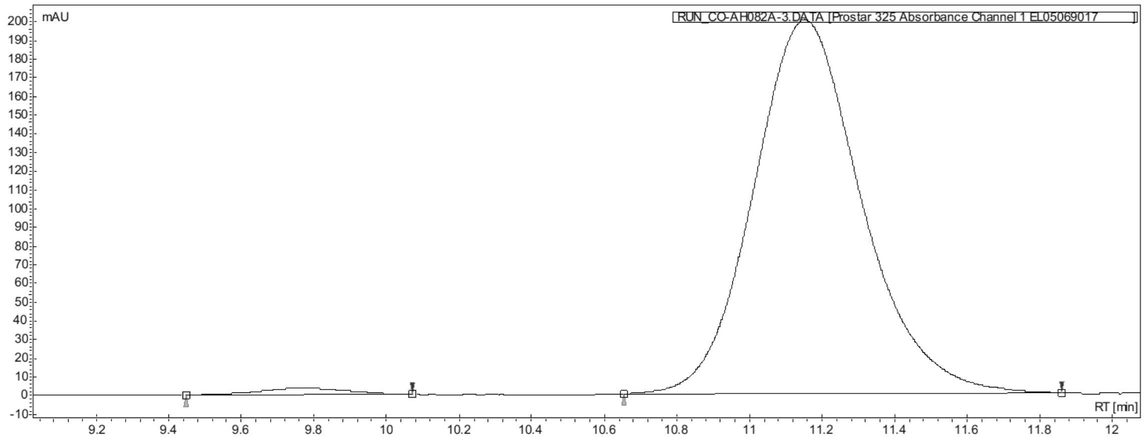

#	Name	Time [Min]	Quantity [% Area	Height [mAU]	Area [mAU.Min]	Area % [%]
1	UNKNOWN	9.77	1.40	3.7	1.0	1.401
2	UNKNOWN	11.15	98.60	199.9	68.8	98.599
Total			100.00	203.6	69.8	100.000

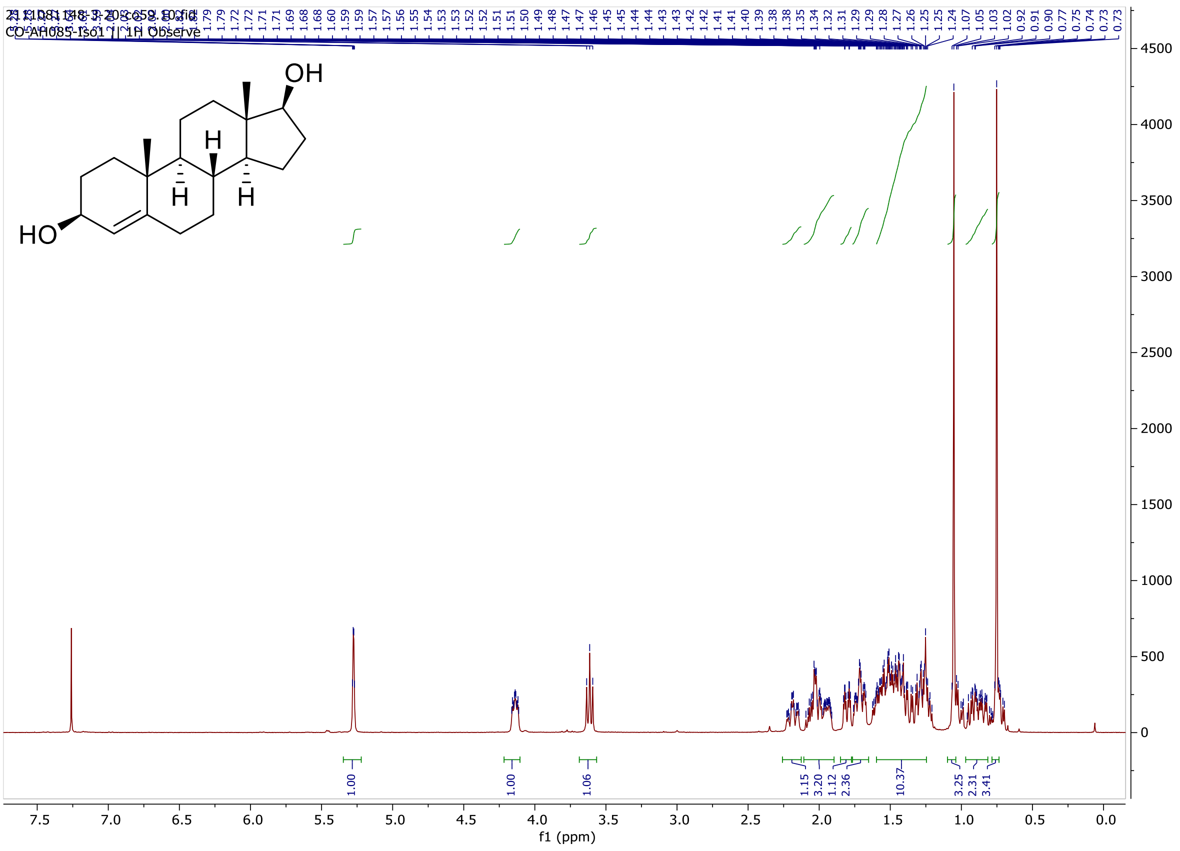
CO-AH085-Iso1 || 1H Observe
1.79 1.79 1.72 1.72 1.71 1.71 1.69 1.68 1.68 1.60 1.59 1.59 1.57 1.57 1.56 1.55 1.54 1.53 1.53 1.52 1.52 1.51 1.51 1.50 1.49 1.48 1.47 1.47 1.46 1.45 1.45 1.44 1.44 1.43 1.43 1.42 1.42 1.41 1.41 1.40 1.39 1.38 1.38 1.35 1.34 1.32 1.31 1.29 1.29 1.28 1.27 1.26 1.25 1.25 1.24 1.07 1.05 1.03 1.02 0.92 0.91 0.90 0.77 0.75 0.74 0.73 0.73
OH
H
H
H
HO
1.00
1.00
1.06
1.15
3.20
1.12
2.36
10.37
3.25
2.31
3.41
f1 (ppm)

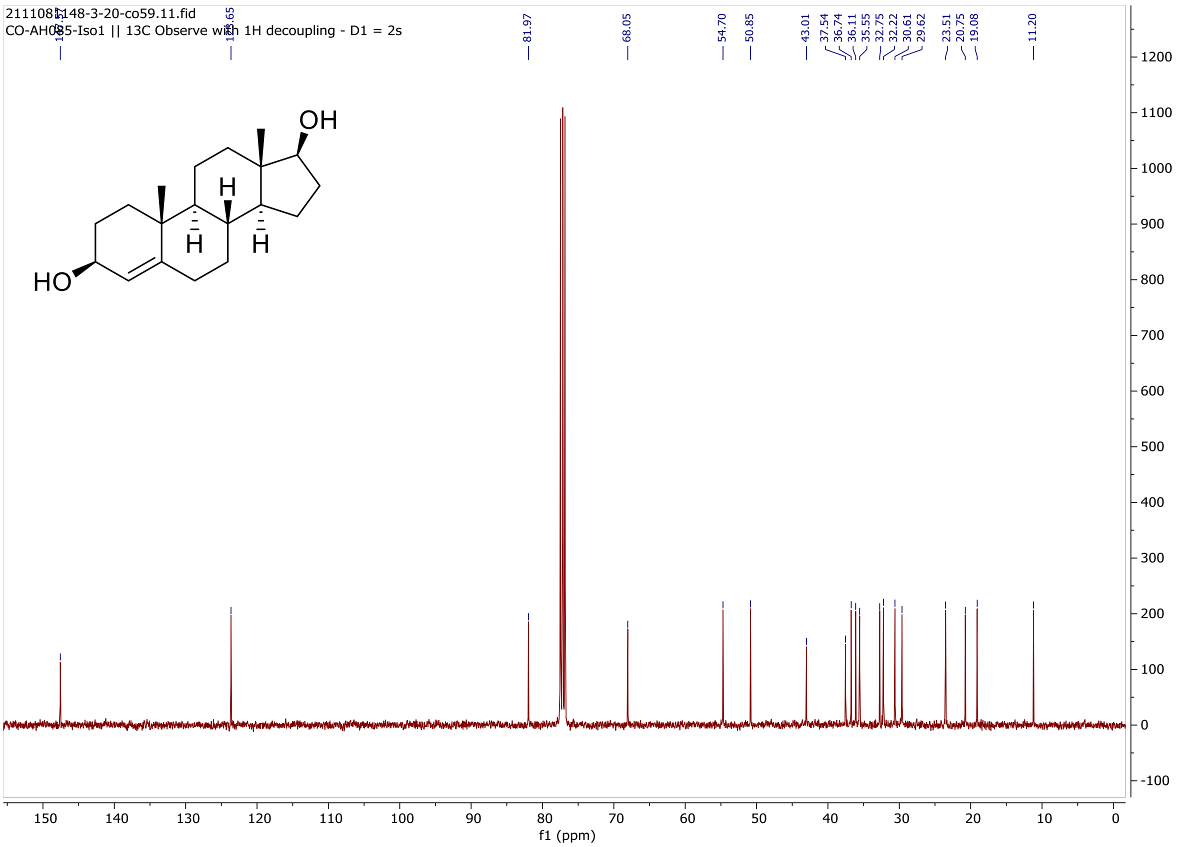
2111081148-3-20-co59.11.fid
CO-AH085-Iso1 || 13C Observe with 1H decoupling - D1 = 2s
81.97
68.05
54.70
50.85
43.01
37.54
36.74
36.11
35.55
32.75
32.22
30.61
29.62
23.51
20.75
19.08
11.20
OH
H
H
H
HO
f1 (ppm)

Section 3.2: Cartesian Coordinates and Energetics of Optimised Structures

Raw data and cartesian coordinates obtained from geometry optimisation and frequency calculations at the level of RI-BP86(PCM_{EtOH})/def2-SVP and single-point energies at the level of PBE0-D3(PCM_{EtOH})/def2-TZVP.

The ligand backbones correspond to the labelling system in the paper **1'**, **3'** and **4'**.

Gibbs Free Energies for individual species have been evaluated at 323.15 K using the Enthalpy correction (δH) and Entropy (S) obtained from frequency calculations at the level of optimisation:

$$G_{323.15} = E_{sp} + \delta H_{298.15} - T * S_{298.15}.$$

```
Filenames correspond to the reaction profile as follows:
0              precatalyst, 6-coordinate cationic complex
1              coordinatively unsaturated catalyst (bare Mn and N)
1s                    mono-solvated
2              H2 eta bound to Mn
2s                    mono-solvated
TSH2           heterolytic splitting of H2
TSH2s                 mono-solvated (solvent assisted)
3              activated catalyst (Mn-H and N-H)
3s                    mono-solvated
TSHy-re/si     hydride transfer
5-R/S          associated product complex

off-cycle intermediates
1s-alkox              alkoxide form with Mn-OEt and N-H
1s-EtOH-alkox                mono-solvated
1s-EtOH-alkox-EtOH           di-solvated
1-HCO3-               bicarb bound (anionic)
1-HCO3--EtOH2                di-solvated
1-HCO3--EtOH5                penta-solvated

1/0-fRSR
Frequencies, energies and thermodynamic properties:
 Lowest Vibrational Mode (1/cm) =                         17.1689
 E(RB-P86) (a.u.) =                                     -4365.14773078
 Thermal correction to Enthalpy (a.u.) =                  0.568188
 Thermal correction to Gibbs Free Energy (a.u.) =         0.460557
 Total Entropy (cal/Kmol) =                               226.527
 E(RPBE1PBE) (a.u.) =                                   -4364.47968835
Optimised cartesian coordinates (Angstrom):
 Fe    -3.021896  -0.732175  -0.846079
 Mn     1.333392  -0.657319   1.485983
 P      0.115969   0.737483  -0.016702
 O      2.109681   1.766888   2.989912
 O     -0.975364  -1.116751   3.276090
 N      0.857274  -2.341328   0.241704
 N      2.975003  -0.651995   0.209584
 C     -1.053269  -0.226484  -1.034682
 C     -1.214936  -1.682209  -1.011717
 C     -2.070279  -2.031193  -2.123103
 H     -2.408835  -3.042067  -2.380987
 C     -2.446487  -0.830419  -2.814752
 H     -3.117259  -0.772026  -3.681874
 C     -1.833271   0.283283  -2.150109
 H     -1.929472   1.336885  -2.440790
 C     -3.724034  -0.522444   1.081547
 H     -3.112772  -0.458085   1.990321
 C     -4.231842  -1.732779   0.492969
 H     -4.072326  -2.751740   0.870127
 C     -4.969904  -1.376138  -0.690549
 H     -5.461033  -2.075676  -1.379860
 C     -4.920761   0.056176  -0.831523
 H     -5.368136   0.638921  -1.647599
 C     -4.148055   0.585076   0.261257
 H     -3.909679   1.642778   0.432187
 C      1.227458   1.571984  -1.241818
 C      2.020709   2.657072  -0.797622
 H      1.931458   3.024847   0.237040
 C      2.915215   3.289015  -1.676495
 H      3.518727   4.138497  -1.320148
 C      3.038723   2.840429  -3.004891
 H      3.739823   3.337739  -3.693326
```

```
 C      2.261557   1.757453  -3.449402
 H      2.348322   1.402201  -4.488206
 C      1.359675   1.123935  -2.573893
 H      0.744167   0.290550  -2.945466
 C     -0.857217   2.167040   0.654524
 C     -1.270179   2.208338   2.003508
 H     -0.986901   1.410677   2.702989
 C     -2.055897   3.275008   2.476512
 H     -2.366949   3.291975   3.532646
 C     -2.436852   4.312784   1.608971
 H     -3.050845   5.147938   1.980920
 C     -2.021961   4.284489   0.264709
 H     -2.307870   5.097429  -0.421031
 C     -1.233473   3.223154  -0.209766
 H     -0.900979   3.228268  -1.259022
 C     -0.600524  -2.663867  -0.027863
 H     -1.091912  -2.543473   0.957164
 C      1.694604  -2.344105  -0.988191
 H      1.902226  -3.374100  -1.343953
 H      1.115883  -1.842513  -1.794590
 C      2.977269  -1.586323  -0.780264
 C      4.084873   0.108486   0.389537
 H      4.052441   0.847951   1.201842
 C      5.229954  -0.029509  -0.401349
 H      6.099212   0.615798  -0.209978
 C      5.241943  -1.000851  -1.415047
 C      4.098717  -1.789324  -1.602529
 H      4.063644  -2.562872  -2.383855
 C     -0.777303  -4.127190  -0.458338
 H     -1.853712  -4.387310  -0.476035
 H     -0.363119  -4.326447  -1.466948
 H     -0.287735  -4.808280   0.267593
 C      1.800890   0.812432   2.389545
 C     -0.089025  -0.908088   2.540161
 C      2.314568  -1.675580   2.622432
 O      2.935353  -2.283184   3.397130
 H      1.189906  -3.115915   0.833723
 H      6.130061  -1.144933  -2.048568
```

1/0-fRSS

```
Frequencies, energies and thermodynamic properties:
 Lowest Vibrational Mode (1/cm) =                           8.3069
 E(RB-P86) (a.u.) =                                     -4365.14017992
 Thermal correction to Enthalpy (a.u.) =                    0.568499
 Thermal correction to Gibbs Free Energy (a.u.) =           0.460049
 Total Entropy (cal/Kmol) =                                 228.253
 E(RPBE1PBE) (a.u.) =                                   -4364.47162048
Optimised cartesian coordinates (Angstrom):
 Fe    -2.979361  -0.138333  -0.678576
 Mn     2.074265  -0.421164  -0.676628
 P      0.300001   0.871908   0.330959
 N      0.597779  -1.439094  -1.894743
 N      1.851085  -2.197107   0.406041
 C     -1.188664   0.888989  -0.734462
 C     -1.382646   0.152290  -1.979052
 C     -2.627061   0.604708  -2.555138
 H     -3.066610   0.231761  -3.490108
 C     -3.190946   1.616220  -1.705922
 H     -4.141946   2.139376  -1.869948
 C     -2.325375   1.780708  -0.573146
 H     -2.489070   2.472317   0.262342
 C     -3.052662  -1.663614   0.728309
 H     -2.215745  -2.096926   1.290955
 C     -3.565005  -2.125035  -0.536348
 H     -3.186427  -2.971638  -1.123677
 C     -4.672251  -1.282335  -0.898398
 H     -5.268890  -1.359133  -1.816881
 C     -4.846933  -0.303829   0.145212
 H     -5.597062   0.497858   0.156134
 C     -3.841949  -0.536216   1.149129
 H     -3.693040   0.048369   2.065923
 C      0.801075   2.659607   0.384443
 C      1.607064   3.118942   1.452459
 H      1.882193   2.439630   2.274091
 C      2.052951   4.450894   1.483267
 H      2.674541   4.796248   2.324349
 C      1.704087   5.338620   0.448907
```

```
 H      2.051932   6.383245   0.475948
 C      0.908803   4.886296  -0.618025
 H      0.630445   5.574057  -1.432023
 C      0.459948   3.553565  -0.653936
 H     -0.162734   3.216258  -1.496073
 C     -0.318197   0.608662   2.062597
 C     -0.087327  -0.610314   2.734412
 H      0.488034  -1.406770   2.246520
 C     -0.582897  -0.815238   4.035295
 H     -0.389742  -1.771822   4.545357
 C     -1.312685   0.197136   4.680425
 H     -1.698955   0.038109   5.699454
 C     -1.538843   1.420544   4.022316
 H     -2.100811   2.223968   4.524002
 C     -1.041082   1.629593   2.725517
 H     -1.205568   2.602819   2.238706
 C     -0.443400  -0.758670  -2.744348
 H     -1.072268  -1.576165  -3.166436
 C     -0.046772  -2.493996  -1.068141
 H     -0.910682  -2.010136  -0.560635
 H     -0.460119  -3.302956  -1.707547
 C      0.888357  -3.049612  -0.039006
 C      2.699153  -2.624119   1.378820
 H      3.471857  -1.915935   1.709765
 C      2.609497  -3.896670   1.950918
 H      3.319738  -4.185256   2.738851
 C      1.616381  -4.779331   1.494393
 C      0.748549  -4.347829   0.482416
 H     -0.038453  -5.006895   0.087939
 C      0.201859  -0.038910  -3.939345
 H     -0.588482   0.254320  -4.657132
 H      0.740903   0.878461  -3.641800
 H      0.903835  -0.712405  -4.474391
 C      2.361505   0.989960  -1.743566
 O      2.667968   1.890568  -2.421954
 C      3.210301   0.275549   0.512185
 O      3.970929   0.720039   1.279710
 C      3.438350  -1.241677  -1.538346
 O      4.348355  -1.722822  -2.082148
 H      1.206736  -1.920314  -2.572449
 H      1.523962  -5.791530   1.916069
```

```
1/0-mRSR
Frequencies, energies and thermodynamic properties:
 Lowest Vibrational Mode (1/cm) =                          26.5948
 E(RB-P86) (a.u.) =                                     -4365.13956155
 Thermal correction to Enthalpy (a.u.) =                   0.568053
 Thermal correction to Gibbs Free Energy (a.u.) =          0.460578
 Total Entropy (cal/Kmol) =                                226.200
 E(RPBE1PBE) (a.u.) =                                   -4364.47016730
Optimised cartesian coordinates (Angstrom):
 Fe    -1.692902  -2.508908  -0.449385
 Mn     1.520504   0.614950   0.339860
 P     -0.760520   0.905635   0.069880
 O      1.634083   2.773722   2.343620
 N      1.559050  -0.998222  -1.119313
 N      3.522080   0.095952   0.425957
 C     -1.513801  -0.492765  -0.827986
 C     -0.806955  -1.286293  -1.826673
 C     -1.772989  -2.147803  -2.463949
 H     -1.551289  -2.873571  -3.258062
 C     -3.056065  -1.916541  -1.860477
 H     -3.986313  -2.447063  -2.102193
 C     -2.902658  -0.912417  -0.844164
 H     -3.697380  -0.532687  -0.190175
 C     -1.328913  -2.985463   1.532588
 H     -1.150373  -2.284343   2.356596
 C     -0.324543  -3.576226   0.682719
 H      0.762400  -3.452890   0.780731
 C     -0.990674  -4.431786  -0.265341
 H     -0.508409  -5.020553  -1.056591
 C     -2.404048  -4.375312   0.005425
 H     -3.188726  -4.901266  -0.554077
 C     -2.611360  -3.481198   1.115414
 H     -3.580894  -3.208263   1.552066
 C     -1.236848   2.386460  -0.950301
 C     -0.870958   3.669353  -0.479182
```

H	-0.329313	3.779756	0.474016
C	-1.206402	4.817658	-1.214114
H	-0.918976	5.810490	-0.833768
C	-1.905764	4.700179	-2.430322
H	-2.166020	5.601504	-3.007205
C	-2.272663	3.429151	-2.903451
H	-2.824334	3.328816	-3.851436
C	-1.943115	2.274942	-2.167471
H	-2.247842	1.286931	-2.544191
C	-1.853833	1.158107	1.553882
C	-1.430758	0.849364	2.864092
H	-0.419261	0.469184	3.055040
C	-2.295777	1.035227	3.958144
H	-1.944923	0.791897	4.973185
C	-3.593518	1.534461	3.757843
H	-4.268798	1.681709	4.615290
C	-4.021251	1.857474	2.457197
H	-5.032434	2.260493	2.289818
C	-3.157349	1.678757	1.364045
H	-3.500400	1.960369	0.356604
C	0.617937	-1.119308	-2.294512
H	0.897455	-2.063182	-2.816658
C	2.967314	-1.188840	-1.557182
H	3.140295	-2.223956	-1.922074
H	3.152473	-0.514599	-2.420653
C	3.947773	-0.824820	-0.478547
C	4.390354	0.539961	1.369603
H	4.007012	1.282665	2.083491
C	5.709910	0.082926	1.447349
H	6.372687	0.479182	2.229982
C	6.151214	-0.881855	0.527098
C	5.254042	-1.341734	-0.446579
H	5.555965	-2.095299	-1.188695
C	0.737777	0.026677	-3.308902
H	0.078995	-0.197580	-4.170739
H	0.410598	0.994307	-2.883030
H	1.765675	0.140754	-3.706373
C	1.585362	1.910861	1.550933
C	1.941842	1.900618	-0.919585
O	2.268980	2.764422	-1.622630
C	1.314489	-0.589483	1.709496
O	1.325324	-1.311400	2.623027
H	1.301885	-1.807451	-0.530935
H	7.179252	-1.272064	0.568157

1/0-mRSS

Frequencies, energies and thermodynamic properties:

Lowest Vibrational Mode (1/cm) =	19.4053
E(RB-P86) (a.u.) =	-4365.13534949
Thermal correction to Enthalpy (a.u.) =	0.568078
Thermal correction to Gibbs Free Energy (a.u.) =	0.460404
Total Entropy (cal/Kmol) =	226.619
E(RPBE1PBE) (a.u.) =	-4364.46576243

Optimised cartesian coordinates (Angstrom):

Fe	-2.913344	-1.558909	-0.485998
Mn	1.874796	0.167486	0.256878
P	-0.291892	0.935598	-0.037677
O	2.677083	2.805247	1.303230
O	1.412707	-0.806261	3.061536
N	1.481349	-1.843236	-0.484480
N	3.784920	-0.617316	0.324398
C	-1.329054	-0.316951	-0.881110
C	-0.953024	-1.723276	-1.011091
C	-1.797863	-2.304100	-2.032330
H	-1.786473	-3.348376	-2.366794
C	-2.700581	-1.294202	-2.509310
H	-3.484799	-1.437919	-3.264021
C	-2.426664	-0.075193	-1.802188
H	-2.936418	0.884111	-1.958366
C	-3.546994	-1.354877	1.472138
H	-3.087125	-0.719220	2.239086
C	-3.204963	-2.723533	1.192816
H	-2.445818	-3.317371	1.719144
C	-4.030995	-3.175681	0.102699
H	-4.004227	-4.171346	-0.359470
C	-4.884194	-2.082924	-0.291000
H	-5.620692	-2.100873	-1.105093

```
 C     -4.584112  -0.956392   0.555244
 H     -5.065228   0.028861   0.507018
 C     -0.356746   2.392239  -1.195129
 C     -0.301298   3.704673  -0.672475
 H     -0.294359   3.868840   0.415875
 C     -0.263448   4.813638  -1.535955
 H     -0.224560   5.829677  -1.112536
 C     -0.282132   4.627704  -2.929510
 H     -0.258857   5.497440  -3.604787
 C     -0.330620   3.324720  -3.456438
 H     -0.344594   3.168132  -4.546605
 C     -0.361482   2.212509  -2.597278
 H     -0.393965   1.201146  -3.029955
 C     -1.241446   1.607118   1.409961
 C     -0.621765   1.885956   2.645822
 H      0.446208   1.681656   2.799186
 C     -1.358819   2.452601   3.703491
 H     -0.858303   2.661472   4.661845
 C     -2.719747   2.753720   3.534328
 H     -3.295788   3.197151   4.361651
 C     -3.342801   2.493392   2.298780
 H     -4.407605   2.734270   2.153723
 C     -2.609836   1.927502   1.243929
 H     -3.111611   1.731249   0.285138
 C      0.112342  -2.448229  -0.213110
 H     -0.067040  -2.269646   0.867980
 C      2.574047  -2.697514   0.061467
 H      2.290544  -2.980471   1.100012
 H      2.692017  -3.639700  -0.509816
 C      3.879367  -1.956876   0.118545
 C      4.923348   0.105068   0.473685
 H      4.806982   1.185550   0.638588
 C      6.193104  -0.480362   0.422501
 H      7.083482   0.151232   0.553738
 C      6.298122  -1.861852   0.193802
 C      5.121452  -2.607799   0.036975
 H      5.153801  -3.692199  -0.144183
 C      0.066148  -3.963107  -0.472425
 H     -0.967860  -4.325609  -0.317433
 H      0.363570  -4.212343  -1.511851
 H      0.713758  -4.526868   0.224347
 C      2.343746   1.760688   0.887849
 C      1.556679  -0.435772   1.968597
 C      2.297927   0.729869  -1.449091
 O      2.649593   1.079442  -2.498129
 H      1.593150  -1.817350  -1.510971
 H      7.282083  -2.351062   0.136026

1/3-fRSR
Frequencies, energies and thermodynamic properties:
 Lowest Vibrational Mode (1/cm) =                          22.8984
 E(RB-P86) (a.u.) =                                      -4252.59625634
 Thermal correction to Enthalpy (a.u.) =                   0.563059
 Thermal correction to Gibbs Free Energy (a.u.) =          0.459114
 Total Entropy (cal/Kmol) =                                218.770
 E(RPBE1PBE) (a.u.) =                                    -4251.91602182
Optimised cartesian coordinates (Angstrom):
 Fe     2.929780  -0.835431   0.735619
 Mn    -1.259583  -0.397702  -1.708544
 P     -0.249528   0.662580   0.033109
 O     -2.200394   2.042618  -3.048429
 O      0.985879  -0.554665  -3.601720
 N     -0.831721  -2.300219  -0.800680
 N     -3.033888  -0.770169  -0.750561
 C      0.932578  -0.414412   0.949965
 C      1.140486  -1.837558   0.675462
 C      1.981102  -2.359052   1.730262
 H      2.342747  -3.391331   1.815027
 C      2.306289  -1.290883   2.635117
 H      2.952234  -1.371596   3.519659
 C      1.670495  -0.094932   2.158021
 H      1.729195   0.894732   2.628739
 C      3.634866  -0.315107  -1.128848
 H      3.017684  -0.151088  -2.022155
 C      4.193522  -1.574637  -0.712249
 H      4.083199  -2.534655  -1.234400
 C      4.905286  -1.361270   0.522043
```

```
 H      5.425375  -2.130390   1.108654
 C      4.789485   0.032633   0.866618
 H      5.206195   0.512036   1.762579
 C      4.002720   0.679126  -0.151080
 H      3.714543   1.738015  -0.171196
 C     -1.421347   1.220902   1.372471
 C     -2.314719   2.274376   1.057372
 H     -2.239286   2.781171   0.081087
 C     -3.292844   2.686568   1.977111
 H     -3.971089   3.515310   1.717463
 C     -3.409759   2.041992   3.223939
 H     -4.178268   2.363555   3.944723
 C     -2.540595   0.983784   3.539888
 H     -2.623525   0.471467   4.512011
 C     -1.553281   0.575553   2.621791
 H     -0.871504  -0.245286   2.893974
 C      0.714660   2.234486  -0.250642
 C      1.198531   2.534818  -1.542104
 H      0.984231   1.850811  -2.376021
 C      1.956852   3.697841  -1.771029
 H      2.326595   3.916374  -2.785644
 C      2.237571   4.579934  -0.712361
 H      2.828905   5.491946  -0.892060
 C      1.750305   4.295394   0.576995
 H      1.957348   4.984725   1.411315
 C      0.990911   3.134306   0.805215
 H      0.599846   2.935780   1.815526
 C      0.600004  -2.645227  -0.498601
 H      1.145158  -2.340148  -1.413943
 C     -1.767415  -2.547101   0.318269
 H     -1.914128  -3.628267   0.529026
 H     -1.321150  -2.100071   1.234697
 C     -3.088201  -1.873376   0.052978
 C     -4.187959  -0.078627  -0.950877
 H     -4.115619   0.806885  -1.598476
 C     -5.413964  -0.457874  -0.392431
 H     -6.310022   0.146057  -0.599203
 C     -5.473467  -1.607702   0.412337
 C     -4.285333  -2.322029   0.633140
 H     -4.275256  -3.228139   1.257832
 C      0.802256  -4.158995  -0.319487
 H      1.882884  -4.401258  -0.283354
 H      0.338170  -4.543240   0.611558
 H      0.371092  -4.710081  -1.180640
 C     -1.810415   1.066637  -2.495556
 C      0.119100  -0.468315  -2.794717
 H     -1.114473  -2.891829  -1.593884
 H     -1.921183  -1.272907  -2.854240
 H     -6.422646  -1.941937   0.858045

1/3-fRSS
Frequencies, energies and thermodynamic properties:
 Lowest Vibrational Mode (1/cm) =                          17.3251
 E(RB-P86) (a.u.) =                                     -4252.59004135
 Thermal correction to Enthalpy (a.u.) =                   0.563152
 Thermal correction to Gibbs Free Energy (a.u.) =          0.459443
 Total Entropy (cal/Kmol) =                                218.274
 E(RPBE1PBE) (a.u.) =                                   -4251.90944352
Optimised cartesian coordinates (Angstrom):
 Fe     2.870280  -0.649765  -0.332789
 Mn    -1.852793   0.917210  -0.972415
 P     -0.688670  -0.635072   0.236609
 N     -0.060851   1.428606  -2.092379
 N     -1.389130   2.733716  -0.129816
 C      0.877805  -1.177920  -0.580295
 C      1.413398  -0.621411  -1.817198
 C      2.552624  -1.425429  -2.197592
 H      3.183399  -1.257968  -3.081633
 C      2.720132  -2.475967  -1.231172
 H      3.508200  -3.240696  -1.238375
 C      1.706485  -2.315501  -0.225715
 H      1.575772  -2.953410   0.657339
 C      3.143499   0.915498   0.996613
 H      2.370428   1.572092   1.416365
 C      3.899824   1.141918  -0.208872
 H      3.809355   2.004875  -0.881430
 C      4.804432   0.035512  -0.377973
```

```
 H      5.508494  -0.101880  -1.209447
 C      4.609308  -0.871777   0.725232
 H      5.135493  -1.823746   0.876380
 C      3.579287  -0.329531   1.572950
 H      3.182207  -0.788129   2.487646
 C     -1.638247  -2.236606   0.330256
 C     -2.635245  -2.396289   1.321137
 H     -2.785398  -1.613097   2.080821
 C     -3.441312  -3.547598   1.348741
 H     -4.209350  -3.656216   2.131501
 C     -3.269050  -4.556970   0.383181
 H     -3.899120  -5.460392   0.406266
 C     -2.288032  -4.402250  -0.612381
 H     -2.146326  -5.184737  -1.375309
 C     -1.480735  -3.250038  -0.641026
 H     -0.718395  -3.143595  -1.428219
 C     -0.197929  -0.431026   2.031505
 C     -0.219073   0.867116   2.584461
 H     -0.538839   1.711051   1.955928
 C      0.150476   1.086015   3.925241
 H      0.126905   2.106698   4.339404
 C      0.537953   0.003774   4.734340
 H      0.823584   0.171081   5.785151
 C      0.546953  -1.298371   4.198228
 H      0.837677  -2.153471   4.829564
 C      0.177631  -1.515014   2.859207
 H      0.167458  -2.542863   2.464029
 C      0.851086   0.440220  -2.747315
 H      1.727091   1.014243  -3.133476
 C      0.709607   2.386119  -1.269315
 H      1.297152   1.776079  -0.548591
 H      1.439040   2.964090  -1.879654
 C     -0.206080   3.300817  -0.512209
 C     -2.231832   3.489366   0.628839
 H     -3.175431   3.010132   0.927456
 C     -1.942971   4.802531   1.014567
 H     -2.670196   5.354748   1.628304
 C     -0.733851   5.389977   0.603228
 C      0.142784   4.618396  -0.176351
 H      1.102411   5.027779  -0.526810
 C      0.171283  -0.201387  -3.969473
 H      0.914725  -0.792530  -4.539014
 H     -0.657213  -0.873262  -3.679300
 H     -0.228649   0.580120  -4.649956
 C     -2.493035  -0.276037  -2.094167
 O     -3.037758  -0.996434  -2.860038
 C     -3.317126   0.736206  -0.033794
 O     -4.328245   0.634502   0.580370
 H     -2.590131   1.856772  -2.010610
 H     -0.509670   1.954542  -2.855277
 H     -0.480507   6.424530   0.880129

1/3-mRSR
Frequencies, energies and thermodynamic properties:
 Lowest Vibrational Mode (1/cm) =                          24.0983
 E(RB-P86) (a.u.) =                                     -4252.59875015
 Thermal correction to Enthalpy (a.u.) =                   0.562853
 Thermal correction to Gibbs Free Energy (a.u.) =          0.457883
 Total Entropy (cal/Kmol) =                                220.930
 E(RPBE1PBE) (a.u.) =                                   -4251.91638217
Optimised cartesian coordinates (Angstrom):
 Fe     1.227567   2.673346  -0.429992
 Mn    -1.464317  -0.949554   0.230445
 P      0.762278  -0.820761   0.102035
 O     -1.298514  -2.851739   2.459912
 N     -1.690065   0.703635  -1.158179
 N     -3.404533  -0.464096   0.541213
 C      1.372736   0.651430  -0.817858
 C      0.598056   1.336497  -1.842075
 C      1.444452   2.342874  -2.438703
 H      1.147289   3.033296  -3.240094
 C      2.723151   2.305864  -1.781427
 H      3.572948   2.971006  -1.985503
 C      2.679357   1.277489  -0.776449
 H      3.492805   1.013311  -0.088458
 C     -0.002913   3.048295   1.186494
 H     -0.679034   2.313523   1.646488
```

C	-0.322872	3.931779	0.094188
H	-1.297290	4.023824	-0.404842
C	0.862267	4.683825	-0.230541
H	0.953371	5.431801	-1.029431
C	1.912133	4.268219	0.664756
H	2.944412	4.642891	0.664186
C	1.379399	3.253810	1.537125
H	1.930488	2.719499	2.321739
C	1.638876	-2.216319	-0.776927
C	1.466878	-3.526246	-0.268275
H	0.868184	-3.685556	0.643458
C	2.054257	-4.627592	-0.911598
H	1.916254	-5.639272	-0.497177
C	2.810582	-4.442156	-2.085430
H	3.266117	-5.307002	-2.593579
C	2.978586	-3.147180	-2.604384
H	3.568471	-2.991365	-3.522085
C	2.400240	-2.039843	-1.952979
H	2.550545	-1.030247	-2.365288
C	1.768198	-0.738346	1.677818
C	1.183913	-0.249909	2.866348
H	0.124713	0.052335	2.846344
C	1.937284	-0.155588	4.050358
H	1.463554	0.225432	4.969505
C	3.286784	-0.552490	4.064950
H	3.876088	-0.482313	4.993249
C	3.877248	-1.047611	2.888077
H	4.931905	-1.366808	2.889884
C	3.123217	-1.143683	1.704323
H	3.596564	-1.545330	0.794627
C	-0.788251	0.979362	-2.323156
H	-1.191853	1.879778	-2.844958
C	-3.115012	0.701859	-1.552430
H	-3.422348	1.660149	-2.029532
H	-3.257561	-0.102300	-2.308092
C	-3.976452	0.386576	-0.362403
C	-4.158332	-0.851078	1.606655
H	-3.673772	-1.535491	2.317882
C	-5.472677	-0.416245	1.808819
H	-6.026641	-0.771772	2.690474
C	-6.053674	0.471790	0.887059
C	-5.282296	0.878423	-0.213508
H	-5.686710	1.571752	-0.966563
C	-0.737766	-0.166691	-3.346223
H	-0.106923	0.145818	-4.202095
H	-0.298656	-1.082404	-2.905909
H	-1.740594	-0.415532	-3.747670
C	-1.361491	-2.086414	1.553230
H	-1.154868	0.197894	1.317158
H	-1.534034	1.490533	-0.509544
C	-1.717282	-2.264427	-0.967756
O	-1.849499	-3.206891	-1.672146
H	-7.081686	0.840461	1.022135

1/3-mRSS

Frequencies, energies and thermodynamic properties:

Lowest Vibrational Mode (1/cm) =	17.8544
E(RB-P86) (a.u.) =	-4252.59015481
Thermal correction to Enthalpy (a.u.) =	0.563164
Thermal correction to Gibbs Free Energy (a.u.) =	0.458931
Total Entropy (cal/Kmol) =	219.376
E(RPBE1PBE) (a.u.) =	-4251.90587994

Optimised cartesian coordinates (Angstrom):

Fe	-2.784047	-1.518296	-0.565333
Mn	1.919016	0.174156	0.443540
P	-0.135532	0.919477	-0.063624
O	2.701188	2.888360	1.249630
O	1.575345	-0.743266	3.260105
N	1.588445	-1.795713	-0.421429
N	3.828637	-0.461510	0.233353
C	-1.147814	-0.334787	-0.967046
C	-0.805839	-1.752506	-1.013213
C	-1.635273	-2.368011	-2.027781
H	-1.637991	-3.428234	-2.308976
C	-2.496102	-1.361502	-2.586371
H	-3.257997	-1.523511	-3.360566
C	-2.207185	-0.114504	-1.932615

```
 H     -2.688805   0.847698  -2.148921
 C     -3.435526  -1.180952   1.362022
 H     -2.954819  -0.523917   2.097919
 C     -3.153345  -2.577986   1.160699
 H     -2.427769  -3.177076   1.727195
 C     -3.984231  -3.049873   0.081687
 H     -3.997588  -4.069672  -0.325582
 C     -4.779998  -1.941537  -0.383747
 H     -5.505332  -1.969767  -1.207800
 C     -4.439139  -0.785568   0.406955
 H     -4.862778   0.220788   0.296804
 C     -0.097668   2.335677  -1.285006
 C     -0.068759   3.671078  -0.825863
 H     -0.160018   3.885692   0.250011
 C      0.078212   4.739586  -1.730428
 H      0.098473   5.774105  -1.351324
 C      0.196537   4.489993  -3.108694
 H      0.306196   5.326165  -3.817587
 C      0.177954   3.162041  -3.575357
 H      0.274257   2.953350  -4.653158
 C      0.040051   2.094889  -2.671000
 H      0.044161   1.061657  -3.051622
 C     -1.305148   1.632557   1.210463
 C     -0.985389   1.566368   2.582147
 H     -0.047325   1.091464   2.901555
 C     -1.863884   2.090424   3.551295
 H     -1.597549   2.027345   4.618597
 C     -3.070628   2.693747   3.159403
 H     -3.757071   3.106058   3.916100
 C     -3.395327   2.773380   1.791189
 H     -4.336457   3.250256   1.473334
 C     -2.519363   2.250091   0.825929
 H     -2.784614   2.332639  -0.239317
 C      0.251033  -2.447323  -0.173108
 H      0.040200  -2.259082   0.901340
 C      2.731619  -2.602777   0.066513
 H      2.529740  -2.847662   1.134533
 H      2.854237  -3.561898  -0.479281
 C      3.995150  -1.793888  -0.011147
 C      4.939343   0.323618   0.244980
 H      4.775241   1.391851   0.447165
 C      6.226176  -0.176147   0.015480
 H      7.083132   0.513378   0.042102
 C      6.395298  -1.545469  -0.252319
 C      5.252801  -2.361460  -0.268071
 H      5.326458  -3.440198  -0.473268
 C      0.242957  -3.966787  -0.411345
 H     -0.780257  -4.358246  -0.249202
 H      0.548346  -4.225207  -1.446688
 H      0.909085  -4.499766   0.293473
 C      2.366685   1.793678   0.935064
 C      1.688247  -0.412871   2.126459
 H      1.694221  -1.672256  -1.440296
 H      2.082309   0.652558  -1.079923
 H      7.392622  -1.968151  -0.446496

1/TSHy-fRSR-benzophenone
Frequencies, energies and thermodynamic properties:
 Lowest Vibrational Mode (1/cm) =                         -153.2601
 E(RB-P86) (a.u.) =                                       -4828.81243998
 Thermal correction to Enthalpy (a.u.) =                   0.760379
 Thermal correction to Gibbs Free Energy (a.u.) =          0.628570
 Total Entropy (cal/Kmol) =                                277.415
 E(RPBE1PBE) (a.u.) =                                     -4828.10899742
Optimised cartesian coordinates (Angstrom):
 Fe    -3.582836  -1.307214  -1.251160
 Mn     0.735820   0.074414   0.472337
 P     -1.376581   0.889931   0.523061
 O      1.373188   0.602308   3.295518
 O      0.066597  -2.695856   1.198506
 N      0.602472  -0.267718  -1.601632
 N      1.592354   1.796354  -0.232511
 C     -2.378347   0.318747  -0.912474
 C     -1.861073  -0.489153  -2.018046
 C     -2.894386  -0.542162  -3.027694
 H     -2.835984  -1.084332  -3.979688
 C     -4.035311   0.199640  -2.565154
```

H	-4.986414	0.318265	-3.101349
C	-3.726897	0.724878	-1.264578
H	-4.395434	1.335112	-0.644033
C	-3.251674	-2.808719	0.120195
H	-2.358680	-2.908553	0.750600
C	-3.413785	-3.359424	-1.199527
H	-2.671910	-3.958106	-1.745056
C	-4.710525	-2.968480	-1.690420
H	-5.127616	-3.211142	-2.676992
C	-5.351956	-2.177805	-0.671062
H	-6.344135	-1.712735	-0.745225
C	-4.450239	-2.076454	0.446897
H	-4.631861	-1.522467	1.376990
C	-1.474703	2.745782	0.389971
C	-1.038125	3.506178	1.502287
H	-0.728243	2.993930	2.428097
C	-1.002856	4.908896	1.442913
H	-0.670832	5.483987	2.322144
C	-1.386232	5.577768	0.264401
H	-1.357122	6.677935	0.216995
C	-1.804611	4.831945	-0.850818
H	-2.106412	5.345226	-1.777963
C	-1.849103	3.425517	-0.789878
H	-2.194859	2.858812	-1.668248
C	-2.482823	0.595152	1.995195
C	-2.209651	-0.463092	2.888425
H	-1.328795	-1.101043	2.730179
C	-3.059341	-0.715783	3.981305
H	-2.829858	-1.545979	4.668207
C	-4.191810	0.088517	4.199172
H	-4.855315	-0.108054	5.056344
C	-4.467524	1.152661	3.320454
H	-5.347409	1.794494	3.487242
C	-3.617557	1.407065	2.230033
H	-3.836213	2.256483	1.564091
C	-0.494005	-1.158171	-2.115591
H	-0.479532	-2.027724	-1.429234
C	0.732739	0.996387	-2.358461
H	1.172596	0.845762	-3.368222
H	-0.286527	1.415253	-2.518498
C	1.539668	1.999524	-1.580166
C	2.255018	2.713239	0.522774
H	2.288091	2.518620	1.604109
C	2.869583	3.846684	-0.018926
H	3.385803	4.551814	0.648956
C	2.816387	4.054964	-1.407489
C	2.145724	3.108642	-2.194795
H	2.078929	3.221880	-3.287410
C	-0.191270	-1.685843	-3.528500
H	-0.910430	-2.482598	-3.804092
H	-0.252835	-0.893041	-4.301699
H	0.824852	-2.128424	-3.554866
C	1.097401	0.405011	2.159352
C	0.289247	-1.570735	0.898945
H	1.503039	-0.804228	-1.736525
H	2.246965	-0.559247	0.358217
C	3.526530	-1.416410	-0.606354
C	4.573810	-0.353197	-0.317179
C	5.401312	-0.337717	0.831277
C	4.789199	0.630015	-1.312249
C	6.393342	0.646041	0.987299
C	5.779246	1.611036	-1.157722
H	4.167744	0.590183	-2.218773
C	6.584904	1.626552	-0.002884
H	7.028667	0.638745	1.887475
H	5.929988	2.366026	-1.945862
H	7.365868	2.393899	0.119997
O	3.051183	-1.480783	-1.778254
H	3.292102	4.934126	-1.868069
H	5.288461	-1.108802	1.606164
C	3.510709	-2.679855	0.240425
C	3.396169	-2.709421	1.649060
C	3.641671	-3.905436	-0.452871
C	3.442240	-3.928933	2.344695
H	3.225260	-1.773553	2.201880
C	3.690148	-5.125559	0.242473
H	3.710277	-3.882281	-1.551362

```
 C      3.596802  -5.141170   1.645840
 H      3.341672  -3.934234   3.441932
 H      3.800948  -6.069449  -0.315352
 H      3.632301  -6.096072   2.194256
```

```
1/TSHy-fRSR-re-8ke
Frequencies, energies and thermodynamic properties:
 Lowest Vibrational Mode (1/cm) =                         -246.9177
 E(RB-P86) (a.u.) =                                       -4675.30396284
 Thermal correction to Enthalpy (a.u.) =                   0.712461
 Thermal correction to Gibbs Free Energy (a.u.) =          0.589480
 Total Entropy (cal/Kmol) =                                258.836
 E(RPBE1PBE) (a.u.) =                                     -4674.61436103
Optimised cartesian coordinates (Angstrom):
 Fe    -3.642701  -1.202629  -0.855907
 Mn     0.923649  -0.272107   0.489268
 P     -1.005852   0.902787   0.354360
 O      1.742051   0.772806   3.116173
 O     -0.145069  -2.652645   1.842700
 N      0.636066  -1.031042  -1.453503
 N      2.055107   1.055236  -0.593148
 C     -2.159464   0.214858  -0.904808
 C     -1.842578  -0.899049  -1.799413
 C     -2.914803  -0.990632  -2.764157
 H     -2.995155  -1.733634  -3.567442
 C     -3.887211   0.028152  -2.476213
 H     -4.827430   0.192483  -3.019512
 C     -3.432375   0.766966  -1.331579
 H     -3.955535   1.610005  -0.862453
 C     -3.513929  -2.407541   0.811458
 H     -2.623192  -2.535971   1.440140
 C     -3.842000  -3.189705  -0.351166
 H     -3.250037  -4.020024  -0.759257
 C     -5.071688  -2.678583  -0.900845
 H     -5.577131  -3.046022  -1.803989
 C     -5.505812  -1.581159  -0.074575
 H     -6.400598  -0.965698  -0.238165
 C     -4.542489  -1.411158   0.981838
 H     -4.572702  -0.646289   1.768566
 C     -0.778427   2.665910  -0.204111
 C     -0.154503   3.563052   0.696914
 H      0.109208   3.224603   1.712494
 C      0.126029   4.884759   0.313849
 H      0.602118   5.571503   1.031984
 C     -0.196234   5.329943  -0.982804
 H      0.025199   6.366141  -1.283995
 C     -0.800680   4.442933  -1.889825
 H     -1.056879   4.780969  -2.906786
 C     -1.090588   3.119564  -1.504456
 H     -1.579224   2.443677  -2.223241
 C     -2.074701   1.155909   1.860864
 C     -1.954407   0.290429   2.969316
 H     -1.211266  -0.519270   2.954066
 C     -2.781768   0.450365   4.096232
 H     -2.673161  -0.234147   4.952588
 C     -3.738141   1.480448   4.132382
 H     -4.383826   1.607117   5.015925
 C     -3.859397   2.354866   3.036381
 H     -4.599760   3.170537   3.058328
 C     -3.031272   2.197115   1.911419
 H     -3.125107   2.902043   1.070550
 C     -0.617385  -1.804283  -1.744330
 H     -0.723778  -2.480803  -0.873328
 C      0.954171  -0.011208  -2.475531
 H      1.295481  -0.460456  -3.433242
 H      0.027710   0.562073  -2.706540
 C      1.986596   0.948940  -1.951400
 C      2.917074   1.966735  -0.068753
 H      2.954278   2.024690   1.028320
 C      3.724725   2.793935  -0.856541
 H      4.397543   3.514447  -0.368814
 C      3.663227   2.676238  -2.255153
 C      2.783743   1.731701  -2.803999
 H      2.698741   1.599293  -3.893197
 C     -0.472293  -2.688359  -2.994862
 H     -1.329522  -3.386142  -3.074850
 H     -0.429053  -2.097222  -3.932366
```

H	0.450574	-3.298172	-2.917951
C	1.403272	0.365893	2.055586
C	0.229261	-1.671363	1.288222
H	1.433365	-1.729939	-1.475670
H	2.298291	-1.193689	0.472013
C	3.325365	-2.332998	-0.236769
C	4.564131	-1.535947	0.072122
C	3.194915	-3.338696	0.933916
C	4.978893	-1.788252	1.399556
C	5.309139	-0.710314	-0.785840
C	3.998885	-2.716241	2.096123
C	6.157017	-1.198617	1.886231
C	6.490150	-0.123879	-0.296079
H	4.971137	-0.544354	-1.821418
C	6.907965	-0.365964	1.030781
H	6.499261	-1.388510	2.916972
H	7.098483	0.521528	-0.949962
H	7.838347	0.095377	1.399775
O	2.879298	-2.534239	-1.402008
H	4.501863	-3.464844	2.742535
H	3.328447	-2.120238	2.756585
H	2.146264	-3.590247	1.177167
H	3.690359	-4.271528	0.578024
H	4.290530	3.304637	-2.905597

1/TSHy-fRSR-si-8ke
Frequencies, energies and thermodynamic properties:

Lowest Vibrational Mode (1/cm) =	-245.8578
E(RB-P86) (a.u.) =	-4675.30344377
Thermal correction to Enthalpy (a.u.) =	0.712351
Thermal correction to Gibbs Free Energy (a.u.) =	0.589178
Total Entropy (cal/Kmol) =	259.239
E(RPBE1PBE) (a.u.) =	-4674.61294299

Optimised cartesian coordinates (Angstrom):

Fe	-3.109463	-1.736164	-1.058360
Mn	1.087275	0.255866	0.320028
P	-1.105335	0.773625	0.523708
O	1.855422	0.939248	3.076933
O	0.911214	-2.566229	1.128373
N	0.842909	-0.124664	-1.737341
N	1.659039	2.066156	-0.455748
C	-2.124173	0.048810	-0.827744
C	-1.585677	-0.698124	-1.965306
C	-2.676196	-0.914114	-2.889094
H	-2.615700	-1.459809	-3.838969
C	-3.871114	-0.331677	-2.342264
H	-4.868270	-0.357400	-2.801811
C	-3.539923	0.255064	-1.073901
H	-4.237846	0.776036	-0.406176
C	-2.463089	-3.151651	0.291745
H	-1.518831	-3.115623	0.850281
C	-2.648599	-3.743142	-1.007083
H	-1.874661	-4.241616	-1.606229
C	-4.021987	-3.546705	-1.395232
H	-4.476539	-3.862984	-2.343667
C	-4.686894	-2.836007	-0.332942
H	-5.737494	-2.515967	-0.330516
C	-3.723891	-2.589270	0.708615
H	-3.908925	-2.050374	1.646854
C	-1.464373	2.597366	0.391259
C	-1.045320	3.425362	1.461246
H	-0.593914	2.972913	2.359616
C	-1.205928	4.818940	1.395141
H	-0.883827	5.446012	2.241928
C	-1.773150	5.413045	0.251108
H	-1.897992	6.506241	0.198444
C	-2.177118	4.601786	-0.822922
H	-2.621940	5.056288	-1.722740
C	-2.024489	3.203172	-0.754714
H	-2.362220	2.582515	-1.599154
C	-2.046710	0.353693	2.077380
C	-1.562900	-0.642201	2.952682
H	-0.615778	-1.153742	2.730661
C	-2.285033	-0.992895	4.108416
H	-1.891621	-1.772364	4.780222
C	-3.498974	-0.349612	4.407545
H	-4.062902	-0.622561	5.313684

```
 C     -3.984836   0.652374   3.546960
 H     -4.930361   1.169063   3.777092
 C     -3.262395   1.004786   2.393690
 H     -3.645906   1.805429   1.742013
 C     -0.148520  -1.167267  -2.165100
 H      0.040330  -2.016708  -1.478781
 C      0.741734   1.137641  -2.500456
 H      1.090854   1.029688  -3.549960
 H     -0.329402   1.438547  -2.550539
 C      1.508690   2.226583  -1.802965
 C      2.268094   3.065563   0.238809
 H      2.370617   2.911561   1.322444
 C      2.755084   4.228966  -0.366731
 H      3.238892   4.998103   0.253165
 C      2.621302   4.382602  -1.757188
 C      1.988750   3.360593  -2.479874
 H      1.856164   3.433238  -3.569910
 C      0.119243  -1.665831  -3.595751
 H     -0.496798  -2.560888  -3.813423
 H     -0.113863  -0.900682  -4.364109
 H      1.183579  -1.958494  -3.700487
 C      1.525897   0.669695   1.970021
 C      0.925945  -1.428343   0.796127
 H      1.806877  -0.510369  -1.948081
 H      2.654951  -0.198344   0.056717
 C      3.933481  -0.654008  -0.950660
 C      4.317420  -1.829752  -0.091097
 C      4.848092   0.502272  -0.476976
 C      5.134665  -1.397354   0.977066
 C      4.021627  -3.188619  -0.285944
 C      5.292249   0.113121   0.950036
 H      5.721042   0.498298  -1.169913
 C      5.663008  -2.337872   1.876660
 C      4.554742  -4.128373   0.614231
 H      3.392437  -3.500420  -1.135037
 H      6.321683   0.442140   1.201770
 C      5.367604  -3.703692   1.687974
 H      6.306511  -2.019438   2.713398
 H      4.344728  -5.201891   0.481064
 H      5.782727  -4.451185   2.383325
 O      3.448360  -0.745655  -2.113663
 H      4.368617   1.495791  -0.559372
 H      4.614802   0.569610   1.707264
 H      3.002552   5.279740  -2.268172
```

1/TSHy-fRSS-benzophenone

```
Frequencies, energies and thermodynamic properties:
 Lowest Vibrational Mode (1/cm) =                          -207.0407
 E(RB-P86) (a.u.) =                                        -4828.80483173
 Thermal correction to Enthalpy (a.u.) =                    0.760271
 Thermal correction to Gibbs Free Energy (a.u.) =           0.628848
 Total Entropy (cal/Kmol) =                                 276.603
 E(RPBE1PBE) (a.u.) =                                      -4828.10206402
Optimised cartesian coordinates (Angstrom):
 Fe    -3.541251  -0.617477  -1.768289
 Mn     0.824799   0.350403   0.400018
 P     -1.383050   0.798364   0.735959
 N      0.454093  -0.519387  -1.506094
 N      0.971173  -1.639984   0.891073
 C     -2.337306   0.799716  -0.842238
 C     -1.800761   0.463737  -2.154833
 C     -2.833797   0.739569  -3.126673
 H     -2.746836   0.578827  -4.210175
 C     -3.988043   1.255553  -2.443176
 H     -4.939771   1.543945  -2.909002
 C     -3.695879   1.273236  -1.036645
 H     -4.381383   1.599567  -0.244408
 C     -3.487978  -2.490678  -0.878608
 H     -2.764964  -2.857488  -0.139092
 C     -3.384487  -2.610921  -2.310344
 H     -2.561540  -3.079352  -2.866232
 C     -4.554378  -2.005570  -2.889643
 H     -4.771565  -1.916722  -3.962438
 C     -5.381170  -1.515807  -1.814812
 H     -6.335230  -0.983936  -1.928267
 C     -4.718860  -1.810995  -0.571130
 H     -5.075804  -1.551192   0.433894
```

C	-1.622840	2.540417	1.351254
C	-1.420543	2.813876	2.724716
H	-1.199992	1.990859	3.422879
C	-1.498916	4.129524	3.211939
H	-1.344066	4.323184	4.285542
C	-1.774250	5.194802	2.333868
H	-1.837397	6.226074	2.716083
C	-1.965552	4.933571	0.965938
H	-2.177980	5.760536	0.269374
C	-1.888231	3.616134	0.476463
H	-2.038463	3.428797	-0.597653
C	-2.508731	-0.124911	1.912261
C	-2.160445	-1.429326	2.322607
H	-1.220896	-1.875081	1.968746
C	-2.997655	-2.161124	3.185621
H	-2.706660	-3.177447	3.495529
C	-4.194783	-1.594094	3.655946
H	-4.849792	-2.163826	4.334157
C	-4.546479	-0.288626	3.263810
H	-5.478001	0.168144	3.634928
C	-3.708359	0.442169	2.403734
H	-3.988984	1.469887	2.125495
C	-0.387431	0.098339	-2.579085
H	-0.494805	-0.667303	-3.384193
C	0.051089	-1.930812	-1.323678
H	-1.054605	-1.937102	-1.205912
H	0.282014	-2.542659	-2.224525
C	0.664676	-2.530275	-0.096838
C	1.441967	-2.137908	2.067998
H	1.680173	-1.400382	2.847195
C	1.608054	-3.505904	2.305710
H	1.983856	-3.841024	3.283442
C	1.301451	-4.420455	1.283942
C	0.829599	-3.916436	0.062822
H	0.576431	-4.588742	-0.770709
C	0.324932	1.300070	-3.222507
H	-0.237734	1.630791	-4.117299
H	0.404842	2.157248	-2.528857
H	1.344030	1.003752	-3.544081
C	1.084340	2.007849	-0.134677
O	1.344105	3.126836	-0.413543
C	1.336677	0.875857	1.992964
O	1.733651	1.238757	3.049381
H	2.431570	0.238641	-0.001976
H	1.447058	-0.534254	-1.872226
C	3.713600	-0.224982	-1.108866
O	3.110908	-0.552555	-2.177042
C	5.529393	1.420651	-0.249355
C	4.419125	1.128066	-1.076856
C	6.189960	2.659291	-0.340863
C	4.010204	2.106646	-2.012911
C	5.759264	3.627719	-1.263938
H	7.055547	2.861989	0.310127
C	4.665222	3.342801	-2.103841
H	3.162864	1.875485	-2.673034
H	6.277802	4.597220	-1.335217
H	4.322407	4.091125	-2.836474
H	1.429810	-5.503195	1.433808
H	5.904450	0.669868	0.460248
C	4.265043	-1.333174	-0.225604
C	4.498236	-1.205122	1.163519
C	4.610664	-2.548063	-0.860548
C	5.100192	-2.248369	1.886475
H	4.182626	-0.289097	1.686256
C	5.209202	-3.593647	-0.137121
H	4.409856	-2.648330	-1.938319
C	5.465270	-3.443544	1.238053
H	5.274532	-2.132723	2.968236
H	5.482089	-4.529387	-0.651347
H	5.938935	-4.259627	1.806838

1/TSHy-fRSS-re-8ke

Frequencies, energies and thermodynamic properties:

Lowest Vibrational Mode (1/cm) =	-263.4461
E(RB-P86) (a.u.) =	-4675.29661526
Thermal correction to Enthalpy (a.u.) =	0.712347
Thermal correction to Gibbs Free Energy (a.u.) =	0.589365

Total Entropy (cal/Kmol) = 258.838
E(RPBE1PBE) (a.u.) = -4674.60633753
Optimised cartesian coordinates (Angstrom):

Fe	-3.383527	-0.575450	-1.608485
Mn	1.152692	0.011559	0.359497
P	-0.905701	0.970477	0.499210
N	0.543694	-1.251938	-1.236420
N	0.941839	-1.762254	1.376688
C	-1.884162	0.747022	-1.048465
C	-1.463808	-0.031762	-2.206702
C	-2.435613	0.190042	-3.252380
H	-2.409891	-0.266505	-4.251496
C	-3.436343	1.101883	-2.769494
H	-4.314612	1.452612	-3.327700
C	-3.113936	1.429927	-1.408388
H	-3.694411	2.094372	-0.756039
C	-3.724898	-2.087068	-0.230906
H	-3.095736	-2.363106	0.625076
C	-3.644846	-2.629536	-1.563119
H	-2.937036	-3.392183	-1.913793
C	-4.660922	-1.994588	-2.360238
H	-4.851966	-2.175073	-3.426481
C	-5.370283	-1.063409	-1.518652
H	-6.192290	-0.407550	-1.835066
C	-4.788265	-1.117187	-0.202850
H	-5.087575	-0.515898	0.665258
C	-0.770906	2.824310	0.620450
C	-0.508516	3.412845	1.880048
H	-0.465085	2.783584	2.783066
C	-0.302794	4.798314	1.993998
H	-0.105150	5.240179	2.983858
C	-0.349710	5.617886	0.850577
H	-0.190551	6.704264	0.940216
C	-0.598522	5.039930	-0.406634
H	-0.633847	5.671912	-1.308593
C	-0.805265	3.652648	-0.522469
H	-0.997086	3.215372	-1.514191
C	-2.150152	0.637092	1.855404
C	-2.021657	-0.532422	2.634255
H	-1.181201	-1.215024	2.447807
C	-2.950356	-0.827732	3.650036
H	-2.831503	-1.745174	4.248215
C	-4.018863	0.049110	3.905021
H	-4.744880	-0.177873	4.701890
C	-4.149537	1.226061	3.143539
H	-4.977790	1.924674	3.343614
C	-3.220460	1.520785	2.130633
H	-3.325307	2.457764	1.561780
C	-0.170955	-0.787007	-2.464533
H	-0.452977	-1.702326	-3.037979
C	-0.140568	-2.434759	-0.671737
H	-1.209848	-2.160808	-0.536162
H	-0.114854	-3.298545	-1.373448
C	0.434721	-2.807806	0.659534
C	1.397919	-2.018590	2.635382
H	1.793825	-1.157834	3.193303
C	1.372358	-3.292857	3.211006
H	1.753993	-3.430068	4.233419
C	0.866064	-4.371559	2.464356
C	0.395156	-4.117620	1.167661
H	-0.010303	-4.926410	0.541481
C	0.772413	0.021714	-3.374027
H	0.312879	0.142124	-4.374762
H	0.980439	1.028596	-2.967708
H	1.734783	-0.517889	-3.489264
C	1.742705	1.358757	-0.608686
O	2.241584	2.261686	-1.185729
C	1.801284	0.842738	1.762025
O	2.280353	1.397618	2.693790
H	2.673221	-0.557152	0.010468
H	1.497995	-1.583718	-1.557056
C	3.751127	-1.503939	-0.845012
C	4.246732	-2.275411	0.403634
C	5.120447	-1.271640	1.185240
O	3.088844	-2.033807	-1.783727
C	5.561433	-0.279110	0.123693
C	4.745983	-0.385227	-1.025727

```
 C      6.604152   0.661194   0.158288
 C      4.958632   0.429068  -2.150108
 C      6.820263   1.482726  -0.966933
 H      7.251902   0.752939   1.045772
 C      6.005638   1.367564  -2.114150
 H      4.319253   0.323955  -3.040930
 H      7.639977   2.219268  -0.954090
 H      6.197386   2.013761  -2.985878
 H      4.514628  -0.743834   1.956786
 H      5.974391  -1.741725   1.715498
 H      3.426218  -2.731763   0.988213
 H      4.872362  -3.109120   0.009310
 H      0.841458  -5.389158   2.882686
```

```
1/TSHy-fRSS-si-8ke
Frequencies, energies and thermodynamic properties:
 Lowest Vibrational Mode (1/cm) =                           -255.9195
 E(RB-P86) (a.u.) =                                         -4675.29762747
 Thermal correction to Enthalpy (a.u.) =                     0.712422
 Thermal correction to Gibbs Free Energy (a.u.) =            0.589381
 Total Entropy (cal/Kmol) =                                  258.961
 E(RPBE1PBE) (a.u.) =                                       -4674.60806072
Optimised cartesian coordinates (Angstrom):
 Fe    -3.375313  -1.385624  -1.076794
 Mn     1.096516   0.599099  -0.111191
 P     -1.090296   0.977409   0.398492
 N      0.585350  -0.961206  -1.457109
 N      1.599928  -1.038505   1.029350
 C     -2.227284   0.336031  -0.906152
 C     -1.819330  -0.428543  -2.078468
 C     -2.983637  -0.585279  -2.919135
 H     -3.009657  -1.126507  -3.875007
 C     -4.096312   0.080633  -2.298930
 H     -5.121794   0.125971  -2.689130
 C     -3.641951   0.629704  -1.051398
 H     -4.259135   1.187587  -0.336004
 C     -3.041576  -2.763036   0.436248
 H     -2.226356  -2.759476   1.171288
 C     -3.034655  -3.415568  -0.848210
 H     -2.209462  -3.998643  -1.277783
 C     -4.306506  -3.170123  -1.475232
 H     -4.613551  -3.517681  -2.470655
 C     -5.099580  -2.369731  -0.575763
 H     -6.114008  -1.996981  -0.770580
 C     -4.315406  -2.113999   0.604404
 H     -4.623331  -1.517544   1.472807
 C     -1.470728   2.801111   0.395437
 C     -1.170579   3.567063   1.546488
 H     -0.791518   3.071187   2.454044
 C     -1.352868   4.960529   1.546846
 H     -1.118812   5.540299   2.454157
 C     -1.832891   5.612533   0.395448
 H     -1.978033   6.704561   0.396540
 C     -2.123762   4.860574  -0.756287
 H     -2.497143   5.361324  -1.664043
 C     -1.941916   3.464993  -0.758267
 H     -2.172226   2.891085  -1.668797
 C     -1.921752   0.470687   1.996288
 C     -1.315239  -0.511788   2.807312
 H     -0.354627  -0.947929   2.500145
 C     -1.920893  -0.929945   4.007431
 H     -1.430591  -1.697209   4.627528
 C     -3.140637  -0.363855   4.416256
 H     -3.614551  -0.686828   5.356850
 C     -3.747974   0.627752   3.622385
 H     -4.698510   1.085202   3.940685
 C     -3.141531   1.045454   2.425134
 H     -3.618899   1.839002   1.829514
 C     -0.434259  -0.845704  -2.543903
 H     -0.546634  -1.863689  -2.988260
 C      0.361363  -2.202015  -0.684730
 H     -0.701511  -2.196951  -0.357239
 H      0.498264  -3.108860  -1.315981
 C      1.243000  -2.255010   0.525149
 C      2.340652  -1.016396   2.171683
 H      2.623209  -0.022912   2.548268
 C      2.722405  -2.175594   2.854401
```

```
 H      3.311110  -2.087296   3.779163
 C      2.351143  -3.428434   2.336425
 C      1.610127  -3.461563   1.146071
 H      1.300781  -4.416202   0.694538
 C      0.071993   0.068841  -3.674854
 H     -0.569801  -0.054066  -4.569415
 H      0.057082   1.135540  -3.384849
 H      1.111462  -0.208986  -3.945411
 C      1.082656   1.934272  -1.253227
 O      1.194290   2.866365  -1.975503
 C      1.722419   1.735568   1.071303
 O      2.177176   2.512703   1.841973
 H      2.625367   0.396318  -0.717550
 H      1.520376  -1.073268  -1.942545
 C      3.738365  -0.277392  -1.780395
 C      4.737114  -0.584494  -0.696326
 C      4.183228   1.090663  -2.354680
 C      5.507008   0.566613  -0.412273
 C      4.999775  -1.814976  -0.072067
 C      5.025951   1.746207  -1.238916
 H      4.826945   0.847239  -3.231329
 C      6.552567   0.493877   0.522484
 C      6.050272  -1.885219   0.860782
 H      4.398054  -2.702137  -0.326793
 H      5.860075   2.371549  -1.619029
 C      6.817682  -0.737756   1.156519
 H      7.167280   1.379991   0.751689
 H      6.283342  -2.840098   1.358766
 H      7.641467  -0.808229   1.885227
 H      3.340823   1.706076  -2.719638
 H      4.390078   2.408898  -0.608624
 O      3.110843  -1.155835  -2.439713
 H      2.639970  -4.360742   2.845065
```

```
1/TSHy-mRSR-benzophenone
Frequencies, energies and thermodynamic properties:
 Lowest Vibrational Mode (1/cm) =                            -276.1902
 E(RB-P86) (a.u.) =                                          -4828.79367941
 Thermal correction to Enthalpy (a.u.) =                      0.760081
 Thermal correction to Gibbs Free Energy (a.u.) =             0.629375
 Total Entropy (cal/Kmol) =                                   275.095
 E(RPBE1PBE) (a.u.) =                                        -4828.09456674
Optimised cartesian coordinates (Angstrom):
 Fe    -2.774299  -0.729144  -2.301970
 Mn     0.664442   1.227726   0.513932
 P     -1.503436   0.549196   0.786745
 O      1.359728   0.757679   3.324252
 N      0.486225   0.960974  -1.603966
 N      2.425712   2.066327  -0.056359
 C     -2.463380   0.633776  -0.781127
 C     -1.964378   1.134626  -2.054780
 C     -3.076369   1.174824  -2.976225
 H     -3.022292   1.494434  -4.026124
 C     -4.248898   0.692193  -2.298863
 H     -5.247275   0.575015  -2.741095
 C     -3.873904   0.339541  -0.957346
 H     -4.541758  -0.075501  -0.191776
 C     -1.434870  -2.306339  -2.270851
 H     -0.468105  -2.341320  -1.745578
 C     -1.632000  -1.832506  -3.616801
 H     -0.852074  -1.439358  -4.282950
 C     -3.032045  -1.948483  -3.932863
 H     -3.509240  -1.649736  -4.875927
 C     -3.702415  -2.498404  -2.781747
 H     -4.780144  -2.691726  -2.695742
 C     -2.717754  -2.715663  -1.755152
 H     -2.909224  -3.108612  -0.748539
 C     -2.450152   1.710403   1.897755
 C     -2.209370   1.658683   3.291132
 H     -1.523783   0.901564   3.704355
 C     -2.838942   2.565808   4.159868
 H     -2.644529   2.507937   5.242898
 C     -3.713763   3.543953   3.649763
 H     -4.207755   4.254978   4.330955
 C     -3.950371   3.608528   2.265785
 H     -4.630694   4.372420   1.856116
 C     -3.321724   2.699058   1.394168
```

```
 H     -3.515957   2.763499   0.312621
 C     -2.044642  -1.087662   1.494262
 C     -1.173009  -2.186791   1.379550
 H     -0.184187  -2.040171   0.922678
 C     -1.554657  -3.457229   1.848395
 H     -0.855377  -4.302421   1.751323
 C     -2.815918  -3.638657   2.443243
 H     -3.116001  -4.631480   2.814943
 C     -3.690807  -2.543083   2.570850
 H     -4.677863  -2.675393   3.042462
 C     -3.307454  -1.273358   2.104061
 H     -3.995775  -0.422546   2.226024
 C     -0.584197   1.633957  -2.412084
 H     -0.415770   1.336110  -3.474127
 C      1.833425   1.148219  -2.214630
 H      2.296395   0.139353  -2.266366
 H      1.759635   1.520242  -3.258595
 C      2.716073   2.037115  -1.386731
 C      3.235722   2.785677   0.764068
 H      2.973077   2.777668   1.832026
 C      4.338187   3.509035   0.298249
 H      4.953569   4.075809   1.012271
 C      4.628309   3.498741  -1.076790
 C      3.802689   2.747104  -1.925762
 H      3.990267   2.704829  -3.009324
 C     -0.494913   3.167273  -2.357791
 H     -1.206663   3.598908  -3.089355
 H     -0.747903   3.559065  -1.355302
 H      0.519571   3.525511  -2.628440
 C      1.056022   0.966344   2.197060
 H      1.480016  -0.310083   0.462234
 H      0.304005  -0.058596  -1.655574
 C      0.106017   2.878145   0.833987
 O     -0.140963   3.991679   1.146438
 C      2.312706  -1.676958  -0.306331
 C      3.725553  -1.088429  -0.390948
 C      4.405568  -0.446109   0.667499
 C      4.425396  -1.319673  -1.598999
 C      5.745810  -0.050967   0.522426
 C      5.767756  -0.929779  -1.743410
 H      3.898415  -1.831958  -2.419008
 C      6.434887  -0.294677  -0.680863
 H      6.256223   0.456305   1.356565
 H      6.296800  -1.129996  -2.689161
 H      7.487929   0.010644  -0.788393
 O      1.677552  -1.860822  -1.382990
 H      5.480977   4.065769  -1.480367
 H      3.880660  -0.230631   1.608465
 C      2.159096  -2.714095   0.812845
 C      2.305857  -2.464857   2.196082
 C      1.954380  -4.047360   0.385681
 C      2.262387  -3.521699   3.121142
 H      2.412363  -1.435931   2.566052
 C      1.922830  -5.105854   1.310453
 H      1.833713  -4.241089  -0.691254
 C      2.082121  -4.847473   2.683565
 H      2.364936  -3.305615   4.196719
 H      1.775335  -6.138214   0.954232
 H      2.057490  -5.673831   3.411703
```

```
1/TSHy-mRSR-re-8ke
Frequencies, energies and thermodynamic properties:
 Lowest Vibrational Mode (1/cm) =                          -416.0531
 E(RB-P86) (a.u.) =                                        -4675.28571112
 Thermal correction to Enthalpy (a.u.) =                    0.712179
 Thermal correction to Gibbs Free Energy (a.u.) =           0.590224
 Total Entropy (cal/Kmol) =                                 256.676
 E(RPBE1PBE) (a.u.) =                                      -4674.59834075
Optimised cartesian coordinates (Angstrom):
 Fe    -2.601336  -2.146659  -1.138894
 Mn     0.884628   1.079619  -0.126791
 P     -1.326157   0.844307   0.435113
 O      1.516000   2.044307   2.575710
 N      0.651092  -0.081934  -1.899868
 N      2.763954   1.260503  -0.862818
 C     -2.325377  -0.134047  -0.755227
 C     -1.852960  -0.481820  -2.087039
```

C	-2.970844	-1.040349	-2.814097
H	-2.935537	-1.404927	-3.850188
C	-4.120754	-1.050788	-1.951932
H	-5.115113	-1.442043	-2.205722
C	-3.725561	-0.513566	-0.678640
H	-4.375753	-0.410603	0.198586
C	-1.117392	-3.514671	-0.701402
H	-0.035916	-3.305733	-0.742047
C	-1.934976	-3.976236	-1.793361
H	-1.586538	-4.203967	-2.809767
C	-3.296726	-4.064753	-1.330189
H	-4.166156	-4.365476	-1.930085
C	-3.322833	-3.658401	0.052185
H	-4.214575	-3.597096	0.690102
C	-1.979134	-3.319276	0.438336
H	-1.674645	-2.953250	1.427673
C	-2.232303	2.481244	0.431035
C	-1.923859	3.417565	1.446251
H	-1.204155	3.153298	2.238114
C	-2.527714	4.685361	1.458027
H	-2.279805	5.400053	2.259235
C	-3.444885	5.042730	0.450785
H	-3.918059	6.037516	0.459578
C	-3.751941	4.121683	-0.564959
H	-4.468945	4.390518	-1.357341
C	-3.150624	2.847731	-0.575562
H	-3.408926	2.134596	-1.373043
C	-1.887268	0.229395	2.107497
C	-0.969066	-0.412960	2.959743
H	0.071842	-0.522045	2.627400
C	-1.365846	-0.890137	4.223044
H	-0.630365	-1.387240	4.875529
C	-2.693654	-0.723321	4.651925
H	-3.008392	-1.094278	5.640349
C	-3.616105	-0.062511	3.818278
H	-4.654516	0.089209	4.153891
C	-3.214949	0.418618	2.560228
H	-3.939963	0.965529	1.937670
C	-0.572381	-0.063943	-2.774641
H	-0.396531	-0.802533	-3.592378
C	1.866234	0.015794	-2.760038
H	2.208281	-1.003741	-3.040377
H	1.616309	0.535584	-3.706858
C	2.995434	0.762974	-2.106170
C	3.764862	1.951572	-0.251343
H	3.547170	2.326738	0.758549
C	5.001041	2.193248	-0.856014
H	5.765386	2.766729	-0.311438
C	5.235843	1.697697	-2.150731
C	4.218085	0.965101	-2.773897
H	4.354009	0.549853	-3.784296
C	-0.808829	1.312767	-3.429651
H	-1.637771	1.230634	-4.159822
H	-1.087899	2.070110	-2.672174
H	0.079606	1.685815	-3.977431
C	1.252044	1.665558	1.483353
H	1.483577	-0.425331	0.530524
H	0.729803	-1.039266	-1.471616
C	0.561756	2.710854	-0.742729
O	0.461970	3.829973	-1.106445
C	2.169576	-1.944365	0.323653
C	3.600320	-1.601148	0.665689
C	1.596096	-2.506185	1.651860
C	3.774061	-1.591501	2.068085
C	4.695400	-1.453155	-0.200702
C	2.457574	-1.874918	2.766590
H	0.507538	-2.354423	1.758135
C	5.048857	-1.382212	2.618515
C	5.973672	-1.251447	0.351196
H	4.551567	-1.518665	-1.290027
H	2.576241	-2.522836	3.659226
C	6.146155	-1.205801	1.750913
H	5.197245	-1.376969	3.710977
H	6.847659	-1.139649	-0.310308
H	7.153317	-1.050899	2.170675
H	2.011801	-0.917788	3.122261
H	1.776986	-3.604994	1.612016

```
 O      1.805357  -2.333548  -0.827303
 H      6.194502   1.875529  -2.661327

1/TSHy-mRSR-si-8ke
Frequencies, energies and thermodynamic properties:
 Lowest Vibrational Mode (1/cm) =                        -446.5334
 E(RB-P86) (a.u.) =                                      -4675.28553779
 Thermal correction to Enthalpy (a.u.) =                  0.712067
 Thermal correction to Gibbs Free Energy (a.u.) =         0.590480
 Total Entropy (cal/Kmol) =                               255.901
 E(RPBE1PBE) (a.u.) =                                    -4674.60204138
Optimised cartesian coordinates (Angstrom):
 Fe     2.732004   0.617435  -1.980443
 Mn    -1.261080  -0.997538   0.267127
 P      0.940750  -0.728101   0.823219
 O     -2.117142  -0.360743   3.004488
 N     -0.830698  -0.844945  -1.816571
 N     -3.092406  -1.504240  -0.458544
 C      2.083118  -0.781891  -0.613560
 C      1.673415  -1.142246  -1.964079
 C      2.870347  -1.264012  -2.764922
 H      2.896071  -1.515433  -3.834292
 C      4.009070  -0.979882  -1.935501
 H      5.057552  -0.963286  -2.261844
 C      3.531014  -0.665717  -0.617372
 H      4.157526  -0.385816   0.238339
 C      1.517141   2.238357  -2.376776
 H      0.416400   2.252108  -2.314579
 C      2.305332   1.897407  -3.533084
 H      1.915462   1.605088  -4.517452
 C      3.697021   1.982203  -3.169775
 H      4.551040   1.760205  -3.823729
 C      3.771903   2.380294  -1.786810
 H      4.692436   2.514339  -1.203137
 C      2.428126   2.535927  -1.297854
 H      2.145789   2.805594  -0.271778
 C      1.568398  -2.156098   1.853482
 C      1.176166  -2.235991   3.210419
 H      0.566615  -1.433175   3.655599
 C      1.557664  -3.332098   4.001889
 H      1.247754  -3.375818   5.058393
 C      2.332345  -4.369710   3.448850
 H      2.632245  -5.228851   4.069753
 C      2.719249  -4.302063   2.099437
 H      3.324640  -5.109181   1.656416
 C      2.339158  -3.203114   1.305370
 H      2.652020  -3.163837   0.250738
 C      1.558982   0.680294   1.874843
 C      0.734951   1.804676   2.074826
 H     -0.255321   1.836859   1.596179
 C      1.168040   2.873534   2.882728
 H      0.508717   3.743232   3.028144
 C      2.430816   2.828726   3.497378
 H      2.768554   3.664114   4.131346
 C      3.258409   1.705122   3.309521
 H      4.245546   1.656499   3.796587
 C      2.822459   0.634133   2.511725
 H      3.465214  -0.253585   2.405795
 C      0.313337  -1.555177  -2.479272
 H      0.291006  -1.264466  -3.556369
 C     -2.086793  -1.011715  -2.611667
 H     -2.385177   0.011724  -2.934629
 H     -1.910883  -1.606309  -3.531392
 C     -3.215411  -1.594612  -1.812570
 C     -4.134440  -1.919990   0.312376
 H     -3.998998  -1.827080   1.399643
 C     -5.315125  -2.441053  -0.224967
 H     -6.122685  -2.758056   0.451119
 C     -5.437596  -2.553667  -1.621307
 C     -4.369688  -2.121011  -2.419934
 H     -4.420461  -2.181672  -3.517641
 C      0.161406  -3.085506  -2.412223
 H      0.933494  -3.560734  -3.049088
 H      0.287252  -3.458128  -1.377906
 H     -0.829917  -3.417874  -2.782203
 C     -1.761533  -0.634246   1.908012
 H     -1.695876   0.722957   0.055059
```

```
 H     -0.646546   0.183174  -1.875754
 C     -1.087045  -2.722022   0.619411
 O     -1.098064  -3.866897   0.909875
 C     -2.212407   1.958791  -0.869517
 C     -1.957211   3.104740   0.093579
 C     -3.733144   1.685787  -0.688334
 C     -3.034420   3.224566   1.001533
 C     -0.926127   4.058396   0.065691
 C     -4.059767   2.135720   0.749314
 C     -3.066061   4.284970   1.921937
 C     -0.967533   5.129739   0.977506
 H     -0.113635   3.977273  -0.672768
 H     -3.908316   1.296125   1.464881
 C     -2.025142   5.235770   1.905599
 H     -3.904337   4.390690   2.630228
 H     -0.175498   5.895514   0.962441
 H     -2.047659   6.081582   2.611672
 H     -5.103063   2.489374   0.881450
 O     -1.609485   1.849392  -1.984256
 H     -4.232976   2.364369  -1.417753
 H     -4.043551   0.655538  -0.928527
 H     -6.348095  -2.970116  -2.078362
```

1/TSHy-mRSS-benzophenone

```
Frequencies, energies and thermodynamic properties:
 Lowest Vibrational Mode (1/cm) =                        -431.3341
 E(RB-P86) (a.u.) =                                      -4828.79326717
 Thermal correction to Enthalpy (a.u.) =                  0.759983
 Thermal correction to Gibbs Free Energy (a.u.) =         0.630192
 Total Entropy (cal/Kmol) =                               273.168
 E(RPBE1PBE) (a.u.) =                                    -4828.09643453
Optimised cartesian coordinates (Angstrom):
 Fe    -3.633763  -0.113891  -1.555654
 Mn     0.880156  -1.136667   0.703282
 P     -0.983247   0.122361   1.011678
 O      1.866012  -0.258037   3.333071
 O     -0.303749  -3.654535   1.694858
 N      0.496406  -1.637658  -1.328313
 N      2.588468  -2.146273   0.269504
 C     -1.885230   0.413387  -0.570692
 C     -1.615725  -0.374504  -1.770533
 C     -2.165068   0.348171  -2.897588
 H     -2.143948   0.028138  -3.946369
 C     -2.796509   1.544150  -2.411817
 H     -3.327899   2.285532  -3.023158
 C     -2.640680   1.581447  -0.984288
 H     -3.002654   2.377883  -0.321520
 C     -4.803435  -1.378634  -0.412882
 H     -4.542817  -1.791672   0.569382
 C     -4.506940  -1.976978  -1.686649
 H     -3.989221  -2.932624  -1.843769
 C     -5.004948  -1.101522  -2.717106
 H     -4.922438  -1.264692  -3.799959
 C     -5.610315   0.039188  -2.077088
 H     -6.070862   0.895638  -2.587216
 C     -5.484554  -0.131029  -0.652204
 H     -5.850098   0.567447   0.111646
 C     -0.697133   1.848624   1.685436
 C     -0.656722   2.032573   3.088815
 H     -0.837650   1.181655   3.762369
 C     -0.398577   3.297202   3.644479
 H     -0.375029   3.414415   4.739886
 C     -0.183895   4.407305   2.808079
 H      0.005243   5.402430   3.241379
 C     -0.209804   4.233424   1.413917
 H     -0.036009   5.090028   0.744358
 C     -0.451237   2.963933   0.855152
 H     -0.447797   2.851935  -0.238505
 C     -2.262104  -0.408840   2.270973
 C     -1.929984  -1.301366   3.312072
 H     -0.926782  -1.747076   3.356836
 C     -2.869196  -1.620347   4.312223
 H     -2.590775  -2.321289   5.115318
 C     -4.150504  -1.045021   4.289286
 H     -4.885440  -1.294772   5.071053
 C     -4.486212  -0.140010   3.264309
 H     -5.484927   0.324839   3.241972
```

C	-3.548340	0.177769	2.267822
H	-3.823486	0.886522	1.473062
C	-0.922929	-1.726868	-1.826490
H	-1.434985	-2.404993	-1.109726
C	1.244739	-2.890054	-1.595161
H	0.637262	-3.745909	-1.220520
H	1.414477	-3.062165	-2.678723
C	2.559192	-2.887784	-0.870839
C	3.713282	-2.185369	1.031074
H	3.704293	-1.575968	1.944844
C	4.828884	-2.959417	0.694896
H	5.705915	-2.957517	1.358291
C	4.804487	-3.716603	-0.488579
C	3.650568	-3.670620	-1.283818
H	3.579092	-4.246775	-2.218670
C	-1.036243	-2.342368	-3.234151
H	-2.093474	-2.329383	-3.561536
H	-0.439688	-1.775420	-3.978704
H	-0.705148	-3.397753	-3.253648
C	1.453113	-0.603649	2.277681
C	0.131463	-2.623564	1.312710
H	1.025648	-0.891938	-1.857428
H	1.633186	0.275176	-0.020831
C	2.635813	0.790563	-1.324231
C	2.045722	2.190177	-1.525037
C	2.600779	3.364344	-0.967232
C	0.990370	2.326641	-2.457139
C	2.112149	4.633526	-1.328466
C	0.491289	3.589851	-2.806962
H	0.576401	1.418089	-2.918382
C	1.054113	4.753357	-2.245556
H	2.569329	5.535681	-0.891277
H	-0.334780	3.669868	-3.531585
H	0.674220	5.747775	-2.529615
O	2.437067	-0.041806	-2.264886
H	5.668038	-4.330772	-0.786376
H	3.442497	3.296760	-0.263313
C	3.958326	0.714379	-0.569347
C	4.139153	1.145436	0.763609
C	5.077938	0.238091	-1.285438
C	5.412112	1.117323	1.357981
H	3.269272	1.480001	1.349308
C	6.352635	0.210316	-0.693021
H	4.929208	-0.108177	-2.319909
C	6.525405	0.654852	0.629930
H	5.534978	1.450873	2.401026
H	7.216212	-0.160137	-1.268863
H	7.523048	0.634435	1.097173

1/TSHy-mRSS-re-8ke
Frequencies, energies and thermodynamic properties:

Lowest Vibrational Mode (1/cm) =	-469.7872
E(RB-P86) (a.u.) =	-4675.28293429
Thermal correction to Enthalpy (a.u.) =	0.712146
Thermal correction to Gibbs Free Energy (a.u.) =	0.590084
Total Entropy (cal/Kmol) =	256.902
E(RPBE1PBE) (a.u.) =	-4674.59624415

Optimised cartesian coordinates (Angstrom):

Fe	-3.236964	-0.253782	-1.612963
Mn	1.282723	-1.046818	0.777123
P	-0.621342	0.182161	0.986364
O	2.309464	0.169018	3.253525
O	0.198896	-3.480892	2.049028
N	0.892699	-1.741977	-1.192781
N	2.998276	-2.077360	0.433593
C	-1.509731	0.341547	-0.623090
C	-1.215090	-0.533185	-1.754451
C	-1.730911	0.106206	-2.945514
H	-1.684653	-0.290461	-3.967043
C	-2.371562	1.335300	-2.564797
H	-2.885018	2.030471	-3.242258
C	-2.252999	1.476223	-1.139810
H	-2.630665	2.317964	-0.545547
C	-4.445583	-1.440045	-0.424899
H	-4.215714	-1.793737	0.587747
C	-4.119491	-2.118168	-1.650177
H	-3.605065	-3.084818	-1.732799

C	-4.583786	-1.305999	-2.746075
H	-4.473458	-1.536647	-3.814064
C	-5.198285	-0.124532	-2.194845
H	-5.639287	0.700778	-2.769468
C	-5.111581	-0.206149	-0.759374
H	-5.493841	0.540154	-0.050709
C	-0.394140	1.959179	1.541477
C	-0.408843	2.251745	2.926574
H	-0.594437	1.451599	3.658430
C	-0.201191	3.561878	3.390637
H	-0.220398	3.763413	4.473786
C	0.016220	4.609707	2.478431
H	0.164197	5.640180	2.839009
C	0.047013	4.327960	1.102055
H	0.225391	5.134213	0.374028
C	-0.141387	3.013043	0.636184
H	-0.090777	2.818300	-0.444635
C	-1.895955	-0.300144	2.268224
C	-1.535174	-1.064155	3.398655
H	-0.509888	-1.445473	3.502447
C	-2.474323	-1.333043	4.413179
H	-2.173660	-1.933418	5.286628
C	-3.784328	-0.835022	4.315159
H	-4.519259	-1.046055	5.108247
C	-4.148967	-0.056683	3.200212
H	-5.170560	0.347823	3.118167
C	-3.210918	0.211745	2.189151
H	-3.506322	0.821968	1.323111
C	-0.522120	-1.884458	-1.688177
H	-1.040712	-2.498836	-0.920099
C	1.656908	-3.002305	-1.349114
H	1.071260	-3.829958	-0.885907
H	1.818191	-3.273934	-2.413370
C	2.979100	-2.906717	-0.645687
C	4.127933	-2.038488	1.190958
H	4.104569	-1.373788	2.066480
C	5.264576	-2.799812	0.898381
H	6.145962	-2.726291	1.552371
C	5.254374	-3.636362	-0.231247
C	4.090201	-3.685736	-1.011074
H	4.027985	-4.331179	-1.900176
C	-0.620648	-2.618074	-3.039301
H	-1.673009	-2.627726	-3.381730
H	-0.010754	-2.119125	-3.820807
H	-0.295088	-3.672731	-2.963940
C	1.876422	-0.318076	2.264311
C	0.588343	-2.480729	1.553689
H	1.419867	-1.041002	-1.789756
H	2.003312	0.298857	-0.111703
C	2.935165	0.694887	-1.407044
C	2.399436	2.088951	-1.656316
C	4.244234	0.933716	-0.605470
C	3.111065	3.030467	-0.881769
C	1.470654	2.493545	-2.628624
C	4.078972	2.322005	0.048242
C	2.891131	4.404214	-1.074606
C	1.245816	3.870212	-2.815253
H	0.948740	1.739557	-3.239773
H	5.034244	2.871485	0.177591
C	1.954946	4.817275	-2.044482
H	3.449614	5.152377	-0.488222
H	0.528115	4.214384	-3.577321
H	1.782973	5.892865	-2.212400
H	3.625205	2.222506	1.061049
O	2.797904	-0.235017	-2.261122
H	4.485934	0.117937	0.098025
H	5.055358	0.964875	-1.368614
H	6.134851	-4.241219	-0.496351

1/TSHy-mRSS-si-8ke

Frequencies, energies and thermodynamic properties:

Lowest Vibrational Mode (1/cm) =	-373.5563
E(RB-P86) (a.u.) =	-4675.28490902
Thermal correction to Enthalpy (a.u.) =	0.712009
Thermal correction to Gibbs Free Energy (a.u.) =	0.590534
Total Entropy (cal/Kmol) =	255.666
E(RPBE1PBE) (a.u.) =	-4674.59802484

Optimised cartesian coordinates (Angstrom):

Fe	-3.350474	-0.837418	-1.580724
Mn	1.129771	-0.183730	1.051013
P	-0.880915	0.803925	0.590237
O	2.457904	2.403463	1.435704
O	0.679747	-0.421886	3.958232
N	0.569173	-2.013727	0.117106
N	2.802326	-1.287807	1.348737
C	-1.593697	0.070394	-0.947684
C	-1.391376	-1.344460	-1.226516
C	-1.742253	-1.575308	-2.609234
H	-1.709017	-2.537176	-3.135782
C	-2.188084	-0.333098	-3.177994
H	-2.552211	-0.187021	-4.203794
C	-2.120640	0.678328	-2.154984
H	-2.424181	1.725218	-2.273559
C	-4.731010	-1.042089	-0.054104
H	-4.540784	-0.904145	1.017013
C	-4.601125	-2.274794	-0.782791
H	-4.303052	-3.243689	-0.360879
C	-4.928143	-2.014610	-2.161672
H	-4.907155	-2.746604	-2.980123
C	-5.264288	-0.618389	-2.282787
H	-5.542872	-0.100792	-3.210477
C	-5.138458	-0.016318	-0.980315
H	-5.314792	1.039682	-0.737997
C	-0.790178	2.646521	0.344886
C	-0.772229	3.458478	1.505551
H	-0.911094	3.001974	2.498397
C	-0.580350	4.846511	1.412919
H	-0.576391	5.456707	2.330218
C	-0.392020	5.454124	0.157840
H	-0.245592	6.543311	0.083702
C	-0.382475	4.656597	-0.998690
H	-0.224599	5.116014	-1.987453
C	-0.571041	3.264321	-0.906067
H	-0.539270	2.663202	-1.824809
C	-2.304977	0.689797	1.805810
C	-2.372235	-0.381177	2.721938
H	-1.563241	-1.125095	2.755614
C	-3.462125	-0.509162	3.603906
H	-3.493118	-1.352674	4.311833
C	-4.500114	0.438273	3.586939
H	-5.349041	0.344790	4.282645
C	-4.448527	1.506007	2.671012
H	-5.260013	2.250737	2.643602
C	-3.364770	1.627124	1.784391
H	-3.346038	2.465004	1.070749
C	-0.856486	-2.361633	-0.232946
H	-1.436688	-2.269297	0.710731
C	1.251892	-3.082840	0.879191
H	0.677460	-3.274652	1.815109
H	1.303505	-4.045209	0.326347
C	2.641751	-2.636927	1.237284
C	4.014452	-0.815696	1.742104
H	4.110556	0.277008	1.811415
C	5.088886	-1.656160	2.055005
H	6.041919	-1.213466	2.379913
C	4.928859	-3.046276	1.932131
C	3.685685	-3.537651	1.504077
H	3.509962	-4.617857	1.388355
C	-1.020181	-3.797634	-0.761797
H	-2.064333	-3.952118	-1.095612
H	-0.352293	-3.991773	-1.626526
H	-0.811053	-4.554614	0.018147
C	1.901511	1.366471	1.309496
C	0.767860	-0.372796	2.778673
H	1.090848	-1.920804	-0.805601
H	1.732556	0.180603	-0.514575
C	2.375437	-0.347980	-1.967521
C	3.781330	0.141535	-1.727498
C	1.698050	0.797707	-2.765336
C	3.870052	1.519430	-2.027620
C	4.919418	-0.604870	-1.381261
C	2.508859	2.064784	-2.418251
H	1.834504	0.534547	-3.839896
C	5.110836	2.173459	-1.957004

C	6.162977	0.050762	-1.317829
H	4.831527	-1.685551	-1.185758
H	2.056302	2.588999	-1.546312
C	6.254941	1.430933	-1.597806
H	5.197128	3.247008	-2.193141
H	7.072049	-0.514628	-1.056672
H	7.235676	1.931412	-1.548990
H	2.562354	2.803131	-3.244893
H	0.610302	0.864342	-2.577475
O	2.068465	-1.570801	-2.083672
H	5.756445	-3.734872	2.160772

3/0-fRSR

Frequencies, energies and thermodynamic properties:

Lowest Vibrational Mode (1/cm) =	17.9738
E(RB-P86) (a.u.) =	-4499.03335668
Thermal correction to Enthalpy (a.u.) =	0.643384
Thermal correction to Gibbs Free Energy (a.u.) =	0.524981
Total Entropy (cal/Kmol) =	249.201
E(RPBE1PBE) (a.u.) =	-4498.35777264

Optimised cartesian coordinates (Angstrom):

Fe	-3.273425	-1.173250	-1.007267
Mn	0.636163	-0.165667	1.829804
P	-0.450646	0.795330	-0.061626
O	0.932885	2.552111	2.953601
O	-1.868755	-0.601050	3.339409
N	0.544181	-2.073047	0.842805
N	2.454297	-0.161187	0.822604
C	-1.360305	-0.458222	-1.029193
C	-1.371875	-1.899448	-0.769146
C	-2.013339	-2.529208	-1.900475
H	-2.203108	-3.603261	-2.016110
C	-2.406630	-1.513593	-2.836566
H	-2.944910	-1.681889	-3.778518
C	-2.017111	-0.238376	-2.306807
H	-2.179508	0.734134	-2.788211
C	-4.270948	-0.748481	0.747420
H	-3.810109	-0.463270	1.701352
C	-4.557373	-2.092001	0.320605
H	-4.349420	-3.008784	0.888266
C	-5.144939	-2.026107	-0.992205
H	-5.453145	-2.883598	-1.604948
C	-5.224350	-0.640158	-1.375553
H	-5.604218	-0.256962	-2.331932
C	-4.680864	0.150586	-0.302890
H	-4.580925	1.243624	-0.291594
C	0.751547	1.531139	-1.265649
C	1.392427	2.742527	-0.911702
H	1.140839	3.248682	0.034057
C	2.342917	3.320367	-1.768570
H	2.826467	4.268166	-1.484280
C	2.675647	2.692080	-2.983528
H	3.420748	3.146622	-3.655180
C	2.051457	1.483632	-3.335944
H	2.302785	0.986250	-4.285929
C	1.093724	0.903657	-2.482662
H	0.600795	-0.032893	-2.784734
C	-1.645405	2.193847	0.185983
C	-2.275371	2.408378	1.430829
H	-2.040991	1.772792	2.295301
C	-3.217685	3.441428	1.584896
H	-3.697361	3.595838	2.564007
C	-3.540699	4.273022	0.498968
H	-4.277357	5.082495	0.621411
C	-2.910869	4.072344	-0.743145
H	-3.150423	4.724124	-1.597991
C	-1.966025	3.044391	-0.899032
H	-1.467561	2.915671	-1.871984
C	-0.811224	-2.622849	0.444895
H	-1.457897	-2.412479	1.318903
C	1.554388	-2.148617	-0.248333
H	1.894725	-3.189780	-0.422459
H	1.060973	-1.813599	-1.186098
C	2.722936	-1.240440	0.032241
C	3.470834	0.718244	1.033734
H	3.250254	1.580332	1.679520
C	4.740121	0.579325	0.489473

```
 H      5.489394   1.347218   0.718377
 C      5.045724  -0.549620  -0.335610
 C      3.971820  -1.470792  -0.541719
 H      4.102746  -2.370308  -1.156716
 C     -0.764920  -4.146978   0.261940
 H     -1.791906  -4.544442   0.142847
 H     -0.177262  -4.451738  -0.627095
 H     -0.328796  -4.632037   1.159344
 C      0.812676   1.478590   2.504678
 C     -0.902709  -0.412483   2.702766
 N      6.278532  -0.737071  -0.884529
 C      6.542903  -1.911948  -1.713151
 H      5.881600  -1.939697  -2.606267
 H      6.399187  -2.856125  -1.144090
 H      7.589455  -1.879609  -2.063446
 C      7.347205   0.229760  -0.636884
 H      7.595288   0.299868   0.444531
 H      7.071981   1.244305  -0.997635
 H      8.255622  -0.090045  -1.176803
 C      1.519447  -0.857154   3.251924
 O      2.069624  -1.252296   4.199440
 H      0.868843  -2.695429   1.597028
```

```
3/0-fRSS
Frequencies, energies and thermodynamic properties:
 Lowest Vibrational Mode (1/cm) =                         14.2119
 E(RB-P86) (a.u.) =                                     -4499.02627959
 Thermal correction to Enthalpy (a.u.) =                  0.643445
 Thermal correction to Gibbs Free Energy (a.u.) =         0.524752
 Total Entropy (cal/Kmol) =                               249.809
 E(RPBE1PBE) (a.u.) =                                   -4498.35049919
Optimised cartesian coordinates (Angstrom):
 Fe     1.358039   2.853532  -0.573893
 Mn    -0.335325  -1.914813  -0.902761
 P      1.133495  -0.570838   0.460234
 N     -0.596912  -0.217374  -2.229983
 N     -2.165965  -1.172780  -0.218982
 C      1.814184   0.839627  -0.489678
 C      1.468344   1.219136  -1.855186
 C      2.377570   2.268801  -2.251617
 H      2.371019   2.781693  -3.222965
 C      3.285769   2.529152  -1.169747
 H      4.082248   3.284590  -1.163958
 C      2.931514   1.671653  -0.074695
 H      3.429790   1.640707   0.902099
 C     -0.363645   3.385843   0.456427
 H     -1.133229   2.717862   0.864194
 C     -0.357758   3.986651  -0.852598
 H     -1.117999   3.855723  -1.633541
 C      0.823923   4.799043  -0.960354
 H      1.132294   5.376823  -1.841613
 C      1.545121   4.703501   0.283865
 H      2.501621   5.191951   0.512141
 C      0.813174   3.825475   1.158684
 H      1.104739   3.531411   2.175081
 C      2.649365  -1.558774   0.881363
 C      2.613978  -2.449184   1.980104
 H      1.720002  -2.508322   2.619978
 C      3.724114  -3.258702   2.274318
 H      3.684216  -3.942599   3.136783
 C      4.880113  -3.193889   1.474891
 H      5.750326  -3.827357   1.708289
 C      4.918886  -2.316269   0.377713
 H      5.819218  -2.258561  -0.254146
 C      3.810054  -1.503854   0.079066
 H      3.856813  -0.822021  -0.783214
 C      0.661943   0.119281   2.118902
 C     -0.695863   0.262388   2.475698
 H     -1.481446  -0.062653   1.781555
 C     -1.052745   0.810986   3.721439
 H     -2.117128   0.911549   3.985334
 C     -0.057415   1.220028   4.624848
 H     -0.336718   1.646915   5.600966
 C      1.299453   1.071979   4.281080
 H      2.086687   1.380576   4.986872
 C      1.659065   0.520735   3.040029
 H      2.725127   0.389490   2.799805
```

C	0.525139	0.571547	-2.850470
H	0.038174	1.402584	-3.411096
C	-1.584991	0.711574	-1.616858
H	-1.004312	1.394356	-0.959985
H	-2.067435	1.343684	-2.392381
C	-2.607876	-0.020661	-0.799833
C	-3.047049	-1.839316	0.578632
H	-2.691465	-2.775704	1.032348
C	-4.335311	-1.397945	0.845483
H	-4.963698	-2.005648	1.508325
C	-4.816411	-0.186844	0.251093
C	-3.890259	0.489680	-0.603026
H	-4.163776	1.419849	-1.116942
C	1.286600	-0.271072	-3.886575
H	1.935372	0.392925	-4.490092
H	1.932074	-1.036037	-3.418393
H	0.582691	-0.772522	-4.583486
C	1.137183	-2.610066	-1.647720
O	2.042396	-3.173700	-2.128703
C	-0.281362	-3.182179	0.351594
O	-0.262248	-4.023991	1.162808
N	-6.073426	0.285534	0.477689
C	-6.523401	1.516670	-0.170158
H	-6.514868	1.424377	-1.277994
H	-5.886722	2.382876	0.112146
H	-7.558057	1.735112	0.147057
C	-6.987680	-0.447609	1.352797
H	-6.569633	-0.559739	2.376210
H	-7.214205	-1.460755	0.955139
H	-7.937220	0.110168	1.431611
C	-1.301517	-2.991779	-1.985944
O	-1.897070	-3.729429	-2.663123
H	-1.066561	-0.666570	-3.029690

3/0-mRSR

Frequencies, energies and thermodynamic properties:

Lowest Vibrational Mode (1/cm) =	20.7414
E(RB-P86) (a.u.) =	-4499.02484347
Thermal correction to Enthalpy (a.u.) =	0.643104
Thermal correction to Gibbs Free Energy (a.u.) =	0.524886
Total Entropy (cal/Kmol) =	248.811
E(RPBE1PBE) (a.u.) =	-4498.34822879

Optimised cartesian coordinates (Angstrom):

Fe	1.827553	2.725813	-0.378200
Mn	-0.850786	-0.891737	0.247396
P	1.450694	-0.799832	0.085874
O	-0.715831	-3.056668	2.241855
N	-1.090418	0.702384	-1.219149
N	-2.914305	-0.700394	0.235555
C	2.007337	0.712301	-0.772163
C	1.227703	1.387772	-1.802569
C	2.065988	2.406305	-2.387218
H	1.765266	3.093744	-3.189385
C	3.338437	2.386080	-1.720333
H	4.177518	3.067361	-1.913063
C	3.305166	1.358153	-0.716723
H	4.118818	1.108493	-0.024569
C	1.288000	3.107107	1.583965
H	1.187615	2.373473	2.392845
C	0.243140	3.532230	0.685484
H	-0.810602	3.225420	0.725660
C	0.802829	4.500230	-0.222425
H	0.268900	5.009389	-1.035444
C	2.189886	4.678689	0.121843
H	2.902053	5.336858	-0.392975
C	2.488202	3.817218	1.237061
H	3.466300	3.705677	1.722969
C	2.217254	-2.169896	-0.913723
C	2.040131	-3.501756	-0.470313
H	1.474464	-3.711613	0.451793
C	2.594117	-4.570642	-1.192831
H	2.451926	-5.602134	-0.833806
C	3.326072	-4.325155	-2.370170
H	3.757321	-5.164625	-2.937879
C	3.506053	-3.005020	-2.816112
H	4.081378	-2.803764	-3.733558
C	2.957393	-1.929577	-2.091581

```
 H      3.116613  -0.900139  -2.446408
 C      2.503323  -0.878331   1.619130
 C      1.973844  -0.643830   2.905929
 H      0.906055  -0.431451   3.044772
 C      2.803771  -0.690646   4.041224
 H      2.370221  -0.508533   5.037008
 C      4.172864  -0.975400   3.906179
 H      4.820947  -1.014268   4.795800
 C      4.708818  -1.223297   2.629325
 H      5.778419  -1.458713   2.512904
 C      3.880921  -1.182772   1.495033
 H      4.312660  -1.402744   0.506486
 C     -0.124956   0.991549  -2.342383
 H     -0.530768   1.881450  -2.876619
 C     -2.487101   0.647736  -1.729634
 H     -2.804057   1.631486  -2.137513
 H     -2.509560  -0.071761  -2.575296
 C     -3.447227   0.153331  -0.681771
 C     -3.763352  -1.255808   1.140700
 H     -3.318490  -1.942586   1.875475
 C     -5.127135  -0.997550   1.173574
 H     -5.731177  -1.500101   1.939148
 C     -5.707776  -0.088151   0.232488
 C     -4.800010   0.488127  -0.710436
 H     -5.145721   1.192058  -1.478105
 C     -0.000938  -0.145169  -3.367244
 H      0.673381   0.188065  -4.180519
 H      0.438445  -1.058902  -2.923496
 H     -0.970880  -0.405586  -3.835151
 C     -0.765802  -2.189669   1.452477
 N     -7.037435   0.210513   0.235439
 C     -7.582053   1.146409  -0.746436
 H     -8.668134   1.254483  -0.579808
 H     -7.118004   2.152508  -0.655064
 H     -7.427910   0.785380  -1.786609
 C     -7.925681  -0.404187   1.220852
 H     -7.940927  -1.511550   1.124826
 H     -7.624128  -0.148956   2.259872
 H     -8.953296  -0.032931   1.062184
 C     -1.011198  -2.218315  -1.025711
 O     -1.171281  -3.119159  -1.741562
 C     -0.928387   0.313620   1.627203
 O     -1.117343   1.009228   2.542749
 H     -1.008831   1.539437  -0.619700
```

```
3/0-mRSS
Frequencies, energies and thermodynamic properties:
 Lowest Vibrational Mode (1/cm) =                         5.5789
 E(RB-P86) (a.u.) =                                    -4499.02050978
 Thermal correction to Enthalpy (a.u.) =                 0.643116
 Thermal correction to Gibbs Free Energy (a.u.) =        0.523132
 Total Entropy (cal/Kmol) =                              252.527
 E(RPBE1PBE) (a.u.) =                                  -4498.34377256
Optimised cartesian coordinates (Angstrom):
 Fe    -3.118004  -2.022539  -0.483045
 Mn     1.218482   0.607218   0.283287
 P     -1.045664   0.931495  -0.043106
 O      1.465414   3.322950   1.404327
 O      0.978093  -0.497298   3.064869
 N      1.244040  -1.429709  -0.502115
 N      3.249086   0.216790   0.354994
 C     -1.810363  -0.499266  -0.895494
 C     -1.166503  -1.804626  -1.025935
 C     -1.891497  -2.543815  -2.037372
 H     -1.679438  -3.566939  -2.370029
 C     -2.977873  -1.731846  -2.509149
 H     -3.724687  -2.028785  -3.257056
 C     -2.940468  -0.479673  -1.808153
 H     -3.628980   0.360797  -1.962666
 C     -3.756033  -1.922339   1.480133
 H     -3.416291  -1.202020   2.234820
 C     -3.162779  -3.204011   1.207538
 H     -2.298339  -3.637105   1.728290
 C     -3.899989  -3.816778   0.132171
 H     -3.689554  -4.794312  -0.321571
 C     -4.950229  -2.910581  -0.259149
 H     -5.679242  -3.077206  -1.063094
```

```
 C     -4.860483  -1.738498   0.573912
 H     -5.518038  -0.861398   0.522885
 C     -1.378145   2.344230  -1.209716
 C     -1.574566   3.646084  -0.694438
 H     -1.608104   3.812671   0.393175
 C     -1.737187   4.739172  -1.563704
 H     -1.893533   5.746232  -1.145792
 C     -1.707153   4.547656  -2.956416
 H     -1.841033   5.403647  -3.636416
 C     -1.504270   3.256727  -3.476169
 H     -1.477181   3.096194  -4.565566
 C     -1.334357   2.161913  -2.610757
 H     -1.170009   1.160942  -3.037840
 C     -2.143527   1.401401   1.380258
 C     -1.623156   1.710685   2.653650
 H     -0.543201   1.661948   2.845828
 C     -2.479081   2.105569   3.700123
 H     -2.054146   2.341660   4.688246
 C     -3.862696   2.202913   3.482312
 H     -4.531946   2.512169   4.300519
 C     -4.389767   1.912029   2.209293
 H     -5.472524   1.995543   2.025856
 C     -3.537892   1.517886   1.165840
 H     -3.966940   1.300873   0.176540
 C      0.029007  -2.304960  -0.238256
 H     -0.179168  -2.180167   0.845204
 C      2.492594  -2.055466   0.022779
 H      2.274184  -2.411973   1.053845
 H      2.792764  -2.941345  -0.571404
 C      3.622910  -1.065633   0.096028
 C      4.237867   1.134336   0.522396
 H      3.922306   2.167313   0.729342
 C      5.590345   0.827925   0.445411
 H      6.314152   1.637631   0.600927
 C      6.008160  -0.511166   0.159410
 C      4.954360  -1.461433  -0.019282
 H      5.167099  -2.515276  -0.239583
 C      0.289512  -3.795225  -0.514419
 H     -0.647541  -4.362206  -0.355669
 H      0.622051  -3.969851  -1.558406
 H      1.044312  -4.221885   0.171750
 C      1.352820   2.244048   0.957157
 C      1.032342  -0.083701   1.978168
 N      7.322544  -0.858347   0.060984
 C      7.696387  -2.240259  -0.234111
 H      8.797217  -2.319306  -0.263360
 H      7.322150  -2.940930   0.543623
 H      7.301134  -2.570928  -1.219310
 C      8.364332   0.150836   0.245355
 H      8.280489   0.968196  -0.503379
 H      8.322728   0.602641   1.260242
 H      9.353521  -0.324213   0.123076
 C      1.561206   1.280994  -1.398770
 O      1.867015   1.719065  -2.429491
 H      1.339961  -1.359099  -1.528007

3/3-fRSR
Frequencies, energies and thermodynamic properties:
 Lowest Vibrational Mode (1/cm) =                          20.2581
 E(RB-P86) (a.u.) =                                      -4386.47666671
 Thermal correction to Enthalpy (a.u.) =                   0.638122
 Thermal correction to Gibbs Free Energy (a.u.) =          0.523989
 Total Entropy (cal/Kmol) =                                240.213
 E(RPBE1PBE) (a.u.) =                                    -4385.79016859
Optimised cartesian coordinates (Angstrom):
 Fe    -3.224849  -1.302099  -0.777978
 Mn     0.632465   0.035074   1.852175
 P     -0.390810   0.769377  -0.039514
 O      1.070799   2.692583   3.026837
 O     -1.707996  -0.343088   3.587718
 N      0.614308  -1.972763   1.074519
 N      2.529526  -0.096111   1.026457
 C     -1.315084  -0.560054  -0.925571
 C     -1.299438  -1.973313  -0.544111
 C     -1.972427  -2.708222  -1.592286
 H     -2.149753  -3.790828  -1.608501
 C     -2.414011  -1.781570  -2.598442
```

H	-2.979462	-2.037219	-3.504630
C	-2.018585	-0.462261	-2.191102
H	-2.211849	0.465511	-2.744745
C	-4.119982	-0.743915	0.990620
H	-3.592043	-0.384793	1.883855
C	-4.417633	-2.119189	0.686980
H	-4.166136	-2.987452	1.310975
C	-5.082478	-2.161114	-0.590550
H	-5.421258	-3.065999	-1.112731
C	-5.198923	-0.809441	-1.075394
H	-5.642090	-0.503898	-2.032749
C	-4.602451	0.066272	-0.100399
H	-4.510899	1.157191	-0.179972
C	0.761466	1.408851	-1.362263
C	1.455885	2.613235	-1.089670
H	1.241404	3.168437	-0.161504
C	2.411160	3.112823	-1.989795
H	2.932379	4.057212	-1.764271
C	2.705800	2.408667	-3.173600
H	3.456677	2.798789	-3.879036
C	2.036635	1.203170	-3.445799
H	2.259883	0.642850	-4.368159
C	1.071631	0.706479	-2.547619
H	0.547649	-0.232282	-2.785925
C	-1.619015	2.174482	0.035601
C	-2.245688	2.490208	1.260477
H	-1.989832	1.918597	2.164500
C	-3.197326	3.524238	1.333703
H	-3.676360	3.756786	2.298341
C	-3.532550	4.261469	0.183866
H	-4.275496	5.072952	0.241888
C	-2.905838	3.962047	-1.040141
H	-3.155030	4.539309	-1.945110
C	-1.953735	2.929826	-1.112636
H	-1.458088	2.720013	-2.073601
C	-0.709619	-2.587205	0.720424
H	-1.362770	-2.319067	1.574714
C	1.660124	-2.134351	0.040012
H	1.991912	-3.188750	-0.074696
H	1.216863	-1.829085	-0.933888
C	2.833375	-1.233229	0.337370
C	3.551736	0.773369	1.239203
H	3.300256	1.688458	1.795729
C	4.860417	0.563294	0.813532
H	5.611149	1.331049	1.040619
C	5.199489	-0.632739	0.109294
C	4.118826	-1.539084	-0.114783
H	4.271558	-2.486805	-0.647909
C	-0.634738	-4.121902	0.653889
H	-1.652002	-4.555058	0.580712
H	-0.050527	-4.482125	-0.217283
H	-0.171589	-4.524928	1.578222
C	0.880007	1.624840	2.540523
C	-0.799138	-0.178699	2.837030
H	0.935312	-2.439039	1.933785
H	1.351649	-0.635955	3.100932
N	6.473730	-0.894431	-0.319047
C	6.767381	-2.136046	-1.026827
H	6.178392	-2.224987	-1.966391
H	6.552437	-3.031101	-0.401487
H	7.838856	-2.156862	-1.294521
C	7.545038	0.059624	-0.050542
H	7.696605	0.216537	1.040578
H	7.342124	1.050213	-0.514360
H	8.489429	-0.326789	-0.473536

3/3-fRSS

Frequencies, energies and thermodynamic properties:

Lowest Vibrational Mode (1/cm) =	16.6102
E(RB-P86) (a.u.) =	-4386.47038875
Thermal correction to Enthalpy (a.u.) =	0.638204
Thermal correction to Gibbs Free Energy (a.u.) =	0.523779
Total Entropy (cal/Kmol) =	240.829
E(RPBE1PBE) (a.u.) =	-4385.78404788

Optimised cartesian coordinates (Angstrom):

Fe	1.398419	2.816189	-0.212098
Mn	-0.364166	-1.796461	-1.176823

P	1.059510	-0.749472	0.266782
N	-0.674279	0.068832	-2.253440
N	-2.258782	-1.252764	-0.507898
C	1.800497	0.789912	-0.445289
C	1.428719	1.387286	-1.722717
C	2.353530	2.467095	-1.983887
H	2.333898	3.123403	-2.865040
C	3.297705	2.538677	-0.902506
H	4.115719	3.265003	-0.805868
C	2.948645	1.524723	0.053776
H	3.472496	1.330482	0.998140
C	-0.288835	3.189292	0.930087
H	-1.044865	2.462928	1.255468
C	-0.316048	3.971284	-0.279889
H	-1.096408	3.948386	-1.051847
C	0.864263	4.794890	-0.300659
H	1.149339	5.495193	-1.097112
C	1.618219	4.523153	0.897837
H	2.580494	4.977221	1.169624
C	0.907094	3.527244	1.656780
H	1.224104	3.088710	2.611676
C	2.575323	-1.794998	0.561402
C	2.535672	-2.810972	1.545317
H	1.646964	-2.919499	2.186903
C	3.620024	-3.689260	1.715067
H	3.571640	-4.471233	2.490134
C	4.762038	-3.571467	0.900864
H	5.613042	-4.258096	1.035457
C	4.806684	-2.572005	-0.087344
H	5.694150	-2.472460	-0.733171
C	3.721148	-1.692948	-0.258379
H	3.771171	-0.918334	-1.038956
C	0.657737	-0.254241	2.029623
C	-0.700053	-0.194534	2.410663
H	-1.473827	-0.454145	1.673142
C	-1.066943	0.178694	3.717891
H	-2.132248	0.218594	3.996516
C	-0.076625	0.488866	4.666084
H	-0.360326	0.777049	5.690959
C	1.282004	0.416723	4.302431
H	2.064792	0.646433	5.043356
C	1.646212	0.043777	2.996806
H	2.713926	-0.030421	2.737278
C	0.425019	0.938124	-2.772489
H	-0.052027	1.867311	-3.166900
C	-1.666316	0.864122	-1.496252
H	-1.103734	1.378628	-0.687529
H	-2.128064	1.655791	-2.128003
C	-2.713500	-0.023646	-0.885123
C	-3.157302	-2.066236	0.109756
H	-2.786358	-3.058353	0.408123
C	-4.478240	-1.715497	0.372179
H	-5.119585	-2.447633	0.879422
C	-4.971234	-0.433188	-0.023423
C	-4.024542	0.411689	-0.679303
H	-4.302685	1.414004	-1.030592
C	1.130088	0.266749	-3.964269
H	1.826642	0.985907	-4.437828
H	1.706353	-0.625286	-3.657443
H	0.389146	-0.038506	-4.733549
C	0.947648	-2.437672	-2.145292
O	1.756219	-2.977658	-2.826211
C	-0.378686	-3.302772	-0.291254
O	-0.413102	-4.340402	0.287427
H	-1.220591	-2.428101	-2.353650
H	-1.146942	-0.325072	-3.078686
N	-6.263322	-0.042557	0.206612
C	-6.714774	1.278380	-0.218121
H	-6.639187	1.408355	-1.320686
H	-6.126071	2.090047	0.263849
H	-7.773344	1.410190	0.068541
C	-7.195034	-0.951242	0.867301
H	-6.850947	-1.225110	1.889183
H	-7.336461	-1.891316	0.289321
H	-8.178667	-0.457490	0.960501

3/3-mRSR

```
Frequencies, energies and thermodynamic properties:
 Lowest Vibrational Mode (1/cm) =                          19.3172
 E(RB-P86) (a.u.) =                                       -4386.47843457
 Thermal correction to Enthalpy (a.u.) =                   0.637857
 Thermal correction to Gibbs Free Energy (a.u.) =          0.522698
 Total Entropy (cal/Kmol) =                                242.373
 E(RPBE1PBE) (a.u.) =                                     -4385.79012111
Optimised cartesian coordinates (Angstrom):
 Fe     1.492000   2.789601  -0.352873
 Mn    -0.773161  -1.144343   0.075903
 P      1.413085  -0.741633   0.133244
 O     -0.587885  -3.043545   2.306111
 N     -1.100130   0.486035  -1.326100
 N     -2.800618  -0.897893   0.217324
 C      1.913979   0.804390  -0.734884
 C      1.143557   1.403389  -1.814639
 C      1.903763   2.513901  -2.338754
 H      1.587422   3.173008  -3.159035
 C      3.122072   2.625225  -1.582521
 H      3.897429   3.391450  -1.716887
 C      3.126086   1.585699  -0.587879
 H      3.909438   1.414540   0.161742
 C      0.103496   2.980062   1.163341
 H     -0.503631   2.153005   1.559048
 C     -0.241158   3.832913   0.054273
 H     -1.178118   3.808272  -0.518928
 C      0.859857   4.734362  -0.172175
 H      0.915922   5.500724  -0.956789
 C      1.881877   4.441334   0.800601
 H      2.854736   4.944043   0.883612
 C      1.416991   3.353966   1.622611
 H      1.969439   2.881185   2.444869
 C      2.529393  -2.007459  -0.669656
 C      2.471656  -3.336005  -0.183974
 H      1.817973  -3.577837   0.670013
 C      3.241205  -4.350484  -0.775937
 H      3.189623  -5.377165  -0.378796
 C      4.069890  -4.060143  -1.877556
 H      4.668692  -4.857374  -2.346207
 C      4.125427  -2.747217  -2.375350
 H      4.769976  -2.509465  -3.237106
 C      3.363663  -1.725935  -1.773687
 H      3.426775  -0.700073  -2.168167
 C      2.275349  -0.548789   1.784887
 C      1.539693  -0.154967   2.923420
 H      0.455340   0.008303   2.815930
 C      2.177191   0.020278   4.165006
 H      1.586293   0.325963   5.043643
 C      3.561096  -0.200596   4.288428
 H      4.060149  -0.067074   5.261653
 C      4.302724  -0.601759   3.162333
 H      5.386080  -0.783771   3.249289
 C      3.664496  -0.778363   1.921029
 H      4.256568  -1.104978   1.051644
 C     -0.146868   0.889734  -2.408615
 H     -0.611869   1.744608  -2.956144
 C     -2.476744   0.314994  -1.839472
 H     -2.852813   1.233726  -2.344625
 H     -2.455261  -0.494877  -2.601441
 C     -3.393991  -0.110404  -0.724357
 C     -3.604090  -1.366885   1.208034
 H     -3.117696  -2.000346   1.964881
 C     -4.965014  -1.089947   1.305886
 H     -5.523934  -1.522407   2.145808
 C     -5.598674  -0.254002   0.335176
 C     -4.746165   0.238036  -0.699486
 H     -5.132061   0.888324  -1.495648
 C      0.125046  -0.224024  -3.432888
 H      0.789373   0.172880  -4.226212
 H      0.623614  -1.092199  -2.960830
 H     -0.803786  -0.577073  -3.924290
 C     -0.653727  -2.277277   1.398642
 N     -6.931788   0.057658   0.394513
 C     -7.525660   0.922771  -0.618791
 H     -8.600501   1.054179  -0.400461
 H     -7.053311   1.930317  -0.632260
 H     -7.434336   0.488348  -1.639120
```

C	-7.756789	-0.466342	1.477960
H	-7.774169	-1.578755	1.483782
H	-7.399049	-0.124305	2.474666
H	-8.794661	-0.111054	1.348505
H	-0.722080	0.023107	1.190896
H	-1.098110	1.273196	-0.659754
C	-0.757032	-2.463515	-1.135871
O	-0.694182	-3.410109	-1.848585

3/3-mRSS

Frequencies, energies and thermodynamic properties:

Lowest Vibrational Mode (1/cm) =	16.7247
E(RB-P86) (a.u.) =	-4386.47003862
Thermal correction to Enthalpy (a.u.) =	0.638138
Thermal correction to Gibbs Free Energy (a.u.) =	0.523355
Total Entropy (cal/Kmol) =	241.580
E(RPBE1PBE) (a.u.) =	-4385.77957941

Optimised cartesian coordinates (Angstrom):

Fe	-3.055059	-1.918384	-0.619751
Mn	1.246724	0.598280	0.511500
P	-0.887232	0.955791	-0.053592
O	1.578476	3.435425	1.194135
O	1.030149	-0.216672	3.368100
N	1.291750	-1.436698	-0.265358
N	3.260960	0.273383	0.357651
C	-1.625132	-0.473710	-0.968303
C	-1.043199	-1.811169	-0.949894
C	-1.691366	-2.584423	-1.988342
H	-1.494420	-3.635044	-2.234792
C	-2.678822	-1.755559	-2.624612
H	-3.353977	-2.064485	-3.433916
C	-2.648768	-0.463268	-1.995080
H	-3.276719	0.395183	-2.265983
C	-3.866951	-1.662315	1.258921
H	-3.550468	-0.917916	2.000400
C	-3.336844	-2.992651	1.113949
H	-2.553014	-3.445178	1.736109
C	-4.009133	-3.622478	0.005416
H	-3.822615	-4.636716	-0.372260
C	-4.955589	-2.678539	-0.534557
H	-5.615778	-2.848513	-1.395552
C	-4.866012	-1.465922	0.239506
H	-5.450082	-0.551024	0.077534
C	-1.075351	2.346475	-1.292260
C	-1.311329	3.666731	-0.848894
H	-1.478987	3.866484	0.220715
C	-1.334030	4.739438	-1.760166
H	-1.519865	5.761575	-1.392492
C	-1.123067	4.509339	-3.130757
H	-1.146314	5.347585	-3.845331
C	-0.876845	3.198753	-3.581777
H	-0.704629	3.006031	-4.653133
C	-0.844885	2.129549	-2.669849
H	-0.632525	1.113627	-3.037442
C	-2.217496	1.448294	1.168851
C	-1.946158	1.433733	2.552388
H	-0.951504	1.132206	2.909504
C	-2.943139	1.785272	3.484400
H	-2.713060	1.765214	4.561843
C	-4.222507	2.162823	3.043201
H	-5.001961	2.440082	3.770847
C	-4.501470	2.188927	1.662788
H	-5.500309	2.488407	1.306469
C	-3.507432	1.837124	0.734828
H	-3.740871	1.872961	-0.340514
C	0.069597	-2.288160	-0.033906
H	-0.220020	-2.089022	1.020179
C	2.534847	-2.022030	0.290896
H	2.341900	-2.242818	1.365249
H	2.824825	-2.974480	-0.200911
C	3.654454	-1.020340	0.193992
C	4.240076	1.214611	0.336578
H	3.912892	2.256620	0.469685
C	5.591485	0.927935	0.162678
H	6.303856	1.763019	0.164321
C	6.020785	-0.422873	-0.020739
C	4.982559	-1.403464	-0.005422

H	5.203043	-2.470437	-0.141710
C	0.316253	-3.797877	-0.193102
H	-0.639067	-4.341194	-0.055427
H	0.711114	-4.049933	-1.199309
H	1.022805	-4.183645	0.566379
C	1.420134	2.287981	0.929209
C	1.101318	0.066574	2.215748
H	1.414682	-1.333963	-1.284429
H	1.366435	1.029106	-1.034943
N	7.337612	-0.754981	-0.202563
C	7.719379	-2.149699	-0.395360
H	8.814657	-2.212546	-0.524095
H	7.440635	-2.779728	0.478432
H	7.242578	-2.588710	-1.299965
C	8.358902	0.287179	-0.213585
H	8.198896	1.016799	-1.038474
H	8.382395	0.852727	0.744185
H	9.350539	-0.177671	-0.357721

4/0-fRSR

Frequencies, energies and thermodynamic properties:

Lowest Vibrational Mode (1/cm) =	22.2594
E(RB-P86) (a.u.) =	-4499.01032924
Thermal correction to Enthalpy (a.u.) =	0.642365
Thermal correction to Gibbs Free Energy (a.u.) =	0.526146
Total Entropy (cal/Kmol) =	244.604
E(RPBE1PBE) (a.u.) =	-4498.34207951

Optimised cartesian coordinates (Angstrom):

Fe	3.454009	-0.503299	0.597235
Mn	-1.110075	-0.871522	-1.261978
P	0.110592	0.666262	0.118659
O	-2.062660	1.443791	-2.855227
O	1.090303	-1.081579	-3.212491
N	-0.366958	-2.462155	-0.028129
N	-2.665134	-1.006553	0.172257
C	1.476766	-0.175592	0.996519
C	1.770089	-1.610284	0.966761
C	2.770218	-1.860495	1.979605
H	3.226161	-2.831868	2.206672
C	3.107262	-0.618306	2.616361
H	3.860312	-0.483393	3.403775
C	2.324499	0.422456	2.014363
H	2.353810	1.485324	2.284589
C	3.922351	-0.217682	-1.391392
H	3.209848	-0.183734	-2.224624
C	4.572207	-1.398213	-0.888152
H	4.440440	-2.420468	-1.267134
C	5.410816	-1.008155	0.215122
H	6.021547	-1.681432	0.831124
C	5.280711	0.415317	0.391934
H	5.775735	1.016008	1.166474
C	4.358032	0.904765	-0.598388
H	4.031271	1.945693	-0.719419
C	-0.896871	1.426766	1.477833
C	-1.786508	2.476968	1.148027
H	-1.845132	2.848949	0.112731
C	-2.580335	3.074071	2.141076
H	-3.254892	3.902730	1.873758
C	-2.509427	2.621622	3.472117
H	-3.131272	3.092041	4.249775
C	-1.637104	1.571627	3.804306
H	-1.570565	1.213723	4.843666
C	-0.832956	0.976469	2.814269
H	-0.136258	0.173671	3.099614
C	0.888146	2.168006	-0.652482
C	1.152769	2.235182	-2.037268
H	0.858630	1.416808	-2.706857
C	1.799411	3.356969	-2.587385
H	1.994347	3.391980	-3.670585
C	2.188711	4.425956	-1.762757
H	2.693431	5.304093	-2.195198
C	1.921591	4.372134	-0.382418
H	2.214742	5.207750	0.272355
C	1.272168	3.255336	0.169160
H	1.054312	3.241526	1.247839
C	1.133731	-2.662808	0.074481
H	1.499342	-2.528538	-0.961784

```
 C     -1.044820  -2.469089   1.291889
 H     -1.143602  -3.496656   1.700228
 H     -0.401692  -1.912925   2.007387
 C     -2.392662  -1.799627   1.245589
 C     -3.870802  -0.350674   0.133808
 C     -4.764986  -0.412981   1.223654
 H     -5.716668   0.133072   1.165668
 C     -4.466903  -1.210766   2.332644
 C     -3.270346  -1.937245   2.332428
 H     -2.995481  -2.591555   3.172384
 C      1.476955  -4.096130   0.505012
 H      2.567349  -4.266845   0.411723
 H      1.188092  -4.305917   1.554484
 H      0.973180  -4.830977  -0.155863
 C     -1.776233   0.537150  -2.175073
 C      0.249873  -0.963953  -2.402065
 H     -0.704470  -3.287572  -0.543500
 H     -5.172231  -1.285866   3.174246
 N     -4.191899   0.380947  -1.026929
 C     -4.769239  -0.400908  -2.130803
 H     -5.868819  -0.539840  -1.993396
 H     -4.307515  -1.400999  -2.192576
 H     -4.600128   0.125426  -3.092790
 C     -4.892696   1.650541  -0.837248
 H     -4.754457   2.266171  -1.750487
 H     -4.463911   2.201127   0.020574
 H     -5.991861   1.528431  -0.677494
 C     -1.937563  -2.062254  -2.336360
 O     -2.393647  -2.833305  -3.084448
```

4/0-fRSS

```
Frequencies, energies and thermodynamic properties:
 Lowest Vibrational Mode (1/cm) =                         11.0635
 E(RB-P86) (a.u.) =                                     -4499.00399779
 Thermal correction to Enthalpy (a.u.) =                  0.642300
 Thermal correction to Gibbs Free Energy (a.u.) =         0.525330
 Total Entropy (cal/Kmol) =                               246.185
 E(RPBE1PBE) (a.u.) =                                   -4498.33479681
Optimised cartesian coordinates (Angstrom):
 Fe    -3.343593  -0.652280  -0.361484
 Mn     1.624886   0.206423  -1.171203
 P     -0.160914   0.861320   0.329721
 N      0.251530  -1.195631  -2.068173
 N      2.187116  -1.592321  -0.207673
 C     -1.763878   0.662664  -0.548101
 C     -1.994045  -0.042874  -1.808302
 C     -3.356332   0.223928  -2.207645
 H     -3.845259  -0.175449  -3.106394
 C     -3.965253   1.087834  -1.235654
 H     -4.999195   1.455804  -1.256808
 C     -3.002117   1.344604  -0.203635
 H     -3.167703   1.973682   0.679420
 C     -3.006165  -2.127024   1.054266
 H     -2.074781  -2.314049   1.604874
 C     -3.390205  -2.716863  -0.202963
 H     -2.810460  -3.444069  -0.785768
 C     -4.682304  -2.195657  -0.561165
 H     -5.245769  -2.435631  -1.472419
 C     -5.099096  -1.287629   0.477675
 H     -6.033670  -0.711650   0.492688
 C     -4.061531  -1.243539   1.475195
 H     -4.068412  -0.637320   2.389635
 C     -0.075937   2.686611   0.666471
 C      0.704559   3.144961   1.753457
 H      1.208042   2.426035   2.418168
 C      0.836918   4.521162   2.003232
 H      1.443603   4.862297   2.856860
 C      0.196227   5.456878   1.170887
 H      0.297325   6.535340   1.370220
 C     -0.574366   5.008331   0.084272
 H     -1.080179   5.732963  -0.573140
 C     -0.709181   3.631608  -0.170825
 H     -1.316313   3.299330  -1.025438
 C     -0.417804   0.208380   2.052411
 C      0.356932  -0.860021   2.549268
 H      1.132962  -1.314702   1.922842
 C      0.153323  -1.347053   3.854818
```

H	0.769224	-2.181399	4.224790
C	-0.823158	-0.766419	4.679820
H	-0.982251	-1.145143	5.701643
C	-1.590557	0.311499	4.198191
H	-2.349440	0.782415	4.842681
C	-1.387672	0.799689	2.897685
H	-1.981040	1.658798	2.551315
C	-1.047137	-0.807058	-2.716353
H	-1.558880	-1.762801	-2.974872
C	0.044967	-2.300171	-1.100894
H	-0.733595	-1.964176	-0.380368
H	-0.356589	-3.205970	-1.604477
C	1.306238	-2.620721	-0.356443
C	3.319492	-1.799977	0.536828
C	3.540040	-3.029822	1.190332
H	4.452987	-3.158744	1.788795
C	2.627286	-4.080704	1.040506
C	1.497560	-3.877837	0.237566
H	0.753548	-4.672083	0.081456
C	-0.793329	-0.075386	-4.044502
H	-1.747680	0.024870	-4.596453
H	-0.380326	0.939096	-3.899818
H	-0.100068	-0.658723	-4.686131
C	1.161618	1.659774	-2.088738
O	0.970589	2.634527	-2.709854
C	2.781209	1.247465	-0.247396
O	3.441792	2.075214	0.247065
H	0.819030	-1.581389	-2.837429
N	4.241362	-0.735638	0.645936
C	4.734880	-0.438678	1.991099
H	5.131702	0.598217	1.996143
H	5.557636	-1.118070	2.322707
H	3.906903	-0.493080	2.723464
C	5.309845	-0.719490	-0.362807
H	6.136979	-1.422070	-0.097936
H	5.731541	0.304144	-0.438625
H	4.919702	-1.009635	-1.354378
H	2.804503	-5.050837	1.529031
C	2.822854	-0.087574	-2.483054
O	3.563565	-0.211906	-3.376842

4/0-mRSR

Frequencies, energies and thermodynamic properties:

Lowest Vibrational Mode (1/cm) =	25.0711
E(RB-P86) (a.u.) =	-4499.00548314
Thermal correction to Enthalpy (a.u.) =	0.642578
Thermal correction to Gibbs Free Energy (a.u.) =	0.526491
Total Entropy (cal/Kmol) =	244.325
E(RPBE1PBE) (a.u.) =	-4498.33444448

Optimised cartesian coordinates (Angstrom):

Fe	-2.559299	-2.147916	-0.068261
Mn	1.328017	0.261715	-0.124590
P	-0.858453	0.995858	0.038802
O	2.044535	2.444200	1.722857
N	0.697188	-1.378607	-1.403682
N	3.221652	-0.725346	-0.374021
C	-2.052483	-0.226781	-0.600464
C	-1.751937	-1.168906	-1.671164
C	-2.985387	-1.816033	-2.045072
H	-3.089731	-2.587937	-2.819412
C	-4.037066	-1.303495	-1.210293
H	-5.086405	-1.625830	-1.226069
C	-3.469843	-0.337240	-0.310403
H	-4.012941	0.218287	0.464194
C	-1.869317	-2.661472	1.816734
H	-1.362528	-2.006813	2.535922
C	-1.233096	-3.475915	0.810926
H	-0.151461	-3.600762	0.665036
C	-2.263555	-4.175453	0.087104
H	-2.109469	-4.870173	-0.748881
C	-3.533510	-3.798996	0.652294
H	-4.518167	-4.145260	0.311844
C	-3.288715	-2.862954	1.719344
H	-4.052865	-2.373355	2.336959
C	-1.223262	2.516332	-0.970494
C	-0.529241	3.710697	-0.663715
H	0.197438	3.736761	0.163906

```
 C     -0.768064   4.880154  -1.403149
 H     -0.223801   5.803838  -1.150896
 C     -1.697060   4.871990  -2.460923
 H     -1.881098   5.789297  -3.041975
 C     -2.390426   3.689802  -2.769963
 H     -3.123030   3.675776  -3.592299
 C     -2.158765   2.515922  -2.027816
 H     -2.719436   1.601435  -2.273051
 C     -1.564120   1.505546   1.683965
 C     -0.997542   1.090869   2.907981
 H     -0.090778   0.473510   2.928744
 C     -1.578163   1.471880   4.131476
 H     -1.117679   1.141049   5.075482
 C     -2.731325   2.274260   4.148733
 H     -3.183144   2.574288   5.107252
 C     -3.298906   2.701369   2.934737
 H     -4.197531   3.338126   2.935965
 C     -2.717693   2.326719   1.711746
 H     -3.164141   2.687181   0.772340
 C     -0.439397  -1.337232  -2.395208
 H     -0.463025  -2.342302  -2.875824
 C      1.922429  -1.940428  -2.023599
 H      1.749848  -2.977775  -2.381197
 H      2.170813  -1.323517  -2.913800
 C      3.091135  -1.885019  -1.079710
 C      4.347686  -0.537679   0.391534
 C      5.263888  -1.606845   0.582903
 H      6.148443  -1.453108   1.214720
 C      5.075302  -2.817983  -0.080969
 C      3.986101  -2.953788  -0.959998
 H      3.821326  -3.875313  -1.535793
 C     -0.264768  -0.296891  -3.509271
 H     -1.085147  -0.426089  -4.242537
 H     -0.322698   0.739016  -3.125078
 H      0.688587  -0.423091  -4.059854
 C      1.816232   1.562864   0.983163
 H      0.360516  -2.059473  -0.702772
 C      1.689301   1.376029  -1.550670
 O      1.990969   2.116303  -2.394324
 H      5.793552  -3.641283   0.053240
 N      4.564912   0.710439   0.961408
 C      5.401253   0.799772   2.154575
 H      6.494463   0.769085   1.928746
 H      5.189920   1.765078   2.657728
 H      5.155705  -0.015231   2.861774
 C      4.723125   1.858455   0.058835
 H      4.349021   2.783828   0.540204
 H      5.798670   2.003513  -0.199573
 H      4.166165   1.697469  -0.879350
 C      1.213632  -0.832470   1.344036
 O      1.287474  -1.514633   2.285551

4/0-mRSS
Frequencies, energies and thermodynamic properties:
 Lowest Vibrational Mode (1/cm) =                          14.1783
 E(RB-P86) (a.u.) =                                      -4498.99758026
 Thermal correction to Enthalpy (a.u.) =                   0.642141
 Thermal correction to Gibbs Free Energy (a.u.) =          0.524784
 Total Entropy (cal/Kmol) =                                246.999
 E(RPBE1PBE) (a.u.) =                                    -4498.32630200
Optimised cartesian coordinates (Angstrom):
 Fe    -3.476465  -1.242944  -0.382184
 Mn     1.544912  -0.152088   0.092612
 P     -0.522767   0.882508  -0.062330
 O      2.475424   2.478188   1.075577
 O      1.182435  -1.054436   2.934996
 N      0.841776  -2.101672  -0.581945
 N      3.363153  -1.199045   0.088681
 C     -1.759838  -0.217954  -0.851666
 C     -1.577948  -1.660689  -0.989615
 C     -2.534803  -2.126946  -1.969994
 H     -2.674213  -3.163785  -2.298792
 C     -3.317714  -1.007364  -2.413109
 H     -4.145852  -1.046712  -3.132773
 C     -2.856067   0.165203  -1.724800
 H     -3.243309   1.182709  -1.863084
 C     -3.997413  -0.956701   1.598354
```

H	-3.427085	-0.386927	2.342360
C	-3.849964	-2.358600	1.313595
H	-3.155735	-3.047143	1.813475
C	-4.771813	-2.698567	0.260097
H	-4.895302	-3.689372	-0.196827
C	-5.489169	-1.503197	-0.105386
H	-6.253992	-1.424662	-0.889216
C	-5.009176	-0.425386	0.721284
H	-5.356661	0.615021	0.685707
C	-0.481264	2.356788	-1.201731
C	-0.237001	3.644286	-0.670569
H	-0.140527	3.786285	0.416258
C	-0.121874	4.756764	-1.522525
H	0.064625	5.752998	-1.091250
C	-0.250506	4.600695	-2.913904
H	-0.166377	5.473955	-3.579793
C	-0.488142	3.322672	-3.450273
H	-0.590707	3.188853	-4.538769
C	-0.597253	2.206405	-2.602714
H	-0.780176	1.215232	-3.043741
C	-1.310842	1.647834	1.437238
C	-0.614002	1.811958	2.651953
H	0.421263	1.462980	2.758193
C	-1.227714	2.448509	3.748196
H	-0.668228	2.565698	4.689431
C	-2.540011	2.935627	3.638914
H	-3.019201	3.434161	4.496142
C	-3.237545	2.791417	2.424302
H	-4.263941	3.178316	2.325298
C	-2.627677	2.155742	1.331629
H	-3.186106	2.053643	0.389607
C	-0.580389	-2.513146	-0.232374
H	-0.677451	-2.298743	0.853052
C	1.826942	-3.085385	-0.062229
H	1.537241	-3.322506	0.985851
H	1.799495	-4.040888	-0.624044
C	3.230186	-2.547059	-0.036138
C	4.619903	-0.674504	0.247049
C	5.749082	-1.516959	0.318295
H	6.741179	-1.062175	0.450766
C	5.608897	-2.901940	0.180869
C	4.324694	-3.425424	-0.014299
H	4.156019	-4.506289	-0.126155
C	-0.842092	-4.011354	-0.461509
H	-1.908213	-4.228619	-0.260214
H	-0.623884	-4.312119	-1.506969
H	-0.248481	-4.649385	0.219225
C	2.197761	1.411441	0.674185
C	1.283105	-0.699108	1.831059
H	0.903386	-2.114627	-1.612688
H	6.489158	-3.561871	0.211145
N	4.750143	0.726748	0.357939
C	4.981283	1.448264	-0.898884
H	4.364915	1.027893	-1.713514
H	4.705711	2.515392	-0.768854
H	6.052052	1.398311	-1.216031
C	5.591759	1.211883	1.450912
H	5.395895	0.629281	2.371774
H	6.686077	1.172810	1.224791
H	5.328261	2.271656	1.651457
C	1.857888	0.291962	-1.665317
O	2.091355	0.522061	-2.781009

4/1-fRSR-HCO3--EtOH2

Frequencies, energies and thermodynamic properties:

Lowest Vibrational Mode (1/cm) =	14.0408
E(RB-P86) (a.u.) =	-4959.53638672
Thermal correction to Enthalpy (a.u.) =	0.817729
Thermal correction to Gibbs Free Energy (a.u.) =	0.670154
Total Entropy (cal/Kmol) =	310.598
E(RPBE1PBE) (a.u.) =	-4958.89777614

Optimised cartesian coordinates (Angstrom):

Fe	-3.658429	-2.159339	-0.345692
Mn	0.520345	0.179672	0.605399
P	-1.596397	0.752524	0.078707
O	0.412878	2.143171	2.806152
O	-0.205293	-1.802751	2.637221

N	0.506368	-1.202968	-0.989016
N	1.417578	1.372649	-0.952094
C	-2.494498	-0.545991	-0.871775
C	-1.894676	-1.792489	-1.339161
C	-2.853356	-2.427851	-2.213879
H	-2.719866	-3.391650	-2.721163
C	-4.033807	-1.609255	-2.281390
H	-4.947274	-1.840403	-2.845839
C	-3.822776	-0.455858	-1.450810
H	-4.540165	0.358940	-1.289936
C	-3.414629	-2.681172	1.633304
H	-2.573222	-2.381821	2.271233
C	-3.461785	-3.856582	0.804643
H	-2.669128	-4.610815	0.708689
C	-4.720693	-3.858599	0.103766
H	-5.053531	-4.611008	-0.623829
C	-5.453914	-2.684202	0.503442
H	-6.444285	-2.385871	0.133943
C	-4.646150	-1.954657	1.446101
H	-4.910060	-1.002726	1.924649
C	-1.749032	2.250051	-1.026406
C	-1.468323	3.517660	-0.461515
H	-1.219224	3.596327	0.608957
C	-1.516246	4.679759	-1.248870
H	-1.312551	5.659848	-0.788258
C	-1.824523	4.594454	-2.620545
H	-1.860790	5.505801	-3.238339
C	-2.086209	3.338914	-3.194657
H	-2.327996	3.260248	-4.266797
C	-2.050988	2.174175	-2.403308
H	-2.278712	1.201538	-2.866054
C	-2.821574	1.214170	1.415196
C	-2.636168	0.780189	2.745784
H	-1.747243	0.195508	3.017526
C	-3.584108	1.085637	3.739824
H	-3.418490	0.738941	4.772363
C	-4.732836	1.830717	3.420893
H	-5.473466	2.070698	4.200339
C	-4.925146	2.274634	2.099702
H	-5.817755	2.865262	1.838010
C	-3.975671	1.973444	1.107663
H	-4.134023	2.345685	0.083692
C	-0.501566	-2.305010	-0.999133
H	-0.510278	-2.682711	0.043025
C	0.645740	-0.540447	-2.289534
H	1.205919	-1.163707	-3.024501
H	-0.363140	-0.383539	-2.737187
C	1.312351	0.808249	-2.186025
C	1.989362	2.622688	-0.859809
C	2.352997	3.342670	-2.026286
C	2.213152	2.758454	-3.287474
C	1.709985	1.455172	-3.369573
C	-0.070490	-3.479332	-1.898234
H	-0.743097	-4.348160	-1.749822
H	-0.091440	-3.218197	-2.976384
H	0.957173	-3.794389	-1.628995
C	0.519856	1.397158	1.893482
C	0.020528	-0.998008	1.795298
H	1.596076	0.940038	-4.334863
H	2.802271	4.339645	-1.927159
O	2.488109	-0.402463	0.989560
O	4.343621	-1.613562	1.196053
O	2.752217	-2.293101	-0.248179
H	1.461980	-1.680326	-0.729406
C	3.183704	-1.455570	0.630635
H	2.523217	3.304184	-4.192490
H	5.447119	-2.636103	0.628204
O	6.206656	-3.214677	0.275487
C	6.051953	-3.275451	-1.129624
H	6.481470	-2.367910	-1.635923
H	4.969433	-3.292604	-1.406167
C	6.751021	-4.516490	-1.681916
H	6.310821	-5.440183	-1.248863
H	6.662693	-4.572093	-2.787674
H	7.832065	-4.506735	-1.424085
H	5.105624	-0.370732	1.901683
O	5.759083	0.328200	2.241145

C	6.866319	0.267755	1.362160
H	6.713865	0.913588	0.453745
H	7.006405	-0.771488	0.975078
C	8.135941	0.730101	2.075596
H	8.359545	0.072961	2.943096
H	9.011840	0.717879	1.392577
H	8.015831	1.765791	2.460608
N	2.201723	3.183220	0.404142
C	2.226680	4.637189	0.499475
C	3.211034	2.548259	1.264294
H	1.409080	5.083371	-0.099390
H	2.071658	4.920701	1.561286
H	3.197678	5.090259	0.175513
H	3.007658	2.809968	2.322738
H	3.182668	1.444874	1.165612
H	4.233500	2.918215	1.004224

4/1-fRSR-HCO3--EtOH5

Frequencies, energies and thermodynamic properties:

Lowest Vibrational Mode (1/cm) =	10.0089
E(RB-P86) (a.u.) =	-5424.35370483
Thermal correction to Enthalpy (a.u.) =	1.073568
Thermal correction to Gibbs Free Energy (a.u.) =	0.883033
Total Entropy (cal/Kmol) =	401.014
E(RPBE1PBE) (a.u.) =	-5423.73545420

Optimised cartesian coordinates (Angstrom):

Fe	-3.714176	-2.434938	-1.176221
Mn	-0.204473	0.433550	0.670700
P	-2.419770	0.583492	0.273557
O	-0.784103	1.580703	3.326683
O	-0.334197	-2.201943	1.947925
N	0.065762	-0.356969	-1.265727
N	0.264745	2.236742	-0.425237
C	-2.996055	-0.509746	-1.091638
C	-2.124420	-1.355536	-1.901990
C	-2.921540	-1.880271	-2.986470
H	-2.570393	-2.564854	-3.768714
C	-4.267038	-1.391968	-2.850043
H	-5.111655	-1.637926	-3.507620
C	-4.321772	-0.556983	-1.682069
H	-5.212104	-0.035809	-1.308218
C	-3.283153	-3.504641	0.531742
H	-2.517194	-3.242221	1.272952
C	-3.076940	-4.317618	-0.637530
H	-2.130613	-4.786143	-0.939592
C	-4.322979	-4.388181	-1.357457
H	-4.491393	-4.915104	-2.306257
C	-5.301282	-3.620818	-0.629369
H	-6.346656	-3.461586	-0.926243
C	-4.659017	-3.072463	0.536642
H	-5.126705	-2.423534	1.288339
C	-2.997616	2.271334	-0.272789
C	-3.063193	3.296957	0.700994
H	-2.807115	3.074841	1.749263
C	-3.467675	4.594492	0.346783
H	-3.532557	5.376107	1.120705
C	-3.792031	4.895544	-0.990136
H	-4.110107	5.913114	-1.267448
C	-3.707974	3.889475	-1.967425
H	-3.956889	4.114897	-3.016789
C	-3.313720	2.584875	-1.612075
H	-3.271064	1.804553	-2.387267
C	-3.656857	0.246839	1.635365
C	-3.306427	-0.525305	2.763881
H	-2.283189	-0.906110	2.878750
C	-4.259108	-0.819690	3.756823
H	-3.963090	-1.421754	4.630592
C	-5.577470	-0.346598	3.638820
H	-6.322011	-0.576481	4.417507
C	-5.936534	0.430643	2.522288
H	-6.964434	0.814257	2.421413
C	-4.983658	0.729171	1.533064
H	-5.279114	1.355956	0.677702
C	-0.646298	-1.615605	-1.653797
H	-0.556195	-2.272103	-0.765667
C	0.030684	0.674370	-2.318059
H	0.815017	0.488410	-3.088340

H	-0.952509	0.641988	-2.838320
C	0.231336	2.066313	-1.775454
C	0.442047	3.515829	0.059706
C	0.475547	4.628691	-0.818196
C	0.409745	4.436791	-2.200132
C	0.314516	3.130285	-2.691413
C	0.039581	-2.340307	-2.825311
H	-0.446480	-3.321084	-3.002769
H	-0.017274	-1.761811	-3.770380
H	1.105903	-2.535495	-2.586813
C	-0.484269	1.173704	2.258457
C	-0.344530	-1.140282	1.420479
H	0.277798	2.920205	-3.770394
H	0.613817	5.637201	-0.407146
O	1.909201	0.244524	0.953187
O	4.097593	-0.126582	0.723667
O	2.683997	-0.725007	-0.939352
H	1.096118	-0.611494	-1.140154
C	2.911954	-0.204407	0.238957
H	0.463537	5.297265	-2.885385
H	3.068912	-0.120164	-2.509135
O	3.171221	0.155864	-3.462113
C	4.035751	1.287779	-3.488529
H	4.880708	1.152179	-2.771440
H	3.497254	2.218872	-3.172226
C	4.576712	1.480450	-4.901597
H	3.747971	1.627738	-5.626864
H	5.242053	2.367300	-4.956008
H	5.155652	0.589392	-5.224629
H	3.254216	-2.236633	-1.490657
O	3.436903	-3.144330	-1.867694
C	3.421276	-4.061808	-0.780185
H	2.405250	-4.125211	-0.310447
H	4.122304	-3.734743	0.026154
C	3.826579	-5.443929	-1.282044
H	4.843741	-5.414150	-1.727224
H	3.831238	-6.183459	-0.454369
H	3.125406	-5.805559	-2.064361
H	5.405837	0.314609	-0.231354
O	6.241213	0.660043	-0.676068
C	6.554145	1.900077	-0.060261
H	6.659306	1.786969	1.048460
H	5.739573	2.652578	-0.217986
C	7.857836	2.443737	-0.636279
H	7.771402	2.603486	-1.731977
H	8.126102	3.413187	-0.167045
H	8.693085	1.732555	-0.462644
H	4.874868	-1.517836	1.316488
O	5.398481	-2.300199	1.659641
C	6.753469	-1.880663	1.776476
H	6.911101	-1.284165	2.712730
H	7.028484	-1.209719	0.927803
C	7.672703	-3.097571	1.803198
H	7.592475	-3.675993	0.858635
H	8.731857	-2.792020	1.934531
H	7.405001	-3.775713	2.641458
H	2.252146	0.111517	2.659977
O	2.377659	0.040384	3.649259
C	2.764612	-1.291200	3.950323
H	3.658140	-1.603866	3.355030
H	1.952912	-2.023197	3.700603
C	3.085404	-1.396080	5.438596
H	2.202041	-1.120700	6.053955
H	3.387859	-2.428775	5.711348
H	3.914294	-0.708351	5.711126
N	0.584638	3.705595	1.436333
C	0.216066	5.003168	1.987394
C	1.776715	3.140545	2.080626
H	-0.736690	5.357792	1.549262
H	0.072095	4.888664	3.081664
H	0.997209	5.789892	1.835991
H	1.574997	2.963011	3.155860
H	2.045668	2.172783	1.619222
H	2.638566	3.848093	1.994402

4/1-fRSR-HCO3-
Frequencies, energies and thermodynamic properties:

```
 Lowest Vibrational Mode (1/cm) =                         19.5805
 E(RB-P86) (a.u.) =                                     -4649.64573054
 Thermal correction to Enthalpy (a.u.) =                  0.646804
 Thermal correction to Gibbs Free Energy (a.u.) =         0.526691
 Total Entropy (cal/Kmol) =                               252.799
 E(RPBE1PBE) (a.u.) =                                   -4649.00364237
Optimised cartesian coordinates (Angstrom):
 Fe     3.550536  -0.530858   0.687352
 Mn    -1.059482  -0.763875  -0.902403
 P      0.290137   0.821897  -0.040815
 O     -1.740193   1.127697  -3.065645
 O      0.896275  -1.858236  -2.788821
 N     -0.509553  -2.012739   0.691469
 N     -2.693100  -0.399534   0.465026
 C      1.635794   0.154006   1.029334
 C      1.758583  -1.249446   1.409576
 C      2.790800  -1.331477   2.417065
 H      3.141592  -2.250569   2.903494
 C      3.317024  -0.013115   2.651877
 H      4.130175   0.243066   3.344638
 C      2.617346   0.902919   1.792897
 H      2.789073   1.985373   1.732570
 C      3.885685  -0.975129  -1.298501
 H      3.104999  -1.148380  -2.050522
 C      4.469877  -1.980311  -0.450838
 H      4.220715  -3.050047  -0.452108
 C      5.420409  -1.334222   0.418589
 H      6.019588  -1.823989   1.198028
 C      5.425806   0.072259   0.104311
 H      6.031087   0.841793   0.602222
 C      4.475633   0.295074  -0.954413
 H      4.228270   1.262786  -1.409562
 C     -0.554036   2.050502   1.087573
 C     -1.461249   2.963832   0.497285
 H     -1.604882   2.961506  -0.595361
 C     -2.173565   3.880397   1.287882
 H     -2.862706   4.594513   0.808783
 C     -2.009285   3.888022   2.686937
 H     -2.569971   4.605245   3.307342
 C     -1.125180   2.973862   3.284568
 H     -0.988880   2.970163   4.378103
 C     -0.400522   2.062903   2.491035
 H      0.301416   1.366530   2.975122
 C      1.211987   2.005491  -1.160989
 C      1.498268   1.650709  -2.497440
 H      1.150772   0.688808  -2.898124
 C      2.232111   2.516042  -3.329550
 H      2.442642   2.220216  -4.369835
 C      2.690901   3.752142  -2.840849
 H      3.263259   4.430090  -3.493995
 C      2.406449   4.119618  -1.512924
 H      2.755046   5.088100  -1.119422
 C      1.669868   3.256116  -0.682785
 H      1.442101   3.568067   0.348361
 C      0.915348  -2.398721   0.868430
 H      1.271223  -2.636345  -0.154696
 C     -1.153606  -1.615123   1.941134
 H     -1.342354  -2.481636   2.619137
 H     -0.491645  -0.926139   2.519973
 C     -2.457306  -0.894441   1.710925
 C     -3.862406   0.299120   0.257798
 C     -4.743243   0.577774   1.334409
 C     -4.472201   0.084197   2.613308
 C     -3.321496  -0.689140   2.801842
 C      1.074848  -3.685910   1.702876
 H      2.123047  -4.046799   1.668772
 H      0.805930  -3.536562   2.769253
 H      0.424746  -4.477979   1.280846
 C     -1.550290   0.358813  -2.182840
 C      0.158164  -1.378484  -1.990840
 H     -3.067995  -1.117479   3.782949
 H     -5.670055   1.135099   1.144435
 O     -2.280266  -2.313587  -1.595603
 O     -3.254820  -4.310185  -1.834936
 O     -1.715958  -4.031397  -0.207613
 H     -1.058808  -2.962995   0.288904
 C     -2.429000  -3.587052  -1.218380
```

```
 H     -5.168933   0.276501   3.444470
 N     -4.171633   0.750002  -1.030177
 C     -5.034037   1.918293  -1.146562
 C     -4.450953  -0.260715  -2.059662
 H     -4.740109   2.697796  -0.416792
 H     -4.916939   2.339016  -2.167002
 H     -6.121170   1.689737  -1.006713
 H     -4.293693   0.186546  -3.062583
 H     -3.769867  -1.133310  -1.948766
 H     -5.516089  -0.599290  -1.992526
```

4/1-fRSR

```
Frequencies, energies and thermodynamic properties:
 Lowest Vibrational Mode (1/cm) =                        13.8385
 E(RB-P86) (a.u.) =                                    -4385.26260425
 Thermal correction to Enthalpy (a.u.) =                 0.616418
 Thermal correction to Gibbs Free Energy (a.u.) =        0.503822
 Total Entropy (cal/Kmol) =                              236.980
 E(RPBE1PBE) (a.u.) =                                  -4384.58040491
Optimised cartesian coordinates (Angstrom):
 Fe     3.437364  -0.806867   0.248761
 Mn    -1.068071  -0.316046  -1.289731
 P      0.136370   0.596981   0.282491
 O     -2.119549   2.319875  -2.164068
 O      0.869448  -0.007146  -3.460630
 N     -0.530827  -2.064560  -0.720910
 N     -2.906636  -1.035209  -0.458788
 C      1.497370  -0.498831   0.851510
 C      1.674111  -1.871880   0.395048
 C      2.703172  -2.467053   1.213178
 H      3.090788  -3.489407   1.115806
 C      3.167447  -1.486007   2.159692
 H      3.962062  -1.632808   2.903923
 C      2.435267  -0.268415   1.935564
 H      2.558863   0.668843   2.493687
 C      3.794220  -0.061709  -1.639562
 H      3.024532   0.172776  -2.386484
 C      4.439279  -1.336715  -1.471281
 H      4.254432  -2.237899  -2.071420
 C      5.351680  -1.233310  -0.360576
 H      5.980736  -2.041602   0.036004
 C      5.272685   0.109614   0.155990
 H      5.831668   0.503738   1.015387
 C      4.308484   0.833159  -0.631838
 H      4.002824   1.876890  -0.482985
 C     -0.894569   0.823251   1.818817
 C     -1.939103   1.779040   1.775396
 H     -2.080925   2.394175   0.871872
 C     -2.793572   1.954256   2.875277
 H     -3.593681   2.710352   2.830064
 C     -2.632154   1.163913   4.030570
 H     -3.305447   1.299090   4.891936
 C     -1.608787   0.202500   4.077117
 H     -1.475463  -0.420381   4.976179
 C     -0.742609   0.032196   2.979213
 H      0.064374  -0.713857   3.035799
 C      0.924185   2.273473   0.112264
 C      1.248647   2.780286  -1.165194
 H      1.009711   2.197303  -2.066095
 C      1.881259   4.029638  -1.297907
 H      2.125873   4.411606  -2.301868
 C      2.195570   4.790231  -0.157308
 H      2.688274   5.769853  -0.262959
 C      1.869686   4.296656   1.119184
 H      2.105271   4.888198   2.018316
 C      1.234866   3.049223   1.253637
 H      0.969851   2.683606   2.258152
 C      0.870741  -2.515790  -0.744043
 H      1.291166  -2.133979  -1.695905
 C     -1.310809  -2.666993   0.338782
 H     -1.468055  -3.766935   0.196933
 H     -0.799940  -2.586988   1.335714
 C     -2.664207  -2.025322   0.444048
 C     -4.178465  -0.507663  -0.533449
 C     -5.143421  -0.826627   0.457524
 H     -6.145640  -0.381540   0.412814
 C     -4.841322  -1.774210   1.441692
```

C	-3.599281	-2.424176	1.412353
H	-3.341931	-3.215706	2.131274
C	1.036782	-4.047689	-0.772897
H	2.090899	-4.316590	-0.988121
H	0.759483	-4.527543	0.188177
H	0.406571	-4.481754	-1.575965
C	-1.756708	1.245361	-1.820102
C	0.133043	-0.114385	-2.531452
H	-5.595868	-2.038221	2.199320
N	-4.488493	0.321255	-1.601014
C	-4.168617	-0.113895	-2.962932
H	-5.018758	-0.687463	-3.404471
H	-3.263791	-0.747230	-2.960909
H	-3.964659	0.767348	-3.603549
C	-5.672250	1.165149	-1.521723
H	-5.590249	1.960499	-2.289707
H	-5.737263	1.654411	-0.530626
H	-6.624177	0.610284	-1.709152

4/1s-fRSR-EtOH-alkox-EtOH

Frequencies, energies and thermodynamic properties:

Lowest Vibrational Mode (1/cm) =	13.0207
E(RB-P86) (a.u.) =	-4850.09429559
Thermal correction to Enthalpy (a.u.) =	0.870215
Thermal correction to Gibbs Free Energy (a.u.) =	0.721998
Total Entropy (cal/Kmol) =	311.949
E(RPBE1PBE) (a.u.) =	-4849.43560542

Optimised cartesian coordinates (Angstrom):

Fe	-2.787974	-2.481312	-0.767273
Mn	0.760292	0.481403	0.839688
P	-1.369809	0.716482	0.086718
O	0.062759	2.209285	3.122481
O	0.071939	-1.772802	2.581085
N	1.178927	-0.747360	-0.864440
N	1.545310	1.953809	-0.558083
C	-1.895691	-0.647940	-1.034194
C	-1.025723	-1.725021	-1.504349
C	-1.745848	-2.438264	-2.534629
H	-1.380701	-3.314208	-3.085033
C	-3.042227	-1.838488	-2.694577
H	-3.828452	-2.178162	-3.382041
C	-3.144272	-0.744611	-1.769241
H	-4.015143	-0.088433	-1.645820
C	-2.704234	-3.118519	1.191606
H	-2.026974	-2.724034	1.959334
C	-2.412545	-4.205731	0.295312
H	-1.481570	-4.787805	0.267412
C	-3.545546	-4.381032	-0.576904
H	-3.627452	-5.115200	-1.389578
C	-4.539850	-3.402536	-0.216364
H	-5.512500	-3.261556	-0.706564
C	-4.019478	-2.620206	0.874559
H	-4.524770	-1.779500	1.367480
C	-1.680953	2.247307	-0.935028
C	-1.674279	3.493069	-0.262061
H	-1.520124	3.526030	0.828553
C	-1.875494	4.689412	-0.969393
H	-1.886123	5.648620	-0.427374
C	-2.065121	4.664611	-2.364723
H	-2.222310	5.603295	-2.919303
C	-2.052962	3.434893	-3.043949
H	-2.199349	3.403736	-4.135484
C	-1.864250	2.232807	-2.334348
H	-1.880249	1.277636	-2.881279
C	-2.794932	0.843543	1.290354
C	-2.690335	0.337261	2.604092
H	-1.749826	-0.107073	2.954580
C	-3.787345	0.390073	3.483729
H	-3.682556	-0.008506	4.505360
C	-5.007135	0.949591	3.065458
H	-5.864743	0.991588	3.755557
C	-5.121401	1.463297	1.760795
H	-6.069404	1.911188	1.422397
C	-4.024143	1.415544	0.883556
H	-4.127681	1.841240	-0.126296
C	0.393164	-2.020197	-1.036513
H	0.344686	-2.456697	-0.019470

```
 C      1.236947   0.060147  -2.096930
 H      1.919103  -0.381667  -2.856521
 H      0.227346   0.067117  -2.563304
 C      1.632499   1.490948  -1.835289
 C      1.834188   3.283172  -0.335505
 C      2.113081   4.158981  -1.416388
 C      2.182458   3.666734  -2.720776
 C      1.965927   2.300250  -2.934586
 C      1.117386  -3.034667  -1.936991
 H      0.572649  -4.000295  -1.932475
 H      1.183473  -2.692767  -2.990455
 H      2.142025  -3.231715  -1.558240
 C      0.392825   1.564346   2.187799
 C      0.302873  -0.862634   1.855886
 H      3.533545  -0.517595   0.422382
 H      2.167169  -1.026373  -0.663224
 O      3.997967  -1.103441  -0.383405
 C      4.900217  -0.317674  -1.160628
 H      4.658373  -0.445128  -2.242942
 H      4.752205   0.763708  -0.930836
 H      2.021002   1.857381  -3.939771
 H      2.334798   5.214705  -1.213082
 C      6.358903  -0.713102  -0.922615
 H      6.496346  -1.797069  -1.118991
 H      7.039767  -0.147503  -1.593820
 H      6.663946  -0.513681   0.125945
 O      2.775927   0.176109   1.326135
 C      3.114900  -0.220205   2.644964
 C      4.627962  -0.184124   2.883265
 H      2.737866  -1.248971   2.874216
 H      2.613794   0.455533   3.381281
 H      4.864639  -0.490809   3.924474
 H      5.157324  -0.875151   2.195196
 H      5.037812   0.835813   2.724900
 H      4.257588  -2.763750  -0.489406
 O      4.410650  -3.743441  -0.648596
 C      4.434933  -4.394731   0.612231
 H      3.455531  -4.296091   1.147355
 H      5.203425  -3.941971   1.289451
 C      4.751645  -5.873866   0.415583
 H      5.735800  -6.000086  -0.083261
 H      4.784006  -6.407295   1.388154
 H      3.983083  -6.361277  -0.220961
 H      2.431868   4.338076  -3.557301
 N      1.846151   3.763706   0.975519
 C      1.608618   5.186355   1.184388
 C      2.870895   3.219452   1.874633
 H      0.767558   5.539176   0.556886
 H      1.334569   5.341549   2.248243
 H      2.504780   5.822803   0.975910
 H      2.536820   3.320274   2.927095
 H      3.032140   2.146578   1.645291
 H      3.831468   3.779911   1.755560
```

4/1s-fRSR-EtOH-alkox

Frequencies, energies and thermodynamic properties:

```
 Lowest Vibrational Mode (1/cm) =                          8.8855
 E(RB-P86) (a.u.) =                                    -4695.15277805
 Thermal correction to Enthalpy (a.u.) =                   0.786792
 Thermal correction to Gibbs Free Energy (a.u.) =          0.651569
 Total Entropy (cal/Kmol) =                                284.601
 E(RPBE1PBE) (a.u.) =                                  -4694.49002942
```

Optimised cartesian coordinates (Angstrom):

```
 Fe    -3.574342   1.136540   0.742642
 Mn     0.967886   0.405229  -0.895863
 P     -0.688920  -0.881691  -0.023842
 O      1.276779  -1.534890  -3.089394
 O     -0.861604   1.791890  -2.715503
 N      0.701844   1.747980   0.746103
 N      2.452958  -0.340138   0.510495
 C     -1.841415   0.072263   1.056738
 C     -1.669737   1.473301   1.440152
 C     -2.648518   1.762158   2.463272
 H     -2.797147   2.731547   2.955282
 C     -3.427914   0.579098   2.706223
 H     -4.267703   0.494085   3.408839
 C     -2.943846  -0.458562   1.838850
```

H	-3.336666	-1.481661	1.782992
C	-3.870252	1.592750	-1.246902
H	-3.097981	1.568079	-2.025793
C	-4.188751	2.724634	-0.417603
H	-3.710228	3.712275	-0.461242
C	-5.228945	2.328908	0.497068
H	-5.677866	2.958957	1.276624
C	-5.555103	0.950977	0.230220
H	-6.296593	0.347962	0.771335
C	-4.713613	0.494717	-0.845236
H	-4.700157	-0.516232	-1.272860
C	-0.123511	-2.270240	1.089125
C	0.545183	-3.359366	0.479415
H	0.686040	-3.373078	-0.613531
C	1.019722	-4.431628	1.252033
H	1.522528	-5.279019	0.758962
C	0.852826	-4.425671	2.650449
H	1.225062	-5.266832	3.256527
C	0.209159	-3.339603	3.266836
H	0.074914	-3.323667	4.360300
C	-0.277534	-2.268531	2.492034
H	-0.797219	-1.436531	2.991513
C	-1.851637	-1.830665	-1.139612
C	-2.048495	-1.447316	-2.484062
H	-1.491080	-0.601683	-2.906845
C	-2.961219	-2.138876	-3.301894
H	-3.096993	-1.824290	-4.348913
C	-3.691873	-3.225367	-2.790434
H	-4.404710	-3.767017	-3.432310
C	-3.499013	-3.621112	-1.454245
H	-4.059598	-4.475700	-1.042766
C	-2.583865	-2.933580	-0.638417
H	-2.432331	-3.271695	0.398169
C	-0.618418	2.440945	0.912372
H	-0.901995	2.750915	-0.113210
C	1.220764	1.180299	2.002214
H	1.603211	1.966938	2.688772
H	0.382378	0.683444	2.537558
C	2.299639	0.153360	1.769007
C	3.409072	-1.313113	0.313105
C	4.124195	-1.863695	1.408542
C	3.932261	-1.361419	2.696697
C	3.025270	-0.310533	2.879467
C	-0.499091	3.715367	1.765262
H	-1.436743	4.303885	1.712882
H	-0.298351	3.493131	2.833110
H	0.321922	4.354476	1.381286
C	1.222267	-0.768441	-2.189307
C	-0.162064	1.214490	-1.949425
H	3.081925	2.660001	-0.258351
H	1.396582	2.457133	0.440477
O	3.242237	3.369633	0.473107
C	4.458054	3.116782	1.156751
H	4.443794	3.713369	2.098362
H	4.540523	2.045731	1.472476
H	2.849659	0.135341	3.869384
H	4.874714	-2.644081	1.227872
C	5.694845	3.498926	0.336681
H	5.649889	4.566969	0.035524
H	6.628375	3.341693	0.918910
H	5.763918	2.886648	-0.587705
O	2.464265	1.762847	-1.366938
C	2.395621	2.646339	-2.467060
C	2.664238	2.005384	-3.834269
H	3.151486	3.456795	-2.304415
H	1.408212	3.173969	-2.515798
H	2.591534	2.768617	-4.639623
H	3.679525	1.558539	-3.881479
H	1.929491	1.204384	-4.057222
H	4.509180	-1.765025	3.543456
N	3.666374	-1.754409	-0.984551
C	4.251118	-3.078012	-1.155416
C	4.190434	-0.768477	-1.937671
H	5.352101	-3.107468	-0.958960
H	3.752483	-3.815011	-0.496472
H	4.095144	-3.394862	-2.207100
H	3.959913	-1.093694	-2.972262

H 3.716524 0.220164 -1.762647
H 5.301092 -0.681705 -1.835894

4/1s-fRSR-alkox
Frequencies, energies and thermodynamic properties:
Lowest Vibrational Mode (1/cm) = 21.2526
E(RB-P86) (a.u.) = -4540.21614648
Thermal correction to Enthalpy (a.u.) = 0.701896
Thermal correction to Gibbs Free Energy (a.u.) = 0.581268
Total Entropy (cal/Kmol) = 253.883
E(RPBE1PBE) (a.u.) = -4539.54052681
Optimised cartesian coordinates (Angstrom):

Fe	3.517607	0.372781	-0.745438
Mn	-1.088094	1.005414	0.694948
P	0.221052	-0.766086	0.166233
O	-1.962803	-0.315150	3.179163
O	0.953426	2.246715	2.388631
N	-0.476578	1.933002	-1.113201
N	-2.683576	0.406788	-0.667101
C	1.587503	-0.303101	-0.989387
C	1.763306	1.010940	-1.608435
C	2.805780	0.883456	-2.600781
H	3.191140	1.691332	-3.235510
C	3.287626	-0.470853	-2.595525
H	4.098470	-0.868885	-3.220291
C	2.550765	-1.200799	-1.601531
H	2.688705	-2.260751	-1.353291
C	3.862730	1.090394	1.157360
H	3.087690	1.381402	1.877458
C	4.462301	1.955159	0.175974
H	4.232144	3.018569	0.025568
C	5.401201	1.176761	-0.590873
H	6.007131	1.540305	-1.431650
C	5.383272	-0.170576	-0.080204
H	5.973621	-1.013192	-0.464591
C	4.430328	-0.225184	0.997847
H	4.165758	-1.114970	1.583770
C	-0.629281	-2.161744	-0.738833
C	-1.549303	-2.947018	-0.002542
H	-1.709769	-2.741159	1.068437
C	-2.251766	-3.994929	-0.619187
H	-2.952405	-4.605083	-0.026855
C	-2.062626	-4.264750	-1.988661
H	-2.613836	-5.086469	-2.472889
C	-1.167249	-3.478147	-2.732607
H	-1.013475	-3.679038	-3.805038
C	-0.453969	-2.433482	-2.112628
H	0.255970	-1.839188	-2.708030
C	1.102272	-1.747290	1.491937
C	1.336517	-1.192634	2.768961
H	0.973943	-0.184753	3.009440
C	2.037766	-1.918144	3.749882
H	2.207241	-1.466909	4.740532
C	2.515393	-3.210606	3.471635
H	3.062673	-3.778312	4.240911
C	2.280705	-3.777309	2.205368
H	2.642460	-4.792674	1.977022
C	1.576270	-3.054526	1.226990
H	1.384091	-3.523533	0.249698
C	0.962316	2.273604	-1.307106
H	1.290509	2.674704	-0.327413
C	-1.117453	1.361532	-2.304880
H	-1.330235	2.130625	-3.081535
H	-0.409253	0.643932	-2.775414
C	-2.393173	0.623758	-1.976386
C	-3.858393	-0.244158	-0.364452
C	-4.652517	-0.823319	-1.387981
C	-4.308425	-0.634937	-2.728557
C	-3.180838	0.137686	-3.034354
C	1.175794	3.379399	-2.354786
H	2.231223	3.717979	-2.348924
H	0.935270	3.041769	-3.383480
H	0.542298	4.258156	-2.116655
C	-1.680579	0.218441	2.160194
C	0.165602	1.709866	1.679543
H	-1.024630	2.757603	-0.757241
H	-2.887833	0.345353	-4.074079

```
 H     -5.573842  -1.359203  -1.124988
 N     -4.260435  -0.304239   0.968147
 C     -5.193463  -1.349502   1.363852
 H     -5.162425  -1.446716   2.468495
 H     -6.253072  -1.132525   1.077622
 H     -4.899963  -2.323200   0.925475
 C     -4.500368   0.978589   1.643081
 H     -5.545257   1.327806   1.450765
 H     -4.368905   0.856118   2.737072
 H     -3.775711   1.733265   1.269066
 H     -4.938934  -1.054708  -3.528023
 O     -2.128341   2.761442   0.661277
 C     -2.127500   3.680678   1.716761
 H     -1.091353   3.980791   2.028610
 H     -2.596813   3.243527   2.642150
 C     -2.901176   4.949838   1.333463
 H     -3.955837   4.712539   1.076472
 H     -2.907391   5.684975   2.167407
 H     -2.440939   5.438689   0.447741
```

```
4/1s-fRSR
Frequencies, energies and thermodynamic properties:
 Lowest Vibrational Mode (1/cm) =                          21.2411
 E(RB-P86) (a.u.) =                                      -4540.20006328
 Thermal correction to Enthalpy (a.u.) =                   0.701182
 Thermal correction to Gibbs Free Energy (a.u.) =          0.574539
 Total Entropy (cal/Kmol) =                                266.543
 E(RPBE1PBE) (a.u.) =                                    -4539.52636272
Optimised cartesian coordinates (Angstrom):
 Fe    -3.457553   1.124679   0.556150
 Mn     1.034886   0.253070  -0.866676
 P     -0.575626  -0.932374   0.011252
 O      1.656027  -1.898151  -2.809647
 O     -0.561695   1.485986  -2.983901
 N      0.800259   1.646503   0.481513
 N      2.843088   0.072207   0.240975
 C     -1.746678   0.078433   0.995810
 C     -1.557921   1.497892   1.272703
 C     -2.556150   1.879891   2.242403
 H     -2.701575   2.888303   2.650669
 C     -3.358872   0.728072   2.561426
 H     -4.213170   0.708089   3.251673
 C     -2.871630  -0.383219   1.789791
 H     -3.275220  -1.404045   1.805001
 C     -3.670142   1.457551  -1.468059
 H     -2.867483   1.391754  -2.213983
 C     -4.028469   2.635288  -0.723753
 H     -3.552080   3.621413  -0.807570
 C     -5.104395   2.288986   0.170119
 H     -5.589289   2.963237   0.888812
 C     -5.413521   0.895129  -0.025188
 H     -6.175896   0.321465   0.518887
 C     -4.525366   0.379841  -1.034899
 H     -4.491895  -0.654514  -1.400840
 C      0.150741  -2.100798   1.267847
 C      0.995082  -3.134680   0.793568
 H      1.160611  -3.255103  -0.289203
 C      1.619057  -4.015250   1.691288
 H      2.264217  -4.820846   1.305971
 C      1.424135  -3.867727   3.078685
 H      1.916746  -4.556797   3.782951
 C      0.598837  -2.837215   3.559085
 H      0.440406  -2.714887   4.642518
 C     -0.036711  -1.957969   2.660757
 H     -0.693597  -1.165900   3.050794
 C     -1.652130  -2.073313  -0.985464
 C     -1.879123  -1.824994  -2.356825
 H     -1.395681  -0.969939  -2.850329
 C     -2.725853  -2.664461  -3.102996
 H     -2.891113  -2.458289  -4.172426
 C     -3.354012  -3.763580  -2.490299
 H     -4.014492  -4.421873  -3.076733
 C     -3.127452  -4.023147  -1.126323
 H     -3.608926  -4.886134  -0.639318
 C     -2.279133  -3.187064  -0.379244
 H     -2.096894  -3.413007   0.683133
 C     -0.474967   2.375820   0.635567
```

```
 H     -0.805075   2.604722  -0.398116
 C      1.461421   1.363262   1.747492
 H      1.812475   2.284420   2.277067
 H      0.770216   0.865546   2.477144
 C      2.651628   0.469179   1.529707
 C      3.997419  -0.602331  -0.089461
 C      4.847605  -1.096936   0.937692
 C      4.579422  -0.767873   2.269355
 C      3.495025   0.070912   2.576737
 C     -0.323544   3.730380   1.354811
 H     -1.259201   4.320157   1.274409
 H     -0.088222   3.620070   2.433426
 H      0.487647   4.310300   0.870177
 C      1.458752  -1.026178  -2.033456
 C      0.020251   0.956622  -2.092505
 H      1.881972   2.780983  -0.447655
 O      2.420684   3.455472  -0.969587
 C      3.538963   3.853192  -0.188761
 H      4.204647   2.983540   0.045689
 H      3.216137   4.274750   0.797112
 H      3.291753   0.401051   3.605709
 H      5.747934  -1.670256   0.681966
 C      4.336799   4.909968  -0.945765
 H      5.211952   5.247996  -0.352921
 H      4.707872   4.508838  -1.912447
 H      3.703008   5.794860  -1.165702
 N      4.304658  -0.759669  -1.424654
 C      5.298081  -1.744981  -1.827354
 H      5.154434  -1.972775  -2.902591
 H      6.347400  -1.387958  -1.691741
 H      5.166812  -2.687597  -1.261772
 C      4.140491   0.350743  -2.366679
 H      5.139139   0.778769  -2.618858
 H      3.662582   0.004988  -3.306128
 H      3.517057   1.151152  -1.928547
 H      5.251383  -1.125706   3.065354

4/2-fRSR
Frequencies, energies and thermodynamic properties:
 Lowest Vibrational Mode (1/cm) =                          21.3167
 E(RB-P86) (a.u.) =                                     -4386.42413055
 Thermal correction to Enthalpy (a.u.) =                   0.632601
 Thermal correction to Gibbs Free Energy (a.u.) =          0.519997
 Total Entropy (cal/Kmol) =                                236.994
 E(RPBE1PBE) (a.u.) =                                   -4385.74439566
Optimised cartesian coordinates (Angstrom):
 Fe     3.416965  -0.659279   0.400294
 Mn    -1.038766  -0.427986  -1.560406
 P      0.024956   0.497495   0.210701
 O     -2.033281   2.231719  -2.420633
 O      1.162124   0.018897  -3.446054
 N     -0.379834  -2.261485  -0.913926
 N     -2.753074  -1.097138  -0.454634
 C      1.410122  -0.528228   0.840956
 C      1.736457  -1.863401   0.350730
 C      2.735864  -2.405550   1.240749
 H      3.212003  -3.389913   1.144469
 C      3.040908  -1.430808   2.255157
 H      3.779621  -1.544136   3.060397
 C      2.235268  -0.266080   2.007756
 H      2.237526   0.654249   2.605916
 C      3.882081   0.231564  -1.401031
 H      3.163091   0.484883  -2.190035
 C      4.570161  -1.024547  -1.267858
 H      4.472702  -1.888285  -1.939231
 C      5.388419  -0.961661  -0.083129
 H      6.022258  -1.769026   0.307756
 C      5.207707   0.337918   0.513716
 H      5.681050   0.693565   1.438881
 C      4.274133   1.074603  -0.298477
 H      3.909248   2.091807  -0.105442
 C     -1.059963   0.739357   1.707786
 C     -2.070657   1.727763   1.632505
 H     -2.175226   2.337176   0.720154
 C     -2.934203   1.951837   2.717301
 H     -3.706077   2.734996   2.647814
 C     -2.813321   1.180450   3.889260
```

```
 H     -3.490555   1.356162   4.740130
 C     -1.823618   0.185861   3.966350
 H     -1.721441  -0.423515   4.878602
 C     -0.950857  -0.034118   2.883009
 H     -0.172301  -0.807966   2.963266
 C      0.770018   2.205559   0.073627
 C      1.123077   2.746131  -1.181770
 H      0.925040   2.181876  -2.102916
 C      1.734257   4.010359  -1.272422
 H      1.999419   4.415237  -2.262067
 C      2.001724   4.754605  -0.110201
 H      2.478730   5.744950  -0.182506
 C      1.647813   4.228851   1.145904
 H      1.845345   4.805566   2.063656
 C      1.032596   2.968240   1.236558
 H      0.744782   2.581852   2.226502
 C      1.053747  -2.545800  -0.845499
 H      1.501666  -2.117106  -1.766336
 C     -1.094729  -2.748301   0.239782
 H     -1.206359  -3.865860   0.257591
 H     -0.582484  -2.521533   1.222055
 C     -2.471229  -2.157257   0.353583
 C     -4.007499  -0.534107  -0.379546
 C     -4.937118  -0.954287   0.598308
 C     -4.619117  -2.015296   1.455248
 C     -3.380821  -2.647976   1.309237
 C      1.369051  -4.058923  -0.877244
 H      2.456583  -4.239463  -1.006252
 H      1.053132  -4.576818   0.052472
 H      0.838974  -4.528391  -1.731029
 C     -1.714834   1.149223  -2.069788
 C      0.320324  -0.144742  -2.627659
 H     -1.547932  -1.713967  -2.625303
 H     -1.893256  -1.063298  -2.989176
 H     -5.928200  -0.482801   0.640573
 H     -3.091962  -3.502470   1.939115
 N     -4.343454   0.466064  -1.302856
 C     -4.517045   0.052362  -2.697860
 H     -5.567071  -0.278905  -2.890937
 H     -4.282539   0.895087  -3.380541
 H     -3.844510  -0.789013  -2.937940
 C     -5.309297   1.477882  -0.895477
 H     -5.206230   2.354966  -1.567987
 H     -6.372040   1.133343  -0.957449
 H     -5.104242   1.812468   0.139577
 H     -5.347762  -2.363178   2.204243
```

4/2s-fRSR

Frequencies, energies and thermodynamic properties:

```
 Lowest Vibrational Mode (1/cm) =                          22.1081
 E(RB-P86) (a.u.) =                                     -4541.36832638
 Thermal correction to Enthalpy (a.u.) =                   0.717356
 Thermal correction to Gibbs Free Energy (a.u.) =          0.591853
 Total Entropy (cal/Kmol) =                                264.143
 E(RPBE1PBE) (a.u.) =                                   -4540.69377140
```

Optimised cartesian coordinates (Angstrom):

```
 Fe    -3.458882   0.921764   0.622173
 Mn     1.075950   0.457442  -1.112495
 P     -0.400063  -0.854243   0.024294
 O      1.546981  -1.760037  -3.022246
 O     -0.890847   1.475447  -3.035935
 N      0.746496   1.959623   0.297554
 N      2.714474   0.119535   0.217172
 C     -1.611139   0.105259   1.009118
 C     -1.572433   1.553896   1.181126
 C     -2.548854   1.882915   2.193267
 H     -2.786367   2.893428   2.549232
 C     -3.194030   0.674964   2.634456
 H     -3.998765   0.607094   3.379043
 C     -2.629185  -0.423832   1.900353
 H     -2.910407  -1.479563   2.005393
 C     -3.856874   1.067667  -1.397308
 H     -3.111565   1.019462  -2.200871
 C     -4.274885   2.262502  -0.714308
 H     -3.909102   3.279429  -0.910152
 C     -5.238834   1.888395   0.289221
 H     -5.733137   2.568987   0.995439
```

```
 C     -5.418346   0.459849   0.223016
 H     -6.074613  -0.137811   0.869904
 C     -4.561710  -0.048526  -0.816851
 H     -4.450498  -1.101124  -1.107873
 C      0.388252  -1.990510   1.274324
 C      1.183879  -3.053827   0.783809
 H      1.295811  -3.200191  -0.302818
 C      1.820700  -3.938125   1.669898
 H      2.424350  -4.769246   1.271600
 C      1.687238  -3.764670   3.060944
 H      2.186940  -4.458084   3.755827
 C      0.912526  -2.702361   3.556749
 H      0.801931  -2.557968   4.643366
 C      0.265439  -1.820167   2.669967
 H     -0.350083  -1.002288   3.074509
 C     -1.461249  -2.076750  -0.906407
 C     -1.754934  -1.892232  -2.274842
 H     -1.325259  -1.044261  -2.824620
 C     -2.601840  -2.787463  -2.953653
 H     -2.815909  -2.627124  -4.022279
 C     -3.168149  -3.881225  -2.276012
 H     -3.829544  -4.582557  -2.809075
 C     -2.877397  -4.078343  -0.913745
 H     -3.309567  -4.935542  -0.373189
 C     -2.027355  -3.187182  -0.236101
 H     -1.796486  -3.368292   0.824990
 C     -0.616570   2.512433   0.463905
 H     -1.005370   2.664707  -0.564155
 C      1.350501   1.619101   1.578293
 H      1.649344   2.518646   2.173284
 H      0.642246   1.066788   2.254285
 C      2.556748   0.736513   1.421998
 C      3.801193  -0.707037   0.043644
 C      4.662789  -1.012939   1.121931
 C      4.466701  -0.405760   2.367070
 C      3.417016   0.508067   2.511295
 C     -0.607969   3.904035   1.135761
 H     -1.610670   4.375815   1.082593
 H     -0.317920   3.855295   2.206108
 H      0.110868   4.559880   0.604767
 C      1.434041  -0.884164  -2.239904
 C     -0.155711   1.035314  -2.219040
 H      2.231937   1.215325  -2.120611
 H      2.019481   1.854622  -1.631251
 H      1.587029   3.163828  -0.365183
 O      2.103779   3.897491  -0.910104
 C      3.353297   4.147594  -0.294213
 H      3.227157   4.503296   0.761765
 H      3.977353   3.217997  -0.230508
 H      3.235129   1.030765   3.461870
 H      5.517263  -1.683151   0.956840
 C      4.119283   5.206631  -1.083919
 H      3.536915   6.150962  -1.136220
 H      5.099613   5.429078  -0.612580
 H      4.304470   4.865146  -2.124482
 N      4.034410  -1.239949  -1.231658
 C      4.671153  -2.547660  -1.322366
 H      4.458673  -2.974404  -2.324905
 H      5.782824  -2.512771  -1.202933
 H      4.252620  -3.233981  -0.561551
 C      4.496544  -0.314860  -2.270224
 H      5.612505  -0.259621  -2.288327
 H      4.145845  -0.646962  -3.269661
 H      4.112895   0.702915  -2.083313
 H      5.149195  -0.619476   3.204501
```

```
4/3-fRSR
Frequencies, energies and thermodynamic properties:
 Lowest Vibrational Mode (1/cm) =                          19.6732
 E(RB-P86) (a.u.) =                                      -4386.45866100
 Thermal correction to Enthalpy (a.u.) =                   0.637322
 Thermal correction to Gibbs Free Energy (a.u.) =          0.525625
 Total Entropy (cal/Kmol) =                                235.085
 E(RPBE1PBE) (a.u.) =                                    -4385.77799462
Optimised cartesian coordinates (Angstrom):
 Fe     3.406489  -0.682557   0.369539
 Mn    -1.057548  -0.393765  -1.510524
```

P	0.026007	0.551591	0.256095
O	-2.017305	2.214967	-2.477957
O	1.014455	-0.113726	-3.561606
N	-0.403773	-2.315405	-0.825931
N	-2.776853	-1.068136	-0.440365
C	1.422131	-0.485954	0.875882
C	1.714801	-1.844213	0.416200
C	2.730702	-2.384942	1.291909
H	3.186488	-3.379866	1.212781
C	3.082346	-1.387218	2.265124
H	3.846293	-1.492442	3.047185
C	2.288125	-0.217942	2.009281
H	2.325473	0.720543	2.577412
C	3.815817	0.124540	-1.481129
H	3.070527	0.345289	-2.256237
C	4.504999	-1.127869	-1.313269
H	4.385090	-2.020402	-1.942158
C	5.363221	-1.014874	-0.161572
H	6.006441	-1.806943	0.244813
C	5.206365	0.310518	0.381091
H	5.709786	0.705185	1.273885
C	4.248596	1.013936	-0.431882
H	3.893030	2.039946	-0.272218
C	-0.995009	0.824542	1.797523
C	-2.051642	1.764199	1.707190
H	-2.192374	2.338007	0.776209
C	-2.918833	1.978573	2.790683
H	-3.725872	2.723955	2.704720
C	-2.761095	1.241912	3.981122
H	-3.443182	1.407096	4.830275
C	-1.728427	0.293702	4.075672
H	-1.596106	-0.289370	5.001410
C	-0.850831	0.086735	2.993160
H	-0.038123	-0.649350	3.093558
C	0.808943	2.239317	0.079100
C	1.163184	2.720577	-1.200228
H	0.951496	2.111672	-2.090442
C	1.789404	3.971747	-1.347985
H	2.057211	4.330748	-2.354687
C	2.068483	4.763215	-0.219389
H	2.556460	5.744064	-0.336040
C	1.712648	4.296923	1.059533
H	1.920256	4.911447	1.950324
C	1.084308	3.047442	1.206920
H	0.796430	2.704503	2.213102
C	1.074053	-2.583061	-0.752685
H	1.472056	-2.161701	-1.697133
C	-1.137425	-2.687173	0.397680
H	-1.189577	-3.787867	0.550436
H	-0.575466	-2.278955	1.265961
C	-2.528140	-2.103963	0.411829
C	-4.047337	-0.531492	-0.433324
C	-5.006897	-0.939041	0.529161
H	-6.005473	-0.482402	0.521966
C	-4.712523	-1.966168	1.427904
C	-3.458790	-2.587991	1.343461
H	-3.183737	-3.424363	2.002919
C	1.386009	-4.088874	-0.743165
H	2.473409	-4.257522	-0.873952
H	1.078642	-4.581090	0.201986
H	0.871444	-4.594692	-1.586252
C	-1.687036	1.152683	-2.063738
C	0.225627	-0.210212	-2.674892
H	-0.767472	-2.874899	-1.609339
H	-1.658082	-1.221278	-2.727724
H	-5.464682	-2.302887	2.158124
N	-4.378686	0.425152	-1.396448
C	-4.366440	-0.004499	-2.797267
H	-5.311346	-0.540001	-3.062127
H	-3.493832	-0.663382	-2.974018
H	-4.263066	0.881219	-3.456142
C	-5.451646	1.362211	-1.094824
H	-5.366992	2.226278	-1.785897
H	-5.357879	1.741240	-0.058448
H	-6.475568	0.930055	-1.225343

4/3-fRSS

Frequencies, energies and thermodynamic properties:

Lowest Vibrational Mode (1/cm) =	15.2991
E(RB-P86) (a.u.) =	-4386.45301587
Thermal correction to Enthalpy (a.u.) =	0.637294
Thermal correction to Gibbs Free Energy (a.u.) =	0.524898
Total Entropy (cal/Kmol) =	236.556
E(RPBE1PBE) (a.u.) =	-4385.77160807

Optimised cartesian coordinates (Angstrom):

Fe	-3.264417	-0.765039	-0.263134
Mn	1.588741	0.355371	-1.234766
P	-0.061227	0.860637	0.267591
N	0.268644	-1.082256	-2.147921
N	2.407347	-1.498909	-0.542936
C	-1.740337	0.623780	-0.486662
C	-2.007612	-0.054211	-1.751997
C	-3.399539	0.159839	-2.076981
H	-3.915374	-0.234780	-2.963142
C	-3.994680	0.970272	-1.049965
H	-5.042647	1.295721	-1.009085
C	-2.985201	1.242551	-0.064927
H	-3.127920	1.843530	0.841925
C	-2.759062	-2.259428	1.074317
H	-1.783613	-2.408553	1.556052
C	-3.190092	-2.827787	-0.178095
H	-2.606575	-3.496883	-0.823662
C	-4.530443	-2.369945	-0.436061
H	-5.137637	-2.613066	-1.318180
C	-4.929060	-1.521374	0.659024
H	-5.891669	-1.001474	0.753871
C	-3.833074	-1.451532	1.591259
H	-3.813372	-0.876583	2.525564
C	-0.089880	2.684225	0.659505
C	0.646437	3.177413	1.761351
H	1.165849	2.476112	2.433019
C	0.721879	4.558588	2.014571
H	1.297337	4.922614	2.881069
C	0.065563	5.471324	1.168822
H	0.120689	6.553162	1.369746
C	-0.659534	4.991567	0.063008
H	-1.174895	5.697213	-0.608536
C	-0.733155	3.610317	-0.192838
H	-1.298643	3.252237	-1.066198
C	-0.218271	0.170425	2.005513
C	0.726454	-0.790985	2.422611
H	1.515003	-1.104542	1.723129
C	0.677186	-1.337434	3.720268
H	1.423930	-2.087484	4.026297
C	-0.316024	-0.919733	4.621725
H	-0.356693	-1.342623	5.638268
C	-1.253756	0.053146	4.222816
H	-2.027087	0.396139	4.929044
C	-1.203038	0.595917	2.927891
H	-1.932473	1.369073	2.643075
C	-1.067313	-0.749188	-2.724716
H	-1.553727	-1.716095	-2.998603
C	0.179557	-2.215042	-1.209211
H	-0.531934	-1.919703	-0.406671
H	-0.246229	-3.122722	-1.695317
C	1.516793	-2.531822	-0.602962
C	3.641500	-1.754802	0.022798
C	3.906077	-2.999884	0.653090
H	4.888752	-3.173113	1.111077
C	2.957383	-4.024190	0.625725
C	1.748432	-3.799415	-0.049896
H	0.978183	-4.580094	-0.132407
C	-0.908310	0.047742	-4.031178
H	-1.890920	0.139773	-4.533648
H	-0.511736	1.064043	-3.853821
H	-0.226145	-0.483302	-4.728593
C	1.201080	1.747683	-2.210784
O	1.083771	2.697876	-2.913987
C	2.750947	1.396114	-0.421430
O	3.483401	2.180471	0.084038
H	2.476704	0.096426	-2.520346
H	0.862372	-1.370965	-2.938417
N	4.622761	-0.764625	-0.011360
C	5.667695	-0.790283	1.002765

```
 H      6.139050   0.213305   1.044541
 H      6.478683  -1.531471   0.792583
 H      5.239105  -1.009054   2.000294
 C      5.095869  -0.307953  -1.319770
 H      5.875049  -0.997316  -1.727819
 H      5.533906   0.706044  -1.222301
 H      4.239554  -0.241835  -2.019561
 H      3.174395  -4.998145   1.091102
```

```
4/3-mRSR
Frequencies, energies and thermodynamic properties:
 Lowest Vibrational Mode (1/cm) =                          20.9601
 E(RB-P86) (a.u.) =                                       -4386.46181056
 Thermal correction to Enthalpy (a.u.) =                   0.637269
 Thermal correction to Gibbs Free Energy (a.u.) =          0.524308
 Total Entropy (cal/Kmol) =                                237.747
 E(RPBE1PBE) (a.u.) =                                     -4385.77844573
Optimised cartesian coordinates (Angstrom):
 Fe    -2.081762  -2.457931   0.011085
 Mn     1.300483   0.551247  -0.258691
 P     -0.869968   0.914414   0.001539
 O      1.821413   2.757491   1.612689
 N      0.932636  -1.154322  -1.541940
 N      3.191841  -0.384855  -0.266837
 C     -1.927505  -0.478079  -0.574476
 C     -1.534301  -1.384226  -1.640963
 C     -2.662347  -2.236354  -1.937303
 H     -2.680189  -3.031747  -2.695209
 C     -3.738087  -1.886661  -1.048796
 H     -4.720298  -2.375889  -1.000801
 C     -3.285594  -0.816586  -0.199868
 H     -3.865548  -0.337840   0.599801
 C     -0.594218  -3.002925   1.338140
 H      0.314142  -2.415850   1.533611
 C     -0.758317  -4.009951   0.321453
 H      0.013812  -4.363978  -0.375109
 C     -2.120257  -4.477235   0.375845
 H     -2.569528  -5.234339  -0.280693
 C     -2.795264  -3.760918   1.428280
 H     -3.849952  -3.876352   1.711700
 C     -1.853794  -2.846326   2.019984
 H     -2.060712  -2.141631   2.835774
 C     -1.623919   2.364389  -0.907695
 C     -1.077599   3.647411  -0.663013
 H     -0.264606   3.764552   0.071869
 C     -1.564128   4.775432  -1.343547
 H     -1.132307   5.767482  -1.134339
 C     -2.594093   4.640028  -2.294810
 H     -2.971209   5.524102  -2.833303
 C     -3.135567   3.369313  -2.553566
 H     -3.941437   3.252385  -3.296162
 C     -2.657216   2.238798  -1.862225
 H     -3.099956   1.252064  -2.067533
 C     -1.550404   1.222800   1.718979
 C     -0.869931   0.722491   2.850050
 H      0.073689   0.177379   2.696653
 C     -1.384735   0.919749   4.143963
 H     -0.837786   0.524086   5.015018
 C     -2.588004   1.624994   4.327815
 H     -2.989526   1.783322   5.341559
 C     -3.271480   2.133525   3.208628
 H     -4.212260   2.691759   3.341195
 C     -2.755501   1.936998   1.914508
 H     -3.297244   2.350602   1.049573
 C     -0.253767  -1.341840  -2.439373
 H     -0.141062  -2.339482  -2.927878
 C      2.198101  -1.337946  -2.274806
 H      2.245092  -2.322409  -2.796547
 H      2.252441  -0.551981  -3.058978
 C      3.385113  -1.170032  -1.365880
 C      4.269918  -0.188609   0.572458
 C      5.561034  -0.662797   0.218677
 H      6.406493  -0.477341   0.894252
 C      5.744570  -1.421366  -0.938941
 C      4.624444  -1.714524  -1.731372
 H      4.702901  -2.332379  -2.637941
 C     -0.347409  -0.287994  -3.555963
```

H	-1.246490	-0.497387	-4.168810
H	-0.440657	0.733684	-3.139567
H	0.527646	-0.315000	-4.235569
C	1.652430	1.838335	0.878998
H	0.859317	-0.444489	0.928272
H	0.854477	-1.895749	-0.829077
C	1.599147	1.685926	-1.609733
O	1.747761	2.520730	-2.439701
H	6.741131	-1.810548	-1.199227
N	4.078471	0.497805	1.771498
C	3.145183	-0.076483	2.740880
H	3.639073	-0.869069	3.355055
H	2.774857	0.718214	3.419827
H	2.274129	-0.499994	2.203485
C	5.223727	1.152037	2.387791
H	4.849281	1.897836	3.118954
H	5.897237	0.450829	2.941423
H	5.822904	1.689562	1.627026

4/3-mRSS

Frequencies, energies and thermodynamic properties:

Lowest Vibrational Mode (1/cm) =	12.4391
E(RB-P86) (a.u.) =	-4386.45415387
Thermal correction to Enthalpy (a.u.) =	0.637413
Thermal correction to Gibbs Free Energy (a.u.) =	0.524523
Total Entropy (cal/Kmol) =	237.597
E(RPBE1PBE) (a.u.) =	-4385.76874190

Optimised cartesian coordinates (Angstrom):

Fe	-3.336159	-1.176966	-0.699834
Mn	1.575068	-0.162431	0.375095
P	-0.383059	0.845981	0.025253
O	2.571629	2.423514	1.368429
O	1.337634	-1.075590	3.196440
N	0.953500	-2.073752	-0.429160
N	3.430141	-1.098820	0.016443
C	-1.535240	-0.205097	-0.970540
C	-1.386358	-1.651086	-1.073983
C	-2.238633	-2.104101	-2.152927
H	-2.368758	-3.140828	-2.487135
C	-2.930332	-0.965435	-2.693747
H	-3.668912	-0.987030	-3.506316
C	-2.510544	0.200234	-1.963388
H	-2.851738	1.227371	-2.145289
C	-4.028683	-0.865157	1.218662
H	-3.497658	-0.329466	2.015525
C	-3.935690	-2.273516	0.937599
H	-3.330142	-3.002387	1.492868
C	-4.770102	-2.556823	-0.202999
H	-4.906432	-3.538331	-0.676504
C	-5.378640	-1.320468	-0.626379
H	-6.059367	-1.195895	-1.479102
C	-4.918454	-0.274219	0.251715
H	-5.192212	0.786806	0.191031
C	-0.208967	2.371602	-1.047329
C	-0.055341	3.644710	-0.455094
H	-0.124567	3.755488	0.637657
C	0.190080	4.782680	-1.246777
H	0.307631	5.766037	-0.763442
C	0.282961	4.667033	-2.644462
H	0.468752	5.558186	-3.265062
C	0.141811	3.401229	-3.244177
H	0.218099	3.296220	-4.338564
C	-0.092163	2.263894	-2.452027
H	-0.178890	1.278559	-2.935436
C	-1.470267	1.544772	1.381677
C	-1.178907	1.275528	2.734540
H	-0.308204	0.657441	2.993887
C	-2.002264	1.779537	3.761519
H	-1.759598	1.556308	4.812935
C	-3.123625	2.565611	3.447915
H	-3.766070	2.962553	4.250135
C	-3.419602	2.846511	2.099707
H	-4.294716	3.465201	1.843421
C	-2.599890	2.341803	1.076949
H	-2.843029	2.577498	0.029286
C	-0.473004	-2.510313	-0.220090
H	-0.680184	-2.299116	0.851147

C	1.937118	-3.021926	0.129767
H	1.672413	-3.184276	1.198822
H	1.914450	-4.016089	-0.367090
C	3.331668	-2.458433	0.048565
C	4.693186	-0.548954	-0.058499
C	5.847104	-1.373274	0.046307
H	6.843306	-0.914350	-0.000149
C	5.721274	-2.760167	0.134766
C	4.435312	-3.321316	0.098031
H	4.277434	-4.409370	0.129420
C	-0.709406	-4.008269	-0.477613
H	-1.787385	-4.234954	-0.363454
H	-0.404682	-4.303345	-1.503476
H	-0.164082	-4.644502	0.245322
C	2.206505	1.378413	0.941931
C	1.425938	-0.736290	2.060279
H	1.114588	-1.999744	-1.445801
H	1.558793	0.273966	-1.176582
H	6.616769	-3.398317	0.191766
N	4.828983	0.828027	-0.215083
C	4.210120	1.465388	-1.377379
H	3.230918	0.989951	-1.583765
H	4.027354	2.537490	-1.160742
H	4.871666	1.391170	-2.275067
C	6.075865	1.456452	0.197139
H	6.415376	1.052818	1.171049
H	6.903893	1.338255	-0.545595
H	5.895033	2.543923	0.321523

4/3s-fRSR

Frequencies, energies and thermodynamic properties:

Lowest Vibrational Mode (1/cm) =	15.8860
E(RB-P86) (a.u.) =	-4541.39672019
Thermal correction to Enthalpy (a.u.) =	0.721663
Thermal correction to Gibbs Free Energy (a.u.) =	0.594879
Total Entropy (cal/Kmol) =	266.839
E(RPBE1PBE) (a.u.) =	-4540.72099008

Optimised cartesian coordinates (Angstrom):

Fe	-3.490062	0.841109	0.668352
Mn	1.109034	0.583261	-0.897082
P	-0.421345	-0.887084	-0.061635
O	1.700419	-1.182659	-3.176822
O	-0.631945	2.004396	-2.777343
N	0.705773	1.877017	0.763257
N	2.696511	0.059720	0.431238
C	-1.685236	-0.082439	1.013950
C	-1.644228	1.318604	1.432200
C	-2.672985	1.498268	2.431878
H	-2.918558	2.438825	2.940725
C	-3.356046	0.247982	2.624329
H	-4.205083	0.075750	3.299431
C	-2.759076	-0.723603	1.750590
H	-3.059980	-1.775129	1.658332
C	-3.731353	1.361903	-1.310913
H	-2.921589	1.453998	-2.046328
C	-4.207435	2.413498	-0.451422
H	-3.832454	3.445570	-0.425158
C	-5.246005	1.870046	0.386321
H	-5.796961	2.412870	1.166093
C	-5.414258	0.481356	0.041270
H	-6.116439	-0.219386	0.512633
C	-4.476747	0.165938	-1.004930
H	-4.336530	-0.816014	-1.474741
C	0.268235	-2.226793	1.042375
C	1.116461	-3.187335	0.438372
H	1.288835	-3.155496	-0.650041
C	1.733827	-4.186384	1.208418
H	2.378605	-4.933222	0.717834
C	1.532721	-4.232683	2.601881
H	2.019368	-5.014227	3.206782
C	0.707493	-3.274283	3.214091
H	0.543086	-3.300581	4.303394
C	0.078028	-2.279133	2.440687
H	-0.578771	-1.547953	2.936992
C	-1.457448	-1.919414	-1.224350
C	-1.673020	-1.490865	-2.552244
H	-1.206927	-0.562612	-2.910933

```
 C     -2.486199  -2.238046  -3.424055
 H     -2.641498  -1.887711  -4.457043
 C     -3.094531  -3.426789  -2.983100
 H     -3.729117  -4.012607  -3.667095
 C     -2.880661  -3.866974  -1.663947
 H     -3.346870  -4.800220  -1.309160
 C     -2.065478  -3.121945  -0.793533
 H     -1.894252  -3.489999   0.230150
 C     -0.693787   2.399648   0.933628
 H     -1.001568   2.691099  -0.090059
 C      1.271515   1.300151   1.995043
 H      1.500666   2.070890   2.764348
 H      0.507398   0.630663   2.448258
 C      2.505768   0.481336   1.714618
 C      3.811871  -0.707666   0.172160
 C      4.652839  -1.153021   1.223330
 C      4.415672  -0.744956   2.537552
 C      3.339289   0.117970   2.783961
 C     -0.725257   3.668448   1.802899
 H     -1.729138   4.136773   1.768341
 H     -0.484475   3.462652   2.865975
 H      0.001752   4.408341   1.410796
 C      1.520054  -0.492186  -2.229929
 C      0.013494   1.398927  -1.982145
 H      2.124783   1.710367  -1.423005
 H      2.163236   3.138751  -1.095869
 H      1.281303   2.702333   0.492809
 O      2.178384   4.007896  -0.585876
 C      3.547445   4.330529  -0.322097
 H      3.542947   5.091504   0.488531
 H      4.095677   3.442725   0.075147
 H      3.121271   0.493770   3.794481
 H      5.527124  -1.774077   0.987093
 C      4.266778   4.882770  -1.552436
 H      3.738703   5.775311  -1.948048
 H      5.308060   5.177497  -1.302415
 H      4.313851   4.124331  -2.362521
 N      4.105512  -1.043521  -1.154084
 C      4.862997  -2.263542  -1.398738
 H      4.723914  -2.555671  -2.460297
 H      5.962918  -2.151942  -1.225841
 H      4.486481  -3.088561  -0.763220
 C      4.485386   0.052156  -2.049834
 H      5.564200   0.317845  -1.925713
 H      4.313203  -0.250786  -3.102491
 H      3.855752   0.938310  -1.843878
 H      5.082851  -1.066478   3.352330
```

```
4/5-fRSR-R-8al
Frequencies, energies and thermodynamic properties:
 Lowest Vibrational Mode (1/cm) =                          9.2591
 E(RB-P86) (a.u.) =                                     -4809.17061051
 Thermal correction to Enthalpy (a.u.) =                   0.791265
 Thermal correction to Gibbs Free Energy (a.u.) =          0.656055
 Total Entropy (cal/Kmol) =                                284.574
 E(RPBE1PBE) (a.u.) =                                   -4808.48439393
Optimised cartesian coordinates (Angstrom):
 Fe    -2.216030  -2.982401  -0.845969
 Mn     0.499359   0.754949   0.410567
 P     -1.649650   0.354420   0.329772
 O      0.252829   1.915262   3.128240
 O      1.219543  -1.692169   1.847758
 N      0.780833   0.053619  -1.386283
 N      0.794148   2.581316  -0.664573
 C     -2.057734  -0.936238  -0.907736
 C     -1.083193  -1.547927  -1.804522
 C     -1.822559  -2.340735  -2.758295
 H     -1.387761  -2.948311  -3.562081
 C     -3.226911  -2.236749  -2.461541
 H     -4.039169  -2.745604  -2.998094
 C     -3.378314  -1.380871  -1.316541
 H     -4.328165  -1.103571  -0.841221
 C     -1.314393  -3.787307   0.824114
 H     -0.569141  -3.282010   1.451794
 C     -1.028647  -4.587261  -0.337035
 H     -0.031032  -4.803576  -0.742383
 C     -2.281433  -5.034402  -0.890909
```

H	-2.406562	-5.646707	-1.794046
C	-3.342922	-4.512493	-0.067908
H	-4.418713	-4.658300	-0.234413
C	-2.746321	-3.739225	0.990267
H	-3.284436	-3.193442	1.776105
C	-2.565166	1.853735	-0.287275
C	-2.628628	2.983697	0.564075
H	-2.182422	2.937854	1.570779
C	-3.261942	4.162414	0.139262
H	-3.314134	5.028901	0.817619
C	-3.826398	4.238085	-1.149264
H	-4.321285	5.163971	-1.482828
C	-3.753834	3.127322	-2.006515
H	-4.191659	3.178068	-3.016251
C	-3.127899	1.939908	-1.579788
H	-3.092846	1.072610	-2.256261
C	-2.651769	-0.097075	1.830542
C	-2.041564	-0.716475	2.943054
H	-0.959805	-0.909659	2.944492
C	-2.809607	-1.096615	4.058549
H	-2.317147	-1.576712	4.919042
C	-4.196062	-0.862178	4.078636
H	-4.795611	-1.158116	4.954091
C	-4.811894	-0.239928	2.977420
H	-5.896224	-0.045332	2.985935
C	-4.045488	0.144283	1.863190
H	-4.539300	0.646978	1.016975
C	0.435696	-1.345167	-1.737205
H	0.816595	-1.958400	-0.895642
C	0.483194	0.984136	-2.465296
H	1.168448	0.869082	-3.341880
H	-0.542148	0.809339	-2.883358
C	0.559109	2.407300	-1.995381
C	0.965869	3.866157	-0.191318
C	0.714199	4.980086	-1.031251
C	0.399892	4.780480	-2.379652
C	0.362506	3.475160	-2.886175
H	0.163337	3.271103	-3.948300
C	1.134130	-1.858210	-3.012947
H	0.992956	-2.953558	-3.112344
H	0.737841	-1.385635	-3.935215
H	2.221466	-1.656345	-2.946073
C	0.385049	1.515945	2.022105
C	0.864265	-0.730499	1.246677
H	2.557931	-0.010400	-1.411098
H	3.480243	0.158757	0.477957
C	4.220478	-0.076372	-0.335566
C	4.938418	-1.368598	0.051168
C	5.375635	0.962642	-0.294813
C	6.172609	-1.081615	0.675326
C	4.517566	-2.694502	-0.132585
C	6.387216	0.421031	0.744282
H	5.834825	0.962749	-1.307796
C	6.996134	-2.126251	1.128851
C	5.344517	-3.742638	0.319187
H	3.558402	-2.911002	-0.630255
H	7.436712	0.721990	0.542307
C	6.574916	-3.459485	0.946686
H	7.964167	-1.911156	1.611437
H	5.031706	-4.789956	0.177518
H	7.215904	-4.287386	1.290891
O	3.551692	-0.112614	-1.577603
H	5.024573	1.992813	-0.084576
H	6.143451	0.795137	1.765589
H	0.229211	5.645611	-3.039374
H	0.833128	5.998249	-0.639104
N	1.396992	4.040302	1.118498
C	1.172923	5.326281	1.765572
C	2.641434	3.391779	1.540608
H	0.149157	5.692845	1.558364
H	1.272537	5.189999	2.861571
H	1.905033	6.111846	1.455429
H	2.609489	3.179131	2.627665
H	2.772850	2.432599	1.010164
H	3.520393	4.047734	1.330345

4/5-fRSR-S-8al

```
Frequencies, energies and thermodynamic properties:
 Lowest Vibrational Mode (1/cm) =                          10.8159
 E(RB-P86) (a.u.) =                                      -4809.17182546
 Thermal correction to Enthalpy (a.u.) =                   0.791239
 Thermal correction to Gibbs Free Energy (a.u.) =          0.655489
 Total Entropy (cal/Kmol) =                                285.709
 E(RPBE1PBE) (a.u.) =                                    -4808.48444891
Optimised cartesian coordinates (Angstrom):
 Fe    -3.203352  -2.312645  -0.802621
 Mn     0.558595   0.382040   0.429732
 P     -1.603165   0.698298   0.314105
 O      0.690082   1.651016   3.106955
 O      0.416948  -2.125504   1.933739
 N      0.612401  -0.414686  -1.347937
 N      1.462691   1.972409  -0.676217
 C     -2.395562  -0.426239  -0.899422
 C     -1.662376  -1.339349  -1.769619
 C     -2.609040  -1.873283  -2.720012
 H     -2.385841  -2.606580  -3.505429
 C     -3.907928  -1.317173  -2.446109
 H     -4.836314  -1.550517  -2.984849
 C     -3.785704  -0.432955  -1.319219
 H     -4.599935   0.144668  -0.862723
 C     -2.616514  -3.333695   0.889720
 H     -1.751319  -3.082535   1.516605
 C     -2.597508  -4.203949  -0.255730
 H     -1.720160  -4.735502  -0.648206
 C     -3.924968  -4.235068  -0.815337
 H     -4.235787  -4.790903  -1.710137
 C     -4.766334  -3.384974  -0.011702
 H     -5.831113  -3.180480  -0.187032
 C     -3.957836  -2.825096   1.040138
 H     -4.296351  -2.122003   1.812216
 C     -1.971300   2.395050  -0.359958
 C     -1.663096   3.509241   0.458239
 H     -1.259306   3.351024   1.471464
 C     -1.874899   4.816694  -0.007273
 H     -1.641799   5.672971   0.645516
 C     -2.380637   5.033221  -1.304168
 H     -2.544292   6.059356  -1.669848
 C     -2.674384   3.934090  -2.128500
 H     -3.069598   4.094275  -3.144367
 C     -2.472527   2.620905  -1.660892
 H     -2.722319   1.769584  -2.311936
 C     -2.716224   0.645025   1.803202
 C     -2.354365  -0.103121   2.944648
 H     -1.394273  -0.636884   2.974291
 C     -3.217711  -0.176956   4.052767
 H     -2.918867  -0.762981   4.936353
 C     -4.452291   0.496244   4.036370
 H     -5.125951   0.439768   4.906110
 C     -4.818762   1.249039   2.905829
 H     -5.780686   1.785694   2.885473
 C     -3.955793   1.327044   1.798754
 H     -4.249083   1.934762   0.928540
 C     -0.160049  -1.635058  -1.678854
 H     -0.007214  -2.319536  -0.820134
 C      0.637660   0.538642  -2.448318
 H      1.258129   0.191430  -3.312150
 H     -0.385522   0.691695  -2.879524
 C      1.164744   1.871278  -2.001554
 C      2.061042   3.129494  -0.222195
 C      2.162886   4.262967  -1.068846
 C      1.771877   4.166530  -2.408239
 C      1.308380   2.939187  -2.901639
 H      1.038243   2.804790  -3.959153
 C      0.355104  -2.372855  -2.930995
 H     -0.119373  -3.371815  -3.011753
 H      0.138151  -1.822535  -3.869584
 H      1.450628  -2.517048  -2.847089
 C      0.688449   1.188551   2.017724
 C      0.404834  -1.115590   1.305850
 H      2.292578  -1.040141  -1.299450
 H      3.197753  -1.182136   0.649622
 C      3.700438  -1.804801  -0.141210
 C      3.511956  -3.305331   0.229710
 C      4.655471  -3.621838   1.226138
```

O	3.221337	-1.427042	-1.413887
H	1.875161	5.037092	-3.074839
H	2.613688	5.189973	-0.692033
N	2.569524	3.146605	1.067836
C	2.835349	4.427113	1.708587
C	3.490282	2.083548	1.478885
H	1.999802	5.131682	1.532798
H	2.918963	4.262797	2.801938
H	3.785540	4.903863	1.364194
H	3.362096	1.856486	2.556162
H	3.286969	1.163227	0.904661
H	4.548359	2.391685	1.302122
C	5.205003	-1.623448	0.009312
C	5.750553	-2.657207	0.802862
C	6.022295	-0.612269	-0.518417
C	7.126430	-2.677314	1.087238
C	7.403479	-0.635818	-0.237209
H	5.587844	0.176625	-1.153510
C	7.950349	-1.659923	0.562578
H	7.562632	-3.482755	1.701361
H	8.062010	0.145865	-0.649681
H	9.032537	-1.671207	0.771144
H	3.652070	-3.890619	-0.705547
H	2.501420	-3.523829	0.628514
H	4.978871	-4.683917	1.212099
H	4.337514	-3.403483	2.272040

4/TSHy-fRSR-re-10ke
Frequencies, energies and thermodynamic properties:

Lowest Vibrational Mode (1/cm) =	-420.2956
E(RB-P86) (a.u.) =	-4734.18906272
Thermal correction to Enthalpy (a.u.) =	0.794876
Thermal correction to Gibbs Free Energy (a.u.) =	0.664385
Total Entropy (cal/Kmol) =	274.642
E(RPBE1PBE) (a.u.) =	-4733.50659475

Optimised cartesian coordinates (Angstrom):

Fe	-3.735982	-1.197647	-0.709037
Mn	0.941322	-0.370509	0.352669
P	-0.959577	0.857372	0.289208
O	1.608139	0.574763	3.061791
O	-0.196094	-2.641996	1.812362
N	0.509188	-1.155149	-1.536215
N	2.072557	0.966323	-0.871094
C	-2.207074	0.166243	-0.877997
C	-1.977601	-0.982446	-1.751890
C	-3.101516	-1.064303	-2.656622
H	-3.248856	-1.826807	-3.431778
C	-4.022593	-0.004003	-2.348732
H	-4.984546	0.178161	-2.846502
C	-3.482636	0.749798	-1.251426
H	-3.950564	1.624036	-0.781097
C	-3.562479	-2.368577	0.978598
H	-2.642900	-2.515164	1.560041
C	-3.980377	-3.163651	-0.145754
H	-3.441103	-4.024186	-0.564140
C	-5.217969	-2.618660	-0.642979
H	-5.783841	-2.986696	-1.509322
C	-5.567106	-1.487312	0.177996
H	-6.446252	-0.842292	0.046497
C	-4.543412	-1.330478	1.178143
H	-4.503296	-0.547051	1.945933
C	-0.750379	2.605578	-0.327419
C	-0.119770	3.538162	0.531413
H	0.179614	3.231253	1.546649
C	0.119262	4.855627	0.107346
H	0.592882	5.573475	0.796229
C	-0.245828	5.259688	-1.191605
H	-0.056819	6.292588	-1.524532
C	-0.853757	4.336150	-2.058670
H	-1.143481	4.640989	-3.077099
C	-1.106813	3.018578	-1.629889
H	-1.603907	2.315599	-2.316003
C	-1.949303	1.168575	1.842208
C	-1.821973	0.310008	2.955566
H	-1.114167	-0.529131	2.925181
C	-2.599476	0.512785	4.110738
H	-2.483729	-0.167378	4.969642

```
 C     -3.515476   1.577620   4.171718
 H     -4.122102   1.737107   5.077358
 C     -3.646632   2.443016   3.070000
 H     -4.356803   3.284558   3.108186
 C     -2.867539   2.242881   1.917072
 H     -2.970924   2.941038   1.071971
 C     -0.772116  -1.912640  -1.732124
 H     -0.844967  -2.560630  -0.836686
 C      0.770874  -0.181969  -2.607185
 H      1.096182  -0.671933  -3.552135
 H     -0.176293   0.351752  -2.848576
 C      1.789164   0.850573  -2.200649
 C      2.942860   1.966045  -0.490287
 C      3.456090   2.888209  -1.434669
 H      4.146209   3.673434  -1.098623
 C      3.144259   2.759925  -2.790495
 C      2.313512   1.704916  -3.184985
 H      2.039864   1.549277  -4.238988
 C     -0.712768  -2.838842  -2.959279
 H     -1.591873  -3.513730  -2.975401
 H     -0.699840  -2.278287  -3.916461
 H      0.197364  -3.468675  -2.902159
 C      1.398378   0.250591   1.941372
 C      0.213188  -1.704343   1.204695
 H      1.286926  -1.895361  -1.591611
 H      2.284426  -1.435131   0.240943
 O      2.483325  -2.875411  -1.558663
 H      3.564978   3.458644  -3.530234
 N      3.315387   2.062732   0.857990
 C      3.692124   3.378046   1.361352
 H      2.983372   4.148535   1.001459
 H      3.645254   3.354848   2.469880
 H      4.730740   3.685583   1.079963
 C      4.185200   0.999918   1.370301
 H      3.870551   0.023210   0.962335
 H      5.250947   1.185350   1.087302
 H      4.112506   0.956191   2.474957
 C      3.086651  -2.647893  -0.445841
 C      4.439963  -2.014004  -0.514870
 C      2.923260  -3.652722   0.706282
 C      5.434674  -2.226790   0.390865
 H      4.635082  -1.427515  -1.430178
 C      3.698580  -3.237870   1.960504
 H      3.321355  -4.618318   0.313152
 C      5.190948  -3.036430   1.649353
 H      3.573148  -3.991044   2.766924
 H      1.847813  -3.814636   0.912925
 H      3.272886  -2.285597   2.346728
 H      5.702716  -4.023007   1.534290
 H      5.709183  -2.546873   2.504685
 C      6.832079  -1.703171   0.183817
 H      6.939548  -1.162347  -0.777368
 H      7.124798  -1.015709   1.008767
 H      7.572342  -2.534322   0.202480

4/TSHy-fRSR-re-11ke
Frequencies, energies and thermodynamic properties:
 Lowest Vibrational Mode (1/cm) =                           -407.3415
 E(RB-P86) (a.u.) =                                         -4773.46498472
 Thermal correction to Enthalpy (a.u.) =                     0.823145
 Thermal correction to Gibbs Free Energy (a.u.) =            0.690253
 Total Entropy (cal/Kmol) =                                  279.693
 E(RPBE1PBE) (a.u.) =                                       -4772.78233175
Optimised cartesian coordinates (Angstrom):
 Fe    -3.620853  -1.638082  -0.663035
 Mn     0.912496  -0.099922   0.162389
 P     -1.166933   0.768451   0.382917
 O      1.633429   0.650595   2.918650
 O      0.302802  -2.681458   1.404966
 N      0.449716  -0.710403  -1.784252
 N      1.699795   1.550334  -0.947211
 C     -2.378777  -0.003243  -0.771153
 C     -2.036593  -0.984615  -1.798822
 C     -3.210705  -1.173281  -2.619809
 H     -3.295443  -1.858451  -3.472620
 C     -4.269672  -0.343891  -2.111894
 H     -5.292161  -0.289672  -2.509199
```

C	-3.767239	0.370508	-0.971246
H	-4.334354	1.084292	-0.360179
C	-3.092404	-2.917548	0.863718
H	-2.117351	-2.937840	1.367172
C	-3.441368	-3.660737	-0.318093
H	-2.784051	-4.350509	-0.864513
C	-4.795993	-3.323342	-0.674133
H	-5.350071	-3.705695	-1.541911
C	-5.285720	-2.372648	0.291496
H	-6.279117	-1.904235	0.288083
C	-4.232813	-2.119443	1.240415
H	-4.279917	-1.426410	2.090326
C	-1.310958	2.584087	-0.020833
C	-0.741514	3.505386	0.891380
H	-0.280418	3.138507	1.822792
C	-0.767352	4.884447	0.627147
H	-0.336982	5.588481	1.357395
C	-1.341329	5.367270	-0.565129
H	-1.359590	6.448867	-0.773118
C	-1.890757	4.459670	-1.486327
H	-2.342093	4.826018	-2.422348
C	-1.878454	3.077295	-1.215955
H	-2.333550	2.383480	-1.939502
C	-2.052919	0.725080	2.026183
C	-1.688195	-0.216073	3.013181
H	-0.855702	-0.909193	2.832468
C	-2.385172	-0.282196	4.233739
H	-2.083758	-1.022596	4.991820
C	-3.456647	0.592010	4.487498
H	-4.000405	0.540860	5.444225
C	-3.824695	1.538303	3.513418
H	-4.658570	2.233005	3.703237
C	-3.126108	1.607375	2.295427
H	-3.416492	2.366300	1.552466
C	-0.688676	-1.666912	-1.993408
H	-0.560938	-2.424096	-1.194701
C	0.424276	0.426838	-2.716747
H	0.710426	0.131632	-3.750891
H	-0.617533	0.813618	-2.784814
C	1.305633	1.556208	-2.253464
C	2.435930	2.627699	-0.500146
C	2.698703	3.734970	-1.343834
H	3.287079	4.577558	-0.957230
C	2.275078	3.726549	-2.674856
C	1.585918	2.603353	-3.146723
H	1.234848	2.536542	-4.186973
C	-0.580011	-2.404196	-3.339451
H	-1.317280	-3.230345	-3.387576
H	-0.762617	-1.737469	-4.207007
H	0.432206	-2.845628	-3.436340
C	1.390453	0.413834	1.783517
C	0.493713	-1.626888	0.888278
H	1.339273	-1.264488	-2.010375
H	2.389636	-0.913126	-0.162525
O	2.747376	-1.901693	-2.250760
H	2.503252	4.573822	-3.340059
N	2.923684	2.617552	0.813362
C	3.154612	3.903711	1.459563
H	2.309222	4.592136	1.267327
H	3.223629	3.737387	2.554573
H	4.103614	4.402771	1.139281
C	3.978253	1.644509	1.110419
H	3.757421	0.684032	0.613240
H	4.974756	2.014070	0.763021
H	4.025777	1.465836	2.202930
C	3.324051	-1.877449	-1.105811
C	4.597304	-1.099775	-0.981943
C	3.277579	-3.128781	-0.216462
C	5.583876	-1.418325	-0.108289
H	4.734841	-0.294689	-1.724238
C	4.010382	-2.942530	1.115893
H	3.759259	-3.931344	-0.822118
C	5.492952	-2.511438	0.944959
H	6.542312	-0.867084	-0.157216
H	3.961827	-3.873252	1.722108
H	2.227891	-3.445200	-0.063114
H	3.482121	-2.157805	1.701047

```
 C      6.033108  -1.967738   2.288796
 H      5.964387  -2.746611   3.078006
 H      7.098435  -1.665024   2.202724
 H      5.451968  -1.085622   2.629860
 C      6.377687  -3.706723   0.501015
 H      7.425482  -3.383925   0.324343
 H      6.392116  -4.490869   1.288254
 H      6.006542  -4.166541  -0.437911
```

4/TSHy-fRSR-re-12ke

```
Frequencies, energies and thermodynamic properties:
 Lowest Vibrational Mode (1/cm) =                          -319.8590
 E(RB-P86) (a.u.) =                                        -5119.80411550
 Thermal correction to Enthalpy (a.u.) =                    0.947890
 Thermal correction to Gibbs Free Energy (a.u.) =           0.796155
 Total Entropy (cal/Kmol) =                                 319.353
 E(RPBE1PBE) (a.u.) =                                      -5119.09864707
Optimised cartesian coordinates (Angstrom):
 Fe     4.475578   0.925060  -1.285198
 Mn    -0.021629   0.168355   0.421670
 P      1.928305  -0.982037   0.388874
 O     -0.358362  -0.263081   3.315248
 O      1.155544   2.716166   1.267344
 N      0.172862   0.594135  -1.621822
 N     -1.193634  -1.412979  -0.394888
 C      3.011642  -0.492293  -1.018660
 C      2.634110   0.454299  -2.066422
 C      3.654519   0.391426  -3.088435
 H      3.682434   0.990471  -4.007178
 C      4.656074  -0.558243  -2.686216
 H      5.570466  -0.804191  -3.242696
 C      4.270979  -1.097834  -1.411901
 H      4.829398  -1.847208  -0.836547
 C      4.420462   2.388367   0.165509
 H      3.562429   2.607195   0.814211
 C      4.664088   2.970051  -1.128036
 H      4.031984   3.714560  -1.630560
 C      5.869388   2.387194  -1.660078
 H      6.313691   2.604697  -2.640638
 C      6.373005   1.446375  -0.692026
 H      7.268948   0.821469  -0.806192
 C      5.476804   1.444435   0.434802
 H      5.568211   0.820681   1.333429
 C      1.751170  -2.823735   0.149738
 C      1.247807  -3.581798   1.234743
 H      1.024789  -3.084250   2.192514
 C      1.041098  -4.965164   1.108069
 H      0.667456  -5.542339   1.969121
 C      1.311556  -5.613388  -0.112817
 H      1.148436  -6.698243  -0.212486
 C      1.790915  -4.866368  -1.202093
 H      2.004952  -5.362821  -2.162193
 C      2.011391  -3.481206  -1.072436
 H      2.407570  -2.916815  -1.930612
 C      3.086521  -0.949525   1.853692
 C      3.032273   0.108957   2.786349
 H      2.284670   0.905535   2.672821
 C      3.933809   0.161183   3.865494
 H      3.873686   0.994580   4.583519
 C      4.902899  -0.844027   4.030080
 H      5.606783  -0.803468   4.876548
 C      4.962486  -1.906630   3.109708
 H      5.713574  -2.703502   3.231535
 C      4.059570  -1.961229   2.033580
 H      4.109304  -2.809929   1.333972
 C      1.391644   1.335108  -2.099666
 H      1.518801   2.153402  -1.364211
 C     -0.136907  -0.583057  -2.449578
 H     -0.582540  -0.306633  -3.431163
 H      0.816220  -1.107944  -2.684626
 C     -1.038504  -1.560652  -1.742108
 C     -1.972543  -2.342896   0.261982
 C     -2.500611  -3.466613  -0.420916
 H     -3.110443  -4.193988   0.130820
 C     -2.308409  -3.612485  -1.796663
 C     -1.587195  -2.624171  -2.477208
 H     -1.416465  -2.678237  -3.562495
```

```
 C      1.166138   1.986558  -3.475054
 H      2.004661   2.669124  -3.718736
 H      1.090826   1.240137  -4.292082
 H      0.236266   2.588987  -3.456205
 C     -0.275853  -0.150879   2.138611
 C      0.722388   1.670019   0.909836
 H     -0.626590   1.288398  -1.722742
 H     -1.413042   1.133892   0.315456
 C     -2.375508   2.276279  -0.539177
 C     -3.701544   1.535183  -0.435077
 C     -4.702122   1.820671   0.522852
 C     -4.008662   0.581425  -1.430201
 C     -5.943243   1.165794   0.486765
 C     -5.245774  -0.079564  -1.454996
 H     -3.257511   0.376639  -2.207469
 C     -6.249773   0.194963  -0.496353
 H     -6.694862   1.432332   1.247275
 H     -5.428035  -0.816741  -2.250368
 O     -1.846728   2.351070  -1.696693
 H     -2.741228  -4.470632  -2.333922
 H     -4.526334   2.581681   1.296989
 C     -2.122009   3.417355   0.437539
 C     -2.181640   3.283918   1.843201
 C     -1.837091   4.687378  -0.110438
 C     -1.986320   4.397340   2.676663
 H     -2.346009   2.290350   2.288001
 C     -1.641049   5.803250   0.722002
 H     -1.775194   4.782254  -1.205520
 C     -1.720408   5.663199   2.119152
 H     -2.028544   4.274854   3.770963
 H     -1.425060   6.787909   0.276555
 H     -1.565962   6.535404   2.774452
 C     -7.629569  -0.497773  -0.501224
 C     -7.844278  -1.224804   0.852298
 C     -8.736469   0.573178  -0.690687
 C     -7.756626  -1.536137  -1.636470
 H     -7.067280  -2.000875   1.015603
 H     -7.808755  -0.522627   1.710272
 H     -8.836142  -1.724726   0.868492
 H     -8.613153   1.107639  -1.655939
 H     -9.739525   0.095741  -0.689172
 H     -8.722994   1.329499   0.120714
 H     -8.758364  -2.012301  -1.598220
 H     -7.647668  -1.071898  -2.638668
 H     -7.000180  -2.343348  -1.546497
 N     -2.237286  -2.162240   1.624717
 C     -2.528310  -3.346061   2.423593
 C     -3.086196  -1.021147   1.979525
 H     -1.822741  -4.163203   2.178248
 H     -2.397428  -3.085999   3.494355
 H     -3.573795  -3.724983   2.299158
 H     -2.933886  -0.758804   3.045149
 H     -2.812697  -0.147160   1.362699
 H     -4.165970  -1.257433   1.816192

4/TSHy-fRSR-re-13ke
Frequencies, energies and thermodynamic properties:
 Lowest Vibrational Mode (1/cm) =                         -120.9220
 E(RB-P86) (a.u.) =                                       -5375.62327407
 Thermal correction to Enthalpy (a.u.) =                   0.855677
 Thermal correction to Gibbs Free Energy (a.u.) =          0.706873
 Total Entropy (cal/Kmol) =                                313.184
 E(RPBE1PBE) (a.u.) =                                     -5374.99791443
Optimised cartesian coordinates (Angstrom):
 Fe    -2.556139  -3.331051  -0.762180
 Mn    -0.115734   0.758159   0.216752
 P     -2.275269   0.075639   0.333815
 O     -0.241758   1.816149   2.963708
 O      1.073307  -1.617106   1.450459
 N      0.037888  -0.009573  -1.733041
 N     -0.529837   2.524489  -0.923877
 C     -2.648050  -1.280690  -0.854676
 C     -1.704500  -1.804864  -1.840574
 C     -2.443173  -2.693521  -2.709435
 H     -2.027520  -3.266144  -3.547813
 C     -3.811187  -2.740298  -2.270558
 H     -4.609195  -3.350229  -2.714782
```

C	-3.941270	-1.881066	-1.127021
H	-4.863670	-1.701799	-0.559984
C	-1.402490	-3.985206	0.814467
H	-0.666196	-3.378179	1.356782
C	-1.138437	-4.770327	-0.362387
H	-0.167465	-4.873339	-0.865344
C	-2.375793	-5.378164	-0.780472
H	-2.514854	-6.020356	-1.660506
C	-3.405079	-4.970734	0.141635
H	-4.466116	-5.249063	0.087173
C	-2.805128	-4.108049	1.125788
H	-3.325236	-3.613134	1.956062
C	-3.539227	1.372806	-0.109247
C	-3.756611	2.423667	0.814860
H	-3.228186	2.419871	1.781987
C	-4.649357	3.466319	0.517791
H	-4.820194	4.265590	1.256787
C	-5.324924	3.490776	-0.717909
H	-6.023958	4.309469	-0.951395
C	-5.101010	2.463287	-1.649783
H	-5.623897	2.472301	-2.619612
C	-4.215979	1.409962	-1.348057
H	-4.069971	0.602831	-2.082294
C	-2.985917	-0.551301	1.942725
C	-2.136860	-1.044350	2.957130
H	-1.047266	-1.031000	2.818402
C	-2.671713	-1.562069	4.151182
H	-1.992260	-1.940742	4.931324
C	-4.063098	-1.593390	4.350683
H	-4.480805	-1.997034	5.286734
C	-4.918417	-1.098485	3.349019
H	-6.010207	-1.111898	3.496543
C	-4.384365	-0.577796	2.157377
H	-5.067895	-0.178257	1.392239
C	-0.221430	-1.476534	-1.956832
H	0.317918	-1.977612	-1.129527
C	-0.651519	0.847438	-2.712759
H	-0.164380	0.822148	-3.712652
H	-1.679720	0.449032	-2.862261
C	-0.769010	2.272991	-2.242647
C	-0.697889	3.821553	-0.486032
C	-1.204203	4.826004	-1.347726
H	-1.334184	5.847122	-0.965984
C	-1.465173	4.540137	-2.689511
C	-1.214290	3.243714	-3.154302
H	-1.386257	2.963465	-4.204025
C	0.391316	-1.979232	-3.274746
H	0.330746	-3.084641	-3.325997
H	-0.124770	-1.571254	-4.167688
H	1.462205	-1.700325	-3.320936
C	-0.195915	1.461467	1.834537
C	0.539226	-0.683471	0.948284
H	1.072408	0.112476	-1.876499
H	1.531009	1.045389	0.084557
C	3.253654	0.639044	-0.906958
C	3.585013	2.157435	-1.067740
O	2.748532	0.125522	-1.933613
H	-1.830618	5.326227	-3.368360
N	-0.355255	4.139495	0.833788
C	-1.020004	5.277987	1.455193
H	-0.883754	5.205134	2.553938
H	-0.609065	6.267811	1.133791
H	-2.105894	5.257461	1.240259
C	1.067964	4.062687	1.169577
H	1.621832	4.960046	0.799838
H	1.189768	3.995520	2.268716
H	1.502171	3.155627	0.717103
F	3.859024	2.832694	0.072851
F	2.608263	2.819821	-1.721544
F	4.708128	2.235767	-1.842388
C	4.143902	-0.161263	0.019140
C	5.025865	-1.116702	-0.612224
C	4.209425	0.037562	1.440000
C	5.104003	-1.339537	-2.033637
C	5.954697	-1.882530	0.212373
C	5.163103	-0.729076	2.233032
C	3.350593	0.933593	2.162001

```
 C      5.983963  -2.258370  -2.582766
 H      4.451568  -0.763486  -2.699847
 C      6.840999  -2.838986  -0.392915
 C      5.996954  -1.668573   1.601953
 C      5.242776  -0.531543   3.654310
 H      2.569946   1.468137   1.610621
 C      3.450764   1.097363   3.534183
 C      6.854994  -3.034722  -1.759901
 H      6.012208  -2.388103  -3.676523
 H      7.518905  -3.405505   0.265790
 H      6.708164  -2.250006   2.211780
 C      4.413990   0.368832   4.296025
 H      5.980443  -1.123036   4.220275
 H      2.760629   1.787523   4.044290
 H      7.538817  -3.767857  -2.215550
 H      4.480725   0.513989   5.385702
```

```
4/TSHy-fRSR-re-15ke
Frequencies, energies and thermodynamic properties:
 Lowest Vibrational Mode (1/cm) =                           -348.6027
 E(RB-P86) (a.u.) =                                         -4810.35632986
 Thermal correction to Enthalpy (a.u.) =                     0.808335
 Thermal correction to Gibbs Free Energy (a.u.) =            0.673600
 Total Entropy (cal/Kmol) =                                  283.573
 E(RPBE1PBE) (a.u.) =                                       -4809.66547491
Optimised cartesian coordinates (Angstrom):
 Fe    -3.808618  -1.370719  -0.740112
 Mn     0.746909  -0.236140   0.530449
 P     -1.193782   0.893889   0.225203
 O      1.161302   1.029721   3.157982
 O     -0.421271  -2.381454   2.147795
 N      0.475107  -1.205466  -1.309872
 N      1.912879   1.030261  -0.742621
 C     -2.329652   0.040533  -0.948644
 C     -1.997494  -1.174265  -1.690460
 C     -3.056215  -1.394411  -2.649343
 H     -3.121441  -2.233165  -3.353682
 C     -4.037733  -0.354137  -2.503202
 H     -4.972107  -0.265652  -3.073584
 C     -3.601209   0.525415  -1.454571
 H     -4.134848   1.417084  -1.101553
 C     -3.704490  -2.338123   1.075782
 H     -2.825472  -2.361795   1.732610
 C     -3.989363  -3.275654   0.021745
 H     -3.373084  -4.141141  -0.256763
 C     -5.212062  -2.866927  -0.621256
 H     -5.687721  -3.362022  -1.478504
 C     -5.685217  -1.676965   0.039069
 H     -6.585230  -1.106663  -0.227361
 C     -4.752887  -1.348059   1.085877
 H     -4.815693  -0.484770   1.760966
 C     -1.009642   2.588527  -0.532829
 C     -0.454272   3.610795   0.274586
 H     -0.195664   3.398720   1.324736
 C     -0.240935   4.897986  -0.245069
 H      0.174149   5.686454   0.403146
 C     -0.557284   5.181812  -1.587936
 H     -0.388676   6.191145  -1.995692
 C     -1.089955   4.168756  -2.402861
 H     -1.340742   4.379431  -3.454896
 C     -1.316779   2.880791  -1.879548
 H     -1.755041   2.106263  -2.527729
 C     -2.302866   1.302622   1.671872
 C     -2.223517   0.562931   2.871582
 H     -1.481723  -0.239900   2.976076
 C     -3.092526   0.838876   3.943400
 H     -3.013147   0.251178   4.871853
 C     -4.053680   1.858948   3.833565
 H     -4.732417   2.075259   4.673872
 C     -4.137378   2.607046   2.644788
 H     -4.882025   3.413442   2.548928
 C     -3.266583   2.334489   1.575438
 H     -3.334097   2.942611   0.660121
 C     -0.757622  -2.041064  -1.513129
 H     -0.861238  -2.611823  -0.569138
 C      0.747910  -0.303822  -2.440324
 H      1.126951  -0.847969  -3.333751
```

```
 H     -0.207716   0.173230  -2.754174
 C      1.710013   0.793516  -2.070647
 C      2.727011   2.091072  -0.403004
 C      3.256987   2.952952  -1.395333
 H      3.899402   3.790317  -1.092790
 C      3.026015   2.699641  -2.749186
 C      2.258042   1.581833  -3.095950
 H      2.049830   1.327162  -4.145562
 C     -0.578883  -3.063315  -2.648766
 H     -1.422186  -3.782389  -2.655735
 H     -0.534712  -2.585322  -3.648690
 H      0.355310  -3.637615  -2.487877
 C      1.054459   0.570094   2.071429
 C      0.004377  -1.503997   1.467094
 H      1.284968  -1.895895  -1.267836
 H      2.117576  -1.256502   0.640842
 C      3.031605  -2.574406   0.063023
 C      4.402881  -1.936547   0.229855
 C      2.579162  -3.564187   1.149202
 C      5.099643  -1.906984   1.460733
 C      5.039511  -1.409785  -0.917426
 C      6.396770  -1.370650   1.538124
 C      6.330540  -0.864648  -0.840371
 H      4.495536  -1.456947  -1.873120
 C      7.017189  -0.846168   0.389103
 H      6.927028  -1.362407   2.503993
 H      6.810369  -0.458637  -1.745721
 O      2.620582  -2.775419  -1.127216
 H      3.462164   3.351065  -3.522400
 N      3.024085   2.311574   0.946881
 C      3.364424   3.668775   1.354768
 H      2.670685   4.398550   0.894433
 H      3.258593   3.738684   2.457135
 H      4.414181   3.962845   1.102499
 C      3.856936   1.301013   1.605321
 H      3.547755   0.293103   1.278628
 H      4.936895   1.447964   1.360414
 H      3.726140   1.365131   2.703547
 H      8.033950  -0.426357   0.451275
 H      4.629324  -2.303085   2.373541
 H      2.726708  -3.148340   2.166655
 H      1.489669  -3.715058   1.026527
 C      3.303901  -4.915437   1.003057
 H      4.399477  -4.817440   1.152267
 H      2.925899  -5.645990   1.747918
 H      3.136296  -5.339489  -0.008330

4/TSHy-fRSR-re-16ke
Frequencies, energies and thermodynamic properties:
 Lowest Vibrational Mode (1/cm) =                          -373.5685
 E(RB-P86) (a.u.) =                                        -4924.61053726
 Thermal correction to Enthalpy (a.u.) =                    0.827560
 Thermal correction to Gibbs Free Energy (a.u.) =           0.690307
 Total Entropy (cal/Kmol) =                                 288.873
 E(RPBE1PBE) (a.u.) =                                      -4923.91139698
Optimised cartesian coordinates (Angstrom):
 Fe    -4.300534  -1.410631  -0.575767
 Mn     0.316684  -0.300029   0.478473
 P     -1.625967   0.841604   0.243155
 O      0.860826   0.989622   3.070544
 O     -0.782831  -2.428795   2.165140
 N     -0.043442  -1.299837  -1.327258
 N      1.435441   0.932826  -0.857790
 C     -2.820101  -0.015883  -0.867346
 C     -2.530641  -1.243638  -1.605495
 C     -3.631335  -1.467046  -2.515079
 H     -3.734236  -2.314805  -3.203909
 C     -4.596571  -0.415823  -2.341615
 H     -5.553929  -0.326735  -2.872413
 C     -4.107826   0.473976  -1.325162
 H     -4.617873   1.375754  -0.962871
 C     -4.123557  -2.359547   1.244487
 H     -3.215289  -2.386225   1.860175
 C     -4.466938  -3.305061   0.215332
 H     -3.873681  -4.180579  -0.081232
 C     -5.713588  -2.889471  -0.375114
 H     -6.233631  -3.388333  -1.203922
```

C	-6.142645	-1.687040	0.292662
H	-7.047821	-1.109508	0.061656
C	-5.159469	-1.357449	1.291535
H	-5.181718	-0.486571	1.959288
C	-1.455856	2.520574	-0.551484
C	-0.856722	3.551431	0.212463
H	-0.558147	3.356388	1.255269
C	-0.649196	4.825411	-0.341139
H	-0.199638	5.621362	0.274090
C	-1.014766	5.086420	-1.676139
H	-0.850436	6.085232	-2.110656
C	-1.591236	4.064224	-2.448778
H	-1.880868	4.257111	-3.494219
C	-1.812591	2.789985	-1.890793
H	-2.285610	2.008537	-2.505288
C	-2.664170	1.283602	1.731392
C	-2.541066	0.555431	2.934473
H	-1.807694	-0.258192	3.013419
C	-3.355662	0.856355	4.041653
H	-3.242903	0.277374	4.972121
C	-4.304803	1.890648	3.964272
H	-4.940582	2.126931	4.832344
C	-4.431828	2.627027	2.772049
H	-5.167743	3.443958	2.701222
C	-3.615642	2.329102	1.666933
H	-3.716129	2.927627	0.748324
C	-1.294420	-2.122081	-1.465264
H	-1.364515	-2.675760	-0.508189
C	0.197041	-0.423794	-2.485138
H	0.541707	-0.988625	-3.379766
H	-0.766471	0.051951	-2.776150
C	1.176965	0.676188	-2.172406
C	2.273569	1.989281	-0.567840
C	2.769775	2.831049	-1.594076
H	3.432198	3.666171	-1.330984
C	2.478770	2.561032	-2.933167
C	1.687837	1.445000	-3.231020
H	1.434512	1.176822	-4.267247
C	-1.175832	-3.165386	-2.589469
H	-2.031184	-3.869149	-2.552596
H	-1.161537	-2.704545	-3.598327
H	-0.246852	-3.754130	-2.453163
C	0.700653	0.521620	1.994201
C	-0.387443	-1.555820	1.461165
H	0.752562	-2.007558	-1.294335
H	1.691506	-1.311462	0.535737
C	2.551502	-2.654636	-0.008555
C	2.260919	-3.519564	1.218707
O	2.010238	-2.978240	-1.117502
H	2.887078	3.197359	-3.733659
N	2.629041	2.226157	0.765645
C	2.988386	3.588281	1.140125
H	2.277319	4.312894	0.698538
H	2.928383	3.674038	2.244852
H	4.027265	3.876428	0.840599
C	3.489558	1.224127	1.401728
H	3.169825	0.211358	1.100767
H	4.558356	1.370646	1.111058
H	3.404161	1.302530	2.503503
H	2.451093	-3.006101	2.180366
H	1.207062	-3.849915	1.195907
H	2.910521	-4.421837	1.169917
C	3.913713	-1.985248	-0.074606
C	4.820936	-1.994038	0.986730
C	4.311376	-1.393687	-1.316219
C	6.126347	-1.427479	0.859106
H	4.549467	-2.445440	1.953416
C	5.564386	-0.823615	-1.470919
H	3.591897	-1.421004	-2.148109
C	7.068014	-1.438686	1.935832
C	6.511164	-0.822907	-0.397326
H	5.853453	-0.368599	-2.432732
C	8.330597	-0.877056	1.783749
H	6.774808	-1.900636	2.892875
C	7.815844	-0.255305	-0.521177
C	8.707551	-0.280579	0.546133
H	9.045303	-0.891671	2.621948

```
 H      8.106265   0.204559  -1.480062
 H      9.710881   0.161148   0.437183

4/TSHy-fRSR-re-17ke
Frequencies, energies and thermodynamic properties:
 Lowest Vibrational Mode (1/cm) =                          -393.5453
 E(RB-P86) (a.u.) =                                        -4908.32637647
 Thermal correction to Enthalpy (a.u.) =                    0.779111
 Thermal correction to Gibbs Free Energy (a.u.) =           0.646455
 Total Entropy (cal/Kmol) =                                 279.199
 E(RPBE1PBE) (a.u.) =                                      -4907.66967616
Optimised cartesian coordinates (Angstrom):
 Fe    -4.002308  -1.351372  -0.681507
 Mn     0.620857  -0.304104   0.411519
 P     -1.324199   0.848525   0.263268
 O      1.182867   0.801608   3.083241
 O     -0.450636  -2.553467   1.951655
 N      0.249012  -1.180302  -1.455759
 N      1.729237   1.017747  -0.849024
 C     -2.525255   0.061773  -0.891110
 C     -2.239609  -1.113908  -1.710987
 C     -3.346638  -1.278470  -2.625302
 H     -3.453507  -2.078650  -3.368368
 C     -4.311785  -0.242571  -2.375786
 H     -5.273011  -0.120026  -2.892763
 C     -3.816841   0.578718  -1.306064
 H     -4.325311   1.453286  -0.880540
 C     -3.811569  -2.426826   1.065903
 H     -2.898328  -2.502495   1.670203
 C     -4.170663  -3.295922  -0.023463
 H     -3.585329  -4.151201  -0.387143
 C     -5.420796  -2.833620  -0.570212
 H     -5.951977  -3.270342  -1.426566
 C     -5.836570  -1.679276   0.185096
 H     -6.740595  -1.082289   0.004623
 C     -4.841502  -1.425570   1.194230
 H     -4.852098  -0.603395   1.921422
 C     -1.165009   2.575771  -0.423023
 C     -0.568107   3.559984   0.401673
 H     -0.264701   3.302254   1.429297
 C     -0.368729   4.866993  -0.072179
 H      0.079257   5.625261   0.590000
 C     -0.740368   5.208940  -1.387067
 H     -0.582200   6.233689  -1.758973
 C     -1.315160   4.234186  -2.219894
 H     -1.609819   4.490403  -3.250210
 C     -1.528635   2.926702  -1.741471
 H     -2.000837   2.182780  -2.401437
 C     -2.350721   1.188605   1.785616
 C     -2.216473   0.381513   2.936010
 H     -1.480261  -0.433239   2.954235
 C     -3.022750   0.605121   4.067309
 H     -2.901254  -0.034736   4.955856
 C     -3.974762   1.639684   4.066957
 H     -4.604116   1.815411   4.953873
 C     -4.112853   2.454207   2.927998
 H     -4.851088   3.272050   2.918035
 C     -3.304994   2.233488   1.798896
 H     -3.414440   2.892395   0.923589
 C     -1.000130  -1.995447  -1.638445
 H     -1.059865  -2.611317  -0.719515
 C      0.484866  -0.232711  -2.556204
 H      0.829059  -0.740028  -3.484893
 H     -0.478866   0.260849  -2.815503
 C      1.466394   0.844791  -2.176580
 C      2.563837   2.057565  -0.495213
 C      3.056653   2.962539  -1.467484
 H      3.717120   3.781563  -1.153564
 C      2.764689   2.775441  -2.820472
 C      1.974557   1.679255  -3.185888
 H      1.719005   1.476507  -4.236352
 C     -0.887669  -2.963152  -2.829114
 H     -1.739551  -3.672147  -2.829847
 H     -0.884754  -2.438088  -3.806201
 H      0.044911  -3.555089  -2.739395
 C      1.014530   0.410858   1.977692
 C     -0.072042  -1.626234   1.308919
```

```
 H      1.048008  -1.886178  -1.474853
 H      1.999278  -1.321742   0.379694
 C      2.855590  -2.587723  -0.264170
 C      4.219370  -1.957167  -0.166060
 C      2.753449  -3.497557   0.988832
 C      4.775087  -2.197635   1.110683
 C      4.948924  -1.296191  -1.168318
 C      3.780527  -2.930619   1.993027
 C      6.078860  -1.769926   1.406156
 C      6.255212  -0.862480  -0.886353
 H      4.503184  -1.137177  -2.163025
 C      6.790867  -1.107300   0.391948
 H      6.554047  -1.944682   2.383882
 H      6.873416  -0.348326  -1.637772
 O      2.300147  -2.858663  -1.373898
 H      4.259727  -3.704108   2.627873
 H      3.294565  -2.205940   2.685855
 H      1.724657  -3.588634   1.381468
 H      3.072428  -4.507664   0.643532
 H      3.170915   3.460935  -3.580397
 N      2.917943   2.214436   0.850938
 C      3.263937   3.554485   1.308799
 H      2.545139   4.297643   0.912985
 H      3.204231   3.571090   2.416742
 H      4.299646   3.871063   1.027458
 C      3.792280   1.185282   1.421027
 H      3.485882   0.190726   1.053068
 H      4.859063   1.365478   1.142102
 H      3.705064   1.189605   2.525340
 F      8.054726  -0.695298   0.651316
```

```
4/TSHy-fRSR-re-19ke
Frequencies, energies and thermodynamic properties:
 Lowest Vibrational Mode (1/cm) =                         -379.1293
 E(RB-P86) (a.u.) =                                       -5156.65978125
 Thermal correction to Enthalpy (a.u.) =                   0.933180
 Thermal correction to Gibbs Free Energy (a.u.) =          0.782666
 Total Entropy (cal/Kmol) =                                316.783
 E(RPBE1PBE) (a.u.) =                                     -5155.95152415
Optimised cartesian coordinates (Angstrom):
 Fe    -4.184634  -2.481015  -0.072764
 Mn    -0.164322   0.254582  -0.083931
 P     -2.361413   0.584228   0.359456
 O      0.663570   1.517469   2.445178
 O      0.103135  -2.210875   1.474472
 N     -0.664205  -0.720836  -1.867225
 N     -0.001195   1.884006  -1.461352
 C     -3.444281  -0.617622  -0.522852
 C     -2.970702  -1.611071  -1.484844
 C     -4.136914  -2.197148  -2.105250
 H     -4.131624  -2.990020  -2.863676
 C     -5.314358  -1.599536  -1.536516
 H     -6.352304  -1.857834  -1.785953
 C     -4.895405  -0.634746  -0.558068
 H     -5.559656  -0.010487   0.053048
 C     -3.162244  -3.371579   1.479289
 H     -2.163992  -3.081696   1.831873
 C     -3.440380  -4.331047   0.443424
 H     -2.694172  -4.905375  -0.122004
 C     -4.868209  -4.389687   0.260595
 H     -5.400506  -5.010886  -0.472194
 C     -5.473312  -3.467593   1.187719
 H     -6.548087  -3.263498   1.284884
 C     -4.419822  -2.836225   1.939108
 H     -4.547168  -2.067665   2.712397
 C     -3.040922   2.231091  -0.193453
 C     -2.660340   3.381030   0.539953
 H     -2.025629   3.274536   1.434569
 C     -3.094251   4.657714   0.147083
 H     -2.805226   5.539404   0.741423
 C     -3.896404   4.811967  -1.000336
 H     -4.235078   5.813610  -1.309309
 C     -4.262545   3.679195  -1.746658
 H     -4.889740   3.787916  -2.646005
 C     -3.840217   2.396784  -1.345631
 H     -4.155673   1.519207  -1.930633
 C     -3.001680   0.533233   2.112868
```

C	-2.275840	-0.135023	3.122485
H	-1.309882	-0.602364	2.888695
C	-2.779266	-0.215984	4.433999
H	-2.196522	-0.740331	5.208063
C	-4.015433	0.370583	4.757060
H	-4.407888	0.308480	5.784538
C	-4.744658	1.044413	3.759984
H	-5.711695	1.513442	4.002489
C	-4.240147	1.129040	2.450610
H	-4.816076	1.675914	1.687990
C	-1.521133	-1.953534	-1.802352
H	-1.102780	-2.532535	-0.955604
C	-1.100338	0.228909	-2.902813
H	-0.854119	-0.122941	-3.929356
H	-2.209742	0.314342	-2.865315
C	-0.525108	1.604340	-2.689733
C	0.449390	3.168459	-1.239115
C	0.294238	4.178221	-2.220717
H	0.661754	5.191676	-2.012410
C	-0.255668	3.873587	-3.468025
C	-0.650423	2.553943	-3.717131
H	-1.081642	2.253198	-4.683317
C	-1.375293	-2.823386	-3.062936
H	-1.870273	-3.803713	-2.913361
H	-1.824311	-2.351797	-3.960963
H	-0.300857	-3.012494	-3.258726
C	0.350550	1.082468	1.388635
C	-0.063303	-1.218414	0.839675
H	0.305640	-1.065572	-2.154368
H	1.425793	-0.170907	-0.537630
O	1.799807	-1.445210	-2.487669
H	-0.349854	4.650274	-4.242805
N	1.067659	3.467976	-0.017853
C	1.001586	4.845954	0.453682
H	-0.019417	5.252882	0.321399
H	1.241059	4.856432	1.537136
H	1.728871	5.528704	-0.053324
C	2.381058	2.860388	0.214164
H	2.382410	1.816579	-0.144845
H	3.185451	3.428800	-0.314883
H	2.604607	2.852739	1.299041
C	2.490422	-1.019406	-1.499909
C	3.438037	0.113252	-1.740516
C	2.967685	-2.000578	-0.422337
C	4.605734	0.267173	-1.069173
H	3.186317	0.775833	-2.586112
C	3.764552	-1.301763	0.681743
H	3.603244	-2.745561	-0.954698
C	5.032335	-0.584229	0.127031
H	5.306757	1.056189	-1.393170
H	4.051458	-2.019701	1.477914
H	2.105866	-2.557270	-0.007905
H	3.101497	-0.543091	1.149101
C	5.681510	0.365483	1.168359
C	6.880662	1.039029	0.829387
C	5.132753	0.602110	2.446825
C	7.502919	1.920166	1.727254
H	7.343639	0.860879	-0.155026
C	5.757482	1.482184	3.353327
H	4.205718	0.099478	2.757042
C	6.941981	2.147286	2.998737
H	8.434833	2.429952	1.434151
H	5.307930	1.644298	4.346241
H	7.428666	2.835927	3.707596
C	6.082787	-1.652825	-0.284596
C	6.371347	-1.956071	-1.631722
C	6.754944	-2.386420	0.721893
C	7.303065	-2.958844	-1.966077
H	5.858074	-1.403812	-2.434206
C	7.679103	-3.390809	0.392625
H	6.555325	-2.162359	1.782519
C	7.959914	-3.681250	-0.956639
H	7.512547	-3.173945	-3.026403
H	8.187440	-3.948818	1.195524
H	8.688112	-4.465979	-1.216667

4/TSHy-fRSR-re-20ke

Frequencies, energies and thermodynamic properties:

Lowest Vibrational Mode (1/cm) =	-354.9930
E(RB-P86) (a.u.) =	-5224.67841184
Thermal correction to Enthalpy (a.u.) =	0.887698
Thermal correction to Gibbs Free Energy (a.u.) =	0.740020
Total Entropy (cal/Kmol) =	310.813
E(RPBE1PBE) (a.u.) =	-5223.98963782

Optimised cartesian coordinates (Angstrom):

Fe	-4.009947	-2.307940	-0.737992
Mn	0.024382	0.234358	0.242553
P	-2.208450	0.578709	0.417987
O	0.471968	1.094602	3.023983
O	0.004081	-2.442691	1.434681
N	-0.223596	-0.458990	-1.722029
N	0.412553	2.031339	-0.833038
C	-3.175115	-0.430265	-0.782126
C	-2.586208	-1.286097	-1.810367
C	-3.661127	-1.721009	-2.673390
H	-3.561765	-2.389775	-3.537488
C	-4.896610	-1.167141	-2.190060
H	-5.891967	-1.340704	-2.620590
C	-4.605607	-0.380702	-1.023735
H	-5.338982	0.171034	-0.421939
C	-3.239789	-3.469136	0.780518
H	-2.296615	-3.279791	1.309186
C	-3.386141	-4.245141	-0.422614
H	-2.578513	-4.756502	-0.963588
C	-4.773902	-4.214041	-0.808206
H	-5.208226	-4.691797	-1.696646
C	-5.486608	-3.420496	0.160334
H	-6.559814	-3.188106	0.138859
C	-4.539141	-2.957741	1.140655
H	-4.760542	-2.312681	2.000743
C	-2.765517	2.317559	0.036599
C	-2.460567	3.326969	0.981708
H	-1.955258	3.059493	1.923921
C	-2.805842	4.666020	0.736436
H	-2.578462	5.435521	1.491617
C	-3.441000	5.025121	-0.468324
H	-3.710434	6.075700	-0.661065
C	-3.728466	4.034042	-1.421849
H	-4.224179	4.303729	-2.368201
C	-3.395371	2.688580	-1.171381
H	-3.648231	1.923203	-1.921136
C	-3.099255	0.293002	2.034186
C	-2.546734	-0.555733	3.017671
H	-1.569719	-1.028761	2.851187
C	-3.238607	-0.810541	4.216154
H	-2.789349	-1.474310	4.971985
C	-4.492859	-0.220317	4.450911
H	-5.032853	-0.418760	5.390393
C	-5.050294	0.632179	3.480164
H	-6.029617	1.105671	3.655355
C	-4.357558	0.890229	2.284441
H	-4.800755	1.574442	1.544373
C	-1.114522	-1.646283	-1.965963
H	-0.840945	-2.360928	-1.165036
C	-0.481500	0.650671	-2.655102
H	-0.084818	0.448777	-3.674960
H	-1.582729	0.762616	-2.772590
C	0.063833	1.961069	-2.149745
C	0.854398	3.246893	-0.353499
C	0.842900	4.402280	-1.174080
H	1.193467	5.357803	-0.762346
C	0.459714	4.313785	-2.514196
C	0.089970	3.062058	-3.021261
H	-0.207332	2.929627	-4.071933
C	-0.808308	-2.327079	-3.311028
H	-1.346738	-3.293019	-3.384516
H	-1.108086	-1.706788	-4.180385
H	0.276945	-2.539979	-3.382517
C	0.321700	0.824605	1.880720
C	-0.039092	-1.361808	0.939987
H	0.760698	-0.815724	-1.899679
H	1.657808	-0.144839	-0.039290
H	0.478143	5.204752	-3.160937
N	1.316052	3.326246	0.964887

```
 C      1.226622   4.615150   1.639274
 H      0.243181   5.086593   1.448547
 H      1.323091   4.444585   2.731469
 H      2.032597   5.332612   1.343064
 C      2.568488   2.623024   1.263873
 H      2.586488   1.650551   0.742180
 H      3.452025   3.223859   0.940423
 H      2.645759   2.433814   2.351785
 C      4.998113   0.375614  -0.160197
 C      5.562387  -1.941488   0.416143
 C      2.864315  -0.965779  -0.907781
 C      3.073031  -2.021508   0.202765
 O      2.302913  -1.332440  -1.987246
 C      3.862234   0.185118  -0.998866
 C      3.656980   1.106135  -2.054760
 C      5.846139   1.487866  -0.368985
 C      4.488870   2.213924  -2.248120
 H      2.807059   0.917456  -2.727131
 C      5.587668   2.412016  -1.389035
 H      6.724063   1.607836   0.281082
 H      4.289328   2.918904  -3.070440
 H      6.261306   3.271987  -1.531524
 C      4.416408  -2.693696   0.062785
 C      4.591380  -4.009189  -0.412516
 C      6.853134  -2.498855   0.319350
 C      5.876644  -4.572722  -0.504158
 H      3.706714  -4.600095  -0.699721
 C      7.007172  -3.821089  -0.132850
 H      7.728470  -1.891318   0.595676
 H      5.997055  -5.605502  -0.867728
 H      8.014674  -4.259263  -0.209222
 N      5.369319  -0.580217   0.845375
 C      5.597268  -0.157317   2.174105
 O      5.465923   1.020964   2.523176
 N      5.983736  -1.150289   3.041045
 H      5.985948  -0.900554   4.030577
 H      5.885953  -2.139482   2.805838
 H      3.015058  -1.535557   1.197356
 H      2.248596  -2.752008   0.121216

4/TSHy-fRSR-re-22ke
Frequencies, energies and thermodynamic properties:
 Lowest Vibrational Mode (1/cm) =                          -264.3045
 E(RB-P86) (a.u.) =                                        -4807.93640656
 Thermal correction to Enthalpy (a.u.) =                    0.762786
 Thermal correction to Gibbs Free Energy (a.u.) =           0.633060
 Total Entropy (cal/Kmol) =                                 273.032
 E(RPBE1PBE) (a.u.) =                                      -4807.24492254
Optimised cartesian coordinates (Angstrom):
 Fe    -3.839462  -1.294294  -0.732751
 Mn     0.763441  -0.318602   0.481866
 P     -1.146932   0.866931   0.239968
 O      1.261096   0.892460   3.121640
 O     -0.382234  -2.508517   2.054239
 N      0.440642  -1.265458  -1.353326
 N      1.962075   0.907658  -0.780457
 C     -2.321670   0.073571  -0.934366
 C     -2.036490  -1.135950  -1.703639
 C     -3.113761  -1.303835  -2.652611
 H     -3.214806  -2.126240  -3.371708
 C     -4.059940  -0.236526  -2.473721
 H     -4.997990  -0.107751  -3.030184
 C     -3.583241   0.608308  -1.414334
 H     -4.083992   1.509351  -1.037628
 C     -3.735702  -2.309519   1.056625
 H     -2.846313  -2.381952   1.695840
 C     -4.075314  -3.209911  -0.013466
 H     -3.496830  -4.090100  -0.324936
 C     -5.294484  -2.741356  -0.621262
 H     -5.804403  -3.197380  -1.480455
 C     -5.710812  -1.551898   0.076967
 H     -6.594359  -0.943020  -0.157163
 C     -4.746880  -1.282784   1.112001
 H     -4.765369  -0.435068   1.809044
 C     -0.910344   2.565496  -0.490397
 C     -0.316081   3.557206   0.327261
 H     -0.063364   3.323971   1.374265
```

```
 C     -0.055617   4.840998  -0.179097
 H      0.389775   5.606329   0.476639
 C     -0.362919   5.150642  -1.518406
 H     -0.156957   6.157196  -1.915692
 C     -0.934725   4.167307  -2.343234
 H     -1.179170   4.398723  -3.392365
 C     -1.208812   2.883282  -1.833501
 H     -1.676952   2.132659  -2.488753
 C     -2.213815   1.276990   1.715143
 C     -2.136908   0.503608   2.893574
 H     -1.420338  -0.325986   2.962526
 C     -2.975664   0.780644   3.988779
 H     -2.899398   0.166705   4.900303
 C     -3.902717   1.835642   3.923463
 H     -4.557636   2.052998   4.782173
 C     -3.983026   2.617145   2.756131
 H     -4.701072   3.450606   2.695757
 C     -3.142707   2.343135   1.663004
 H     -3.206327   2.976256   0.764452
 C     -0.827693  -2.050284  -1.554733
 H     -0.937034  -2.632304  -0.618697
 C      0.749198  -0.374803  -2.484527
 H      1.119947  -0.931744  -3.373542
 H     -0.192107   0.124960  -2.806010
 C      1.738110   0.697396  -2.109655
 C      2.818940   1.931573  -0.431639
 C      3.359978   2.796249  -1.415338
 H      4.033045   3.607007  -1.106803
 C      3.099985   2.577142  -2.769953
 C      2.296055   1.487913  -3.127457
 H      2.068195   1.258952  -4.178904
 C     -0.703539  -3.059868  -2.708491
 H     -1.579514  -3.738620  -2.718861
 H     -0.646778  -2.567851  -3.700916
 H      0.202456  -3.681082  -2.564192
 C      1.121535   0.456293   2.030201
 C      0.022054  -1.602899   1.400179
 H      1.203862  -2.006357  -1.302213
 H      2.088310  -1.374129   0.638467
 C      2.918767  -2.762398   0.031794
 C      4.291511  -2.124214  -0.022828
 C      2.850119  -3.382023   1.410851
 C      4.947435  -2.394878   1.210474
 C      4.944263  -1.460248  -1.063232
 C      4.026037  -3.167416   2.068404
 C      6.267952  -1.962201   1.414669
 C      6.274365  -1.026859  -0.856588
 H      4.431752  -1.281502  -2.022232
 C      6.923529  -1.272651   0.369638
 H      6.790163  -2.164973   2.363907
 H      6.809640  -0.496804  -1.660549
 O      2.278629  -3.157211  -0.987154
 H      3.543422   3.230812  -3.537059
 N      3.143801   2.112581   0.917286
 C      3.520798   3.451805   1.351784
 H      2.839609   4.207894   0.915910
 H      3.428412   3.499157   2.456586
 H      4.574512   3.726089   1.094395
 C      3.957587   1.067856   1.547071
 H      3.637725   0.074560   1.186655
 H      5.040204   1.206862   1.310345
 H      3.823497   1.100782   2.646250
 H      7.960754  -0.929447   0.513283
 H      1.967475  -3.924563   1.774136
 H      4.274235  -3.505677   3.085766

4/TSHy-fRSR-re-24ke
Frequencies, energies and thermodynamic properties:
 Lowest Vibrational Mode (1/cm) =                          -376.9150
 E(RB-P86) (a.u.) =                                        -4848.40451871
 Thermal correction to Enthalpy (a.u.) =                    0.814076
 Thermal correction to Gibbs Free Energy (a.u.) =           0.678467
 Total Entropy (cal/Kmol) =                                 285.412
 E(RPBE1PBE) (a.u.) =                                      -4847.71357581
Optimised cartesian coordinates (Angstrom):
 Fe    -3.821848  -1.279863  -0.814813
 Mn     0.725071  -0.208481   0.537053
```

P	-1.202212	0.941930	0.234805
O	1.103425	0.988036	3.201397
O	-0.482368	-2.363605	2.114661
N	0.471210	-1.137572	-1.325524
N	1.915993	1.077058	-0.684808
C	-2.330304	0.124863	-0.971030
C	-1.997584	-1.073474	-1.738460
C	-3.046776	-1.262346	-2.714656
H	-3.109915	-2.082998	-3.440129
C	-4.022357	-0.218555	-2.554865
H	-4.949012	-0.108974	-3.134129
C	-3.592033	0.631662	-1.479958
H	-4.123435	1.518374	-1.111430
C	-3.748230	-2.292359	0.978152
H	-2.878284	-2.338449	1.645945
C	-4.025567	-3.201499	-0.102443
H	-3.411659	-4.064337	-0.394083
C	-5.236657	-2.768028	-0.751253
H	-5.704214	-3.238162	-1.626800
C	-5.710432	-1.591475	-0.067672
H	-6.602903	-1.008294	-0.331596
C	-4.789886	-1.295365	0.999169
H	-4.855578	-0.448656	1.694702
C	-0.994197	2.649821	-0.487233
C	-0.445489	3.653063	0.347982
H	-0.205476	3.419546	1.397974
C	-0.215568	4.948291	-0.143978
H	0.193562	5.721932	0.525565
C	-0.508068	5.259255	-1.486265
H	-0.326420	6.274885	-1.872215
C	-1.033967	4.265185	-2.328508
H	-1.266376	4.497081	-3.380308
C	-1.277716	2.969352	-1.832977
H	-1.710907	2.210149	-2.502332
C	-2.327863	1.331964	1.673947
C	-2.274162	0.565754	2.858434
H	-1.542663	-0.247147	2.956708
C	-3.155980	0.828095	3.923217
H	-3.096408	0.219807	4.839831
C	-4.104660	1.860615	3.821682
H	-4.793345	2.066196	4.656580
C	-4.162967	2.634876	2.648263
H	-4.897609	3.451157	2.559004
C	-3.279338	2.375927	1.586072
H	-3.326997	3.004332	0.683301
C	-0.766106	-1.954457	-1.570185
H	-0.884873	-2.553410	-0.645443
C	0.775396	-0.215835	-2.431193
H	1.167391	-0.744873	-3.328090
H	-0.169884	0.275950	-2.753911
C	1.739012	0.866011	-2.021113
C	2.731192	2.124954	-0.309268
C	3.291702	2.997353	-1.275204
H	3.934948	3.823236	-0.944158
C	3.088046	2.770043	-2.638004
C	2.316000	1.666799	-3.020502
H	2.127145	1.433233	-4.078671
C	-0.582802	-2.940387	-2.737361
H	-1.427188	-3.657428	-2.771676
H	-0.534194	-2.429933	-3.720930
H	0.349944	-3.521665	-2.593715
C	1.013771	0.557886	2.100925
C	-0.036529	-1.491323	1.442454
H	1.269695	-1.841265	-1.278588
H	2.090316	-1.238095	0.632288
C	3.013724	-2.512366	0.058855
C	4.361866	-1.808944	0.140740
C	5.189528	-1.859836	1.285933
C	4.846119	-1.150922	-1.012826
C	6.463663	-1.264651	1.276589
C	6.113343	-0.547902	-1.020872
H	4.207219	-1.141187	-1.908823
C	6.930069	-0.604265	0.125354
H	7.098659	-1.321708	2.175385
H	6.472788	-0.037931	-1.929358
O	2.564213	-2.774002	-1.107057
H	3.547394	3.430141	-3.390140

```
 N      2.996316   2.325851   1.050920
 C      3.326496   3.677674   1.484684
 H      2.645451   4.413755   1.015595
 H      3.192047   3.733686   2.584773
 H      4.382746   3.974049   1.263894
 C      3.815596   1.308741   1.716667
 H      3.520328   0.304674   1.366466
 H      4.900894   1.463049   1.502062
 H      3.655388   1.357090   2.811782
 H      7.928799  -0.139023   0.119344
 H      4.846915  -2.376681   2.194586
 C      2.682252  -3.461029   1.198462
 C      1.628279  -4.519533   0.995435
 C      3.081915  -4.924997   0.998714
 H      2.796919  -3.057943   2.216701
 H      1.113488  -4.507062   0.021614
 H      0.998146  -4.782650   1.858359
 H      3.487288  -5.468798   1.867612
 H      3.541165  -5.188757   0.031682
```

```
4/TSHy-fRSR-re-26ke
Frequencies, energies and thermodynamic properties:
 Lowest Vibrational Mode (1/cm) =                         -560.7646
 E(RB-P86) (a.u.) =                                       -4773.45643481
 Thermal correction to Enthalpy (a.u.) =                   0.822902
 Thermal correction to Gibbs Free Energy (a.u.) =          0.691077
 Total Entropy (cal/Kmol) =                                277.450
 E(RPBE1PBE) (a.u.) =                                     -4772.78061247
Optimised cartesian coordinates (Angstrom):
 Fe    -3.557310  -1.515191  -0.811956
 Mn     0.961864  -0.175915   0.428009
 P     -1.067011   0.829774   0.292765
 O      1.424568   0.840807   3.153818
 O     -0.051300  -2.494519   1.904924
 N      0.699958  -1.040721  -1.450996
 N      1.902010   1.329230  -0.793483
 C     -2.171500  -0.003096  -0.926186
 C     -1.780939  -1.129933  -1.770559
 C     -2.843425  -1.337135  -2.728273
 H     -2.871619  -2.118636  -3.497792
 C     -3.882830  -0.375010  -2.482946
 H     -4.831954  -0.298220  -3.030208
 C     -3.479498   0.441944  -1.372624
 H     -4.058660   1.268955  -0.942903
 C     -3.350447  -2.631015   0.908219
 H     -2.453476  -2.662003   1.540166
 C     -3.614176  -3.486483  -0.218669
 H     -2.960050  -4.287581  -0.588437
 C     -4.874773  -3.091763  -0.793707
 H     -5.347112  -3.535133  -1.680574
 C     -5.392141  -1.992863  -0.018665
 H     -6.328621  -1.452545  -0.211850
 C     -4.449841  -1.706101   1.031527
 H     -4.540080  -0.910471   1.782343
 C     -1.061713   2.592336  -0.319355
 C     -0.685291   3.616682   0.582083
 H     -0.446618   3.365826   1.628089
 C     -0.630284   4.956210   0.162053
 H     -0.359641   5.743690   0.883685
 C     -0.924576   5.293555  -1.173040
 H     -0.881652   6.344017  -1.501825
 C     -1.275904   4.280787  -2.081600
 H     -1.507659   4.532630  -3.128875
 C     -1.346332   2.939662  -1.658199
 H     -1.647835   2.163714  -2.378467
 C     -2.153287   1.015265   1.802548
 C     -1.946890   0.214798   2.946654
 H     -1.118260  -0.504258   2.976258
 C     -2.799535   0.321340   4.061417
 H     -2.618996  -0.311852   4.944673
 C     -3.871726   1.229977   4.051211
 H     -4.537771   1.313425   4.924697
 C     -4.083369   2.038260   2.918904
 H     -4.916137   2.759423   2.900660
 C     -3.229442   1.935234   1.807446
 H     -3.400430   2.590223   0.939448
 C     -0.490149  -1.931455  -1.679144
```

```
 H     -0.533659  -2.566067  -0.772492
 C      0.910600  -0.085111  -2.548579
 H      1.397035  -0.559991  -3.429810
 H     -0.080356   0.275407  -2.906612
 C      1.705197   1.123925  -2.128081
 C      2.540635   2.494707  -0.419514
 C      2.917478   3.464584  -1.380569
 H      3.426826   4.379006  -1.049974
 C      2.708374   3.232395  -2.741842
 C      2.108635   2.028242  -3.124749
 H      1.917798   1.788838  -4.181288
 C     -0.286861  -2.870232  -2.880919
 H     -1.097310  -3.625442  -2.919089
 H     -0.284629  -2.328615  -3.849026
 H      0.674787  -3.408499  -2.768154
 C      1.290806   0.495381   2.028806
 C      0.323615  -1.554150   1.283286
 H      1.535297  -1.719577  -1.441976
 H      2.451577  -1.149946   0.386178
 O      2.686446  -2.730008  -1.276979
 H      3.028656   3.972316  -3.491682
 N      2.821426   2.716300   0.938041
 C      2.954539   4.100024   1.381000
 H      2.142991   4.721944   0.957828
 H      2.868198   4.122186   2.486923
 H      3.938580   4.561778   1.115547
 C      3.839606   1.849984   1.535836
 H      3.673415   0.809261   1.208107
 H      4.869555   2.170669   1.241820
 H      3.756910   1.878207   2.639888
 C      3.233872  -2.458854  -0.144218
 C      2.909914  -3.358939   1.015951
 C      4.722132  -1.969311  -0.166620
 C      3.739499  -3.573791   2.064523
 C      5.198447  -1.671075   1.274826
 C      5.057495  -2.866534   2.231759
 H      5.883510  -3.602880   2.081749
 H      5.173847  -2.531845   3.287118
 H      3.459530  -4.320745   2.829930
 H      1.958190  -3.907558   0.930038
 H      6.251849  -1.314437   1.254076
 H      4.590387  -0.832657   1.674141
 C      4.887803  -0.742871  -1.075963
 H      4.476999  -0.958971  -2.082918
 H      4.349801   0.136244  -0.673741
 H      5.959980  -0.474876  -1.186209
 C      5.541580  -3.137050  -0.780308
 H      5.200786  -3.333499  -1.816588
 H      6.620872  -2.874535  -0.808394
 H      5.430499  -4.079748  -0.206653

4/TSHy-fRSR-re-27ke
Frequencies, energies and thermodynamic properties:
 Lowest Vibrational Mode (1/cm) =                          -441.0237
 E(RB-P86) (a.u.) =                                        -4773.47286435
 Thermal correction to Enthalpy (a.u.) =                    0.823337
 Thermal correction to Gibbs Free Energy (a.u.) =           0.689111
 Total Entropy (cal/Kmol) =                                 282.501
 E(RPBE1PBE) (a.u.) =                                      -4772.79064003
Optimised cartesian coordinates (Angstrom):
 Fe    -3.743268  -1.315789  -0.740426
 Mn     0.880781  -0.296364   0.361632
 P     -1.069672   0.854538   0.303914
 O      1.500171   0.611944   3.094832
 O     -0.218585  -2.605491   1.791971
 N      0.501205  -1.048441  -1.550592
 N      1.947999   1.128394  -0.817646
 C     -2.279275   0.121122  -0.877513
 C     -1.991326  -0.999042  -1.771304
 C     -3.106916  -1.118380  -2.682203
 H     -3.214172  -1.872854  -3.471668
 C     -4.079047  -0.109208  -2.359926
 H     -5.046882   0.035412  -2.858617
 C     -3.580333   0.649304  -1.246332
 H     -4.092170   1.490734  -0.762117
 C     -3.534321  -2.491747   0.940158
 H     -2.616885  -2.594436   1.533864
```

C	-3.891980	-3.297470	-0.197167
H	-3.301732	-4.124900	-0.613516
C	-5.148402	-2.813678	-0.709952
H	-5.680528	-3.203102	-1.588246
C	-5.569431	-1.709463	0.114413
H	-6.478986	-1.110165	-0.026350
C	-4.571034	-1.507998	1.132164
H	-4.583371	-0.729971	1.906424
C	-0.938818	2.618770	-0.290096
C	-0.326667	3.558636	0.574190
H	0.014087	3.241693	1.573278
C	-0.159503	4.895224	0.176665
H	0.301490	5.617303	0.869667
C	-0.579842	5.312722	-1.101211
H	-0.447946	6.360971	-1.413044
C	-1.167338	4.382431	-1.974984
H	-1.498093	4.697197	-2.977780
C	-1.347894	3.044741	-1.572558
H	-1.829646	2.335828	-2.263304
C	-2.081187	1.107255	1.853937
C	-1.927857	0.243726	2.960216
H	-1.187365	-0.566413	2.928199
C	-2.721027	0.404309	4.111377
H	-2.584088	-0.278991	4.964655
C	-3.679217	1.431064	4.175642
H	-4.298115	1.557463	5.078194
C	-3.836784	2.301157	3.081182
H	-4.580039	3.113503	3.121885
C	-3.041983	2.143488	1.932422
H	-3.166816	2.845398	1.093414
C	-0.739286	-1.866803	-1.769796
H	-0.781299	-2.540267	-0.891189
C	0.703622	-0.026865	-2.590207
H	1.063005	-0.465002	-3.548199
H	-0.275158	0.451100	-2.820952
C	1.652551	1.055951	-2.147713
C	2.774806	2.150740	-0.401987
C	3.221534	3.143460	-1.308416
H	3.877077	3.946734	-0.947184
C	2.890666	3.063212	-2.663138
C	2.112711	1.983129	-3.097072
H	1.833539	1.860683	-4.153916
C	-0.629014	-2.756557	-3.020091
H	-1.471862	-3.475458	-3.057070
H	-0.642718	-2.171177	-3.962312
H	0.312753	-3.339038	-2.975242
C	1.305870	0.306326	1.966556
C	0.180047	-1.659696	1.190133
H	1.318387	-1.744959	-1.625519
H	2.273552	-1.326992	0.229612
O	2.568492	-2.671631	-1.601710
H	3.259915	3.818245	-3.374480
N	3.173919	2.193311	0.939583
C	3.524711	3.491735	1.501544
H	2.787290	4.258965	1.196214
H	3.502708	3.411507	2.608063
H	4.547620	3.842806	1.214227
C	4.073476	1.126430	1.389400
H	3.769894	0.160009	0.949448
H	5.129957	1.345380	1.096805
H	4.021633	1.034236	2.492250
C	3.088020	-2.513699	-0.433411
C	4.481736	-1.914891	-0.372529
C	2.793945	-3.578440	0.633030
C	5.334920	-2.199136	0.662928
C	3.393962	-3.219976	1.990870
H	3.253334	-4.520159	0.248567
C	4.909370	-3.028300	1.862600
H	3.165321	-4.000036	2.747780
H	1.703301	-3.757409	0.686460
H	2.929167	-2.277100	2.352779
H	5.418409	-4.021100	1.791492
H	5.327028	-2.565519	2.785762
C	4.848812	-1.101565	-1.589285
H	4.788533	-1.725644	-2.505643
H	4.115716	-0.280741	-1.736224
H	5.856765	-0.651846	-1.528659

```
 C      6.781957  -1.768519   0.716019
 H      7.142627  -1.279393  -0.207469
 H      6.950784  -1.067134   1.564210
 H      7.436605  -2.646250   0.915573

4/TSHy-fRSR-re-5ke
Frequencies, energies and thermodynamic properties:
 Lowest Vibrational Mode (1/cm) =                        -373.1014
 E(RB-P86) (a.u.) =                                      -4884.32749008
 Thermal correction to Enthalpy (a.u.) =                  0.792250
 Thermal correction to Gibbs Free Energy (a.u.) =         0.659671
 Total Entropy (cal/Kmol) =                               279.035
 E(RPBE1PBE) (a.u.) =                                    -4883.64872090
Optimised cartesian coordinates (Angstrom):
 Fe    -3.902166  -1.344633  -0.795128
 Mn     0.685824  -0.274308   0.428669
 P     -1.272116   0.857493   0.278296
 O      1.193934   0.749474   3.143354
 O     -0.383516  -2.591505   1.866708
 N      0.363471  -1.099844  -1.470336
 N      1.799348   1.099024  -0.770360
 C     -2.438385   0.091863  -0.924708
 C     -2.119891  -1.053330  -1.775402
 C     -3.203799  -1.200679  -2.719896
 H     -3.284138  -1.977548  -3.490513
 C     -4.186957  -0.184641  -2.459259
 H     -5.137683  -0.056395  -2.993962
 C     -3.726479   0.606928  -1.352449
 H     -4.255140   1.461276  -0.910710
 C     -3.737261  -2.484234   0.914670
 H     -2.835870  -2.577536   1.534290
 C     -4.069587  -3.313497  -0.213531
 H     -3.471762  -4.150836  -0.598018
 C     -5.312199  -2.838350  -0.766424
 H     -5.823811  -3.245287  -1.648941
 C     -5.750218  -1.716183   0.023905
 H     -6.654695  -1.118305  -0.151245
 C     -4.776300  -1.494868   1.060843
 H     -4.806180  -0.700884   1.818161
 C     -1.123685   2.607769  -0.349063
 C     -0.557602   3.572767   0.518961
 H     -0.272516   3.286365   1.544230
 C     -0.365396   4.896544   0.090978
 H      0.058877   5.638738   0.786147
 C     -0.713644   5.275415  -1.220176
 H     -0.561071   6.313328  -1.556176
 C     -1.257797   4.320517  -2.095517
 H     -1.533925   4.605643  -3.123369
 C     -1.464045   2.995897  -1.663342
 H     -1.911968   2.267478  -2.356670
 C     -2.332177   1.133030   1.790181
 C     -2.207900   0.289739   2.915439
 H     -1.460544  -0.515046   2.920179
 C     -3.038516   0.464120   4.037745
 H     -2.924486  -0.203234   4.906841
 C     -4.005387   1.484710   4.053159
 H     -4.653846   1.621928   4.933078
 C     -4.134041   2.334736   2.939279
 H     -4.884000   3.141896   2.942168
 C     -3.301751   2.163346   1.819381
 H     -3.404378   2.849224   0.964205
 C     -0.872312  -1.923559  -1.702283
 H     -0.945047  -2.565410  -0.802458
 C      0.606245  -0.116459  -2.537633
 H      0.976368  -0.590610  -3.473755
 H     -0.360628   0.368445  -2.801306
 C      1.561739   0.964981  -2.106998
 C      2.615012   2.136653  -0.369548
 C      3.107508   3.081737  -1.303068
 H      3.751221   3.899053  -0.952244
 C      2.837899   2.936205  -2.665705
 C      2.072151   1.840073  -3.080012
 H      1.836933   1.668159  -4.140676
 C     -0.724086  -2.855798  -2.916970
 H     -1.567581  -3.573736  -2.955258
 H     -0.706954  -2.303589  -3.878838
 H      0.213143  -3.440119  -2.824922
```

```
 C      1.044048   0.395512   2.022803
 C     -0.006187  -1.635041   1.267205
 H      1.172152  -1.793007  -1.491749
 H      2.075199  -1.259331   0.400429
 C      2.990713  -2.509696  -0.269521
 C      4.344561  -1.851816  -0.199434
 C      2.767463  -3.490151   0.889525
 C      5.171723  -2.016550   0.948299
 C      4.870583  -1.175057  -1.321955
 C      3.360089  -2.970479   2.190911
 C      6.493378  -1.513068   0.943849
 C      6.172878  -0.658838  -1.321612
 H      4.221700  -1.087787  -2.207482
 C      6.985507  -0.837016  -0.180749
 H      7.114431  -1.663980   1.840391
 H      6.563730  -0.131246  -2.205584
 H      8.015290  -0.445089  -0.167885
 O      2.462794  -2.733166  -1.405970
 H      3.244100   3.653265  -3.395931
 N      2.952114   2.248068   0.985217
 C      3.282262   3.573245   1.495118
 H      2.560515   4.324287   1.119960
 H      3.212381   3.548980   2.602279
 H      4.317563   3.908546   1.234958
 C      3.828070   1.204392   1.526000
 H      3.529625   0.222510   1.119264
 H      4.896130   1.398739   1.261920
 H      3.731843   1.168545   2.629078
 H      1.691557  -3.714093   1.017997
 H      3.269412  -4.441016   0.595034
 H      3.286767  -3.717339   3.004884
 H      2.832835  -2.046031   2.515298
 O      4.771163  -2.673446   2.072087
```

```
4/TSHy-fRSR-re-6ke
Frequencies, energies and thermodynamic properties:
 Lowest Vibrational Mode (1/cm) =                           -368.3977
 E(RB-P86) (a.u.) =                                         -4771.07435595
 Thermal correction to Enthalpy (a.u.) =                     0.779180
 Thermal correction to Gibbs Free Energy (a.u.) =            0.647373
 Total Entropy (cal/Kmol) =                                  277.411
 E(RPBE1PBE) (a.u.) =                                       -4770.38626234
Optimised cartesian coordinates (Angstrom):
 Fe    -3.818985  -1.266002  -0.705399
 Mn     0.776314  -0.354690   0.616624
 P     -1.073773   0.875682   0.173874
 O      1.219896   1.062415   3.160492
 O     -0.563933  -2.305549   2.343792
 N      0.480682  -1.446779  -1.146970
 N      2.052199   0.728293  -0.711435
 C     -2.236746   0.017862  -0.969258
 C     -1.972618  -1.270404  -1.607184
 C     -3.017135  -1.488943  -2.581720
 H     -3.123148  -2.372680  -3.223287
 C     -3.924335  -0.374174  -2.546564
 H     -4.833349  -0.264341  -3.153097
 C     -3.454859   0.550563  -1.552400
 H     -3.932091   1.501905  -1.284572
 C     -3.831076  -2.110255   1.174138
 H     -2.970835  -2.158519   1.854415
 C     -4.169924  -3.092916   0.178850
 H     -3.620810  -4.022240  -0.024253
 C     -5.341921  -2.633448  -0.521760
 H     -5.838654  -3.147218  -1.355729
 C     -5.729944  -1.366624   0.044269
 H     -6.574775  -0.745999  -0.283340
 C     -4.795438  -1.041313   1.090128
 H     -4.800854  -0.130602   1.702668
 C     -0.750728   2.491276  -0.700465
 C     -0.146251   3.531162   0.046760
 H      0.069636   3.380927   1.116977
 C      0.171307   4.757707  -0.559111
 H      0.623139   5.562961   0.042217
 C     -0.087799   4.959844  -1.928744
 H      0.162600   5.921440  -2.404215
 C     -0.668036   3.926600  -2.683631
 H     -0.874301   4.073468  -3.755983
```

```
 C     -0.999719   2.700664  -2.074380
 H     -1.473235   1.910752  -2.677576
 C     -2.184727   1.466023   1.554681
 C     -2.183196   0.814213   2.806873
 H     -1.502219  -0.027912   2.989074
 C     -3.053494   1.228388   3.832173
 H     -3.035484   0.707819   4.802948
 C     -3.937983   2.300865   3.622488
 H     -4.617498   2.625569   4.426496
 C     -3.943764   2.961736   2.380259
 H     -4.627922   3.807616   2.205932
 C     -3.071594   2.550998   1.357181
 H     -3.076930   3.091905   0.398189
 C     -0.805133  -2.205579  -1.322163
 H     -0.974128  -2.687391  -0.339115
 C      0.851043  -0.664053  -2.336650
 H      1.222780  -1.305036  -3.166823
 H     -0.060179  -0.156805  -2.726237
 C      1.870104   0.399388  -2.022815
 C      2.926200   1.757512  -0.428863
 C      3.537191   2.503336  -1.467335
 H      4.225008   3.319559  -1.210217
 C      3.324513   2.160134  -2.804361
 C      2.495098   1.068674  -3.087948
 H      2.298591   0.745989  -4.120963
 C     -0.673439  -3.328656  -2.365168
 H     -1.569939  -3.980022  -2.344122
 H     -0.562852  -2.939875  -3.398163
 H      0.207493  -3.956715  -2.124806
 C      1.104779   0.537911   2.104532
 C     -0.068034  -1.503338   1.619479
 H      1.230289  -2.192890  -1.014885
 H      2.081896  -1.434868   0.832528
 C      2.896952  -2.854753   0.426827
 C      4.303180  -2.274296   0.441386
 C      2.457180  -3.633742   1.667858
 C      5.108246  -2.233408   1.603311
 C      4.859860  -1.832479  -0.781102
 C      6.432331  -1.763069   1.542091
 C      6.176701  -1.352017  -0.841895
 H      4.234099  -1.893105  -1.684509
 C      6.970955  -1.317972   0.321247
 H      7.047955  -1.746281   2.455834
 H      6.592746  -1.009688  -1.803332
 O      2.422812  -3.206828  -0.703531
 H      3.821630   2.720267  -3.611555
 N      3.203647   2.062214   0.909239
 C      3.614849   3.424703   1.223789
 H      2.977298   4.155742   0.690088
 H      3.487015   3.585331   2.314312
 H      4.686232   3.637083   0.980464
 C      3.961090   1.058849   1.663231
 H      3.606736   0.047475   1.398787
 H      5.053731   1.130372   1.441593
 H      3.804721   1.209439   2.749654
 H      8.008078  -0.948990   0.274747
 H      4.706583  -2.574477   2.569222
 H      2.596873  -3.080584   2.616025
 H      1.391704  -3.909413   1.571912
 H      3.056690  -4.570124   1.719308
```

```
4/TSHy-fRSR-re-7ke
Frequencies, energies and thermodynamic properties:
 Lowest Vibrational Mode (1/cm) =                          -387.0428
 E(RB-P86) (a.u.) =                                        -4848.44888979
 Thermal correction to Enthalpy (a.u.) =                    0.815661
 Thermal correction to Gibbs Free Energy (a.u.) =           0.683471
 Total Entropy (cal/Kmol) =                                 278.218
 E(RPBE1PBE) (a.u.) =                                      -4847.76102023
Optimised cartesian coordinates (Angstrom):
 Fe    -3.911400  -1.300670  -0.808965
 Mn     0.688704  -0.299479   0.436742
 P     -1.252508   0.858332   0.284353
 O      1.200285   0.701569   3.158897
 O     -0.431920  -2.594984   1.872195
 N      0.361140  -1.116828  -1.463539
 N      1.817825   1.066612  -0.753711
```

C	-2.425483	0.114473	-0.926580
C	-2.119894	-1.032658	-1.779274
C	-3.201078	-1.161071	-2.729597
H	-3.288961	-1.934354	-3.503015
C	-4.170326	-0.131272	-2.470645
H	-5.116225	0.013030	-3.009829
C	-3.703579	0.649923	-1.359057
H	-4.221570	1.510837	-0.917477
C	-3.770868	-2.449203	0.897154
H	-2.873205	-2.557970	1.519732
C	-4.110965	-3.268968	-0.235656
H	-3.524237	-4.113801	-0.620857
C	-5.343940	-2.772909	-0.791871
H	-5.857964	-3.168680	-1.678063
C	-5.768287	-1.647293	0.001060
H	-6.662997	-1.035250	-0.175379
C	-4.795467	-1.444832	1.042891
H	-4.816433	-0.653434	1.803240
C	-1.079226	2.609015	-0.336571
C	-0.514201	3.567140	0.539616
H	-0.244219	3.276352	1.567703
C	-0.304117	4.889730	0.116222
H	0.118477	5.626856	0.817800
C	-0.632700	5.274010	-1.198417
H	-0.466179	6.310940	-1.530824
C	-1.175413	4.325704	-2.081804
H	-1.436322	4.615035	-3.112463
C	-1.399878	3.002592	-1.654197
H	-1.846748	2.279692	-2.353965
C	-2.316217	1.144615	1.792307
C	-2.206834	0.299430	2.917701
H	-1.468990	-0.513982	2.925491
C	-3.040603	0.482700	4.036321
H	-2.938168	-0.186448	4.905495
C	-3.995862	1.514181	4.048012
H	-4.646784	1.658271	4.925019
C	-4.109683	2.366200	2.934021
H	-4.850417	3.181853	2.933881
C	-3.274236	2.185885	1.817910
H	-3.365221	2.873455	0.962795
C	-0.885571	-1.921173	-1.702101
H	-0.972221	-2.563202	-0.803616
C	0.623046	-0.136410	-2.528563
H	0.993934	-0.614842	-3.462301
H	-0.335748	0.360705	-2.799364
C	1.588165	0.933970	-2.091890
C	2.638970	2.097937	-0.347985
C	3.149080	3.035795	-1.279280
H	3.797697	3.847430	-0.924265
C	2.889401	2.890729	-2.643986
C	2.114836	1.802457	-3.062302
H	1.885037	1.631902	-4.124397
C	-0.745965	-2.853783	-2.917638
H	-1.599843	-3.559064	-2.961172
H	-0.716190	-2.300201	-3.878454
H	0.182350	-3.451597	-2.822058
C	1.051275	0.356707	2.035205
C	-0.028361	-1.650291	1.271998
H	1.161575	-1.823488	-1.484784
H	2.072378	-1.307207	0.403193
C	2.961439	-2.550954	-0.266649
C	4.317491	-1.870188	-0.216141
C	2.701994	-3.546119	0.871186
C	5.193441	-2.030436	0.893244
C	4.739858	-1.122227	-1.338581
C	3.287136	-3.092570	2.211125
C	6.468287	-1.422654	0.839213
C	6.004649	-0.520843	-1.373918
H	4.045971	-1.040558	-2.189341
C	6.875016	-0.672483	-0.275717
H	7.154663	-1.545719	1.693979
H	6.318481	0.061373	-2.255185
H	7.873389	-0.206170	-0.289987
O	2.435290	-2.763233	-1.410429
H	3.308933	3.602256	-3.372114
N	2.962963	2.211799	1.010091
C	3.293100	3.537607	1.518214

H	2.578421	4.290222	1.132847
H	3.211968	3.517918	2.624771
H	4.332558	3.867804	1.267739
C	3.833696	1.168637	1.561190
H	3.544747	0.185568	1.150878
H	4.904183	1.365297	1.309566
H	3.723921	1.132906	2.663012
C	4.802805	-2.877228	2.092514
H	1.616998	-3.754636	0.935362
H	3.189416	-4.497585	0.548203
H	3.068936	-3.837670	3.004702
H	2.795249	-2.144009	2.518268
H	5.307873	-3.867950	1.998002
H	5.215582	-2.423606	3.019191

4/TSHy-fRSR-re-8ke

Frequencies, energies and thermodynamic properties:

Lowest Vibrational Mode (1/cm) =	-387.3417
E(RB-P86) (a.u.) =	-4809.16286296
Thermal correction to Enthalpy (a.u.) =	0.786244
Thermal correction to Gibbs Free Energy (a.u.) =	0.655709
Total Entropy (cal/Kmol) =	274.734
E(RPBE1PBE) (a.u.) =	-4808.47552664

Optimised cartesian coordinates (Angstrom):

Fe	-3.860402	-1.290206	-0.720076
Mn	0.766939	-0.331027	0.440580
P	-1.152314	0.858683	0.254307
O	1.313121	0.785135	3.111262
O	-0.374747	-2.545160	1.981465
N	0.406478	-1.217397	-1.424086
N	1.916917	0.960056	-0.816046
C	-2.349702	0.088757	-0.915406
C	-2.075445	-1.100195	-1.719989
C	-3.169594	-1.249073	-2.652326
H	-3.280766	-2.053669	-3.389955
C	-4.116076	-0.190236	-2.429032
H	-5.065074	-0.051331	-2.964151
C	-3.622109	0.629790	-1.357911
H	-4.118917	1.519008	-0.949350
C	-3.722561	-2.354488	1.039144
H	-2.821394	-2.444404	1.659483
C	-4.082065	-3.224970	-0.049001
H	-3.509481	-4.095886	-0.395476
C	-5.312456	-2.740403	-0.620766
H	-5.838477	-3.172790	-1.482482
C	-5.715761	-1.570978	0.117925
H	-6.603615	-0.956359	-0.082556
C	-4.732522	-1.330200	1.141728
H	-4.737632	-0.501863	1.861949
C	-0.947940	2.578354	-0.438659
C	-0.351864	3.557371	0.392763
H	-0.076339	3.301304	1.428614
C	-0.118739	4.857422	-0.084842
H	0.327982	5.612118	0.582258
C	-0.455121	5.196934	-1.409806
H	-0.270652	6.216196	-1.784669
C	-1.028594	4.226812	-2.248958
H	-1.295745	4.481245	-3.287191
C	-1.275782	2.926585	-1.767167
H	-1.746270	2.186627	-2.432804
C	-2.196778	1.228542	1.757585
C	-2.093819	0.430057	2.917135
H	-1.371024	-0.395982	2.954549
C	-2.914318	0.676871	4.033331
H	-2.817307	0.043331	4.929384
C	-3.849431	1.726420	4.008505
H	-4.489982	1.920218	4.883561
C	-3.956111	2.532726	2.860317
H	-4.680718	3.362219	2.831261
C	-3.134011	2.288778	1.746414
H	-3.218608	2.941470	0.863730
C	-0.856929	-2.007605	-1.618242
H	-0.944738	-2.612273	-0.694137
C	0.678695	-0.286730	-2.530596
H	1.033017	-0.810726	-3.446154
H	-0.272149	0.217947	-2.814739
C	1.669062	0.780729	-2.145438

C	2.762240	1.990146	-0.458910
C	3.278694	2.882015	-1.431224
H	3.946481	3.694145	-1.114932
C	3.000591	2.689492	-2.786349
C	2.200919	1.601036	-3.154173
H	1.955927	1.394193	-4.206373
C	-0.746334	-2.989852	-2.797076
H	-1.614298	-3.678981	-2.806066
H	-0.714128	-2.474985	-3.779069
H	0.170662	-3.601931	-2.684800
C	1.152250	0.388906	2.006509
C	0.033728	-1.631356	1.337824
H	1.189884	-1.940714	-1.423386
H	2.125068	-1.374742	0.439769
C	2.961346	-2.671606	-0.178253
C	4.335379	-2.061479	-0.074347
C	2.828356	-3.559590	1.086782
C	4.872338	-2.293038	1.211746
C	5.084773	-1.428667	-1.079962
C	3.854094	-2.994833	2.093695
C	6.183446	-1.882124	1.504779
C	6.396772	-1.017616	-0.783200
H	4.648041	-1.278922	-2.080596
C	6.940195	-1.243933	0.500456
H	6.622327	-2.062875	2.499966
H	7.009922	-0.526207	-1.555808
H	7.972616	-0.924999	0.717141
O	2.410802	-2.948066	-1.288929
H	4.311032	-3.767816	2.745773
H	3.371239	-2.252942	2.770502
H	1.793023	-3.627737	1.466995
H	3.133906	-4.580206	0.760228
H	3.425019	3.364423	-3.545799
N	3.103514	2.149746	0.889772
C	3.468498	3.486099	1.343201
H	2.767794	4.239718	0.934893
H	3.396716	3.510792	2.450283
H	4.512903	3.782063	1.071651
C	3.950822	1.107912	1.478132
H	3.632999	0.116746	1.111033
H	5.024398	1.266313	1.213447
H	3.847451	1.120251	2.581012

4/TSHy-fRSR-re-9ke

Frequencies, energies and thermodynamic properties:

Lowest Vibrational Mode (1/cm) =	-396.2531
E(RB-P86) (a.u.) =	-4694.89954683
Thermal correction to Enthalpy (a.u.) =	0.766585
Thermal correction to Gibbs Free Energy (a.u.) =	0.638964
Total Entropy (cal/Kmol) =	268.600
E(RPBE1PBE) (a.u.) =	-4694.21839927

Optimised cartesian coordinates (Angstrom):

Fe	-3.660908	-0.987541	-0.719588
Mn	1.055274	-0.470017	0.358188
P	-0.760761	0.880309	0.288800
O	1.772748	0.432620	3.068922
O	-0.229902	-2.664656	1.811892
N	0.579334	-1.221993	-1.535088
N	2.277903	0.791311	-0.860179
C	-2.046172	0.274263	-0.884376
C	-1.890217	-0.886965	-1.757886
C	-3.014971	-0.895239	-2.665345
H	-3.209874	-1.646426	-3.441010
C	-3.865719	0.222682	-2.359219
H	-4.812642	0.467124	-2.859082
C	-3.280195	0.939715	-1.260699
H	-3.691435	1.842690	-0.791601
C	-3.568958	-2.160305	0.973001
H	-2.662917	-2.358725	1.560218
C	-4.026111	-2.933276	-0.151481
H	-3.536262	-3.825887	-0.563378
C	-5.225884	-2.317597	-0.658592
H	-5.807175	-2.654650	-1.527338
C	-5.512115	-1.164267	0.156101
H	-6.350627	-0.468747	0.016777
C	-4.487339	-1.064778	1.162461
H	-4.406082	-0.282836	1.928461

C	-0.432024	2.611400	-0.323404
C	0.261454	3.495671	0.538188
H	0.538655	3.165997	1.552603
C	0.590403	4.794928	0.118190
H	1.112207	5.476410	0.809162
C	0.253503	5.227115	-1.179317
H	0.512886	6.245635	-1.509161
C	-0.416577	4.350284	-2.049022
H	-0.684826	4.677600	-3.066359
C	-0.759512	3.051879	-1.624408
H	-1.303547	2.386833	-2.312667
C	-1.734713	1.254403	1.837769
C	-1.670329	0.388720	2.950965
H	-1.019333	-0.495429	2.923783
C	-2.438803	0.642067	4.102193
H	-2.372958	-0.044671	4.961132
C	-3.282653	1.765178	4.159227
H	-3.882244	1.963975	5.061768
C	-3.350212	2.638063	3.057674
H	-4.003201	3.524829	3.092804
C	-2.579885	2.387268	1.908773
H	-2.631976	3.090853	1.063486
C	-0.748556	-1.894479	-1.736333
H	-0.866406	-2.538203	-0.842499
C	0.904886	-0.262627	-2.601602
H	1.197972	-0.768012	-3.548895
H	-0.006315	0.331243	-2.840180
C	1.986882	0.700960	-2.190064
C	3.211646	1.730601	-0.475395
C	3.779906	2.624836	-1.415675
H	4.519200	3.362633	-1.076978
C	3.458307	2.524550	-2.771342
C	2.562707	1.525713	-3.170601
H	2.278662	1.393376	-4.225026
C	-0.745004	-2.819634	-2.965486
H	-1.667414	-3.433718	-2.988092
H	-0.689178	-2.258892	-3.921011
H	0.120491	-3.509303	-2.904787
C	1.545132	0.121150	1.948536
C	0.239695	-1.754257	1.206561
H	1.303650	-2.009265	-1.591904
H	2.316672	-1.621920	0.262357
O	2.426822	-3.094731	-1.550541
H	3.920933	3.199923	-3.507727
N	3.594713	1.793449	0.871290
C	4.071164	3.074063	1.379505
H	3.420903	3.897698	1.026925
H	4.027350	3.048268	2.488018
H	5.128630	3.304044	1.094606
C	4.379116	0.664421	1.379434
H	3.982881	-0.283391	0.975223
H	5.454708	0.761316	1.089499
H	4.309499	0.627327	2.484542
C	3.039246	-2.918145	-0.438514
C	4.431395	-2.366789	-0.490821
C	2.800321	-3.908408	0.713174
C	5.377867	-2.657139	0.436585
H	4.677862	-1.784587	-1.395340
C	3.587508	-3.553949	1.980768
H	3.135126	-4.897922	0.320139
C	5.095527	-3.444343	1.691101
H	6.416000	-2.314482	0.272937
H	3.400501	-4.302825	2.779077
H	1.714350	-3.998557	0.908579
H	3.220550	-2.578107	2.368156
H	5.550894	-4.459623	1.587830
H	5.631703	-2.981364	2.549451

4/TSHy-fRSR-si-10ke

Frequencies, energies and thermodynamic properties:

Lowest Vibrational Mode (1/cm) =	-474.2074
E(RB-P86) (a.u.) =	-4734.18554238
Thermal correction to Enthalpy (a.u.) =	0.794909
Thermal correction to Gibbs Free Energy (a.u.) =	0.664225
Total Entropy (cal/Kmol) =	275.049
E(RPBE1PBE) (a.u.) =	-4733.50561759

Optimised cartesian coordinates (Angstrom):

Fe	-3.536938	-1.243997	-0.788103
Mn	1.085168	-0.204103	0.255252
P	-0.883741	0.905041	0.364801
O	1.756657	0.480442	3.041471
O	0.097118	-2.660214	1.507723
N	0.657627	-0.755784	-1.714968
N	2.117512	1.352978	-0.819193
C	-2.120252	0.247022	-0.832950
C	-1.838730	-0.767509	-1.846629
C	-2.987181	-0.829857	-2.721429
H	-3.105976	-1.503435	-3.579412
C	-3.973348	0.109364	-2.260091
H	-4.965007	0.271831	-2.703404
C	-3.450278	0.766918	-1.094724
H	-3.965096	1.537574	-0.506809
C	-3.231985	-2.581330	0.752491
H	-2.291099	-2.715835	1.301467
C	-3.606964	-3.274972	-0.451147
H	-3.007879	-4.033813	-0.972351
C	-4.895140	-2.782243	-0.867799
H	-5.447276	-3.094923	-1.764235
C	-5.317784	-1.784426	0.081893
H	-6.249165	-1.204211	0.035572
C	-4.289133	-1.657438	1.081304
H	-4.296804	-0.966450	1.934185
C	-0.807886	2.717109	-0.076006
C	-0.157689	3.583768	0.835702
H	0.241708	3.182283	1.781256
C	-0.025610	4.953036	0.552881
H	0.465344	5.616619	1.282787
C	-0.518567	5.477725	-0.657788
H	-0.412866	6.551382	-0.880326
C	-1.145879	4.622091	-1.578838
H	-1.534679	5.021345	-2.529366
C	-1.292779	3.251317	-1.289733
H	-1.807598	2.601307	-2.013781
C	-1.845296	0.997621	1.963754
C	-1.652556	0.031634	2.975336
H	-0.910437	-0.766739	2.841811
C	-2.409171	0.073853	4.160912
H	-2.241830	-0.687875	4.939053
C	-3.369540	1.082224	4.355028
H	-3.959510	1.115691	5.284768
C	-3.566566	2.052796	3.355798
H	-4.312126	2.851401	3.498466
C	-2.808209	2.013506	2.172654
H	-2.963519	2.791955	1.409732
C	-0.561944	-1.587675	-1.984001
H	-0.553121	-2.355828	-1.185375
C	0.790548	0.379396	-2.638888
H	1.071830	0.057126	-3.666367
H	-0.196133	0.886418	-2.735990
C	1.781228	1.396564	-2.141280
C	2.941743	2.352581	-0.343809
C	3.362572	3.416622	-1.179212
H	4.020947	4.193764	-0.769566
C	3.002912	3.442271	-2.528375
C	2.214468	2.398616	-3.025317
H	1.901990	2.362569	-4.079348
C	-0.468978	-2.327581	-3.330225
H	-1.277926	-3.080636	-3.414192
H	-0.552796	-1.643263	-4.199214
H	0.501138	-2.860582	-3.390552
C	1.540911	0.260846	1.897505
C	0.442476	-1.655406	0.976090
H	1.503689	-1.394353	-1.908358
H	2.448423	-1.262912	0.011521
O	2.848769	-2.152116	-2.061863
H	3.353577	4.252878	-3.185889
N	3.362977	2.308522	0.991861
C	3.725618	3.567448	1.631565
H	2.979392	4.350864	1.397286
H	3.731430	3.412179	2.730132
H	4.740040	3.941183	1.343127
C	4.270072	1.216617	1.348233
H	3.925323	0.286842	0.864795
H	5.317811	1.438299	1.026638

```
 H      4.258042   1.059575   2.444649
 C      3.238850  -2.332348  -0.846316
 C      2.780165  -3.579896  -0.151671
 C      4.670333  -1.906670  -0.477127
 C      3.509711  -4.266066   0.768810
 H      1.819633  -3.988922  -0.509801
 C      5.035171  -2.243359   0.972932
 H      4.815733  -0.833965  -0.716224
 C      4.842045  -3.739287   1.264058
 H      4.381082  -1.661922   1.658811
 H      5.333329  -2.470288  -1.174975
 H      6.079347  -1.936891   1.195030
 H      4.936427  -3.940758   2.354762
 H      5.657100  -4.338736   0.790161
 C      3.060178  -5.594958   1.319497
 H      2.107030  -5.935248   0.868220
 H      3.830592  -6.379157   1.144574
 H      2.924774  -5.539910   2.423036
```

```
4/TSHy-fRSR-si-11ke
Frequencies, energies and thermodynamic properties:
 Lowest Vibrational Mode (1/cm) =                         -450.6081
 E(RB-P86) (a.u.) =                                       -4773.46177551
 Thermal correction to Enthalpy (a.u.) =                   0.823178
 Thermal correction to Gibbs Free Energy (a.u.) =          0.690005
 Total Entropy (cal/Kmol) =                                280.285
 E(RPBE1PBE) (a.u.) =                                     -4772.78088065
Optimised cartesian coordinates (Angstrom):
 Fe    -3.449338  -1.834112  -0.719530
 Mn     0.947606   0.024157   0.163354
 P     -1.189308   0.731809   0.401891
 O      1.611752   0.757239   2.938967
 O      0.511120  -2.615897   1.353243
 N      0.532343  -0.545920  -1.805844
 N      1.606682   1.778779  -0.901012
 C     -2.337679  -0.104578  -0.772408
 C     -1.921030  -1.022664  -1.830807
 C     -3.077407  -1.276009  -2.659294
 H     -3.108900  -1.937852  -3.533933
 C     -4.197575  -0.548900  -2.126570
 H     -5.221659  -0.562258  -2.523262
 C     -3.751565   0.165074  -0.962315
 H     -4.372718   0.811117  -0.328867
 C     -2.826197  -3.124895   0.762667
 H     -1.851227  -3.092270   1.265731
 C     -3.123240  -3.849515  -0.444546
 H     -2.419340  -4.470706  -1.014690
 C     -4.499358  -3.598271  -0.789219
 H     -5.026316  -3.989032  -1.670047
 C     -5.054218  -2.719481   0.208763
 H     -6.078651  -2.323714   0.221163
 C     -4.020205  -2.424359   1.166214
 H     -4.115152  -1.766036   2.039495
 C     -1.475990   2.540616   0.044277
 C     -0.964695   3.478673   0.973729
 H     -0.461441   3.123771   1.887804
 C     -1.102197   4.858069   0.749032
 H     -0.715833   5.573668   1.492540
 C     -1.732302   5.326395  -0.420399
 H     -1.838393   6.408444  -0.597481
 C     -2.224761   4.403784  -1.358670
 H     -2.719117   4.758894  -2.277133
 C     -2.100199   3.019790  -1.127968
 H     -2.511733   2.312531  -1.864424
 C     -2.071033   0.580347   2.041663
 C     -1.638697  -0.354146   3.007583
 H     -0.756317  -0.978774   2.814854
 C     -2.332014  -0.502199   4.223066
 H     -1.977083  -1.235646   4.964560
 C     -3.467592   0.281564   4.492793
 H     -4.008488   0.166119   5.445531
 C     -3.903861   1.220285   3.539847
 H     -4.788716   1.844834   3.742200
 C     -3.209119   1.371661   2.327120
 H     -3.554220   2.124351   1.601400
 C     -0.522572  -1.586497  -2.047825
 H     -0.333170  -2.356387  -1.273934
```

```
 C      0.401880   0.617534  -2.695059
 H      0.689701   0.382275  -3.744031
 H     -0.667273   0.926129  -2.732712
 C      1.201164   1.793894  -2.203981
 C      2.247660   2.904601  -0.425736
 C      2.410785   4.054384  -1.237403
 H      2.927360   4.932412  -0.828221
 C      1.983417   4.048541  -2.566946
 C      1.386941   2.885941  -3.067735
 H      1.034273   2.819637  -4.107443
 C     -0.356739  -2.266084  -3.418259
 H     -1.017046  -3.153268  -3.491622
 H     -0.603887  -1.589855  -4.262103
 H      0.690897  -2.609756  -3.533607
 C      1.385915   0.526560   1.799211
 C      0.631614  -1.544988   0.853211
 H      1.467169  -1.009258  -2.057548
 H      2.470775  -0.734724  -0.165968
 O      2.930474  -1.514600  -2.303323
 H      2.136571   4.930577  -3.207982
 N      2.741818   2.902048   0.884972
 C      2.888841   4.186021   1.559393
 H      1.994693   4.816753   1.391394
 H      2.980996   3.999357   2.649227
 H      3.796308   4.758334   1.241800
 C      3.856953   1.993551   1.154700
 H      3.668308   1.029487   0.652242
 H      4.824986   2.420715   0.793160
 H      3.933830   1.804696   2.243501
 C      3.423021  -1.633089  -1.123340
 C      3.247280  -2.947639  -0.420147
 C      4.763590  -0.947196  -0.817456
 C      4.153764  -3.464508   0.442885
 H      2.364734  -3.534673  -0.724894
 C      5.277727  -1.232740   0.598316
 H      4.684479   0.139726  -1.021284
 C      5.407389  -2.746642   0.916446
 H      4.004230  -4.491624   0.827007
 H      4.566714  -0.793219   1.331499
 H      5.473348  -1.344865  -1.579031
 H      6.255041  -0.729940   0.766829
 C      6.641877  -3.367359   0.210611
 H      7.579134  -2.909667   0.594073
 H      6.698256  -4.460970   0.395117
 H      6.607312  -3.215487  -0.887854
 C      5.557037  -2.941446   2.443664
 H      5.668570  -4.015800   2.702828
 H      6.455760  -2.406794   2.818744
 H      4.673151  -2.548159   2.988370

4/TSHy-fRSR-si-12ke
Frequencies, energies and thermodynamic properties:
 Lowest Vibrational Mode (1/cm) =                         -311.5613
 E(RB-P86) (a.u.) =                                       -5119.80392858
 Thermal correction to Enthalpy (a.u.) =                   0.948017
 Thermal correction to Gibbs Free Energy (a.u.) =          0.796466
 Total Entropy (cal/Kmol) =                                318.967
 E(RPBE1PBE) (a.u.) =                                     -5119.09835608
Optimised cartesian coordinates (Angstrom):
 Fe    -2.686834  -3.082039  -1.086640
 Mn     0.077825   0.649498   0.373978
 P     -2.130269   0.148807   0.381706
 O      0.030532   1.378413   3.226685
 O      0.967253  -1.951173   1.392906
 N      0.221055   0.075334  -1.637702
 N     -0.155272   2.548131  -0.566461
 C     -2.590446  -1.033539  -0.954607
 C     -1.668903  -1.533485  -1.973355
 C     -2.457607  -2.251448  -2.949151
 H     -2.069428  -2.766554  -3.836712
 C     -3.836968  -2.218448  -2.545730
 H     -4.672997  -2.699907  -3.070611
 C     -3.923537  -1.479249  -1.317163
 H     -4.842919  -1.278094  -0.752717
 C     -1.634148  -4.009673   0.421603
 H     -0.853739  -3.537361   1.032158
 C     -1.424356  -4.688355  -0.830033
```

H	-0.458327	-4.832632	-1.332349
C	-2.706414	-5.123183	-1.322789
H	-2.889597	-5.651765	-2.267915
C	-3.709457	-4.715701	-0.372178
H	-4.791302	-4.879987	-0.466390
C	-3.048113	-4.025460	0.704445
H	-3.534306	-3.571997	1.577821
C	-3.263425	1.595053	0.060000
C	-3.417006	2.553451	1.091173
H	-2.917917	2.397765	2.061400
C	-4.210405	3.695762	0.895625
H	-4.334325	4.421152	1.715752
C	-4.846372	3.913921	-0.342097
H	-5.467312	4.810728	-0.495796
C	-4.683538	2.979185	-1.378451
H	-5.176174	3.139646	-2.350888
C	-3.899465	1.826145	-1.179247
H	-3.801386	1.094471	-1.995963
C	-2.943997	-0.576585	1.898396
C	-2.173849	-1.238526	2.878926
H	-1.082973	-1.303936	2.770885
C	-2.790104	-1.825932	3.999379
H	-2.170978	-2.336280	4.754331
C	-4.185464	-1.760156	4.157799
H	-4.666811	-2.218610	5.036284
C	-4.962498	-1.097737	3.189574
H	-6.056304	-1.034165	3.305847
C	-4.346802	-0.507156	2.072339
H	-4.969100	0.022941	1.334742
C	-0.160256	-1.328243	-2.022524
H	0.307786	-1.961527	-1.243547
C	-0.365973	1.088728	-2.529703
H	0.141417	1.127755	-3.519364
H	-1.421498	0.804010	-2.739082
C	-0.373855	2.461874	-1.910425
C	-0.220527	3.799808	0.009916
C	-0.605819	4.933703	-0.747973
H	-0.655630	5.915736	-0.259679
C	-0.849702	4.818822	-2.118272
C	-0.702319	3.562515	-2.718447
H	-0.866958	3.414662	-3.795903
C	0.448630	-1.739003	-3.374233
H	0.303585	-2.824122	-3.546795
H	-0.007355	-1.197759	-4.228375
H	1.539033	-1.541442	-3.369076
C	0.046694	1.165522	2.061239
C	0.566761	-0.918569	0.964967
H	1.277107	0.115265	-1.749622
H	1.753004	0.891057	0.244457
C	3.240095	0.679773	-0.619206
C	3.845356	-0.287970	0.388307
C	3.800023	-0.101896	1.788305
C	4.514986	-1.415916	-0.126660
C	4.436168	-1.022063	2.631506
C	5.164975	-2.358296	0.705483
H	4.516673	-1.539923	-1.220816
C	5.116825	-2.135283	2.098645
H	4.394988	-0.883632	3.724134
H	5.603992	-2.836114	2.791890
O	2.897597	0.226243	-1.759358
H	-1.121581	5.704049	-2.713979
H	3.234554	0.741432	2.213134
C	3.691901	2.135393	-0.566885
C	3.237923	3.000977	-1.590645
C	4.623025	2.638308	0.371474
C	3.673720	4.331905	-1.659326
H	2.546117	2.597596	-2.344628
C	5.070143	3.970447	0.296941
H	5.022818	1.981336	1.157351
C	4.593894	4.824680	-0.712669
H	3.302560	4.989337	-2.461982
H	5.803759	4.339052	1.031882
H	4.944271	5.867707	-0.768990
C	5.888745	-3.569122	0.073272
C	4.868358	-4.407141	-0.740787
C	7.007416	-3.059968	-0.873097
C	6.530880	-4.484494	1.137455

H	4.055401	-4.788536	-0.087964
H	4.401064	-3.813833	-1.553236
H	5.370897	-5.280535	-1.208579
H	7.758876	-2.461288	-0.316588
H	7.534077	-3.916461	-1.345609
H	6.601879	-2.424310	-1.686712
H	7.037050	-5.338556	0.641263
H	7.294772	-3.949385	1.739285
H	5.775743	-4.904535	1.834127
N	0.099190	3.938892	1.365487
C	-0.491543	5.056051	2.091849
C	1.497823	3.703886	1.736890
H	-1.565402	5.157367	1.842279
H	-0.407141	4.849806	3.178865
H	0.015909	6.034499	1.899076
H	1.564024	3.479274	2.819793
H	1.890950	2.837419	1.176962
H	2.128546	4.598379	1.513507

4/TSHy-fRSR-si-13ke

Frequencies, energies and thermodynamic properties:

Lowest Vibrational Mode (1/cm) =	-124.3540
E(RB-P86) (a.u.) =	-5375.62218745
Thermal correction to Enthalpy (a.u.) =	0.855717
Thermal correction to Gibbs Free Energy (a.u.) =	0.706571
Total Entropy (cal/Kmol) =	313.905
E(RPBE1PBE) (a.u.) =	-5374.99680524

Optimised cartesian coordinates (Angstrom):

Fe	-4.223544	-1.960068	-0.730767
Mn	-0.017289	0.201376	0.351104
P	-2.190617	0.844561	0.236017
O	0.261650	1.426036	3.016050
O	-0.487571	-2.256631	1.870802
N	-0.185026	-0.664662	-1.568568
N	0.749082	1.789123	-0.887832
C	-3.171882	-0.204793	-0.916255
C	-2.610807	-1.240342	-1.782968
C	-3.661529	-1.661629	-2.681758
H	-3.576866	-2.440110	-3.450210
C	-4.856419	-0.922255	-2.378539
H	-5.829851	-1.041154	-2.872988
C	-4.564354	-0.032192	-1.289701
H	-5.271699	0.665142	-0.822864
C	-3.727772	-2.993484	0.982917
H	-2.818427	-2.839702	1.577902
C	-3.859298	-3.896730	-0.129485
H	-3.073271	-4.555382	-0.522867
C	-5.195259	-3.768656	-0.653182
H	-5.604117	-4.307230	-1.518602
C	-5.891195	-2.786920	0.139193
H	-6.923793	-2.446995	-0.017022
C	-4.984163	-2.305340	1.148285
H	-5.201410	-1.535544	1.900018
C	-2.477272	2.567372	-0.421140
C	-2.068486	3.650532	0.394649
H	-1.646731	3.453807	1.393842
C	-2.205863	4.975098	-0.051068
H	-1.898153	5.805962	0.603938
C	-2.733451	5.241585	-1.329614
H	-2.839026	6.280547	-1.680117
C	-3.123760	4.173076	-2.154369
H	-3.537403	4.369834	-3.156498
C	-2.998627	2.844458	-1.703725
H	-3.328663	2.021766	-2.356534
C	-3.247883	0.899873	1.774230
C	-2.910038	0.129184	2.907966
H	-2.000520	-0.486554	2.905963
C	-3.732183	0.135731	4.049831
H	-3.450134	-0.470659	4.925302
C	-4.903520	0.912942	4.077273
H	-5.545024	0.918819	4.972829
C	-5.246373	1.689595	2.955344
H	-6.158277	2.307844	2.967113
C	-4.423318	1.686695	1.815615
H	-4.697208	2.314714	0.953699
C	-1.182176	-1.771897	-1.776259
H	-1.052949	-2.427283	-0.894080

```
 C     -0.261009   0.371069  -2.612848
 H      0.124276   0.012618  -3.592829
 H     -1.331189   0.627172  -2.776876
 C      0.462147   1.627240  -2.211297
 C      1.344096   2.975991  -0.511341
 C      1.549673   4.022841  -1.444971
 H      2.020015   4.957148  -1.111823
 C      1.229052   3.840411  -2.791450
 C      0.701138   2.606187  -3.189142
 H      0.442730   2.399476  -4.238024
 C     -0.860630  -2.607156  -3.027482
 H     -1.489319  -3.519492  -3.051456
 H     -1.040346  -2.049042  -3.968832
 H      0.198265  -2.932345  -3.005094
 C      0.189618   0.987569   1.918541
 C     -0.359497  -1.266548   1.227536
 H      0.769552  -1.091771  -1.651149
 H      1.495964  -0.534360   0.396969
 C      2.915194  -1.495570  -0.562922
 C      2.529089  -2.951617  -0.097519
 O      2.532428  -1.263215  -1.735791
 H      1.416961   4.640357  -3.524354
 N      1.750106   3.132979   0.815637
 C      1.872801   4.485526   1.342639
 H      1.001691   5.100030   1.043507
 H      1.890675   4.428185   2.450426
 H      2.807959   5.008421   1.020730
 C      2.810944   2.238432   1.281483
 H      2.643688   1.226920   0.872885
 H      3.816765   2.607956   0.963856
 H      2.788391   2.168665   2.386504
 F      1.314425  -3.303330  -0.583632
 F      2.525938  -3.224062   1.222656
 F      3.441909  -3.790627  -0.668294
 C      4.191660  -0.936284   0.021352
 C      4.406932  -0.811722   1.440752
 C      5.277382  -0.649850  -0.890865
 C      3.364293  -0.929772   2.422758
 C      5.734545  -0.486144   1.945710
 C      6.590379  -0.308064  -0.350941
 C      5.179237  -0.713935  -2.326200
 C      3.619431  -0.813937   3.780246
 H      2.333327  -1.076810   2.083901
 C      5.967348  -0.387112   3.360413
 C      6.785425  -0.255087   1.040745
 C      7.691648  -0.041217  -1.235150
 H      4.211223  -0.965941  -2.772702
 C      6.265806  -0.453693  -3.147389
 C      4.937501  -0.559671   4.265429
 H      2.784554  -0.901045   4.493535
 H      6.988638  -0.156095   3.703705
 H      7.787685  -0.014566   1.432438
 C      7.539518  -0.105555  -2.606448
 H      8.665321   0.213568  -0.786366
 H      6.139477  -0.516115  -4.240238
 H      5.122193  -0.478647   5.348047
 H      8.388451   0.101990  -3.276500
```

```
4/TSHy-fRSR-si-15ke
Frequencies, energies and thermodynamic properties:
 Lowest Vibrational Mode (1/cm) =                          -357.6914
 E(RB-P86) (a.u.) =                                        -4810.35453604
 Thermal correction to Enthalpy (a.u.) =                    0.808110
 Thermal correction to Gibbs Free Energy (a.u.) =           0.674072
 Total Entropy (cal/Kmol) =                                 282.106
 E(RPBE1PBE) (a.u.) =                                      -4809.66505524
Optimised cartesian coordinates (Angstrom):
 Fe    -3.479584  -1.275140  -0.993471
 Mn     1.055738  -0.038964   0.267950
 P     -0.997590   0.910556   0.403124
 O      1.601682   0.520555   3.107334
 O      0.209238  -2.650119   1.293437
 N      0.720377  -0.531386  -1.744750
 N      1.948618   1.658759  -0.684053
 C     -2.145456   0.285126  -0.895576
 C     -1.772235  -0.653009  -1.953082
 C     -2.882480  -0.720368  -2.876622
```

H	-2.934150	-1.346785	-3.775840
C	-3.932741	0.139627	-2.403680
H	-4.913510	0.279182	-2.877870
C	-3.489139	0.753294	-1.183141
H	-4.065517	1.462396	-0.575428
C	-3.150265	-2.671310	0.486034
H	-2.221985	-2.778090	1.061945
C	-3.441658	-3.321589	-0.764328
H	-2.781099	-4.016751	-1.299871
C	-4.741648	-2.882782	-1.203739
H	-5.242966	-3.179614	-2.134760
C	-5.255693	-1.962739	-0.221261
H	-6.217862	-1.435675	-0.273091
C	-4.271987	-1.829735	0.821665
H	-4.350278	-1.185528	1.707036
C	-1.040382	2.756911	0.141457
C	-0.532399	3.577516	1.177891
H	-0.176075	3.118037	2.114205
C	-0.489545	4.973519	1.031167
H	-0.109375	5.597775	1.855758
C	-0.932114	5.574364	-0.163423
H	-0.898149	6.669357	-0.278825
C	-1.417593	4.767256	-1.206081
H	-1.765347	5.226619	-2.145299
C	-1.472924	3.367795	-1.055555
H	-1.875564	2.753654	-1.875704
C	-2.009234	0.785612	1.969751
C	-1.747096	-0.226778	2.917907
H	-0.922910	-0.933861	2.756086
C	-2.537357	-0.347980	4.076031
H	-2.314487	-1.144025	4.804315
C	-3.601660	0.541204	4.305605
H	-4.218547	0.446692	5.213429
C	-3.868447	1.558255	3.370451
H	-4.695573	2.265631	3.542137
C	-3.076372	1.682298	2.215807
H	-3.288363	2.496440	1.505551
C	-0.454850	-1.406318	-2.087525
H	-0.427092	-2.211684	-1.327455
C	0.821045	0.648899	-2.620440
H	1.232157	0.395878	-3.623078
H	-0.204160	1.042461	-2.801566
C	1.638374	1.757235	-2.008718
C	2.664211	2.693994	-0.118934
C	2.969139	3.862575	-0.860831
H	3.535546	4.672244	-0.382619
C	2.617518	3.954258	-2.209322
C	1.964477	2.867805	-2.803828
H	1.679595	2.876901	-3.866254
C	-0.292773	-2.070789	-3.465599
H	-1.074251	-2.842462	-3.614860
H	-0.372475	-1.344013	-4.299804
H	0.693174	-2.573242	-3.523729
C	1.428300	0.356636	1.946629
C	0.508875	-1.585538	0.862105
H	1.571454	-1.142891	-1.911477
H	2.516640	-0.887520	0.023760
C	3.452771	-2.030364	-0.839860
C	3.333861	-3.143492	0.193482
C	4.801378	-1.281687	-0.828502
C	3.845079	-3.034757	1.506808
C	2.749494	-4.364482	-0.205669
C	3.770834	-4.119357	2.396344
C	2.680393	-5.453807	0.679050
H	2.358904	-4.439493	-1.232212
C	3.190976	-5.335187	1.984530
H	4.166583	-4.015891	3.419610
H	2.226554	-6.402209	0.348139
O	2.852908	-2.165914	-1.952279
H	2.877936	4.849540	-2.794999
N	3.097165	2.571734	1.206285
C	3.351444	3.794933	1.956906
H	3.374602	3.543701	3.037295
H	4.328190	4.279614	1.706424
H	2.538338	4.527620	1.790659
C	4.085263	1.525127	1.479358
H	5.112199	1.850523	1.180958

```
 H      4.086496   1.284844   2.560757
 H      3.809913   0.609358   0.926261
 H      3.136908  -6.186723   2.681742
 H      4.286352  -2.085627   1.849070
 H      5.038346  -0.893929   0.182464
 H      5.560402  -2.078394  -1.017939
 C      4.924110  -0.191230  -1.889929
 H      4.682778  -0.597598  -2.892311
 H      4.221719   0.642579  -1.690965
 H      5.951822   0.224826  -1.917318
```

4/TSHy-fRSR-si-16ke

```
Frequencies, energies and thermodynamic properties:
 Lowest Vibrational Mode (1/cm) =                        -311.4829
 E(RB-P86) (a.u.) =                                      -4924.60924962
 Thermal correction to Enthalpy (a.u.) =                  0.827545
 Thermal correction to Gibbs Free Energy (a.u.) =         0.689486
 Total Entropy (cal/Kmol) =                               290.569
 E(RPBE1PBE) (a.u.) =                                    -4923.90831716
Optimised cartesian coordinates (Angstrom):
 Fe    -1.987596  -3.314463  -0.870823
 Mn     0.347156   0.866843   0.037669
 P     -1.672763  -0.028476   0.534144
 O      0.769835   1.596655   2.858866
 O      1.957805  -1.480826   0.732190
 N      0.128644   0.294250  -1.972468
 N     -0.437102   2.680593  -0.772845
 C     -2.222114  -1.281089  -0.699326
 C     -1.488859  -1.620127  -1.917092
 C     -2.344727  -2.474988  -2.708688
 H     -2.089021  -2.922178  -3.677376
 C     -3.577493  -2.681930  -1.998648
 H     -4.415510  -3.308343  -2.332654
 C     -3.504899  -1.957625  -0.760306
 H     -4.289533  -1.915804   0.005870
 C     -0.469237  -4.019266   0.328842
 H      0.332476  -3.407814   0.762975
 C     -0.432090  -4.660834  -0.958894
 H      0.403616  -4.631561  -1.671012
 C     -1.693022  -5.327401  -1.162256
 H     -1.988884  -5.890123  -2.057805
 C     -2.508770  -5.100733   0.003442
 H     -3.535487  -5.461439   0.151707
 C     -1.754479  -4.290647   0.924097
 H     -2.101609  -3.925190   1.899094
 C     -3.084062   1.190283   0.537948
 C     -3.167970   2.102049   1.618128
 H     -2.446209   2.034900   2.448206
 C     -4.170755   3.084991   1.649742
 H     -4.234100   3.772672   2.508374
 C     -5.093368   3.191169   0.590737
 H     -5.878680   3.963280   0.615042
 C     -5.005187   2.304721  -0.495957
 H     -5.721348   2.378836  -1.330036
 C     -4.009148   1.309303  -0.522233
 H     -3.968885   0.610236  -1.371729
 C     -1.961252  -0.880804   2.170912
 C     -0.875181  -1.394048   2.912534
 H      0.152204  -1.264881   2.546113
 C     -1.092589  -2.079487   4.122082
 H     -0.231451  -2.470987   4.686766
 C     -2.398526  -2.261543   4.609909
 H     -2.567593  -2.796568   5.557948
 C     -3.488134  -1.748761   3.882060
 H     -4.515795  -1.879756   4.257213
 C     -3.271048  -1.060655   2.675559
 H     -4.134331  -0.650698   2.128764
 C     -0.088788  -1.158266  -2.298010
 H      0.643668  -1.691617  -1.660895
 C     -0.799500   1.183571  -2.689425
 H     -0.525473   1.305108  -3.761102
 H     -1.810476   0.717445  -2.683432
 C     -0.915900   2.539860  -2.043359
 C     -0.609858   3.905525  -0.160795
 C     -1.339367   4.942707  -0.793402
 H     -1.462430   5.904879  -0.279317
 C     -1.836152   4.771270  -2.087317
```

C	-1.596292	3.554591	-2.736402
H	-1.952980	3.366519	-3.759801
C	0.258625	-1.476127	-3.762727
H	0.264625	-2.572147	-3.927030
H	-0.465038	-1.032456	-4.476845
H	1.271453	-1.094135	-3.999299
C	0.586282	1.381460	1.708208
C	1.260110	-0.562876	0.448274
H	1.113059	0.499306	-2.315604
H	1.872514	1.400433	-0.466026
C	3.098557	1.469439	-1.659933
C	3.043229	2.979516	-1.902554
O	2.701412	0.715952	-2.602999
H	-2.378140	5.586640	-2.591031
N	-0.052661	4.117613	1.107308
C	-0.675254	5.121375	1.962223
H	-0.335701	4.951343	3.004734
H	-0.403700	6.172372	1.691105
H	-1.777446	5.021207	1.937496
C	1.409835	4.144943	1.186299
H	1.816207	5.125047	0.833700
H	1.730015	3.981223	2.233883
H	1.824492	3.327085	0.570872
H	2.073571	3.251745	-2.361560
H	3.207313	3.601979	-1.004841
H	3.845572	3.221893	-2.634799
C	4.174392	0.949671	-0.720889
C	4.703990	-0.317849	-0.962320
C	4.694789	1.716646	0.370862
C	5.745469	-0.863751	-0.155960
H	4.299875	-0.898683	-1.806186
C	5.703679	1.214606	1.180018
H	4.285081	2.715289	0.584387
C	6.299427	-2.160006	-0.400371
C	6.262106	-0.081365	0.946108
H	6.089976	1.815705	2.019480
C	7.313843	-2.664308	0.405107
H	5.905285	-2.754676	-1.240702
C	7.304929	-0.628259	1.754731
C	7.820290	-1.892852	1.490931
H	7.730555	-3.664609	0.206506
H	7.696156	-0.030207	2.594118
H	8.624349	-2.303229	2.122454

4/TSHy-fRSR-si-17ke

Frequencies, energies and thermodynamic properties:

Lowest Vibrational Mode (1/cm) =	-442.0793
E(RB-P86) (a.u.) =	-4908.32254264
Thermal correction to Enthalpy (a.u.) =	0.778870
Thermal correction to Gibbs Free Energy (a.u.) =	0.645140
Total Entropy (cal/Kmol) =	281.460
E(RPBE1PBE) (a.u.) =	-4907.66599240

Optimised cartesian coordinates (Angstrom):

Fe	-2.823466	-2.554190	-0.845783
Mn	0.837354	0.482081	0.135636
P	-1.406801	0.484956	0.449925
O	1.337491	1.166380	2.956800
O	1.257676	-2.242926	1.122845
N	0.556167	-0.057678	-1.865619
N	0.881838	2.424130	-0.790233
C	-2.286368	-0.571065	-0.777842
C	-1.646105	-1.243914	-1.906341
C	-2.698543	-1.776807	-2.741243
H	-2.557321	-2.355016	-3.662964
C	-3.968108	-1.461221	-2.144961
H	-4.952602	-1.756203	-2.532257
C	-3.721174	-0.728500	-0.934237
H	-4.487569	-0.345690	-0.248312
C	-1.790188	-3.694280	0.526049
H	-0.852916	-3.402564	1.017062
C	-1.898841	-4.390994	-0.728392
H	-1.061764	-4.730485	-1.353224
C	-3.298278	-4.544144	-1.034458
H	-3.714759	-5.014959	-1.935075
C	-4.055287	-3.943830	0.034434
H	-5.150145	-3.877440	0.090427
C	-3.124174	-3.415874	0.997343

H	-3.381266	-2.877478	1.918680
C	-2.241783	2.140034	0.238141
C	-2.038847	3.112312	1.247238
H	-1.441496	2.858944	2.138056
C	-2.600923	4.394115	1.130453
H	-2.450745	5.131746	1.935122
C	-3.355621	4.736330	-0.008496
H	-3.794840	5.742326	-0.100572
C	-3.545012	3.785241	-1.025187
H	-4.133493	4.041946	-1.920621
C	-2.994475	2.494556	-0.902650
H	-3.173634	1.755644	-1.698803
C	-2.146502	-0.048092	2.080382
C	-1.416677	-0.867686	2.968405
H	-0.390872	-1.172220	2.721958
C	-1.992118	-1.309170	4.174277
H	-1.404908	-1.946480	4.854513
C	-3.305345	-0.938068	4.512056
H	-3.754156	-1.282797	5.457218
C	-4.039950	-0.116592	3.637253
H	-5.067865	0.186152	3.893579
C	-3.464076	0.328280	2.434537
H	-4.047596	0.985301	1.771239
C	-0.150761	-1.346028	-2.178156
H	0.286206	-2.076765	-1.469034
C	0.071296	1.071527	-2.673373
H	0.410269	1.009767	-3.731396
H	-1.041148	1.037835	-2.705247
C	0.471483	2.401158	-2.091876
C	1.140456	3.659017	-0.230767
C	0.924286	4.854602	-0.959403
H	1.143352	5.820178	-0.485132
C	0.502994	4.806793	-2.290045
C	0.293551	3.553276	-2.875511
H	-0.036281	3.451763	-3.919928
C	0.162197	-1.843994	-3.599976
H	-0.213458	-2.877597	-3.737373
H	-0.299972	-1.210071	-4.384154
H	1.259615	-1.857926	-3.754866
C	1.154085	0.968873	1.803378
C	1.037771	-1.154118	0.702413
H	1.574346	-0.215246	-2.146904
H	2.506218	0.273130	-0.265695
C	3.694659	-0.185378	-1.315902
C	4.242832	-1.334493	-0.505925
C	4.669920	0.989299	-1.033405
C	5.237363	-0.872985	0.383785
C	3.954600	-2.703891	-0.624595
C	5.383471	0.635087	0.289928
H	5.396568	0.973671	-1.877141
C	5.945426	-1.777796	1.190285
C	4.658019	-3.622926	0.171602
H	3.193651	-3.050902	-1.340906
C	5.632524	-3.141315	1.064649
H	6.732743	-1.458440	1.890593
H	4.472668	-4.706087	0.112137
O	3.120080	-0.341174	-2.439824
H	4.177725	1.979758	-1.048999
H	0.363287	5.734265	-2.866752
N	1.622629	3.723047	1.084122
C	1.355947	4.937198	1.846416
H	1.513669	4.715623	2.922105
H	2.028831	5.790645	1.580575
H	0.305180	5.257296	1.710013
C	2.977178	3.213315	1.301199
H	3.747787	3.954046	0.972738
H	3.128234	2.992149	2.376025
H	3.104562	2.273532	0.736862
H	6.443274	0.962130	0.322699
H	4.879313	1.110400	1.160829
F	6.309753	-4.034795	1.825350

4/TSHy-fRSR-si-19ke
Frequencies, energies and thermodynamic properties:
Lowest Vibrational Mode (1/cm) = -430.4218
E(RB-P86) (a.u.) = -5156.65632352
Thermal correction to Enthalpy (a.u.) = 0.933179

Thermal correction to Gibbs Free Energy (a.u.) = 0.782777
Total Entropy (cal/Kmol) = 316.546
E(RPBE1PBE) (a.u.) = -5155.94875212
Optimised cartesian coordinates (Angstrom):

Fe	-3.786758	-2.642016	-0.935305
Mn	-0.188117	0.472357	0.027027
P	-2.405227	0.359379	0.478247
O	0.463502	1.010013	2.849746
O	0.407969	-2.291095	0.792248
N	-0.577339	0.057865	-1.987990
N	-0.266153	2.471345	-0.776054
C	-3.319738	-0.647176	-0.766214
C	-2.725392	-1.219875	-1.972755
C	-3.806401	-1.739778	-2.778530
H	-3.700345	-2.250616	-3.743787
C	-5.047981	-1.514612	-2.089468
H	-6.042932	-1.823111	-2.437550
C	-4.754667	-0.851742	-0.849134
H	-5.491367	-0.544839	-0.095804
C	-2.638074	-3.819042	0.306863
H	-1.690980	-3.517111	0.771987
C	-2.778198	-4.437370	-0.984990
H	-1.958898	-4.696255	-1.669203
C	-4.183055	-4.635411	-1.235299
H	-4.622619	-5.065716	-2.145171
C	-4.911721	-4.141671	-0.094556
H	-6.004313	-4.130356	0.016556
C	-3.957950	-3.634650	0.857480
H	-4.192465	-3.170622	1.824193
C	-3.312811	1.989162	0.439892
C	-3.077349	2.897262	1.500659
H	-2.415307	2.607551	2.332685
C	-3.689881	4.161065	1.511320
H	-3.511545	4.846984	2.355028
C	-4.530161	4.550778	0.450206
H	-5.008975	5.542798	0.457994
C	-4.753970	3.664811	-0.617046
H	-5.409501	3.958808	-1.452433
C	-4.151986	2.391395	-0.621984
H	-4.357755	1.702565	-1.455754
C	-3.016555	-0.317413	2.108480
C	-2.200678	-1.166445	2.887234
H	-1.182703	-1.410266	2.555374
C	-2.679455	-1.714901	4.091445
H	-2.026005	-2.373599	4.685425
C	-3.980559	-1.423067	4.536818
H	-4.353745	-1.851614	5.480575
C	-4.800325	-0.573053	3.771844
H	-5.819656	-0.331582	4.113347
C	-4.320971	-0.021496	2.570973
H	-4.970309	0.656080	1.995323
C	-1.246976	-1.239712	-2.339449
H	-0.739263	-1.992692	-1.704961
C	-1.171204	1.212423	-2.678427
H	-0.917134	1.232968	-3.761631
H	-2.280330	1.132752	-2.625048
C	-0.773700	2.516750	-2.042043
C	-0.001330	3.676507	-0.157984
C	-0.314250	4.907765	-0.785298
H	-0.088981	5.848368	-0.265901
C	-0.837915	4.929449	-2.079705
C	-1.049927	3.709281	-2.731065
H	-1.455832	3.662902	-3.752302
C	-0.999906	-1.635978	-3.805447
H	-1.334354	-2.677244	-3.985348
H	-1.537278	-0.979932	-4.520431
H	0.085739	-1.584579	-4.023717
C	0.214798	0.870603	1.700161
C	0.111427	-1.186972	0.469161
H	0.425963	-0.019781	-2.351700
H	1.457547	0.351598	-0.481644
O	1.956549	0.069926	-2.749638
H	-1.053472	5.886167	-2.580282
N	0.586364	3.672797	1.113695
C	0.353852	4.826452	1.974198
H	-0.712679	5.121790	1.946628
H	0.607126	4.541426	3.016153

```
 H      0.979627   5.715766   1.711026
 C      1.963683   3.183833   1.188550
 H      2.064015   2.288732   0.551305
 H      2.690744   3.964950   0.854752
 H      2.203695   2.893015   2.230129
 C      2.574127  -0.016538  -1.631195
 C      2.924522  -1.384248  -1.123609
 C      3.615157   1.051662  -1.271241
 C      4.053350  -1.662517  -0.429316
 H      2.255614  -2.200720  -1.441929
 C      4.307371   0.766272   0.064712
 H      3.147112   2.055820  -1.287723
 C      5.040365  -0.608154   0.070531
 H      4.294805  -2.716330  -0.205498
 H      3.532045   0.746339   0.859012
 H      4.355361   1.042051  -2.103958
 H      5.017234   1.579924   0.321890
 C      5.496204  -1.030116   1.492137
 C      5.216363  -0.275490   2.651194
 C      6.223495  -2.235456   1.648614
 C      5.643413  -0.710923   3.921734
 H      4.660405   0.670155   2.580499
 C      6.646127  -2.675159   2.912606
 H      6.473953  -2.837406   0.759795
 C      6.356298  -1.912378   4.060248
 H      5.412631  -0.099134   4.808704
 H      7.209280  -3.618067   3.002180
 H      6.687942  -2.252894   5.054048
 C      6.294316  -0.520880  -0.843074
 C      6.357334  -1.139159  -2.109471
 C      7.409053   0.240159  -0.417236
 C      7.497639  -1.004746  -2.926173
 H      5.501039  -1.732044  -2.466594
 C      8.544355   0.381832  -1.231047
 H      7.389912   0.724858   0.572571
 C      8.595120  -0.243533  -2.492012
 H      7.522657  -1.500722  -3.909944
 H      9.398324   0.981460  -0.876599
 H      9.487064  -0.137382  -3.129871

4/TSHy-fRSR-si-20ke
Frequencies, energies and thermodynamic properties:
 Lowest Vibrational Mode (1/cm) =                          -379.9908
 E(RB-P86) (a.u.) =                                        -5224.67561594
 Thermal correction to Enthalpy (a.u.) =                    0.887817
 Thermal correction to Gibbs Free Energy (a.u.) =           0.740575
 Total Entropy (cal/Kmol) =                                 309.897
 E(RPBE1PBE) (a.u.) =                                      -5223.98644098
Optimised cartesian coordinates (Angstrom):
 Fe    -4.260770  -1.906338  -0.924988
 Mn     0.005586   0.097162   0.338865
 P     -2.168543   0.739366   0.297579
 O      0.272111   1.033987   3.117858
 O     -0.487509  -2.492569   1.622344
 N     -0.164751  -0.683459  -1.605529
 N      0.714260   1.769526  -0.767421
 C     -3.158090  -0.173705  -0.958317
 C     -2.611295  -1.155957  -1.891989
 C     -3.656166  -1.475519  -2.838494
 H     -3.578941  -2.194786  -3.663361
 C     -4.834498  -0.725486  -2.498679
 H     -5.801166  -0.775106  -3.017609
 C     -4.537349   0.069702  -1.340064
 H     -5.232471   0.752491  -0.835200
 C     -3.810411  -3.077083   0.709407
 H     -2.904867  -2.989820   1.323678
 C     -3.950115  -3.890454  -0.469589
 H     -3.175124  -4.536070  -0.904220
 C     -5.276629  -3.691077  -0.995019
 H     -5.687871  -4.152988  -1.902608
 C     -5.958586  -2.755724  -0.137166
 H     -6.981231  -2.380384  -0.276955
 C     -5.052661  -2.374210   0.914833
 H     -5.260948  -1.658877   1.720922
 C     -2.439906   2.520056  -0.184625
 C     -2.133819   3.520954   0.768937
 H     -1.801975   3.231989   1.779248
```

C	-2.261733	4.881709	0.444902
H	-2.039373	5.647014	1.205795
C	-2.675251	5.267900	-0.844874
H	-2.774407	6.335209	-1.098731
C	-2.962065	4.282231	-1.804312
H	-3.286620	4.572720	-2.816378
C	-2.847558	2.917273	-1.476889
H	-3.098128	2.159301	-2.235037
C	-3.228230	0.644656	1.832311
C	-2.882387	-0.220186	2.893029
H	-1.964099	-0.819873	2.838919
C	-3.707288	-0.328955	4.027889
H	-3.418369	-1.007967	4.845875
C	-4.889998	0.425244	4.120800
H	-5.534053	0.340757	5.010534
C	-5.241385	1.295236	3.072174
H	-6.162483	1.896418	3.136068
C	-4.415526	1.407395	1.940268
H	-4.697287	2.106926	1.138157
C	-1.203398	-1.735343	-1.887388
H	-1.114494	-2.439553	-1.037074
C	-0.154686	0.391311	-2.612070
H	0.322763	0.074351	-3.566035
H	-1.208594	0.641915	-2.868624
C	0.506516	1.650828	-2.111742
C	1.257539	2.951956	-0.307188
C	1.506349	4.031763	-1.190936
H	1.938606	4.960673	-0.796519
C	1.271169	3.895911	-2.560719
C	0.784596	2.671975	-3.034788
H	0.590367	2.503439	-4.104233
C	-0.885385	-2.523643	-3.169904
H	-1.553878	-3.403471	-3.255522
H	-1.013577	-1.912526	-4.086697
H	0.157802	-2.895897	-3.134098
C	0.210776	0.725772	1.976162
C	-0.328162	-1.444445	1.086499
H	0.758682	-1.207190	-1.662193
H	1.604886	-0.504747	0.222265
H	1.490832	4.725199	-3.250870
N	1.556047	3.084370	1.056276
C	1.551065	4.431117	1.617355
H	1.481445	4.348373	2.721555
H	2.475531	5.016896	1.385474
H	0.670761	4.997258	1.256996
C	2.658539	2.272537	1.581659
H	3.650138	2.697422	1.291069
H	2.600279	2.233136	2.686133
H	2.574551	1.236459	1.207605
C	4.507709	-1.641874	1.353668
C	5.857974	-0.722244	-0.476617
C	2.773391	-1.352578	-0.598381
C	3.532763	-0.125859	-1.162712
H	3.572532	0.687714	-0.414170
H	2.973093	0.231305	-2.047229
O	2.142840	-2.074222	-1.434400
C	3.334414	-2.034014	0.647890
C	2.641839	-3.182206	1.099560
C	4.901500	-2.351582	2.511691
C	3.031240	-3.878581	2.248729
H	1.763256	-3.498659	0.520235
C	4.161746	-3.448553	2.970863
H	5.812685	-2.034121	3.037857
H	2.455795	-4.755773	2.584490
H	4.485022	-3.986002	3.876584
C	4.944936	-0.503496	-1.536129
C	5.393136	-0.674190	-2.861205
C	7.195623	-1.081862	-0.735699
C	6.729996	-1.023045	-3.125562
H	4.688273	-0.518083	-3.693704
C	7.633263	-1.221376	-2.064980
H	7.883108	-1.258086	0.106145
H	7.068687	-1.142538	-4.166840
H	8.678192	-1.501839	-2.270535
N	5.368087	-0.596667	0.871601
C	5.727371	0.473086	1.720012
O	5.315055	0.556688	2.882162

```
 N      6.516589   1.434476   1.140234
 H      6.875954   2.164641   1.754793
 H      6.948397   1.317026   0.222989

4/TSHy-fRSR-si-22ke
Frequencies, energies and thermodynamic properties:
 Lowest Vibrational Mode (1/cm) =                        -286.7892
 E(RB-P86) (a.u.) =                                      -4807.93552152
 Thermal correction to Enthalpy (a.u.) =                  0.762831
 Thermal correction to Gibbs Free Energy (a.u.) =         0.632624
 Total Entropy (cal/Kmol) =                               274.044
 E(RPBE1PBE) (a.u.) =                                    -4807.24241802
Optimised cartesian coordinates (Angstrom):
 Fe    -3.575766  -1.530619  -0.793567
 Mn     0.970342  -0.058388   0.140129
 P     -1.112719   0.773055   0.438274
 O      1.659805   0.519345   2.945332
 O      0.390152  -2.739705   1.170229
 N      0.512400  -0.572670  -1.838388
 N      1.742738   1.654702  -0.861520
 C     -2.324394   0.096784  -0.770811
 C     -1.984844  -0.803742  -1.870343
 C     -3.157658  -0.918080  -2.707591
 H     -3.245600  -1.534308  -3.611024
 C     -4.212072  -0.122754  -2.139858
 H     -5.232618  -0.030307  -2.534966
 C     -3.709002   0.496607  -0.945536
 H     -4.272335   1.165709  -0.282677
 C     -3.051300  -2.932515   0.622133
 H     -2.073755  -3.000836   1.116751
 C     -3.413372  -3.575952  -0.613132
 H     -2.765367  -4.225271  -1.217145
 C     -4.766794  -3.199685  -0.932600
 H     -5.328837  -3.506555  -1.824853
 C     -5.242859  -2.325458   0.108905
 H     -6.232087  -1.850033   0.149212
 C     -4.182719  -2.157567   1.068439
 H     -4.219530  -1.534493   1.971395
 C     -1.249338   2.612461   0.171014
 C     -0.711008   3.468110   1.162320
 H     -0.278092   3.038261   2.079975
 C     -0.734056   4.862315   0.993694
 H     -0.330354   5.514511   1.784740
 C     -1.272722   5.426022  -0.179258
 H     -1.289023   6.519446  -0.311696
 C     -1.789849   4.584046  -1.178270
 H     -2.213376   5.014109  -2.100023
 C     -1.780961   3.186531  -1.004564
 H     -2.211261   2.544004  -1.788062
 C     -1.988477   0.599141   2.077016
 C     -1.620669  -0.419861   2.982161
 H     -0.789570  -1.096842   2.742870
 C     -2.313280  -0.584855   4.195769
 H     -2.009759  -1.384813   4.889709
 C     -3.382968   0.266469   4.523862
 H     -3.923240   0.137671   5.475216
 C     -3.753979   1.289315   3.631768
 H     -4.586534   1.966618   3.880770
 C     -3.060004   1.457185   2.420832
 H     -3.352584   2.274253   1.743293
 C     -0.648034  -1.493361  -2.105888
 H     -0.529941  -2.300362  -1.356612
 C      0.520811   0.607979  -2.718392
 H      0.845722   0.361572  -3.753665
 H     -0.521692   0.988632  -2.806972
 C      1.373867   1.724159  -2.173782
 C      2.461276   2.712836  -0.343898
 C      2.728319   3.864493  -1.124433
 H      3.301901   4.690626  -0.683947
 C      2.329791   3.921917  -2.461863
 C      1.660585   2.818953  -3.005425
 H      1.331417   2.803688  -4.054875
 C     -0.556691  -2.144900  -3.496539
 H     -1.316053  -2.946211  -3.594224
 H     -0.724984  -1.419417  -4.318536
 H      0.440792  -2.608976  -3.627890
 C      1.430915   0.355007   1.795737
```

```
 C      0.573731  -1.646046   0.745751
 H      1.368068  -1.164106  -2.068824
 H      2.456665  -0.850731  -0.151083
 C      3.387344  -1.793527  -1.221756
 C      3.935612  -2.656995  -0.098815
 C      4.435866  -0.723124  -1.413056
 C      5.197734  -2.121339   0.282516
 C      3.445469  -3.832653   0.472264
 C      5.475045  -0.938264  -0.555411
 C      5.957108  -2.747695   1.284900
 C      4.214251  -4.465597   1.476437
 H      2.476027  -4.246583   0.155348
 C      5.450197  -3.923867   1.881293
 H      6.934309  -2.340981   1.592336
 H      3.844586  -5.390997   1.946619
 O      2.604333  -2.201530  -2.130843
 H      2.562506   4.804544  -3.077598
 N      2.924945   2.639958   0.976771
 C      3.119229   3.895067   1.693548
 H      3.166586   3.673145   2.779778
 H      4.066686   4.424460   1.421509
 H      2.266548   4.578521   1.517216
 C      3.993885   1.673745   1.248677
 H      4.988866   2.081208   0.942217
 H      4.021666   1.442626   2.331687
 H      3.804739   0.736261   0.697174
 H      6.033994  -4.429306   2.667409
 H      4.348730   0.066662  -2.171875
 H      6.390104  -0.332050  -0.475953

4/TSHy-fRSR-si-24ke
Frequencies, energies and thermodynamic properties:
 Lowest Vibrational Mode (1/cm) =                        -331.9352
 E(RB-P86) (a.u.) =                                      -4848.40471597
 Thermal correction to Enthalpy (a.u.) =                  0.814225
 Thermal correction to Gibbs Free Energy (a.u.) =         0.679370
 Total Entropy (cal/Kmol) =                               283.826
 E(RPBE1PBE) (a.u.) =                                    -4847.71267485
Optimised cartesian coordinates (Angstrom):
 Fe    -3.467368  -1.521574  -1.058414
 Mn     0.922969   0.038858   0.351175
 P     -1.191408   0.851731   0.381519
 O      1.303245   0.699058   3.196037
 O      0.248314  -2.612939   1.401045
 N      0.697977  -0.515190  -1.660639
 N      1.768174   1.756694  -0.605470
 C     -2.245903   0.126694  -0.944406
 C     -1.770243  -0.804402  -1.966228
 C     -2.836299  -0.964525  -2.929394
 H     -2.810779  -1.611027  -3.815487
 C     -3.959194  -0.168113  -2.515193
 H     -4.927982  -0.104648  -3.028679
 C     -3.605337   0.497934  -1.292635
 H     -4.250797   1.178258  -0.722599
 C     -3.098892  -2.863563   0.460921
 H     -2.187764  -2.894660   1.072291
 C     -3.297023  -3.555514  -0.785357
 H     -2.569878  -4.212000  -1.282035
 C     -4.606695  -3.216808  -1.281000
 H     -5.050517  -3.564931  -2.223365
 C     -5.220057  -2.317217  -0.337395
 H     -6.213865  -1.860024  -0.435207
 C     -4.288214  -2.096493   0.737748
 H     -4.445078  -1.444005   1.606369
 C     -1.336904   2.685285   0.069899
 C     -0.924652   3.561952   1.102864
 H     -0.586855   3.149469   2.067433
 C     -0.953090   4.953586   0.916089
 H     -0.647090   5.621033   1.737719
 C     -1.372156   5.495098  -0.314880
 H     -1.393241   6.586623  -0.461854
 C     -1.763822   4.633113  -1.353070
 H     -2.093300   5.045734  -2.320173
 C     -1.748501   3.237541  -1.162573
 H     -2.079704   2.579140  -1.980462
 C     -2.255056   0.695635   1.909829
 C     -1.971111  -0.284038   2.885742
```

```
 H     -1.100595  -0.942943   2.767788
 C     -2.797820  -0.433379   4.014687
 H     -2.557540  -1.202834   4.765658
 C     -3.920854   0.394621   4.186710
 H     -4.566353   0.278257   5.071823
 C     -4.210231   1.378610   3.223368
 H     -5.083909   2.037890   3.350042
 C     -3.382101   1.530788   2.097688
 H     -3.613583   2.318728   1.364318
 C     -0.402242  -1.471354  -2.030449
 H     -0.355198  -2.256392  -1.250267
 C      0.759215   0.653103  -2.553918
 H      1.194915   0.403995  -3.547215
 H     -0.279623   0.998948  -2.754763
 C      1.517641   1.803448  -1.945217
 C      2.409926   2.839254  -0.040906
 C      2.706166   3.995063  -0.805705
 H      3.214100   4.842149  -0.326413
 C      2.420378   4.030518  -2.172395
 C      1.839143   2.901007  -2.760596
 H      1.604337   2.866029  -3.834664
 C     -0.140742  -2.151815  -3.385369
 H     -0.860265  -2.979491  -3.545376
 H     -0.238799  -1.450230  -4.238946
 H      0.879495  -2.584251  -3.396706
 C      1.195111   0.497074   2.033328
 C      0.473522  -1.534374   0.957855
 H      1.597527  -1.066019  -1.783733
 H      2.441514  -0.702865   0.188433
 C      3.535668  -1.700682  -0.662198
 C      3.540825  -2.809984   0.380708
 C      4.022521  -2.632891   1.697854
 C      3.098412  -4.091897  -0.010756
 C      4.062130  -3.711095   2.597060
 C      3.144028  -5.174271   0.884130
 H      2.727236  -4.218874  -1.039164
 C      3.626865  -4.988091   2.192274
 H      4.433729  -3.554418   3.622547
 H      2.801936  -6.170205   0.558697
 O      2.984300  -1.925265  -1.787694
 H      2.674834   4.916852  -2.774116
 N      2.770524   2.783759   1.311033
 C      2.911373   4.043363   2.030585
 H      2.884437   3.829024   3.118920
 H      3.873006   4.575195   1.819929
 H      2.070090   4.722280   1.791766
 C      3.801805   1.808619   1.672773
 H      4.823435   2.194176   1.432628
 H      3.747871   1.590838   2.757636
 H      3.623640   0.868814   1.121528
 H      3.662791  -5.834257   2.897073
 H      4.348847  -1.637577   2.037574
 C      4.730251  -0.765666  -0.627642
 C      5.783707  -0.985585  -1.717479
 C      4.913063   0.244821  -1.735863
 H      5.125853  -0.504705   0.364753
 H      5.565754  -1.771731  -2.458347
 H      6.842531  -0.890670  -1.427201
 H      5.358447   1.218527  -1.477240
 H      4.113551   0.274426  -2.492778

4/TSHy-fRSR-si-26ke
Frequencies, energies and thermodynamic properties:
 Lowest Vibrational Mode (1/cm) =                          -465.2192
 E(RB-P86) (a.u.) =                                        -4773.45899479
 Thermal correction to Enthalpy (a.u.) =                    0.823093
 Thermal correction to Gibbs Free Energy (a.u.) =           0.691881
 Total Entropy (cal/Kmol) =                                 276.158
 E(RPBE1PBE) (a.u.) =                                      -4772.78259575
Optimised cartesian coordinates (Angstrom):
 Fe    -3.662485  -1.392708  -0.762734
 Mn     0.931191  -0.230675   0.437741
 P     -1.056150   0.853381   0.275143
 O      1.430427   0.845464   3.132912
 O     -0.201416  -2.477164   1.944084
 N      0.612579  -1.142001  -1.420298
 N      1.949180   1.167670  -0.814915
```

C	-2.204509	0.045930	-0.919721
C	-1.871513	-1.120692	-1.734166
C	-2.945768	-1.301816	-2.684054
H	-3.014349	-2.101258	-3.432334
C	-3.936277	-0.283702	-2.463338
H	-4.881947	-0.175332	-3.011245
C	-3.490303	0.542361	-1.376259
H	-4.027312	1.407800	-0.967910
C	-3.506528	-2.471071	0.986569
H	-2.609579	-2.530898	1.616648
C	-3.817663	-3.342138	-0.115996
H	-3.207013	-4.185950	-0.464516
C	-5.058344	-2.899019	-0.698998
H	-5.556018	-3.341490	-1.572362
C	-5.516378	-1.754742	0.047238
H	-6.424798	-1.172716	-0.158393
C	-4.556968	-1.488352	1.087241
H	-4.603421	-0.668915	1.816222
C	-0.960246	2.596047	-0.384368
C	-0.517103	3.620277	0.486525
H	-0.288436	3.384257	1.538289
C	-0.381375	4.941488	0.028778
H	-0.056559	5.730084	0.726472
C	-0.663156	5.259334	-1.313901
H	-0.556815	6.295482	-1.672404
C	-1.083287	4.245684	-2.191780
H	-1.306680	4.482519	-3.244377
C	-1.232686	2.923489	-1.730799
H	-1.585696	2.147887	-2.427677
C	-2.127772	1.132556	1.780314
C	-1.961151	0.347457	2.941526
H	-1.172166	-0.414497	2.984626
C	-2.803148	0.524960	4.055270
H	-2.654610	-0.097221	4.952228
C	-3.824588	1.490071	4.026718
H	-4.482272	1.629049	4.899484
C	-3.995887	2.283257	2.877048
H	-4.788447	3.047934	2.844474
C	-3.152388	2.109265	1.766374
H	-3.290311	2.752643	0.883855
C	-0.618737	-1.979053	-1.625267
H	-0.690622	-2.591031	-0.704834
C	0.863046	-0.216997	-2.536706
H	1.311928	-0.733101	-3.415070
H	-0.110506	0.192686	-2.888930
C	1.733462	0.949762	-2.144576
C	2.680069	2.284038	-0.463701
C	3.118808	3.208896	-1.442880
H	3.697652	4.087984	-1.130708
C	2.880137	2.971622	-2.798418
C	2.194652	1.806031	-3.158433
H	1.982785	1.562792	-4.210086
C	-0.463485	-2.951248	-2.807876
H	-1.305711	-3.671539	-2.826007
H	-0.445011	-2.429985	-3.786897
H	0.474413	-3.530716	-2.696500
C	1.283577	0.473844	2.017470
C	0.231986	-1.567797	1.313792
H	1.424271	-1.844378	-1.406575
H	2.393724	-1.195138	0.411714
O	2.643143	-2.814168	-1.227427
H	3.246321	3.674539	-3.562761
N	2.986088	2.502220	0.887787
C	3.203900	3.879389	1.315655
H	2.430578	4.545533	0.887615
H	3.121842	3.918670	2.421570
H	4.213682	4.277954	1.043975
C	3.946131	1.577009	1.495739
H	3.741525	0.550118	1.147025
H	4.994560	1.853053	1.222883
H	3.844855	1.602409	2.598488
C	3.391642	-2.220283	-0.370060
C	4.355061	-1.194464	-0.895981
C	3.952932	-3.066158	0.824042
C	5.528043	-0.878393	-0.296618
C	4.804476	-2.169188	1.752218
C	5.954511	-1.447747	1.029677

```
 H      6.818697  -2.135190   0.863814
 H      6.367864  -0.636177   1.670082
 H      4.091483  -0.763641  -1.877149
 H      6.224804  -0.186065  -0.803585
 H      4.128394  -1.413455   2.208888
 H      5.202327  -2.779004   2.593062
 C      2.832285  -3.745954   1.622223
 H      2.275791  -3.017558   2.241755
 H      2.111440  -4.232837   0.935228
 H      3.251956  -4.522293   2.296932
 C      4.826859  -4.171881   0.171091
 H      5.305773  -4.795156   0.956298
 H      4.195815  -4.828039  -0.461356
 H      5.626792  -3.753981  -0.473791
```

```
4/TSHy-fRSR-si-27ke
Frequencies, energies and thermodynamic properties:
 Lowest Vibrational Mode (1/cm) =                          -494.9072
 E(RB-P86) (a.u.) =                                        -4773.46635864
 Thermal correction to Enthalpy (a.u.) =                    0.823316
 Thermal correction to Gibbs Free Energy (a.u.) =           0.689365
 Total Entropy (cal/Kmol) =                                 281.924
 E(RPBE1PBE) (a.u.) =                                      -4772.78841663
Optimised cartesian coordinates (Angstrom):
 Fe    -3.430627  -1.581158  -0.752465
 Mn     1.044116  -0.032536   0.268687
 P     -1.036610   0.856817   0.358921
 O      1.653347   0.784172   3.033004
 O      0.350496  -2.525799   1.636782
 N      0.693378  -0.685132  -1.684296
 N      1.864122   1.634154  -0.853018
 C     -2.185158   0.052220  -0.837475
 C     -1.792123  -0.951661  -1.824111
 C     -2.924332  -1.161527  -2.697448
 H     -2.966601  -1.866258  -3.537377
 C     -4.007441  -0.322570  -2.261865
 H     -5.009162  -0.279196  -2.710125
 C     -3.562211   0.418909  -1.114979
 H     -4.159530   1.145860  -0.550199
 C     -2.980481  -2.823625   0.829472
 H     -2.032243  -2.830624   1.382179
 C     -3.270121  -3.594870  -0.350460
 H     -2.586584  -4.296767  -0.846952
 C     -4.604873  -3.266631  -0.782053
 H     -5.115029  -3.669745  -1.667190
 C     -5.141682  -2.293221   0.134743
 H     -6.133157  -1.825200   0.070029
 C     -4.137581  -2.017010   1.129062
 H     -4.226639  -1.303520   1.958479
 C     -1.174081   2.659878  -0.102844
 C     -0.706424   3.619782   0.826860
 H     -0.319811   3.291203   1.805072
 C     -0.743830   4.990314   0.521798
 H     -0.397004   5.725019   1.266163
 C     -1.225619   5.425843  -0.727915
 H     -1.253480   6.500721  -0.967264
 C     -1.672359   4.479660  -1.665660
 H     -2.052017   4.808752  -2.646258
 C     -1.649839   3.106084  -1.355247
 H     -2.027886   2.381937  -2.093229
 C     -2.011456   0.856798   1.953360
 C     -1.692240  -0.040808   2.995153
 H     -0.843467  -0.729114   2.890651
 C     -2.457814  -0.071836   4.175611
 H     -2.190428  -0.778650   4.977251
 C     -3.553943   0.793827   4.334310
 H     -4.151351   0.769891   5.259577
 C     -3.877824   1.696890   3.305092
 H     -4.730502   2.385182   3.419732
 C     -3.110833   1.731246   2.127662
 H     -3.367961   2.458332   1.342090
 C     -0.437270  -1.638937  -1.937696
 H     -0.360159  -2.382018  -1.118910
 C      0.724464   0.416602  -2.656577
 H      1.089224   0.085484  -3.654481
 H     -0.313702   0.786143  -2.817201
 C      1.549769   1.580244  -2.179929
```

```
 C      2.505233   2.772331  -0.405424
 C      2.773452   3.857077  -1.275892
 H      3.289352   4.743980  -0.885447
 C      2.449692   3.774550  -2.631845
 C      1.843689   2.603105  -3.097444
 H      1.561318   2.479278  -4.153287
 C     -0.266614  -2.395482  -3.266684
 H     -0.991301  -3.231547  -3.330951
 H     -0.425085  -1.742949  -4.149437
 H      0.754322  -2.823358  -3.321612
 C      1.454070   0.520132   1.895494
 C      0.575388  -1.518720   1.047529
 H      1.602326  -1.243185  -1.846119
 H      2.508198  -0.990411   0.055659
 O      3.008175  -1.881400  -1.975030
 H      2.685932   4.604287  -3.316002
 N      2.896303   2.849403   0.938721
 C      3.034659   4.174400   1.531280
 H      2.176497   4.815492   1.252997
 H      3.041271   4.063606   2.635257
 H      3.981633   4.696756   1.244167
 C      3.972144   1.943910   1.342140
 H      3.802169   0.956292   0.883874
 H      4.971227   2.333227   1.024358
 H      3.969426   1.818626   2.442643
 C      3.474056  -1.904549  -0.771307
 C      3.355047  -3.223292  -0.019203
 C      4.766534  -1.120043  -0.494802
 C      4.288000  -3.610689   0.907147
 C      5.222238  -1.239752   0.959730
 H      4.631985  -0.068261  -0.819289
 C      5.428500  -2.708002   1.337148
 H      4.442370  -0.810514   1.624869
 H      5.538156  -1.557996  -1.171334
 H      6.150026  -0.655029   1.136315
 H      5.583069  -2.812093   2.435187
 H      6.371150  -3.098030   0.880115
 C      2.256691  -4.132643  -0.519343
 H      1.345956  -3.553562  -0.761765
 H      2.557809  -4.641487  -1.461557
 H      1.975411  -4.909639   0.215566
 C      4.329094  -4.980387   1.543586
 H      4.194637  -4.903280   2.646293
 H      3.569941  -5.682641   1.153373
 H      5.330003  -5.443805   1.393418

4/TSHy-fRSR-si-5ke
Frequencies, energies and thermodynamic properties:
 Lowest Vibrational Mode (1/cm) =                          -456.2953
 E(RB-P86) (a.u.) =                                        -4884.32074913
 Thermal correction to Enthalpy (a.u.) =                    0.792118
 Thermal correction to Gibbs Free Energy (a.u.) =           0.659223
 Total Entropy (cal/Kmol) =                                 279.701
 E(RPBE1PBE) (a.u.) =                                      -4883.64536142
Optimised cartesian coordinates (Angstrom):
 Fe    -3.017497  -2.237593  -0.783797
 Mn     0.939125   0.372144   0.233745
 P     -1.298626   0.698139   0.378359
 O      1.375496   1.234368   3.016496
 O      0.918937  -2.276863   1.479945
 N      0.722454  -0.288232  -1.742025
 N      1.299364   2.216732  -0.839504
 C     -2.227037  -0.340317  -0.828191
 C     -1.612000  -1.183064  -1.852171
 C     -2.671798  -1.646991  -2.718678
 H     -2.551344  -2.314805  -3.580806
 C     -3.922951  -1.122614  -2.243238
 H     -4.911637  -1.321843  -2.678005
 C     -3.657212  -0.326801  -1.077382
 H     -4.407365   0.207513  -0.480741
 C     -2.242710  -3.374069   0.752151
 H     -1.313499  -3.159507   1.295349
 C     -2.349761  -4.157593  -0.450148
 H     -1.520849  -4.651009  -0.975323
 C     -3.731284  -4.162539  -0.858510
 H     -4.139033  -4.654108  -1.752002
 C     -4.479353  -3.383254   0.094988
```

H	-5.557439	-3.177479	0.054660
C	-3.559871	-2.893497	1.088849
H	-3.811010	-2.250355	1.942249
C	-1.896360	2.420502	-0.020179
C	-1.643828	3.441582	0.927746
H	-1.151295	3.192987	1.881726
C	-2.027722	4.768117	0.671786
H	-1.842468	5.546004	1.429887
C	-2.649357	5.103886	-0.546624
H	-2.949302	6.144594	-0.747572
C	-2.885388	4.101710	-1.502684
H	-3.371143	4.352608	-2.459292
C	-2.514154	2.768385	-1.241323
H	-2.729726	1.993169	-1.992660
C	-2.207411	0.402026	1.983600
C	-1.651519	-0.422029	2.986018
H	-0.660064	-0.871756	2.844042
C	-2.359139	-0.683795	4.173864
H	-1.906419	-1.328038	4.944363
C	-3.633047	-0.125890	4.379181
H	-4.185449	-0.330189	5.310147
C	-4.194331	0.701997	3.389580
H	-5.189176	1.150569	3.541260
C	-3.485940	0.967399	2.204794
H	-3.933247	1.633085	1.450541
C	-0.129167	-1.495990	-2.013065
H	0.164570	-2.220553	-1.227800
C	0.438766	0.820129	-2.666255
H	0.832954	0.627309	-3.688668
H	-0.664433	0.920083	-2.776821
C	0.967526	2.134427	-2.160702
C	1.669084	3.457256	-0.360504
C	1.642447	4.604858	-1.190228
H	1.946383	5.574844	-0.775565
C	1.301524	4.494929	-2.539979
C	0.978285	3.229299	-3.040767
H	0.702044	3.078738	-4.094730
C	0.193146	-2.132159	-3.377026
H	-0.286521	-3.127560	-3.461709
H	-0.160175	-1.514052	-4.227443
H	1.288414	-2.272235	-3.477208
C	1.223605	0.959855	1.874296
C	0.873812	-1.216687	0.947929
H	1.728978	-0.585382	-1.936507
H	2.572631	-0.148174	-0.000465
C	3.759764	-0.859037	-0.918153
C	3.908539	-2.182527	-0.197717
C	4.874292	0.121820	-0.526166
C	4.860916	-2.343072	0.848297
C	3.223253	-3.328483	-0.656500
C	5.226443	0.011005	0.952381
C	5.085763	-3.617859	1.417851
C	3.436067	-4.592160	-0.091143
H	2.522078	-3.200752	-1.495404
C	4.374259	-4.731633	0.953784
H	5.831529	-3.706392	2.223007
H	2.884152	-5.468996	-0.463954
H	4.556319	-5.718817	1.408230
O	3.296534	-0.848588	-2.106623
H	1.310009	5.380648	-3.193951
N	2.078488	3.574860	0.975095
C	1.922056	4.871887	1.622791
H	1.992820	4.724848	2.720210
H	2.708037	5.614393	1.335194
H	0.927448	5.300844	1.394904
C	3.346767	2.929542	1.313324
H	4.219142	3.544849	0.980619
H	3.412919	2.775609	2.408255
H	3.383822	1.941530	0.824766
H	6.078095	0.664579	1.223683
H	4.354366	0.292348	1.583158
H	4.597553	1.160484	-0.792689
H	5.761618	-0.143029	-1.146583
O	5.630274	-1.322630	1.322572

4/TSHy-fRSR-si-6ke
Frequencies, energies and thermodynamic properties:

```
 Lowest Vibrational Mode (1/cm) =                                      -335.9989
 E(RB-P86) (a.u.) =                                                    -4771.07323967
 Thermal correction to Enthalpy (a.u.) =                                0.779295
 Thermal correction to Gibbs Free Energy (a.u.) =                       0.647870
 Total Entropy (cal/Kmol) =                                             276.607
 E(RPBE1PBE) (a.u.) =                                                  -4770.38386034
Optimised cartesian coordinates (Angstrom):
 Fe    -3.493428  -1.442820  -0.814717
 Mn     1.071656  -0.054668   0.166303
 P     -0.999815   0.823753   0.427226
 O      1.722241   0.518164   2.980948
 O      0.457950  -2.728352   1.198261
 N      0.619721  -0.560851  -1.822435
 N      1.888151   1.641999  -0.827621
 C     -2.214820   0.164635  -0.789389
 C     -1.881028  -0.747701  -1.880757
 C     -3.048376  -0.847650  -2.727583
 H     -3.138682  -1.467362  -3.628422
 C     -4.094541  -0.032002  -2.173257
 H     -5.110060   0.074929  -2.577629
 C     -3.591128   0.585731  -0.978076
 H     -4.148968   1.267157  -0.323106
 C     -3.003130  -2.844594   0.612967
 H     -2.030906  -2.924851   1.116295
 C     -3.364183  -3.489102  -0.622081
 H     -2.720952  -4.151618  -1.216813
 C     -4.708816  -3.093769  -0.955620
 H     -5.267699  -3.396986  -1.851120
 C     -5.180564  -2.206810   0.077102
 H     -6.162721  -1.716131   0.106202
 C     -4.126459  -2.050052   1.045151
 H     -4.161545  -1.421637   1.944424
 C     -1.102114   2.665285   0.150026
 C     -0.540482   3.512568   1.135763
 H     -0.112791   3.075755   2.052663
 C     -0.532055   4.906319   0.962244
 H     -0.109767   5.551856   1.749085
 C     -1.062190   5.478144  -0.210783
 H     -1.053618   6.571164  -0.347437
 C     -1.602324   4.644450  -1.204508
 H     -2.019178   5.080675  -2.126464
 C     -1.625039   3.247572  -1.025341
 H     -2.072458   2.612247  -1.805188
 C     -1.900921   0.676628   2.055900
 C     -1.568635  -0.350760   2.965374
 H     -0.752895  -1.048864   2.733429
 C     -2.277221  -0.497183   4.172098
 H     -2.001462  -1.304294   4.869405
 C     -3.327888   0.381677   4.489259
 H     -3.880695   0.267590   5.435308
 C     -3.663651   1.412870   3.592819
 H     -4.481160   2.111391   3.833115
 C     -2.953496   1.561841   2.388658
 H     -3.218449   2.385238   1.707368
 C     -0.553311  -1.459828  -2.101420
 H     -0.458081  -2.268623  -1.350509
 C      0.652373   0.625102  -2.693238
 H      0.968738   0.380458  -3.731712
 H     -0.381308   1.030669  -2.775595
 C      1.531844   1.718415  -2.143539
 C      2.634511   2.680313  -0.309105
 C      2.942011   3.820859  -1.091462
 H      3.537872   4.630578  -0.650222
 C      2.552951   3.887122  -2.431184
 C      1.855193   2.802593  -2.975784
 H      1.532785   2.793719  -4.027430
 C     -0.458509  -2.111952  -3.491952
 H     -1.227556  -2.902923  -3.598305
 H     -0.609338  -1.383028  -4.314382
 H      0.534081  -2.588592  -3.615917
 C      1.510334   0.350905   1.827214
 C      0.651580  -1.637590   0.770504
 H      1.469452  -1.161497  -2.044441
 H      2.543549  -0.830048  -0.169715
 C      3.505774  -1.770154  -1.187030
 C      3.911474  -2.741032  -0.087794
 C      4.511468  -0.683955  -1.576628
```

```
 C      4.875459  -2.436762   0.901324
 C      3.330637  -4.028920  -0.089993
 C      5.240109  -3.392526   1.865381
 C      3.695555  -4.986210   0.869624
 H      2.590160  -4.255929  -0.871640
 C      4.651862  -4.671108   1.853610
 H      5.991485  -3.137510   2.629963
 H      3.233263  -5.986573   0.850740
 O      2.726014  -2.189373  -2.101391
 H      2.815341   4.760905  -3.047567
 N      3.086866   2.600743   1.015723
 C      3.328726   3.852941   1.723094
 H      3.362404   3.638564   2.811265
 H      4.296875   4.343123   1.450695
 H      2.504156   4.567779   1.537328
 C      4.107334   1.590228   1.305837
 H      5.118525   1.926358   0.966981
 H      4.141990   1.398473   2.396110
 H      3.841482   0.643564   0.802793
 H      4.940239  -5.420368   2.608233
 H      4.013310   0.078163  -2.206044
 H      5.348711  -1.443576   0.929158
 H      5.008475  -0.181584  -0.727674
 H      5.299672  -1.170625  -2.193326

4/TSHy-fRSR-si-7ke
Frequencies, energies and thermodynamic properties:
 Lowest Vibrational Mode (1/cm) =                           -419.7067
 E(RB-P86) (a.u.) =                                         -4848.44226053
 Thermal correction to Enthalpy (a.u.) =                     0.815592
 Thermal correction to Gibbs Free Energy (a.u.) =            0.682779
 Total Entropy (cal/Kmol) =                                  279.527
 E(RPBE1PBE) (a.u.) =                                       -4847.75405127
Optimised cartesian coordinates (Angstrom):
 Fe    -3.406028  -1.723447  -0.803481
 Mn     0.971678   0.142475   0.186771
 P     -1.176121   0.830551   0.397215
 O      1.610650   0.813823   2.981974
 O      0.530875  -2.518892   1.328450
 N      0.604430  -0.435970  -1.790394
 N      1.614806   1.914616  -0.846944
 C     -2.295441   0.006179  -0.812610
 C     -1.854247  -0.899697  -1.871333
 C     -2.992418  -1.144013  -2.728125
 H     -3.005107  -1.796303  -3.610281
 C     -4.124212  -0.422650  -2.212936
 H     -5.139022  -0.431512  -2.632880
 C     -3.704418   0.278613  -1.031705
 H     -4.339305   0.918843  -0.406119
 C     -2.808032  -3.031362   0.673147
 H     -1.841428  -3.003680   1.192517
 C     -3.084913  -3.741786  -0.547337
 H     -2.371513  -4.356172  -1.113002
 C     -4.455143  -3.486410  -0.911788
 H     -4.967338  -3.866578  -1.805854
 C     -5.026663  -2.619590   0.087223
 H     -6.051282  -2.224097   0.087427
 C     -4.008871  -2.336050   1.065291
 H     -4.118665  -1.688529   1.944867
 C     -1.474376   2.640239   0.054525
 C     -1.045986   3.577079   1.025744
 H     -0.599517   3.221683   1.968476
 C     -1.196457   4.956228   0.806630
 H     -0.877768   5.670453   1.582855
 C     -1.754682   5.426083  -0.398053
 H     -1.870968   6.507847  -0.570358
 C     -2.163253   4.504988  -1.377257
 H     -2.601375   4.861023  -2.323479
 C     -2.027308   3.121348  -1.152437
 H     -2.375234   2.414936  -1.921759
 C     -2.087327   0.647012   2.017371
 C     -1.658316  -0.292114   2.980192
 H     -0.762713  -0.900481   2.796504
 C     -2.372036  -0.466206   4.180416
 H     -2.019439  -1.203064   4.919618
 C     -3.524947   0.296024   4.437726
 H     -4.081823   0.160414   5.378525
```

C	-3.958406	1.239125	3.487724
H	-4.857002	1.846852	3.680515
C	-3.243611	1.416203	2.290302
H	-3.587453	2.171905	1.567221
C	-0.453502	-1.467380	-2.063873
H	-0.280236	-2.246465	-1.295576
C	0.516660	0.721584	-2.692781
H	0.868155	0.482542	-3.721080
H	-0.552597	1.015274	-2.794651
C	1.268419	1.915188	-2.167668
C	2.199233	3.060903	-0.347596
C	2.373719	4.209397	-1.157758
H	2.845823	5.102710	-0.728327
C	2.013572	4.186544	-2.507016
C	1.469346	3.007533	-3.027801
H	1.167097	2.927994	-4.082398
C	-0.270073	-2.130336	-3.440377
H	-0.932130	-3.014392	-3.532483
H	-0.506797	-1.442896	-4.278048
H	0.776713	-2.476948	-3.552361
C	1.386695	0.616539	1.835152
C	0.660050	-1.442003	0.846112
H	1.535093	-0.916519	-2.003692
H	2.509419	-0.570305	-0.105778
C	3.560375	-1.420682	-1.080037
C	3.601463	-2.655014	-0.206430
C	4.770034	-0.464107	-0.945373
C	4.279581	-2.627121	1.041733
C	3.012796	-3.853560	-0.659698
C	5.664081	-0.675610	0.298893
C	4.346966	-3.808096	1.807970
C	3.084725	-5.022987	0.113059
H	2.507770	-3.845210	-1.638176
C	3.752428	-4.999292	1.352670
H	4.878831	-3.794027	2.774222
H	2.624854	-5.956328	-0.250172
H	3.817694	-5.913749	1.964269
O	2.993069	-1.483985	-2.221940
H	2.176578	5.068591	-3.145533
N	2.625304	3.082041	0.989299
C	2.678300	4.376459	1.659657
H	2.723210	4.201205	2.754369
H	3.573832	4.988010	1.383946
H	1.765915	4.963364	1.440292
C	3.784175	2.245927	1.306843
H	4.735578	2.724344	0.965252
H	3.839515	2.081271	2.400793
H	3.669551	1.263493	0.818123
C	4.915967	-1.330080	1.479412
H	5.606693	-1.505171	2.330318
H	4.123526	-0.641423	1.851070
H	6.517356	-1.335435	0.030546
H	6.111153	0.289401	0.617926
H	4.399290	0.577466	-1.019775
H	5.359468	-0.631546	-1.873283

4/TSHy-fRSR-si-8ke

Frequencies, energies and thermodynamic properties:

Lowest Vibrational Mode (1/cm) =	-431.8929
E(RB-P86) (a.u.) =	-4809.15893389
Thermal correction to Enthalpy (a.u.) =	0.786166
Thermal correction to Gibbs Free Energy (a.u.) =	0.654949
Total Entropy (cal/Kmol) =	276.170
E(RPBE1PBE) (a.u.) =	-4808.47166755

Optimised cartesian coordinates (Angstrom):

Fe	-3.340718	-1.744644	-0.818791
Mn	1.031875	0.111430	0.210640
P	-1.115169	0.809103	0.394900
O	1.590342	0.818654	3.015316
O	0.568007	-2.537261	1.371230
N	0.674522	-0.454441	-1.772608
N	1.706382	1.877098	-0.817083
C	-2.228599	-0.015318	-0.820151
C	-1.780546	-0.922154	-1.874969
C	-2.911679	-1.166043	-2.740621
H	-2.917532	-1.818595	-3.622706
C	-4.046996	-0.443919	-2.233992

H	-5.058669	-0.452461	-2.661439
C	-3.635975	0.257365	-1.049463
H	-4.275414	0.897504	-0.428386
C	-2.756137	-3.054519	0.661855
H	-1.793554	-3.029130	1.188795
C	-3.024991	-3.763799	-0.561023
H	-2.308499	-4.379301	-1.121552
C	-4.391939	-3.505744	-0.935822
H	-4.897954	-3.884499	-1.833993
C	-4.969529	-2.638469	0.059277
H	-5.993412	-2.241162	0.051891
C	-3.958677	-2.357102	1.045104
H	-4.073957	-1.709805	1.924120
C	-1.396715	2.620092	0.045245
C	-0.932389	3.552746	1.004125
H	-0.464844	3.192660	1.934865
C	-1.071711	4.933228	0.786632
H	-0.724194	5.644666	1.552965
C	-1.654752	5.408115	-0.404338
H	-1.762136	6.490986	-0.575471
C	-2.098785	4.490900	-1.371579
H	-2.555862	4.851079	-2.307212
C	-1.973625	3.105828	-1.148392
H	-2.348173	2.402679	-1.908124
C	-2.043524	0.639644	2.006939
C	-1.637708	-0.303789	2.975678
H	-0.748591	-0.924743	2.803371
C	-2.366494	-0.465810	4.168452
H	-2.031809	-1.206045	4.912600
C	-3.511539	0.312552	4.412368
H	-4.080137	0.186148	5.347420
C	-3.921799	1.259920	3.456439
H	-4.813919	1.880311	3.638677
C	-3.191788	1.425260	2.266504
H	-3.517378	2.184412	1.538578
C	-0.378187	-1.489051	-2.052699
H	-0.213127	-2.263564	-1.277909
C	0.583475	0.711872	-2.663550
H	0.914807	0.478927	-3.699905
H	-0.483723	1.019120	-2.744131
C	1.359592	1.889370	-2.137394
C	2.315495	3.008347	-0.313366
C	2.515388	4.155517	-1.120267
H	3.008255	5.036500	-0.688709
C	2.153610	4.144943	-2.469076
C	1.582615	2.980151	-2.993889
H	1.278119	2.911055	-4.048557
C	-0.176721	-2.160381	-3.422278
H	-0.843453	-3.040210	-3.521140
H	-0.393208	-1.476016	-4.267939
H	0.869637	-2.515047	-3.511216
C	1.409797	0.600093	1.865145
C	0.705693	-1.465945	0.877747
H	1.607246	-0.926239	-1.990467
H	2.569674	-0.621182	-0.083655
C	3.606236	-1.476043	-1.049465
C	3.727058	-2.697139	-0.168194
C	4.883080	-0.648450	-0.744752
C	4.768824	-2.519170	0.768044
C	3.034414	-3.914882	-0.264331
C	5.378058	-1.134176	0.635415
H	5.611818	-0.936598	-1.536245
C	5.114372	-3.564415	1.640934
C	3.384369	-4.962352	0.606464
H	2.240912	-4.042473	-1.017781
C	4.414376	-4.785076	1.555334
H	5.929559	-3.441526	2.373133
H	2.858360	-5.928822	0.546670
H	4.681673	-5.614868	2.229595
O	3.065802	-1.499870	-2.200306
H	4.727536	0.443290	-0.831238
H	2.335990	5.025276	-3.104737
N	2.738063	3.016739	1.023609
C	2.828220	4.306758	1.697471
H	2.861979	4.127225	2.791898
H	3.743712	4.890683	1.427745
H	1.935849	4.922907	1.475486

C	3.858097	2.134833	1.354380
H	4.833261	2.578485	1.033856
H	3.887550	1.958145	2.447519
H	3.713010	1.162002	0.853994
H	6.483839	-1.144144	0.729373
H	4.998338	-0.479870	1.452058

4/TSHy-fRSR-si-9ke

Frequencies, energies and thermodynamic properties:

Lowest Vibrational Mode (1/cm) =	-456.7293
E(RB-P86) (a.u.) =	-4694.89614434
Thermal correction to Enthalpy (a.u.) =	0.766745
Thermal correction to Gibbs Free Energy (a.u.) =	0.640414
Total Entropy (cal/Kmol) =	265.887
E(RPBE1PBE) (a.u.) =	-4694.21697938

Optimised cartesian coordinates (Angstrom):

Fe	-3.608819	-0.969062	-0.739369
Mn	1.092104	-0.438114	0.334310
P	-0.726562	0.909589	0.310976
O	1.807827	0.379872	3.073014
O	-0.205254	-2.654484	1.741568
N	0.626478	-1.091509	-1.598030
N	2.319722	0.891418	-0.835636
C	-2.013732	0.322708	-0.870838
C	-1.848875	-0.801594	-1.790164
C	-2.982541	-0.792917	-2.686177
H	-3.173087	-1.516500	-3.488715
C	-3.847471	0.298690	-2.329285
H	-4.803223	0.546910	-2.810111
C	-3.262326	0.981912	-1.209128
H	-3.683538	1.858793	-0.700957
C	-3.494982	-2.190212	0.917844
H	-2.585916	-2.387940	1.500181
C	-3.934566	-2.938212	-0.230181
H	-3.425952	-3.807876	-0.667654
C	-5.145692	-2.331910	-0.721315
H	-5.718768	-2.654307	-1.601024
C	-5.456340	-1.209264	0.126821
H	-6.308080	-0.526498	0.005900
C	-4.435250	-1.119370	1.137921
H	-4.370323	-0.359003	1.926914
C	-0.415852	2.661320	-0.251612
C	0.308928	3.509023	0.620943
H	0.624479	3.135422	1.608622
C	0.621285	4.825650	0.245268
H	1.168634	5.477878	0.944639
C	0.236292	5.312277	-1.019280
H	0.483152	6.344415	-1.314578
C	-0.465127	4.472291	-1.900439
H	-0.771002	4.842274	-2.892208
C	-0.791835	3.156166	-1.519313
H	-1.361651	2.520572	-2.214445
C	-1.697628	1.236163	1.873104
C	-1.632580	0.339553	2.961669
H	-0.983293	-0.544559	2.910144
C	-2.399284	0.561475	4.120505
H	-2.332544	-0.149043	4.959807
C	-3.242341	1.683012	4.210240
H	-3.840476	1.856879	5.118870
C	-3.310770	2.586250	3.133624
H	-3.963012	3.472183	3.194256
C	-2.541947	2.367216	1.977247
H	-2.594401	3.094518	1.152329
C	-0.685183	-1.784394	-1.828395
H	-0.785150	-2.478017	-0.970064
C	0.913954	-0.061315	-2.606857
H	1.174692	-0.500092	-3.595822
H	-0.002746	0.547972	-2.774186
C	2.009656	0.870288	-2.164446
C	3.251524	1.819835	-0.418703
C	3.806729	2.759589	-1.321971
H	4.547423	3.483193	-0.957202
C	3.470326	2.722667	-2.676924
C	2.570690	1.742880	-3.111584
H	2.271347	1.661470	-4.166869
C	-0.667168	-2.640094	-3.107103
H	-1.565342	-3.288019	-3.151008

```
 H     -0.648021  -2.025800  -4.030508
 H      0.226445  -3.296122  -3.098538
 C      1.579410   0.098049   1.945339
 C      0.267321  -1.740661   1.147266
 H      1.384238  -1.842108  -1.719030
 H      2.319991  -1.656096   0.215597
 O      2.645315  -2.773276  -1.786465
 H      3.923289   3.433174  -3.385706
 N      3.648469   1.826342   0.924775
 C      4.150145   3.077277   1.480258
 H      3.506327   3.923865   1.173194
 H      4.121494   3.004098   2.586988
 H      5.206078   3.305629   1.189122
 C      4.412220   0.663472   1.379209
 H      3.963883  -0.252077   0.957131
 H      5.483240   0.733942   1.065825
 H      4.366678   0.591334   2.483627
 C      2.997139  -2.902776  -0.557804
 C      2.370748  -4.015668   0.233728
 C      4.465684  -2.629031  -0.188633
 C      3.018694  -4.689691   1.214647
 H      1.368655  -4.332469  -0.100250
 C      4.777501  -2.884691   1.292027
 H      4.747242  -1.606629  -0.511987
 C      4.392158  -4.313917   1.709507
 H      2.535673  -5.573436   1.670364
 H      4.198479  -2.168734   1.915109
 H      5.059313  -3.326137  -0.825875
 H      5.850815  -2.692558   1.502559
 H      4.437238  -4.427023   2.815420
 H      5.133547  -5.051775   1.316547

4/TSHy-fRSS-re-8ke
Frequencies, energies and thermodynamic properties:
 Lowest Vibrational Mode (1/cm) =                        -396.3743
 E(RB-P86) (a.u.) =                                      -4809.15610475
 Thermal correction to Enthalpy (a.u.) =                  0.786396
 Thermal correction to Gibbs Free Energy (a.u.) =         0.655523
 Total Entropy (cal/Kmol) =                               275.446
 E(RPBE1PBE) (a.u.) =                                    -4808.46761122
Optimised cartesian coordinates (Angstrom):
 Fe    -3.597383  -1.398526  -1.069335
 Mn     0.934298   0.590826  -0.285560
 P     -1.240718   0.980849   0.282360
 N      0.357789  -0.999407  -1.551166
 N      1.464246  -1.058540   0.961869
 C     -2.423940   0.312248  -0.967344
 C     -2.058346  -0.478986  -2.133585
 C     -3.246920  -0.641097  -2.938094
 H     -3.305699  -1.201771  -3.881188
 C     -4.334297   0.050668  -2.300958
 H     -5.369804   0.100225  -2.663211
 C     -3.838853   0.619711  -1.078080
 H     -4.428862   1.200967  -0.358490
 C     -3.243673  -2.746867   0.465594
 H     -2.412463  -2.736110   1.182400
 C     -3.270771  -3.426419  -0.804456
 H     -2.460372  -4.026615  -1.238660
 C     -4.553640  -3.181550  -1.409003
 H     -4.885778  -3.547047  -2.389849
 C     -5.319307  -2.354434  -0.509819
 H     -6.334214  -1.975921  -0.690582
 C     -4.507143  -2.081624   0.647433
 H     -4.790123  -1.463992   1.509532
 C     -1.635461   2.801710   0.240184
 C     -1.360376   3.600610   1.374547
 H     -0.984025   3.134061   2.298044
 C     -1.566406   4.990557   1.338576
 H     -1.352293   5.596006   2.233973
 C     -2.043934   5.606116   0.166769
 H     -2.207882   6.695178   0.139722
 C     -2.308712   4.821557  -0.969719
 H     -2.680291   5.293513  -1.893493
 C     -2.103945   3.429996  -0.934991
 H     -2.315425   2.830324  -1.833266
 C     -2.034350   0.510142   1.913113
 C     -1.422114  -0.469352   2.723164
```

```
 H     -0.477041  -0.924733   2.398073
 C     -2.003688  -0.863951   3.943078
 H     -1.508806  -1.630839   4.559973
 C     -3.205465  -0.276529   4.374190
 H     -3.660370  -0.580537   5.330360
 C     -3.819555   0.710947   3.580636
 H     -4.756826   1.184474   3.914425
 C     -3.237622   1.103862   2.362910
 H     -3.721655   1.893376   1.767665
 C     -0.688323  -0.918153  -2.618207
 H     -0.813587  -1.952977  -3.018619
 C      0.127200  -2.199388  -0.731201
 H     -0.936268  -2.176386  -0.405753
 H      0.250192  -3.132932  -1.327330
 C      0.989231  -2.249856   0.494642
 C      2.147655  -1.071280   2.161479
 C      2.284523  -2.264400   2.910978
 H      2.832045  -2.238115   3.862730
 C      1.785488  -3.473716   2.418471
 C      1.144102  -3.470477   1.173885
 H      0.734026  -4.391288   0.734005
 C     -0.214996  -0.051374  -3.799932
 H     -0.878740  -0.217723  -4.671305
 H     -0.230065   1.026950  -3.557256
 H      0.819340  -0.337310  -4.079294
 C      0.856753   1.864112  -1.476697
 O      0.925760   2.774809  -2.234381
 C      1.496858   1.876605   0.784878
 O      1.823092   2.844942   1.383839
 H      2.467062   0.330748  -1.006000
 H      1.274819  -1.150124  -2.070424
 C      3.440080  -0.358687  -2.154152
 C      4.593675  -0.695359  -1.244839
 C      3.850932   0.973311  -2.836292
 C      5.476181   0.404286  -1.153401
 C      4.883689  -1.922432  -0.625332
 C      4.920358   1.596382  -1.912579
 H      4.309836   0.674804  -3.806582
 C      6.673614   0.280045  -0.429164
 C      6.081619  -2.042407   0.102007
 H      4.187512  -2.770191  -0.730032
 H      5.704270   2.162089  -2.457540
 C      6.969330  -0.948316   0.196886
 H      7.379136   1.124188  -0.356384
 H      6.337486  -2.995947   0.591481
 H      7.909498  -1.059074   0.761115
 H      2.994615   1.634498  -3.060901
 H      4.453147   2.311224  -1.196694
 O      2.736335  -1.248549  -2.727465
 H      1.916601  -4.407293   2.987224
 N      2.681086   0.129837   2.651615
 C      2.753555   0.291863   4.099288
 H      3.618430  -0.239981   4.569757
 H      2.870599   1.372562   4.323803
 H      1.818291  -0.059507   4.576828
 C      3.876650   0.633561   1.968849
 H      3.997110   1.715185   2.175523
 H      4.793381   0.093511   2.309134
 H      3.767178   0.498167   0.878937

4/TSHy-fRSS-si-8ke
Frequencies, energies and thermodynamic properties:
 Lowest Vibrational Mode (1/cm) =                          -252.9984
 E(RB-P86) (a.u.) =                                        -4809.15864316
 Thermal correction to Enthalpy (a.u.) =                    0.786885
 Thermal correction to Gibbs Free Energy (a.u.) =           0.656368
 Total Entropy (cal/Kmol) =                                 274.698
 E(RPBE1PBE) (a.u.) =                                      -4808.46981133
Optimised cartesian coordinates (Angstrom):
 Fe     3.635816  -0.443065  -1.605371
 Mn    -1.054975  -0.042996   0.129606
 P      1.033000  -0.532959   0.915326
 N     -0.333203   0.126874  -1.855959
 N     -1.037220   2.110460  -0.040303
 C      2.108658  -1.220640  -0.419348
 C      1.761793  -1.317083  -1.830453
 C      2.798491  -2.087931  -2.478414
```

H	2.837538	-2.329876	-3.549522
C	3.772746	-2.474124	-1.493966
H	4.686597	-3.053434	-1.681991
C	3.366616	-1.920494	-0.231770
H	3.905081	-2.024962	0.718988
C	3.859256	1.616400	-1.493740
H	3.169467	2.344762	-1.050405
C	3.872580	1.179833	-2.865311
H	3.182388	1.501986	-3.656270
C	4.953431	0.241122	-3.017516
H	5.221715	-0.291198	-3.939873
C	5.605877	0.100552	-1.739181
H	6.455504	-0.559911	-1.520167
C	4.925222	0.947465	-0.794877
H	5.164205	1.057866	0.270822
C	0.955009	-1.931449	2.141564
C	0.575747	-1.646391	3.474750
H	0.412495	-0.603349	3.789223
C	0.406745	-2.681486	4.409542
H	0.118775	-2.438952	5.445055
C	0.602677	-4.021498	4.025148
H	0.470179	-4.833500	4.757732
C	0.964747	-4.315619	2.699232
H	1.115252	-5.361305	2.386053
C	1.138594	-3.279089	1.762774
H	1.419303	-3.527244	0.728232
C	2.211904	0.635289	1.786611
C	2.132957	2.018162	1.511771
H	1.352543	2.391153	0.831391
C	3.028502	2.925004	2.107234
H	2.948461	4.000183	1.880833
C	4.016969	2.460631	2.993219
H	4.716874	3.169176	3.464012
C	4.101134	1.085747	3.281148
H	4.868967	0.712984	3.978019
C	3.204634	0.180284	2.685587
H	3.278270	-0.890307	2.931937
C	0.501021	-0.893054	-2.564566
H	0.837592	-0.420555	-3.518188
C	0.250452	1.474171	-1.999042
H	1.274268	1.447401	-1.565673
H	0.360057	1.759056	-3.069563
C	-0.577282	2.493398	-1.270895
C	-1.704570	3.053034	0.714708
C	-2.009803	4.332923	0.173801
H	-2.542950	5.067338	0.790838
C	-1.585587	4.678062	-1.109353
C	-0.835151	3.746168	-1.843698
H	-0.458793	3.973760	-2.851247
C	-0.348587	-2.114634	-2.964321
H	0.209084	-2.731955	-3.695844
H	-0.597349	-2.751129	-2.095712
H	-1.293613	-1.775397	-3.435299
C	-1.403144	-1.751985	0.040723
O	-1.734080	-2.889778	0.054427
C	-1.950096	-0.150184	1.638989
O	-2.621893	-0.360982	2.592155
H	-2.536010	0.105371	-0.594782
H	-1.257849	0.147797	-2.369793
C	-3.576113	0.242929	-1.908869
C	-4.458675	-0.894776	-1.462662
C	-4.266753	1.516429	-1.362933
C	-5.387017	-0.430777	-0.502983
C	-4.484201	-2.222036	-1.921556
C	-5.141131	1.033643	-0.186708
H	-3.553617	2.323573	-1.114183
C	-6.354339	-1.306863	0.016283
C	-5.456116	-3.096118	-1.402462
H	-3.758811	-2.562237	-2.677369
H	-4.577959	1.114642	0.770779
C	-6.382447	-2.640765	-0.439197
H	-7.087858	-0.958981	0.762343
H	-5.501365	-4.140499	-1.751034
H	-7.141419	-3.335405	-0.044144
H	-4.907759	1.886975	-2.196258
H	-6.080701	1.610337	-0.060558
O	-2.841731	0.225474	-2.937691

```
 H     -1.813037   5.673171  -1.521996
 N     -2.087284   2.738704   2.016778
 C     -1.054436   2.328167   2.969825
 H     -0.543353   3.216415   3.413806
 H     -1.513271   1.734982   3.784818
 H     -0.304450   1.696480   2.465731
 C     -3.151019   3.519600   2.636997
 H     -3.534886   2.953929   3.510040
 H     -2.811620   4.518460   3.007158
 H     -3.989961   3.669296   1.930444
```

```
4/TSHy-mRSR-re-8ke
Frequencies, energies and thermodynamic properties:
 Lowest Vibrational Mode (1/cm) =                        -418.5687
 E(RB-P86) (a.u.) =                                      -4809.14483614
 Thermal correction to Enthalpy (a.u.) =                  0.786407
 Thermal correction to Gibbs Free Energy (a.u.) =         0.657393
 Total Entropy (cal/Kmol) =                               271.532
 E(RPBE1PBE) (a.u.) =                                    -4808.46107048
Optimised cartesian coordinates (Angstrom):
 Fe    -3.094156  -2.113255  -0.786420
 Mn     0.751325   0.894897  -0.363473
 P     -1.428803   0.922039   0.352095
 O      1.404184   2.019378   2.274707
 N      0.251771  -0.392164  -1.986843
 N      2.653974   0.752062  -1.216343
 C     -2.603768  -0.102801  -0.622192
 C     -2.283746  -0.615712  -1.943390
 C     -3.505844  -1.127348  -2.522573
 H     -3.594574  -1.586160  -3.517258
 C     -4.571817  -0.948426  -1.574712
 H     -5.615371  -1.263673  -1.708754
 C     -4.018686  -0.340374  -0.395267
 H     -4.575927  -0.095555   0.517122
 C     -1.714557  -3.581824  -0.337977
 H     -0.626271  -3.475358  -0.472339
 C     -2.646419  -4.040980  -1.334767
 H     -2.393682  -4.373458  -2.350596
 C     -3.973914  -3.963395  -0.779630
 H     -4.907830  -4.220217  -1.297387
 C     -3.864181  -3.457480   0.565165
 H     -4.698637  -3.265297   1.252719
 C     -2.471265  -3.223072   0.836600
 H     -2.066026  -2.823659   1.775300
 C     -2.196927   2.621425   0.181120
 C     -1.725613   3.652246   1.028678
 H     -0.961425   3.429957   1.790991
 C     -2.222253   4.960269   0.908911
 H     -1.847706   5.749298   1.580806
 C     -3.193013   5.263762  -0.065373
 H     -3.581694   6.290217  -0.160010
 C     -3.662034   4.247891  -0.915111
 H     -4.422442   4.473592  -1.680059
 C     -3.168872   2.933717  -0.792713
 H     -3.555057   2.148129  -1.459551
 C     -1.940830   0.583943   2.119921
 C     -1.057888  -0.070678   2.999279
 H     -0.059066  -0.345231   2.637521
 C     -1.431342  -0.349760   4.327235
 H     -0.722233  -0.860020   4.998556
 C     -2.699982   0.031716   4.795966
 H     -2.995889  -0.183282   5.835158
 C     -3.584561   0.707204   3.933859
 H     -4.574753   1.026334   4.296771
 C     -3.205664   0.989362   2.610146
 H     -3.897270   1.548635   1.961260
 C     -1.037581  -0.374042  -2.764550
 H     -0.976127  -1.204821  -3.507396
 C      1.376710  -0.413680  -2.950059
 H      1.482094  -1.417588  -3.416955
 H      1.151879   0.291254  -3.777147
 C      2.689329   0.024126  -2.367487
 C      3.847623   1.257427  -0.746819
 C      5.065730   0.996401  -1.414300
 H      5.992146   1.432146  -1.014892
 C      5.087731   0.235717  -2.586282
 C      3.872556  -0.260573  -3.070517
```

```
 H      3.822319  -0.863346  -3.989472
 C     -1.251735   0.936977  -3.552583
 H     -2.178878   0.846462  -4.151667
 H     -1.365732   1.798789  -2.867653
 H     -0.429013   1.161616  -4.259840
 C      1.207446   1.585685   1.189169
 H      1.251074  -0.589777   0.420658
 H      0.318676  -1.318488  -1.489582
 C      0.509399   2.455940  -1.155998
 O      0.437785   3.520027  -1.671239
 C      1.709490  -2.234579   0.333586
 C      3.180086  -2.114588   0.653521
 C      1.060349  -2.583854   1.699000
 C      3.369688  -2.044818   2.052123
 C      4.272692  -2.234797  -0.219334
 C      2.032675  -2.037495   2.766379
 H      0.020285  -2.226965   1.789176
 C      4.667222  -2.043586   2.588711
 C      5.572490  -2.241494   0.318243
 H      4.101245  -2.342812  -1.300762
 H      2.043348  -2.629927   3.704191
 C      5.767800  -2.136015   1.711830
 H      4.826608  -1.995426   3.678508
 H      6.443826  -2.341652  -0.348642
 H      6.791056  -2.148485   2.120930
 H      1.769764  -0.992886   3.050195
 H      1.038519  -3.697045   1.745342
 O      1.279205  -2.641653  -0.787082
 H      6.032720   0.045588  -3.118162
 N      3.842660   2.046761   0.417793
 C      3.442906   3.447211   0.254839
 C      4.973779   1.893956   1.325537
 H      2.607760   3.537337  -0.460432
 H      3.106100   3.855337   1.229829
 H      4.294249   4.072429  -0.115165
 H      4.680279   2.287490   2.321669
 H      5.232047   0.823272   1.440916
 H      5.889104   2.454602   1.005968
```

```
4/TSHy-mRSR-si-8ke
Frequencies, energies and thermodynamic properties:
 Lowest Vibrational Mode (1/cm) =                           -486.1131
 E(RB-P86) (a.u.) =                                         -4809.14378928
 Thermal correction to Enthalpy (a.u.) =                     0.786487
 Thermal correction to Gibbs Free Energy (a.u.) =            0.656122
 Total Entropy (cal/Kmol) =                                  274.377
 E(RPBE1PBE) (a.u.) =                                       -4808.46040994
Optimised cartesian coordinates (Angstrom):
 Fe     3.215130  -0.816490  -1.635188
 Mn    -1.324506  -0.226032   0.041387
 P      0.648331  -0.979774   0.882248
 O     -1.922875   0.772263   2.736392
 N     -0.579345  -0.375778  -1.953190
 N     -3.235219  -0.054647  -1.054234
 C      1.805158  -1.643339  -0.381533
 C      1.450415  -1.854874  -1.777046
 C      2.532077  -2.583980  -2.398636
 H      2.571406  -2.885648  -3.454529
 C      3.550260  -2.821898  -1.412413
 H      4.509457  -3.328547  -1.583767
 C      3.117953  -2.230981  -0.175837
 H      3.685851  -2.228869   0.762533
 C      2.980934   1.153715  -2.200897
 H      2.023367   1.691737  -2.297862
 C      3.651147   0.404507  -3.232265
 H      3.297477   0.264833  -4.262717
 C      4.856433  -0.152414  -2.672605
 H      5.576894  -0.793990  -3.197591
 C      4.936012   0.255513  -1.292894
 H      5.726961  -0.020872  -0.582950
 C      3.778696   1.058371  -1.001499
 H      3.531758   1.496751  -0.025787
 C      0.413313  -2.475604   1.979761
 C     -0.094397  -2.291388   3.287303
 H     -0.287614  -1.274760   3.664805
 C     -0.355719  -3.396349   4.114936
 H     -0.746345  -3.234589   5.132501
```

```
 C     -0.118449  -4.703245   3.648422
 H     -0.321298  -5.569284   4.298392
 C      0.377507  -4.895148   2.347325
 H      0.564898  -5.913861   1.971396
 C      0.640366  -3.789219   1.516964
 H      1.028456  -3.956884   0.500658
 C      1.719042   0.044038   2.014737
 C      1.505194   1.433148   2.099795
 H      0.715100   1.892437   1.487365
 C      2.288158   2.225957   2.960589
 H      2.105518   3.310480   3.013246
 C      3.294604   1.636547   3.744211
 H      3.905879   2.256428   4.419474
 C      3.510874   0.247075   3.671540
 H      4.290614  -0.225356   4.290298
 C      2.723102  -0.545421   2.820137
 H      2.879195  -1.635177   2.803451
 C      0.144640  -1.576441  -2.484094
 H      0.409144  -1.346418  -3.543733
 C     -1.655934   0.057330  -2.889974
 H     -1.534182   1.159102  -2.988024
 H     -1.501569  -0.371351  -3.902783
 C     -3.031414  -0.261655  -2.387516
 C     -4.499775  -0.281295  -0.556068
 C     -5.529685  -0.797977  -1.385442
 H     -6.528609  -0.969185  -0.963845
 C     -5.291305  -1.033081  -2.740228
 C     -4.021061  -0.741695  -3.258968
 H     -3.781535  -0.901388  -4.320333
 C     -0.740556  -2.835071  -2.501941
 H     -0.200969  -3.656144  -3.014295
 H     -0.990112  -3.169517  -1.477039
 H     -1.687823  -2.661138  -3.051241
 C     -1.728732   0.365367   1.640680
 H     -0.842184   1.507985  -0.189831
 H      0.086252   0.429925  -1.967187
 C     -2.007000  -1.812952   0.397421
 O     -2.559093  -2.820304   0.675622
 C     -0.564566   2.794781  -1.145043
 C      0.143957   3.750560  -0.200870
 C     -2.041358   3.297171  -1.127870
 C     -0.798222   4.503081   0.534093
 C      1.515050   4.052189  -0.132899
 C     -2.205626   4.044519   0.213233
 C     -0.372164   5.540806   1.380051
 C      1.941326   5.098642   0.705121
 H      2.238129   3.491275  -0.743981
 H     -2.555511   3.354673   1.012841
 C      1.004058   5.832118   1.463649
 H     -1.102815   6.136151   1.952076
 H      3.011300   5.354961   0.763363
 H      1.350176   6.652981   2.112346
 H     -2.933694   4.880801   0.171857
 O      0.008619   2.353037  -2.192765
 H     -2.117278   4.019404  -1.972883
 H     -2.781992   2.494463  -1.299869
 H     -6.092806  -1.420274  -3.388465
 N     -4.751957  -0.005496   0.788507
 C     -5.876560  -0.673183   1.431649
 C     -4.585769   1.381710   1.221533
 H     -5.881729  -1.751839   1.182157
 H     -5.760452  -0.575292   2.530342
 H     -6.870714  -0.236917   1.161803
 H     -4.378835   1.425175   2.308393
 H     -3.731942   1.833032   0.690507
 H     -5.503010   1.982695   1.004145

4/TSHy-mRSS-re-8ke
Frequencies, energies and thermodynamic properties:
 Lowest Vibrational Mode (1/cm) =                          -460.0827
 E(RB-P86) (a.u.) =                                        -4809.14386748
 Thermal correction to Enthalpy (a.u.) =                     0.786413
 Thermal correction to Gibbs Free Energy (a.u.) =            0.656360
 Total Entropy (cal/Kmol) =                                  273.721
 E(RPBE1PBE) (a.u.) =                                      -4808.46053105
Optimised cartesian coordinates (Angstrom):
 Fe    -3.720590   0.013873  -1.339218
```

Mn	1.177188	-0.981120	0.091867
P	-0.775588	-0.186162	0.943399
O	2.237647	-0.426727	2.783981
O	0.384547	-3.803501	0.433965
N	0.551243	-0.898182	-1.935836
N	2.971942	-1.625894	-0.824107
C	-1.925969	0.422218	-0.367827
C	-1.723961	0.092170	-1.774008
C	-2.460277	1.057415	-2.560940
H	-2.526845	1.088071	-3.655381
C	-3.146018	1.948598	-1.665047
H	-3.812652	2.770164	-1.959359
C	-2.834849	1.552616	-0.319179
H	-3.193656	2.044491	0.593814
C	-4.636717	-1.697592	-0.621932
H	-4.234690	-2.384374	0.133259
C	-4.430980	-1.791400	-2.041803
H	-3.850176	-2.567863	-2.557124
C	-5.117701	-0.688708	-2.665172
H	-5.141156	-0.466195	-3.740308
C	-5.748970	0.086442	-1.626932
H	-6.339398	1.000649	-1.773304
C	-5.450594	-0.536240	-0.362829
H	-5.793490	-0.187487	0.620197
C	-0.673734	1.286820	2.105091
C	-0.611232	1.081147	3.503795
H	-0.658868	0.062496	3.915236
C	-0.499467	2.166815	4.389993
H	-0.455324	1.978903	5.474924
C	-0.456808	3.482413	3.896586
H	-0.383735	4.335441	4.590063
C	-0.505334	3.697901	2.508441
H	-0.465802	4.720568	2.102906
C	-0.598517	2.611027	1.619598
H	-0.619438	2.808260	0.538374
C	-1.770169	-1.299853	2.072124
C	-1.137006	-2.307701	2.831719
H	-0.060922	-2.494566	2.709495
C	-1.866376	-3.068846	3.764988
H	-1.353930	-3.851463	4.347028
C	-3.237823	-2.829731	3.956684
H	-3.808832	-3.426466	4.685773
C	-3.874648	-1.815948	3.216754
H	-4.947126	-1.612261	3.366480
C	-3.144794	-1.054903	2.287341
H	-3.650663	-0.263226	1.715776
C	-0.899588	-1.066981	-2.304783
H	-1.229641	-1.992465	-1.784866
C	1.417891	-1.845791	-2.668113
H	1.041855	-2.879710	-2.488852
H	1.401595	-1.673670	-3.763886
C	2.831871	-1.764792	-2.172594
C	4.237580	-1.764650	-0.294859
C	5.371691	-1.848098	-1.142194
H	6.371302	-1.936210	-0.696086
C	5.213967	-1.886559	-2.529919
C	3.914624	-1.881755	-3.056353
H	3.729835	-1.954129	-4.137825
C	-1.128073	-1.255700	-3.816541
H	-2.214228	-1.294088	-4.025480
H	-0.696540	-0.418646	-4.403887
H	-0.692930	-2.202715	-4.187851
C	1.855249	-0.692002	1.695582
C	0.673702	-2.659431	0.326430
H	0.893182	0.053049	-2.248792
H	1.664735	0.678797	-0.264182
C	2.359791	1.678934	-1.404837
C	1.724499	2.990717	-1.000893
C	3.758389	1.710781	-0.730320
C	2.495879	3.617962	0.001130
C	0.646268	3.648717	-1.614764
C	3.623769	2.704081	0.444119
C	2.184780	4.926389	0.406373
C	0.332439	4.957450	-1.203671
H	0.079310	3.147619	-2.415995
H	4.559576	3.254141	0.674127
C	1.099912	5.590702	-0.201658

H	2.786278	5.436361	1.176891
H	-0.502914	5.499583	-1.675461
H	0.855108	6.622290	0.099323
H	3.329319	2.164841	1.373317
O	2.133263	1.152944	-2.536511
H	4.124841	0.710230	-0.445261
H	4.452727	2.118732	-1.500419
H	6.092460	-1.962741	-3.189379
N	4.382619	-1.830394	1.092754
C	5.648950	-1.409967	1.677261
C	3.804530	-2.994731	1.770901
H	6.005004	-0.474754	1.203389
H	5.488401	-1.207775	2.756482
H	6.456068	-2.180726	1.598569
H	3.566914	-2.739618	2.823353
H	2.872034	-3.310834	1.273731
H	4.520522	-3.853036	1.765588

4/TSHy-mRSS-si-8ke
Frequencies, energies and thermodynamic properties:

Lowest Vibrational Mode (1/cm) =	-394.7059
E(RB-P86) (a.u.) =	-4809.14425824
Thermal correction to Enthalpy (a.u.) =	0.786084
Thermal correction to Gibbs Free Energy (a.u.) =	0.656668
Total Entropy (cal/Kmol) =	272.379
E(RPBE1PBE) (a.u.) =	-4808.45749464

Optimised cartesian coordinates (Angstrom):

Fe	-3.703186	-1.294672	-1.104234
Mn	0.925042	0.157710	0.949618
P	-1.106130	0.937322	0.243005
O	2.116009	2.684801	0.046621
O	0.508587	1.088108	3.716751
N	0.264468	-1.859314	0.790021
N	2.660744	-0.841932	1.609067
C	-1.901477	-0.278612	-0.899173
C	-1.742787	-1.698823	-0.629403
C	-2.148290	-2.422052	-1.812267
H	-2.156723	-3.512158	-1.936202
C	-2.583389	-1.470059	-2.797438
H	-2.980056	-1.707851	-3.793667
C	-2.455467	-0.150531	-2.233655
H	-2.736329	0.785178	-2.731655
C	-5.020818	-0.929513	0.447732
H	-4.775229	-0.468285	1.411944
C	-4.975493	-2.335007	0.147662
H	-4.698163	-3.134464	0.847362
C	-5.352859	-2.509353	-1.231799
H	-5.397157	-3.463404	-1.773992
C	-5.636877	-1.208307	-1.782584
H	-5.935094	-0.998147	-2.818474
C	-5.427894	-0.231389	-0.745220
H	-5.549024	0.854620	-0.848214
C	-1.050914	2.570218	-0.648983
C	-1.034716	3.753583	0.128633
H	-1.158830	3.696111	1.221539
C	-0.863764	5.011017	-0.473378
H	-0.862117	5.917416	0.152978
C	-0.691859	5.113019	-1.866005
H	-0.561687	6.098982	-2.339618
C	-0.676572	3.943924	-2.645316
H	-0.529437	4.006245	-3.735355
C	-0.845020	2.683622	-2.041472
H	-0.806129	1.784711	-2.671552
C	-2.477921	1.271370	1.484045
C	-2.502153	0.596646	2.723064
H	-1.687384	-0.091864	2.988053
C	-3.553773	0.799593	3.636983
H	-3.549782	0.261976	4.598556
C	-4.596820	1.689486	3.328614
H	-5.415136	1.857239	4.046718
C	-4.589485	2.361386	2.091775
H	-5.405707	3.055267	1.834434
C	-3.544952	2.148496	1.175875
H	-3.563253	2.676969	0.210667
C	-1.182189	-2.271923	0.659912
H	-1.717445	-1.804987	1.514187
C	0.979712	-2.577921	1.862734

```
 H      0.515131  -2.316265   2.841605
 H      0.922636  -3.681556   1.755763
 C      2.423944  -2.161607   1.867826
 C      3.957503  -0.389904   1.720684
 C      5.021410  -1.294970   1.966846
 H      6.045293  -0.905052   2.044215
 C      4.768649  -2.653337   2.163525
 C      3.437313  -3.094401   2.131376
 H      3.175485  -4.148871   2.301153
 C     -1.394044  -3.794391   0.732231
 H     -2.453619  -4.028415   0.512852
 H     -0.765491  -4.329570  -0.009346
 H     -1.171335  -4.200129   1.737679
 C      1.673778   1.670989   0.465215
 C      0.601265   0.673984   2.607381
 H      0.728486  -2.144844  -0.123823
 H      1.516250  -0.144810  -0.651040
 C      1.953677  -1.227822  -1.884194
 C      3.397536  -0.813060  -2.011868
 C      1.220062  -0.412530  -2.980340
 C      3.501436   0.325087  -2.842961
 C      4.539314  -1.486059  -1.548095
 C      2.121689   0.812062  -3.246386
 H      1.197014  -1.074092  -3.877335
 C      4.767163   0.816071  -3.203289
 C      5.806375  -0.998271  -1.918226
 H      4.431614  -2.389863  -0.927606
 H      1.823468   1.663886  -2.594380
 C      5.917496   0.147026  -2.736605
 H      4.864886   1.698115  -3.857600
 H      6.717819  -1.516411  -1.578921
 H      6.915742   0.514478  -3.024847
 H      2.084043   1.177335  -4.293465
 H      0.172457  -0.184166  -2.713451
 O      1.592979  -2.377012  -1.494704
 H      5.593326  -3.353600   2.368026
 N      4.216341   0.979704   1.594411
 C      5.516076   1.382101   1.071645
 C      3.759566   1.854795   2.678129
 H      5.798191   0.748004   0.209577
 H      5.441033   2.431161   0.716656
 H      6.335060   1.348558   1.834005
 H      3.593341   2.880668   2.290891
 H      2.812364   1.491772   3.108290
 H      4.522154   1.904823   3.494750

4/TSH2-fRSR
Frequencies, energies and thermodynamic properties:
 Lowest Vibrational Mode (1/cm) =                         -572.0868
 E(RB-P86) (a.u.) =                                       -4386.42188423
 Thermal correction to Enthalpy (a.u.) =                   0.631455
 Thermal correction to Gibbs Free Energy (a.u.) =          0.519964
 Total Entropy (cal/Kmol) =                                234.654
 E(RPBE1PBE) (a.u.) =                                     -4385.73901825
Optimised cartesian coordinates (Angstrom):
 Fe     3.408353  -0.664479   0.379796
 Mn    -1.031824  -0.464315  -1.519819
 P      0.021776   0.537518   0.225612
 O     -2.044639   2.095201  -2.608281
 O      1.149727  -0.061808  -3.434241
 N     -0.414409  -2.322155  -0.805716
 N     -2.764736  -1.087271  -0.409154
 C      1.409222  -0.496259   0.847871
 C      1.717395  -1.852836   0.395236
 C      2.725471  -2.373907   1.289318
 H      3.191494  -3.365124   1.219309
 C      3.055681  -1.370063   2.265430
 H      3.807539  -1.463985   3.060808
 C      2.256555  -0.207505   1.992296
 H      2.277935   0.732916   2.558100
 C      3.865870   0.174221  -1.449591
 H      3.142473   0.417828  -2.237381
 C      4.536897  -1.088354  -1.291563
 H      4.420627  -1.966845  -1.940452
 C      5.369505  -1.007292  -0.118205
 H      5.995867  -1.813628   0.286580
 C      5.213996   0.309444   0.447237
```

```
 H      5.702196   0.681232   1.358209
 C      4.282012   1.038766  -0.373307
 H      3.933844   2.065737  -0.202393
 C     -1.034415   0.815070   1.738206
 C     -2.075312   1.769218   1.634715
 H     -2.214031   2.329474   0.695464
 C     -2.927996   2.020068   2.722193
 H     -3.725131   2.774801   2.627993
 C     -2.766036   1.309195   3.926980
 H     -3.435201   1.504920   4.779877
 C     -1.746882   0.347433   4.033213
 H     -1.613193  -0.215715   4.970801
 C     -0.885824   0.100833   2.946318
 H     -0.085945  -0.648253   3.049350
 C      0.778543   2.234760   0.043688
 C      1.120355   2.736694  -1.230976
 H      0.913354   2.143389  -2.131908
 C      1.731112   3.997152  -1.365371
 H      1.987826   4.371955  -2.369020
 C      2.008334   4.776251  -0.228367
 H      2.484594   5.763885  -0.334818
 C      1.665053   4.289248   1.046205
 H      1.870442   4.893941   1.944036
 C      1.050876   3.031900   1.180750
 H      0.771236   2.675155   2.184184
 C      1.023523  -2.598517  -0.755980
 H      1.444923  -2.212679  -1.707581
 C     -1.124200  -2.712141   0.392348
 H     -1.241377  -3.822345   0.494858
 H     -0.586732  -2.404681   1.333468
 C     -2.495421  -2.095488   0.465736
 C     -4.022147  -0.527633  -0.394882
 C     -4.959052  -0.873085   0.608228
 C     -4.646760  -1.867370   1.541533
 C     -3.411136  -2.517330   1.446446
 C      1.337732  -4.110541  -0.718202
 H      2.423558  -4.297505  -0.849541
 H      1.030420  -4.581796   0.238644
 H      0.800337  -4.620437  -1.543438
 C     -1.712519   1.055799  -2.155607
 C      0.322358  -0.211175  -2.597633
 H     -1.245698  -2.014027  -2.149064
 H     -1.670902  -1.472611  -2.752910
 H     -5.951178  -0.402456   0.610183
 H     -3.133076  -3.331133   2.132341
 H     -5.380315  -2.157843   2.309953
 N     -4.355290   0.382977  -1.402909
 C     -5.384202   1.375170  -1.121916
 H     -5.293400   2.195653  -1.863512
 H     -6.425465   0.972803  -1.194534
 H     -5.240141   1.807163  -0.112792
 C     -4.425316  -0.136792  -2.772171
 H     -5.426125  -0.589159  -2.979121
 H     -4.251047   0.683936  -3.497303
 H     -3.647202  -0.904607  -2.928217
```

4/TSH2s-fRSR

```
Frequencies, energies and thermodynamic properties:
 Lowest Vibrational Mode (1/cm) =                        -737.0869
 E(RB-P86) (a.u.) =                                      -4541.36619420
 Thermal correction to Enthalpy (a.u.) =                  0.713567
 Thermal correction to Gibbs Free Energy (a.u.) =         0.589582
 Total Entropy (cal/Kmol) =                               260.948
 E(RPBE1PBE) (a.u.) =                                    -4540.68941830
Optimised cartesian coordinates (Angstrom):
 Fe    -3.475144   0.847778   0.635999
 Mn     1.078789   0.524487  -1.071556
 P     -0.377742  -0.854764   0.007512
 O      1.616208  -1.599437  -3.059967
 O     -0.880611   1.558319  -2.994324
 N      0.700694   1.982655   0.389287
 N      2.710436   0.182712   0.264609
 C     -1.617215   0.051539   1.012261
 C     -1.609936   1.494816   1.234136
 C     -2.600245   1.772127   2.248190
 H     -2.859959   2.765435   2.635598
 C     -3.224728   0.537674   2.642266
```

H	-4.034508	0.429696	3.376453
C	-2.632027	-0.524650	1.877307
H	-2.893905	-1.588408	1.943905
C	-3.855847	1.045511	-1.381955
H	-3.101899	1.031360	-2.178841
C	-4.299004	2.213825	-0.669331
H	-3.947280	3.241447	-0.832335
C	-5.266978	1.796567	0.312916
H	-5.778429	2.448999	1.033321
C	-5.423764	0.368086	0.204171
H	-6.076698	-0.257759	0.827292
C	-4.549221	-0.097246	-0.840854
H	-4.418568	-1.139356	-1.160165
C	0.420889	-2.009375	1.234041
C	1.245625	-3.039620	0.721280
H	1.369514	-3.152771	-0.368035
C	1.896636	-3.932680	1.587919
H	2.522293	-4.738464	1.171926
C	1.749453	-3.800600	2.982187
H	2.260533	-4.500853	3.661750
C	0.946167	-2.770916	3.500546
H	0.824392	-2.658841	4.589777
C	0.284237	-1.880538	2.632957
H	-0.354715	-1.090191	3.055587
C	-1.407992	-2.069524	-0.966416
C	-1.696270	-1.847025	-2.330411
H	-1.281644	-0.972160	-2.848857
C	-2.518605	-2.738015	-3.044070
H	-2.729092	-2.547823	-4.108506
C	-3.064980	-3.865420	-2.406309
H	-3.707003	-4.563440	-2.966705
C	-2.779290	-4.100273	-1.048959
H	-3.196132	-4.983852	-0.539738
C	-1.954048	-3.213037	-0.336342
H	-1.726771	-3.423127	0.720220
C	-0.682437	2.501992	0.556581
H	-1.057123	2.668684	-0.473894
C	1.313511	1.619951	1.667199
H	1.587558	2.510845	2.282027
H	0.601782	1.039759	2.306999
C	2.537882	0.765058	1.484895
C	3.813756	-0.618947	0.076718
C	4.676854	-0.933665	1.152267
C	4.466066	-0.360410	2.410085
C	3.397416	0.528838	2.571996
C	-0.703565	3.874757	1.260027
H	-1.714182	4.328366	1.207649
H	-0.420214	3.807854	2.330880
H	0.004948	4.555845	0.747081
C	1.474641	-0.760493	-2.242181
C	-0.146596	1.111490	-2.179449
H	2.209917	1.390973	-1.993310
H	2.011055	2.094597	-1.548228
H	1.393587	2.940492	-0.180406
O	1.997256	3.701395	-0.899532
C	3.269947	4.031471	-0.400085
H	3.225507	4.320850	0.684461
H	3.978734	3.158557	-0.442743
H	3.201857	1.025010	3.533943
H	5.544245	-1.583790	0.975109
H	5.150110	-0.580204	3.244572
N	4.064211	-1.119564	-1.207891
C	4.734163	-2.409084	-1.320935
H	4.537175	-2.820211	-2.333028
H	5.844000	-2.348597	-1.195291
H	4.329680	-3.121061	-0.576281
C	4.511257	-0.162958	-2.224578
H	5.623857	-0.059038	-2.214036
H	4.198937	-0.501003	-3.234361
H	4.072560	0.832528	-2.039270
C	3.885646	5.191749	-1.187957
H	3.229841	6.087011	-1.135776
H	4.885996	5.469372	-0.791228
H	4.001602	4.921638	-2.259403

small-mol/8al
Frequencies, energies and thermodynamic properties:

```
 Lowest Vibrational Mode (1/cm) =                         107.4633
 E(RB-P86) (a.u.) =                                      -423.896842178
 Thermal correction to Enthalpy (a.u.) =                  0.172666
 Thermal correction to Gibbs Free Energy (a.u.) =         0.130691
 Total Entropy (cal/Kmol) =                               88.346
 E(RPBE1PBE) (a.u.) =                                    -423.881563844
Optimised cartesian coordinates (Angstrom):
 C     -0.344837   0.933846   0.044309
 C      0.073518  -0.407502   0.184895
 C     -0.852349  -1.462018   0.162888
 C     -2.219337  -1.161945  -0.000618
 C     -2.642963   0.176253  -0.134683
 C     -1.709090   1.232424  -0.112662
 C      0.854637   1.864671   0.099607
 C      2.049117   0.913127  -0.165103
 C      1.586979  -0.479091   0.352063
 H     -0.509083  -2.505073   0.259590
 H     -2.961814  -1.975796  -0.028043
 H     -3.714424   0.398241  -0.265944
 H     -2.048109   2.275207  -0.228313
 H      0.789478   2.705687  -0.621872
 H      3.000320   1.249818   0.293340
 O      2.216197  -1.598281  -0.259470
 H      0.934943   2.323860   1.111741
 H      2.218420   0.823100  -1.261759
 H      1.830539  -0.579002   1.436039
 H      1.956109  -1.588373  -1.203195

small-mol/8ke
Frequencies, energies and thermodynamic properties:
 Lowest Vibrational Mode (1/cm) =                         77.6374
 E(RB-P86) (a.u.) =                                      -422.707662993
 Thermal correction to Enthalpy (a.u.) =                  0.149568
 Thermal correction to Gibbs Free Energy (a.u.) =         0.108052
 Total Entropy (cal/Kmol) =                               87.378
 E(RPBE1PBE) (a.u.) =                                    -422.687390854
Optimised cartesian coordinates (Angstrom):
 C     -0.239280   0.904308  -0.000033
 C      0.093984  -0.468025  -0.000001
 C     -0.893069  -1.473639  -0.000112
 C     -2.239981  -1.084556  -0.000256
 C     -2.582051   0.288723  -0.000289
 C     -1.592237   1.289152  -0.000182
 C      1.002911   1.777816   0.000105
 C      2.186726   0.781022   0.000280
 C      1.573223  -0.631466   0.000153
 H     -0.597728  -2.535223  -0.000082
 H     -3.037466  -1.844286  -0.000343
 H     -3.645072   0.579156  -0.000406
 H     -1.877964   2.353473  -0.000212
 H      1.014217   2.446255  -0.887331
 H      2.846492   0.885009   0.887722
 O      2.196924  -1.689468   0.000262
 H      1.013989   2.446304   0.887506
 H      2.846793   0.885041  -0.886934

small-mol/EtOH
Frequencies, energies and thermodynamic properties:
 Lowest Vibrational Mode (1/cm) =                         245.6449
 E(RB-P86) (a.u.) =                                      -154.922969451
 Thermal correction to Enthalpy (a.u.) =                  0.082426
 Thermal correction to Gibbs Free Energy (a.u.) =         0.051666
 Total Entropy (cal/Kmol) =                               64.739
 E(RPBE1PBE) (a.u.) =                                    -154.927492887
Optimised cartesian coordinates (Angstrom):
 C      1.228762  -0.220241   0.000005
 C     -0.089034   0.542785   0.000003
 H      2.089062   0.479844   0.000002
 H      1.307054  -0.868131   0.898094
 H      1.307052  -0.868130  -0.898087
 H     -0.134881   1.211056   0.896116
 H     -0.134861   1.211081  -0.896093
 O     -1.159836  -0.401017  -0.000021
 H     -1.993111   0.107152   0.000088

small-mol/H2
Frequencies, energies and thermodynamic properties:
```

```
 Lowest Vibrational Mode (1/cm) =                         4268.9553
 E(RB-P86) (a.u.) =                                      -1.17256159454
 Thermal correction to Enthalpy (a.u.) =                  0.013030
 Thermal correction to Gibbs Free Energy (a.u.) =        -0.001825
 Total Entropy (cal/Kmol) =                               31.264
 E(RPBE1PBE) (a.u.) =                                    -1.16813917680
Optimised cartesian coordinates (Angstrom):
 H      0.000000   0.000000   0.383930
 H      0.000000   0.000000  -0.383930

small-mol/HCO3--EtOH
Frequencies, energies and thermodynamic properties:
 Lowest Vibrational Mode (1/cm) =                         29.6699
 E(RB-P86) (a.u.) =                                      -419.295512044
 Thermal correction to Enthalpy (a.u.) =                  0.114294
 Thermal correction to Gibbs Free Energy (a.u.) =         0.068818
 Total Entropy (cal/Kmol) =                               95.712
 E(RPBE1PBE) (a.u.) =                                    -419.333598467
Optimised cartesian coordinates (Angstrom):
 O      1.160274  -0.982590   0.073353
 O      1.250521   1.289854  -0.188489
 C      1.733699   0.153566  -0.025905
 O      3.140538   0.099451   0.070447
 H      3.337099  -0.852481   0.188754
 H     -0.428821  -0.980945  -0.198677
 O     -1.433119  -0.980433  -0.363688
 C     -1.971039   0.040152   0.448474
 C     -3.331434   0.478563  -0.092124
 H     -1.282150   0.920197   0.489817
 H     -2.105953  -0.293179   1.516474
 H     -3.789025   1.263961   0.546220
 H     -3.230406   0.880364  -1.123143
 H     -4.033816  -0.381855  -0.135105

small-mol/HCO3--EtOH3
Frequencies, energies and thermodynamic properties:
 Lowest Vibrational Mode (1/cm) =                         15.4998
 E(RB-P86) (a.u.) =                                      -729.182541817
 Thermal correction to Enthalpy (a.u.) =                  0.283587
 Thermal correction to Gibbs Free Energy (a.u.) =         0.207001
 Total Entropy (cal/Kmol) =                               161.189
 E(RPBE1PBE) (a.u.) =                                    -729.218561433
Optimised cartesian coordinates (Angstrom):
 O     -1.112241  -0.643757  -0.111589
 O      0.124252   1.249107   0.016736
 C     -0.013285   0.022983  -0.206999
 O      1.102582  -0.677677  -0.590081
 H      0.816187  -1.632349  -0.737225
 H     -2.471097   0.196533   0.419189
 O     -3.299962   0.693976   0.692348
 C     -3.534953   1.663218  -0.308468
 C     -4.658126   2.596735   0.134855
 H     -3.821912   1.195666  -1.289823
 H     -2.614736   2.265213  -0.520408
 H     -4.872860   3.364727  -0.637347
 H     -5.591934   2.024959   0.323595
 H     -4.384757   3.116657   1.077640
 H     -0.809332  -2.192138  -0.487391
 O     -0.232148  -3.001976  -0.712685
 C     -0.041280  -3.738772   0.497519
 H     -1.014369  -4.158810   0.850828
 H      0.332402  -3.073049   1.313834
 C      0.946939  -4.872374   0.256476
 H      1.938921  -4.478762  -0.050098
 H      1.085098  -5.476655   1.176752
 H      0.582025  -5.545172  -0.547721
 H      1.585462   1.983815  -0.312486
 O      2.461795   2.454255  -0.468317
 C      3.377860   1.929292   0.468296
 H      3.446535   0.812502   0.400720
 H      3.070926   2.146926   1.527466
 C      4.759818   2.530521   0.225881
 H      4.729320   3.636489   0.327169
 H      5.503940   2.136487   0.949324
 H      5.114113   2.295928  -0.800673
```
